# Enhanced Structural Understanding of Dissolved Organic Matter through Comparative LC/MS2 Analysis with Synthetic Carboxylate Rich Alicyclic Molecules

**DOI:** 10.1021/acs.analchem.5c02665

**Published:** 2025-08-21

**Authors:** Jeffrey A. Hawkes, Agnes D. Flygare, Lindon W. K. Moodie, Alexander J. Craig

**Affiliations:** † Department of Chemistry BMC, 8097 Uppsala University, Uppsala 752 37, Sweden; ‡ Department of Medicinal Chemistry, 8097 Uppsala University, Uppsala 752 37, Sweden

## Abstract

Dissolved organic matter (DOM) is one of the most complex chemical mixtures known, with its chemical composition having long puzzled biogeochemists. Identifying the chemical structures within DOM is essential for unraveling its origins and environmental fate. However, DOM’s complexity has impeded structural elucidation, and molecules with accurate functional group compositions for recalcitrant DOM are poorly represented in the synthetic and isolative literature. Consequently, hypothesized DOM compounds are derived from models that inadequately represent true structures. To address this, carboxylic-acid-only CRAM analogues were previously synthesized but failed to replicate the extensive fragmentation observed in marine DOM during tandem mass spectrometry (MS2). Here, we prepared CRAM analogues with varied oxygen functionalities to enable more diverse fragmentation pathways. Liquid chromatography (LC) studies showed that functional group composition better predicted LC polarity than the O/C ratio and that alcohols represented early eluting DOM profiles, while ethers, ketones, and lactones better represented mid-eluting isomers. MS2 studies revealed that the incorporation of α-hydroxy ketones and 1,2-diols led to the most extensive fragmentation. Ether and ester functionalities were labile even at low fragmentation energy, indicating that such groups are likely contributors to core marine DOM carbon backbones and contribute to the extensive fragmentation observed for natural DOM in all MS2 experiments. The data gathered within this work suggest that the widely discussed all carbon-backbone alicyclic model of CRAM is incompatible with the MS2 fragmentation data of DOM.

## Graphical abstract

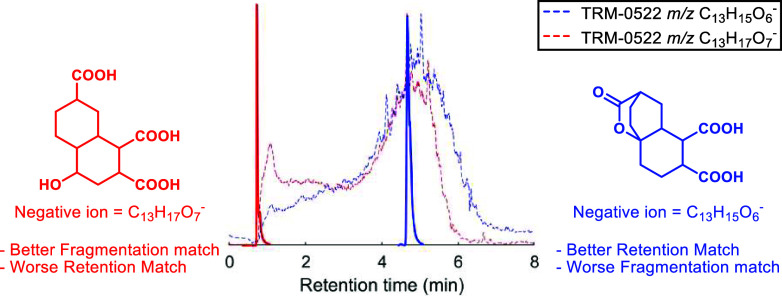

## Introduction

Dissolved organic matter (DOM) is one of the largest reservoirs of organic carbon on the planet and plays a vital role in carbon transport between aquatic microorganisms. Composed predominantly of small organic molecules extruded from living or dead organisms, DOM represents one of the most chemically complex mixtures known, with analytical techniques showing it contains, at minimum, hundreds of thousands of individual compounds.^1–8^ This molecular diversity has long impeded efforts to accurately characterize its chemical structures, limiting our understanding of its origins and environmental role.^9^ Particularly enigmatic is the predominantly marine recalcitrant DOM (RDOM), which persists for millennia and exhibits remarkable physical, chemical, and biological stability.^10^ In contrast, the labile DOM fraction is rapidly turned over within hours to days and primarily consists of transient biological metabolites.^11^ While metabolites have been directly identified within labile DOM,^12–16^ the functional group composition of RDOM is unlike compounds known from biosynthetic pathways, presenting a significant challenge when it comes to unravelling its structural composition, and in turn its source and fate.

Efforts to constrain the chemical classes of RDOM have led to descriptions such as carboxylate-rich alicyclic molecules (CRAM),^17^ material derived from linear terpenoids,^18^ acetyl- or heteropolysaccharides,^17,19,20^ and polycarboxylic acid polyaromatic molecules.^21^ However, these classifications are primarily based on the nuclear magnetic resonance (NMR) and high-resolution mass spectrometry (HRMS) data of bulk DOM. While NMR and HRMS are the gold standards for molecular structural analysis, their application to complex mixtures like DOM presents significant challenges.^9,22–24^ NMR spectroscopy provides broad and poorly differentiated regions for DOM, even with advanced 2D techniques.^22,25^ Similarly, HRMS and tandem MS (MS2) techniques such as higher energy collisional dissociation (HCD) struggle to differentiate isomers with identical molecular formulas, even when paired with liquid chromatography (LC) or ion mobility.^3,7,26^ Consequently, the proposed structural classes reflect averaged representations of dominant functional groups and carbon types rather than precise depictions of functional group arrangements or backbone connectivity. To accurately determine RDOM fluxes and constrain its chemical origins—whether lignin,^27,28^ terpenoid,^17,18,29^ or polysaccharide-derived^19,20^ —greater insight into these specific structural features is critical.

Previously, we disclosed compounds (Figure 1a, **1**–**4**) that mimicked the key features of hypothesized CRAM, including fused alicyclic rings, multiple carboxylic acids, and a predominantly reduced carbon backbone.^30^ Subsequent HCD studies at multiple voltages revealed differences between the data of these molecules and that of DOM. Low-energy (35 V) HCD experiments led solely to losses of H_2_O and CO_2_ for synthetic compounds, which was consistent with the many previous collision induced dissociation studies on DOM in the field, with these losses typically attributed to carboxylic acids.^1–3,31^ However, high-energy (75 V) HCD fragmentation left the carbon scaffold of our decalin backbone analogues largely intact while causing extensive breakdown of DOM carbon backbone structures, indicating something fundamentally dissimilar between the structures of these alicyclic CRAM-like molecules and those found in DOM.

**Figure 1:**
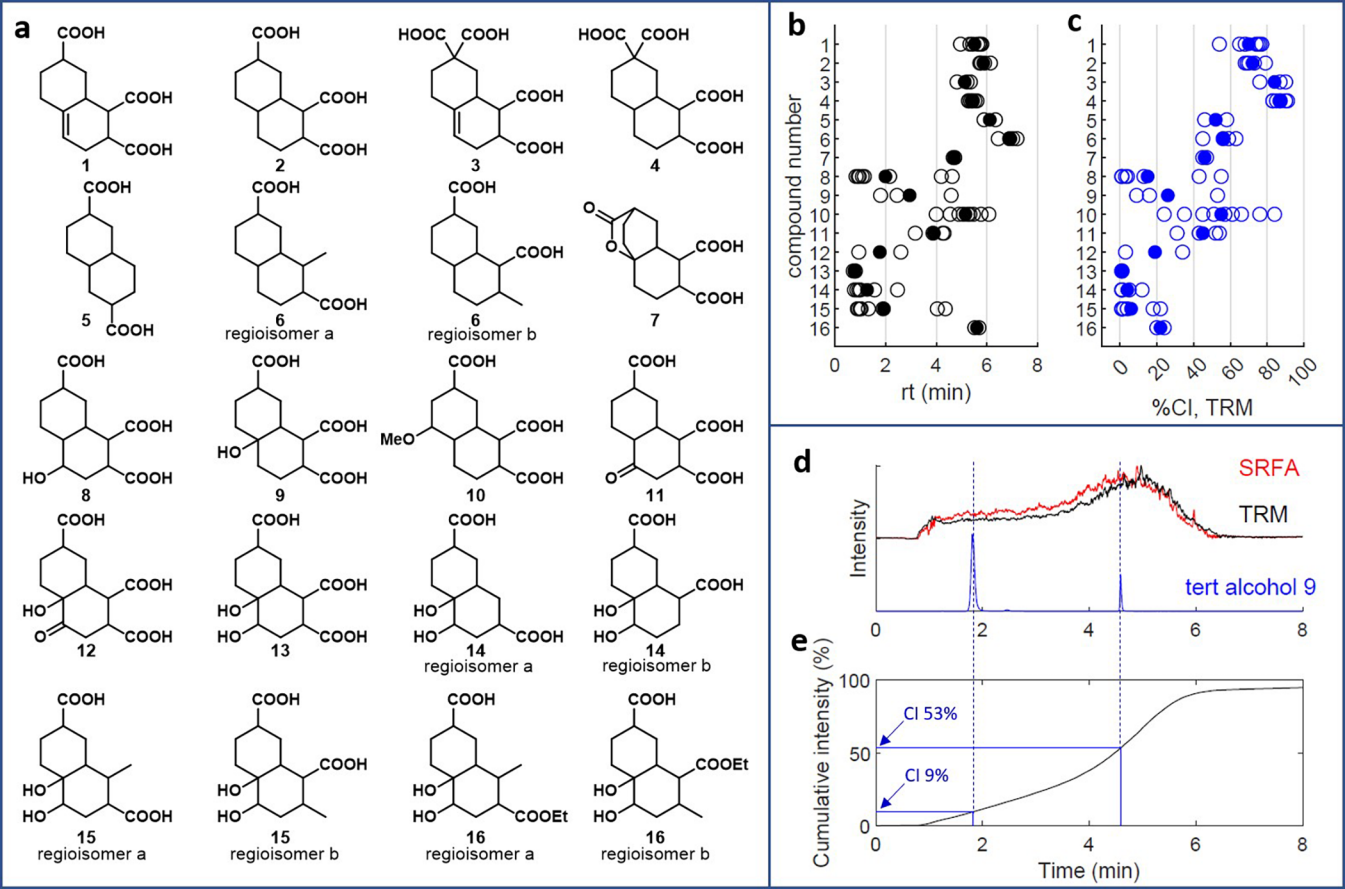
(a) Structures of CRAM analogues with O/C ratios listed in blue **1**–**16**. (b) Compound retention times. (c) Compound CI plotted by compound number. All detected isomers are shown as unfilled circles; the mean of detected isomers are shown as filled circles. (d) Depiction of the CI metric, XIC of *m*/*z* 285.098 in TRM (black), SRFA (red) and the tertiary alcohol **9**. (e) Trace of cumulative intensity for TRM as a black line, which eventually reaches 100% and shows the equivalent points at which the two most intense isomers of **9** elute so that the CI value can be read from the *y* axis.

Given the extensive high-energy HCD fragmentation of the molecules in DOM, we speculated that varied oxygen functionalities might enable additional points of charge stabilization, which could facilitate further carbon–carbon bond fragmentations.^32–34^ Additionally, for almost any given molecular formula in DOM, peaks exist across a broad range of LC retention times. However, little defines what constitutes the functional group features of an early or late eluting isomer. To test the effect of different functional groups on both MS2 fragmentation and LC retention behavior, we prepared 12 synthetic compounds based on our initial CRAM decalin scaffold which also contain additional oxygen functionalities, including alcohols, 1,2 diols, ketones, α-hydroxy ketones, ethers, lactones, and esters. This allows for direct comparison between the previously synthesized and new molecules, but also provides relative comparison between materials where the primary difference is the inclusion of different functional groups. As such, the information gained from LC and MS2 studies is generally transferable to other carbon scaffolds, leading to insights into the relative differences in functional group type and abundance in isomers of natural DOM. Ultimately, this work shows that the type and placement of oxygen functional groups within CRAM-like molecules is critical in determining their MS2 fragmentation behavior and LC retention profiles.

## Methods

Extensive details are available for all experimental methods in the Supporting Information (SI), including their NMR spectral data as well as their LCMS and MS2 data (pages S88–S157).

### Synthetic Information

Synthetic methods are described in the SI, along with spectral data for final CRAM analogues (^1^H, ^13^C, COSY, HSQC, and HMBC NMR, and LCMS, LCMS2, and charged aerosol detection (CAD) spectra). Where noted, compounds were prepared as diastereomeric mixtures, hindering full NMR assignment of individual diastereomers. Fortunately, the nature of this work generating extensive MS2 profiles of these compounds helps to reinforce structural assignments, and CAD data helps in defining their minimum purities.

### Reference Materials

The reference standards SRFA (2S101F) and TRM-0522 ^35^ were used as received and dissolved in 5% acetonitrile in water to a concentration of 5 mg/mL for injection by LCMS.

### Liquid Chromatography–Mass Spectrometry

Liquid chromatography was conducted with a Thermo Vanquish UPLC at a flow rate of 0.5 mL/min from 5% to 100% acetonitrile in water with 0.1% formic acid using a Phenomenex Kinetex C18 column (2.1 × 150 mm, 2.6 μm). Synthetic compounds were dissolved to 100 ppm, while DOM standards were dissolved to 5000 ppm, with both having injection volumes of 10 μL. Mass spectrometry was performed using an Orbitrap Q Exactive (Thermo Fisher), with MS2 experiments being conducted at normalized collision energies of 35 and 75 by HCD experiments using the PRM method.

## Results and Discussion

### Synthetic Design of CRAM Analogues

Molecules were designed based on the parameters Hertkorn et al. described for hypothesized alicyclic CRAM,^17^ which were proposed to be fused alicyclic compounds with predominantly reduced carbon backbones functionalized with several carboxylic acids. These parameters were also used to design our previously disclosed analogues^30^ that were functionalized only with carboxylic acids (hereafter referred to as COOH-CRAM). Similarity of any new compounds to the first set was highly desirable, as while this specific decalin type structure could be poorly represented in real DOM samples, relative comparisons between different functional group compositions and orientations are likely to be transferable between carbon scaffolds. In addition to this, ease of synthetic access was another priority, as the required hydrolysis to CRAM products from ester equivalents was expected to be more problematic with the inclusion of other potentially synthetically sensitive functionalities, such as alcohols, ketones, or ethers. As such, synthetic targets were based predominantly on the chemical transformation of the triester equivalent of alkene CRAM **1** (Figure 1a) where possible, minimizing any potential differences in relative acid regio- and stereochemistry and allowing for bulk modification of a readily accessible starting material.

Choice of oxygen functional group incorporation centered on functionalities that have been described within RDOM chemical classes in the literature, as well as those consistent with the 1D and 2D NMR spectral data of DOM. Alcohols were ideal candidates, described as part of CRAM,^28^ material derived from linear terpenoids,^18^ and heteropolysaccharides,^19,20^ and also frequently identified as contributors from NMR experiments. Similarly, ketones have been suggested as minor contributors to CRAM, and while they typically display little contribution to the ^13^C NMR spectra of marine DOM, they are present in riverine samples.^17,25,28,36,37^ Ethers and esters seemed straightforward inclusions,^38,39^ with their presence in multiple biopolymers known to contribute to DOM (i.e., tannins and lignin), identification of methyl ethers and longer alkyl-ethers in marine samples,^25^ and the virtual indistinguishability of alcohols from ethers and carboxylic acids from carboxylic esters in ^1^H and ^13^C spectra as complex as those of DOM. While common in terrestrial and riverine DOM, phenol and other aromatic functionalities were excluded, as the requirement for several atoms per functionality and low NMR contribution of aromatics in marine DOM limit the number of aromatic molecules than can contribute to most molecular formulas within samples.

Thus, compounds **5**–**16** were synthesized (pages S9–S27) in addition to previously obtained COOH-CRAM analogues **1**–**4**. Diacids **5** and **6** were included to allow for comparisons between O/C ratios and functional group compositions, as well as comparisons between di-, tri-, and tetracarboxylic acid compounds. Lactone **7**, isolated as a transformation product from the attempted SPE extraction of a large-scale preparation of **1** (see additional details in pages S4–S5, S22), provides a cyclic ester with an identical molecular formula to **1**. It should be noted that this compound was isolated using preparative high-performance LC, and it exists as two closely eluting diastereomers. Mono alcohols **8** and **9** are regioisomers of one molecular formula (C_13_H_18_O_7_), providing data for how small positional changes can affect LC and MS2 experimental outcomes. Of note, the tertiary alcohol **9** was prepared directly from the hydrolysis of **7** and as such is a much simpler diastereomeric mixture than secondary alcohol **8**.

Ether **10**, ketone **11**, and α-hydroxy ketone **12** are the sole regioisomers of their respective class, with the positional change in oxygen functionality of ether **10** relative to the other compounds coming as a result of synthetic challenges. It should be noted that α-hydroxy ketone **12** proved relatively unstable in both water and methanol over the course of 24–72 h. This compound was intended to probe how the combination of an alcohol and a ketone affect retention time and fragmentation behavior of a single molecule, and its relative instability should be noted when considering these structures as part of DOM or CRAM. The final set of compounds are diols **13**–**16**. Diol **13** is the only diol containing eight oxygens and was isolated as several single diastereomers from preparative high-performance LC due to challenges with extraction into organic solvents during reaction work-up. Diols **14** are regioisomeric compounds that were inseparable in prior synthetic steps. Diols **15** and **16** were present as a mixture, where some portion of the esters represented in **16** were unhydrolyzable before increasingly harsh conditions led to complete decomposition of the organic material. However, extracted ion chromatograms and LC and MS2 studies allow for easy differentiation of different molecular formulas in the context of this work, and the incorporation of a linear ester functionality (as opposed to cyclic lactone **7**) was seen as a useful comparison.

### Retention Time Investigation

Liquid chromatography (LC) was performed using a 5–100% acetonitrile gradient in H_2_O with 0.1% formic acid over 10 min, allowing assessment of retention time as a measure of polarity. It is important to note that different molecular formulas have significant differences in retention profiles within DOM (see, for example, Figures S23, S25, and S26 and pages S36–37), and critically, the extracted ion chromatogram for a given formula can vary drastically from the total ion chromatogram. For this reason, we present results in the context of the distribution of retention times for the same molecular formula in TRM (Figure 1b, page S28). We use a novel metric, percent cumulative intensity (CI, Figure 1c), which is determined as the percentage of summed intensity that has eluted for the equivalent molecular formula in TRM at the retention time of the compound (Figure 1d,e). CI was also computed for SRFA for comparison (page S28).

To first provide a benchmark for relative elution profiles, we examined the retention times of our COOH-CRAM analogues **1**–**6**. Changing the number of carboxylic acid groups on this scaffold caused only minor difference in absolute retention time, with all isomers of diacid **5**, methyl-diacid **6**, triacid alkene **1**, triacid alkane **2**, tetraacid alkene **3**, and tetraacid alkane **4** eluting between 4.82 and 6.14 min (Figure 1b) (i.e., over less than a 15% change in ACN concentration). These tri- and tetraacids elute late in their corresponding CI, with compounds **1** and **2** eluting between 63% and 87% CI, and compounds **3** and **4** eluting between 76% and 91%. Conversely, diacids **5** and **6** eluted between 45% and 63% CI, indicating that for lower O/C ratios, COOH-CRAM elute near the center of their corresponding molecular formula elution profile in DOM. While O/C ratios have previously been used to indicate or characterize relative hydrophobicity and hydrophilicity in DOM LC studies,^40–42^ this result indicates that O/C ratio alone is a poor indicator of retention time in reverse-phase LC for carboxylic acid rich compounds. Notably, while a more oxygen rich formula in DOM (i.e., Figures S5 and S13, pages S31 and S33) on average elutes earlier than a less oxygen rich formula (i.e., Figures S6 and S15, pages S32 and S34), they still show considerable overlap in their elution profiles. Therefore, the small range in retention of COOH-CRAM analogues **1**–**6**, but relatively large difference in the O/C ratio (0.31–0.57), shows that functionality needs to be extensively considered when examining broad retention times of molecular formulas in DOM. We are confident that the use of standards such as those employed here offers an avenue to determine which functional group compositions elute early or late within LC experiments, for example in conjunction with metrics such as CI.

To explore this idea further, we examined the alcohol, ketone, and diol analogues of CRAM triacid **2**. The inclusion of an alcohol in the structures of **8** and **9** significantly decreased retention time relative to the parent triacid **2**, with the earliest isomers eluting very early at 1% CI for secondary alcohol **8**, the latest eluting by 55% for tertiary alcohol **9**, the average at 15% for **8**, and 26% for **9**. Similarly, the inclusion of a ketone in compound **11** decreased retention times and CI relative to triacid **2** (first isomer = 31% CI, last = 54%, average = 45%). Finally, the incorporation of a 1,2-diol functionality in compound **13** led to extremely early eluting compounds, with all isomers eluting in the first 1% of CI. These isomers of **13** provide a close O/C comparison to tetraacids **3** and **4**, with all three compounds containing eight oxygen atoms, and diols and acids bearing 13 and 14 carbon atoms respectively (O/C ratios; **13** = 0.62, **3**/**4** = 0.57).

To reinforce that functional group composition was a stronger indicator of retention than the O/C ratio, two additional diols, **14** and **15**, were prepared. These compounds contained six oxygen atoms and either 12 or 13 carbon atoms, allowing for more direct comparison to triacids **1** and **2** (C_13_O_6_H_16_ and C_13_O_6_H_18_, respectively). The isomers of **14** and **15** also had very low CI values, with 11 out of 14 isomers eluting within 5% CI, and the latest eluting at 12% for **14** and 22% for **15**. The lower O/C ratios of these compounds compared to diols **13** (**14** = 0.5, **15** = 0.46, **13** = 0.62), but only slightly increased average CIs (**14** = 4%, **15** = 6%, **13** = 1%), reinforce that functional group composition is a far stronger indicator of retention time in LC than the O/C ratio. Finally, α-hydroxy ketone **12** was prepared to examine a molecule containing both a ketone and an alcohol. This compound again eluted early relative to its tetraacid counterparts (isomer 1 = 3% CI, isomer 2 = 34%, average = 19%).

Ultimately, these alcohol and ketone bearing compounds exemplify the relatively strong effect of functional group composition versus molecular formula and O/C ratio. However, even a single alcohol functionality significantly increases individual molecule LC polarity, indicating that switching a carboxylic acid functionality for two alcohol groups and an additional unsaturation is not a viable way to explain the polarity behavior of the majority of observed isomers in DOM mixtures. While ketones appear initially as strong candidates for mid-eluting isomers, their ^13^C NMR derived contributions to terrestrial DOM are low (as low as 1 in 38 C atoms),^28^ and in marine DOM are very rare (as low as 1 in 110 C atoms).^17,25^ As such, they can explain some portion of observed isomers, but additional functionality must be required to describe the retention profile of any specific molecular formula within DOM, especially when considering long-lived CRAM molecules.

Ethers and esters were subsequently examined as the last prominent oxygen functionalities consistent with the NMR and MS data of marine DOM. Ether **10** showed a broad diversity in polarity between isomers, with CI’s between 24% and 84%, and an average of 55%. This was particularly notable to us, as it allows for a range of retention times earlier than those of the COOH-CRAMs **1**–**6**, while not displaying the same propensity for the drastic retention time reduction seen for alcohols **8**–**9** and **12**–**16**. Furthermore, regions that can indicate ether functionalities are highly prevalent in the NMR spectra of terrestrial DOM and RDOM, being observed at ^1^H NMR chemical shifts between 2.9 and 4.1 ppm, and ^13^C NMR chemical shifts between 47 and 90 ppm, with these regions contain predominantly alcohols, ethers, and esters. Furthermore, methyl ethers have been shown to be prevalent in some marine DOM samples.^25,35^ Conversely, less variety in retention time was observed for lactone **7** in comparison to those for ether **10**. However, only two isomers of **7** were isolated (vs nine for **10**), which limits definitive conclusions.

The comparatively shorter retention times of **7** were somewhat surprising, as both triacid alkene **1** and lactone **7** share the same molecular formula (C_13_O_6_H_16_), but all observed isomers of **7** are more polar than all isomers of **1**. Our initial expectation was that the additional acid of alkene **1** would provide another site for ionization and stronger H-bonding in solution, and thus lower retention time. However, it appears that at least for the decalin scaffolds examined here, that the incorporation of a cyclic ester can lead to decreased retention time relative to its corresponding acid equivalent. This may be due to a conformational difference, as the extra cyclization in the lactone moves the hydrophobic regions toward the center of the molecule, and positions the oxygen atoms toward the outside. One additional compound bearing an ester did exist within our data set; however, it was observed as an impurity from the hydrolysis to form diol **15**. Ester **16**, as such, contains an acid, two alcohols, and an ethyl ester, with isomers eluting at 5.52 and 5.69 min. It is notable that the absolute retention times of the diastereomers of **16** are significantly higher than those for the other diols examined, indicating that carboxylic acid–alcohol intramolecular interactions may play a key role in the very low LC retention times of these other diols. However, they still follow the same relative CI trend of all diols, with isomers of **16** eluting at 20% and 24% compared to the equivalent CI of TRM.

### MS2 Investigation

Fragmentation studies were performed using higher energy collisional dissociation (HCD) at 35 V (low energy) and 75 V (high energy). Additional fragmentation metrics are provided (pages S29–S30) to reinforce the qualitative trends discussed here through the observation of various diagnostic fragment peaks. For COOH-CRAM analogues **1**–**4**, we previously showed that low-energy fragmentation led predominantly to neutral losses of H_2_O, CO_2_, and minor losses of CO (‘functional group’ fragments), as well as trace ‘fingerprint’ fragments from decomposition of the carbon backbone.^30^ High-energy fragmentation provided high intensities of functional group fragments, as well as slightly increased fingerprint fragmentation for alkanes **2** and **4**, and moderate fingerprint fragmentation of alkenes **1** and **3**. Added to this initial set of acid-only compounds, diacids **5** and **6** show comparatively decreased fragmentation, with parent ions being the dominant peaks at both low and high energy (Figure 2a; shown for **5**). This is in stark contrast with our previously disclosed CRAM-analogues, none of which retained their parent ions under high-energy fragmentation, and likely suggests that intramolecular proximity of functionalities (i.e., like in **2** and **4**, Figure 2b) promotes the sequential loss of H_2_O and CO_2_ typically associated with the fragmentation of DOM.^34,43,44^

**Figure 2:**
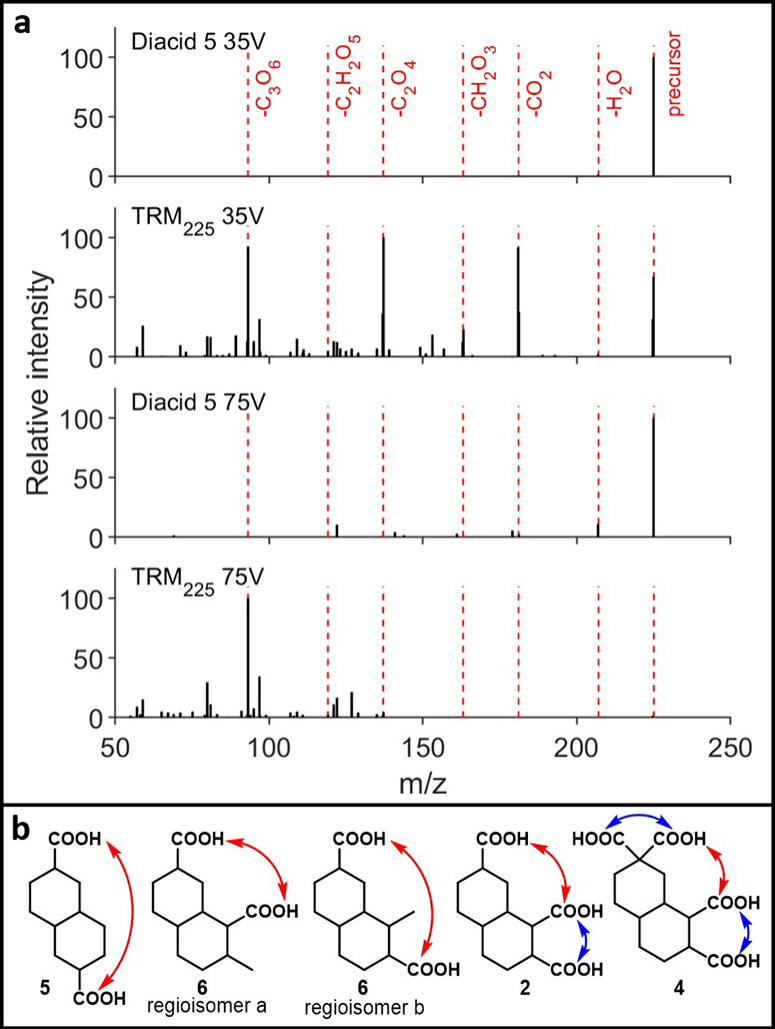
(a) HCD fragmentation data of **5** at 35 and 75 V vs same mass in TRM (35 and 75 V). (b) Relative proximity of acids **5** vs **6** vs **2** vs **4** (red = far, blue = near).

Comparison of acids **5** and **6** with TRM highlights similar trends to the previously examined acids **1**-**4**.^30^ In DOM, an increased number of neutral H_2_O and CO_2_ losses are observed during the low-energy fragmentation of the same masses, alongside moderate fingerprint fragmentation (Figure 2a). When fragmented at high energy, DOM shows very low or no intensity of functional group fragments and instead undergoes extensive fragmentation to fingerprint ions. As we previously highlighted,^30^ it is important to note that utilizing an orbitrap for these types of fragmentations leads to the isolation of ions across a single unit mass, and the diversity of fragments seen here arises from the fact that several different parent ions and isomers are being fragmented. Nevertheless, the stability of the parent ion and functional group fragments for diacids **5** and **6** reinforces that COOH-CRAM does not fragment in the same way as DOM.

For triacid alcohols **8** and **9**, low-energy fragmentation showed the sequential neutral losses of two CO_2_ and one H_2_O (Figure 3). Similarly, at low energy, diacid diols **14** and **15** showed sequential losses of up to two CO_2_ and up to two H_2_O (pages S50–S51), while triacid diols **13** showed losses of up to three CO_2_ and up to two H_2_O (Figure 4). Pleasingly, fragmentation at high energy for alcohol **8** and diols **13**–**15** resulted in extensive fingerprint fragmentation (**8** and **9** in Figure 3, **13** in Figure 4; **14**, **15** in pages S50–S51). However, it is important to note that for all of these compounds several functional group fragments remained, with prominent ions observed for **8**, **9**, and **13**–**15** corresponding to decalin backbone fragments (ketene anion **i**/**ii** in Figure 3b; oxy-anion **iii**/**iv** in Figure 4b). For tertiary alcohol **9**, fragmentation was less extensive (Figure 3a). While this could be due to the relative instability of a formal or partial negative charge at a tertiary center relative to a secondary center, investigation of the well-resolved individual isomers of alcohol **8** (see Figure S44, page S53) showed significantly fewer fragments compared with the total HCD spectrum. As such, the relative simplicity of the high-energy fragmentation of tertiary alcohol **9** is most likely due to its existence as relatively few isomers compared to secondary alcohol **8**.

**Figure 3:**
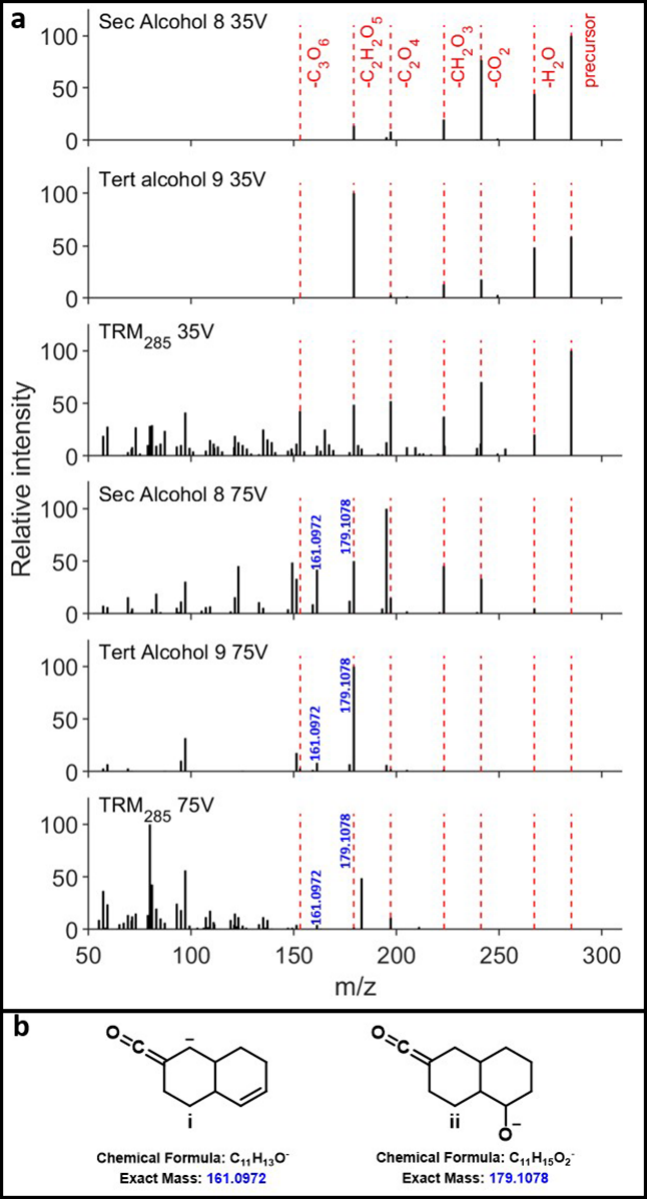
(a) Top: 35 V HCD fragmentations of secondary alcohol **8**, tertiary alcohol **9**, and TRM285. Bottom: 75 V HCD fragmentations of secondary alcohol **8**, tertiary alcohol **9**, and TRM285. (b) Idealized functional group fragments (**i**/**ii**) for alcohols **8** and **9**.

**Figure 4:**
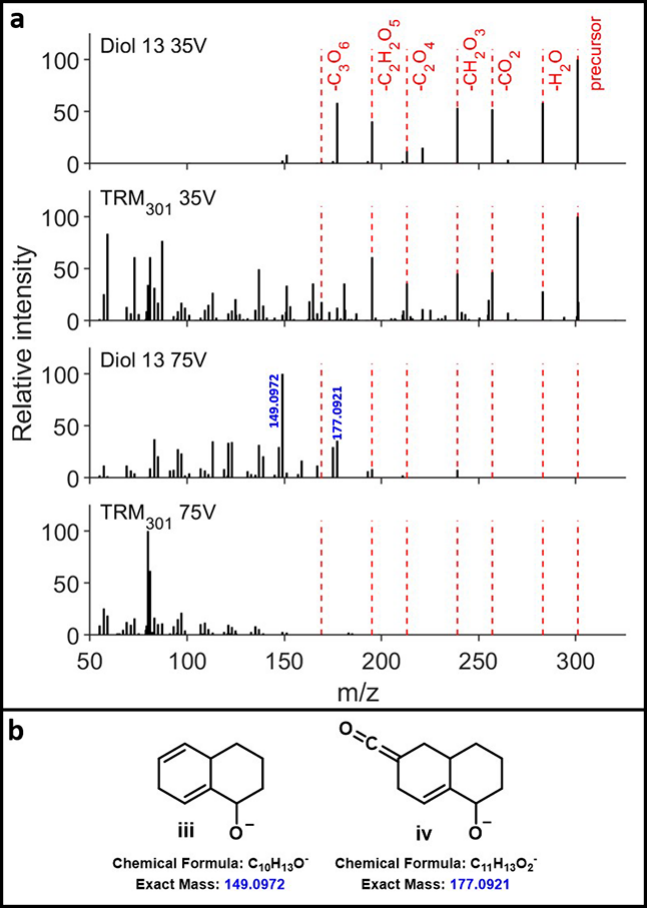
(a) HCD fragmentation of diol **13** and TRM301. (b) Idealized functional group fragments (**iii**/**iv**) for diol **13**.

Turning to a comparison of the alcohols and diols to DOM at *m*/*z* 285, similar patterns emerge as for the COOH-CRAM analogues. At low energy, DOM exhibits the same functional group fragments, while undergoing significantly greater fingerprint fragmentation across all masses corresponding to alcohols **8**–**9** and diols **13**–**15** (pages S42–S43, S48–S51). This is true even for triacid diol **13**, which, alongside ketone **11**, α-hydroxy ketone **12**, and diol **16**, underwent the most extensive fragmentation of all our synthetic compounds (page S29). At higher energy, functional group fragments are again almost entirely lost for all alcohol and diol masses in TRM (*m*/*z* 285 and 301). This suggests that while the incorporation of these additional oxygen functionalities may promote the increased fragmentation seen in high-energy HCD experiments on natural samples, it does not enable the breakdown extent seen in DOM. This is highlighted by the presence of decalin-type fragments of 161.0972 (**i**) and 179.1078 (**ii**) for **8** and **9** that are at very low intensity in DOM (Figure 3a). As the LC data suggested that alcohols are likely present only as relatively early eluting isomers of any specific formula (*vide supra*), it was also examined whether fragmentation data corresponding to later LC retention led to greater conservation of higher mass fragments. However, investigation of three separate areas within DOM for the mass corresponding to diol **13** showed essentially no variation in average fragment mass, suggesting that other structural features must contribute to this extensive DOM fragmentation (page S55).

For lactone **7**, ketone **11**, and α-hydroxy ketone **12**, analogous trends were observed in the low and high-energy HCD spectra. Low-energy spectra show functional group fragments that correspond predominantly to water, CO_2_, and CO losses, which are also seen in the low-energy fragmentation spectra of TRM (along with mild fingerprint fragmentation). At high energy, smaller functional group fragments are observed for **7**, **11**, and **12**, while DOM undergoes extensive breakdown to fingerprint fragments. It should be noted that ketone **11** emerged as one of the best matches to DOM based on fragmentation metrics (page S29). It demonstrated one of the most comparable extents of fragmentation to TRM, and it exhibited the highest number of fragmentation peaks observed in the same experiment. This adds to the relative consistency of the retention profile of **9** to DOM (*vide supra*). However, it should be stressed that the relatively high intensity of MS2 peaks corresponding to functional group fragments of **11** (see Figures S36 and S37), and relative lack of them in the same nominal mass in TRM (pages S45–S46), limits how much decalin ketones such as **11** are likely to contribute to the fragmentation data of marine mixtures.

For ester acid diol **16**, the majority of functional group fragments seen in both low- and high-energy HCD fragments are absent in the fragmentation spectra of DOM (35 V HCD shown in Figure 5a). However, it should be noted that for the fragmentation of **16**, a neutral loss of C_2_H_4_ (ethene) corresponding to fragmentation of the ester is observed immediately from the parent ion during low-energy HCD fragmentation, and from the first H_2_O loss fragment (299.1508 to 271.1189, 281.1379 to 253.1081, Figure 5a, idealized fragmentation in Figure 5b). Subsequent fragments are more typical, corresponding to losses of CO_2_, H_2_O, and CO. Enticingly, ether **10** also shows neutral losses of CH_3_OH (255.1239 to 223.0978 in 35 V HCD shown in Figure 5a, idealized fragmentation in Figure 5b) during low-energy HCD fragmentation, which are also observable in TRM, with subsequent losses to 205.0872 (−H_2_O) and 161.0974 (−H_2_O, CO_2_) being reliant on the initial loss of CO_2_ and CH_3_OH.

**Figure 5:**
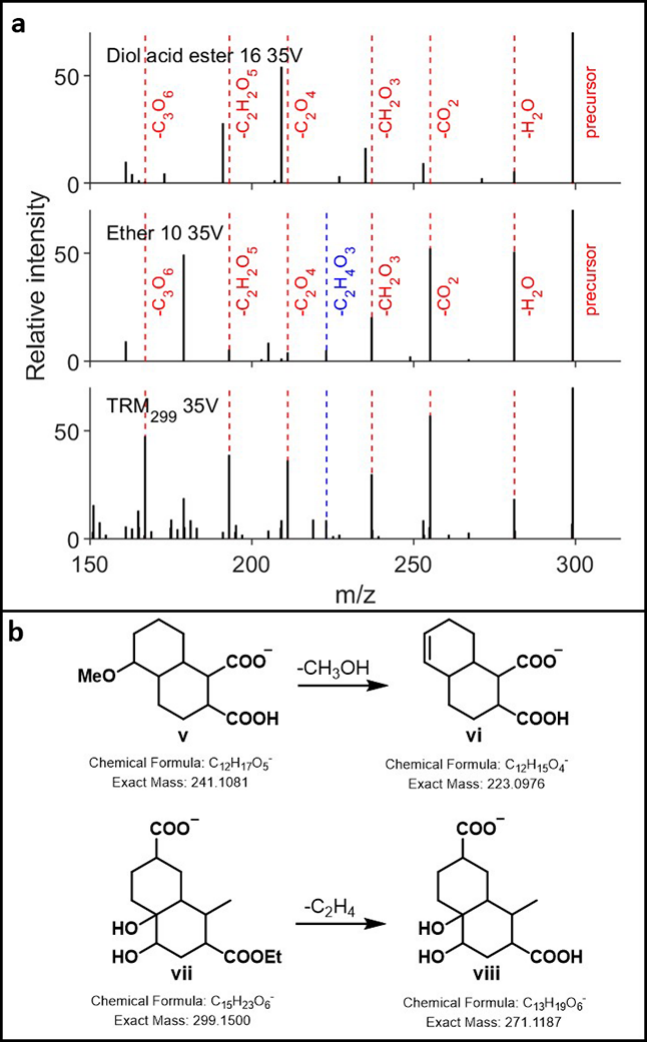
(a) 35 V HCD spectra of diol acid ester (top), ether (mid), and correpsonding DOM mass 299 (bottom); blue dashed line indicates CO_2_ and CH_3_OH loss. (b) Idealized CH_3_OH losses of ether **10** (**v** to **vi**), ethene losses of ester **16** (**vii** to **viii**).

The presence of these methyl ethers in TRM is not unexpected, with this functionality existing as a clear derivative of lignin-like structures, and the presence of ethers being documented at several ocean depths from both 1D and 2D NMR techniques.^25^ However, we believe that the neutral loss of CH_3_OH for ether **10**, as well as the neutral loss of ethene for ester **16**, is an important indicator of why the fragmentation spectra of our synthetic molecules and DOM differ so markedly, especially at higher energies. The extensive fingerprint fragmentation of DOM in HCD experiments suggests that molecules ionized and fragmented from these DOM samples must have backbones that break into significantly smaller fragments than the fused alicyclic structures presented here. Here, the incorporation of esters and ethers into core carbon backbones would remedy this lack of fingerprint fragmentation for fused alicyclic structures. Their presence could enable ring-opening type fragmentations (Figure 6a), or the possibility for small fragments linked through ethers or esters to cleave from larger structures (Figure 6b), allowing for the generation of diverse and plentiful fingerprint fragments observed in natural DOM samples. To exemplify this, masses corresponding to small C_7_H_*x*_O_*y*_ fragments (**ix**, **x**, **xi**, Figure 6c, ketenes are −H_2_O loss products of carboxylic acids) that were seen in all HCD75 spectra gathered in TRM regardless of parent ion mass (exemplified in Figure 6d, visible in pages S39–S52 for all masses) have been imagined as idealized breakdown products of these pathways.

**Figure 6:**
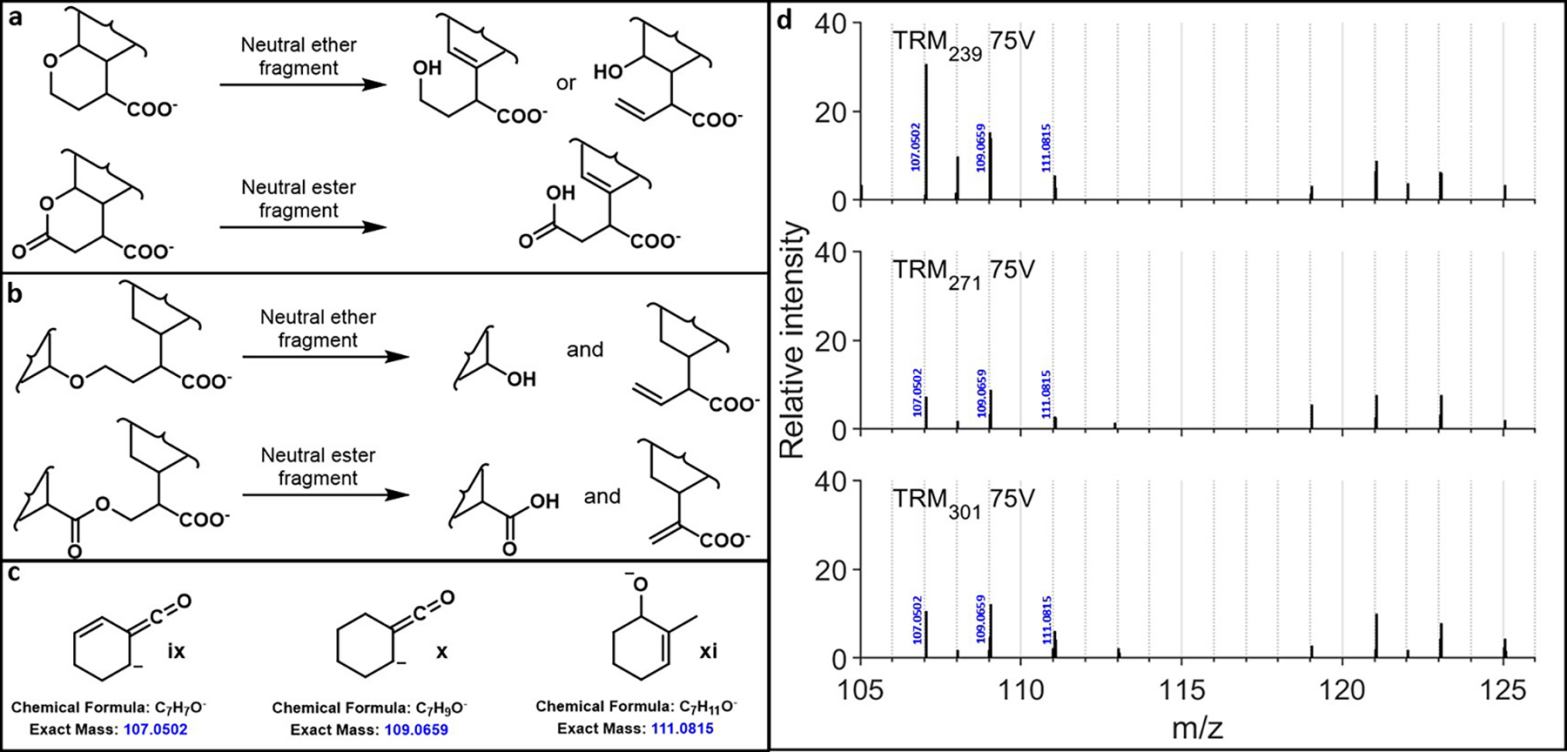
(a) Hypothetical DOM cyclic ester and ether fragmentations; (b) hypothetical DOM linear ether and ester fragmentations; (c) idealized DOM fragments from masses observed in all TRM HCD fragmentation spectra gathered in this work; (d) zoomed-in HCD75 fragmentation spectra showing masses 107.0502, 109.0659, and 111.0815, with repeating mass set from 119 to 125 *m*/*z* also shown.

## Conclusions

Twelve CRAM analogues with varying oxygen functional groups and O/C ratios were prepared, analyzed by LCMS and MS2, and compared with representative freshwater and marine DOM reference materials (SRFA/TRM0522, respectively). Notably, we have expanded the range of O/C and H/C ratios accessible for these types of compounds beyond those prepared in our previous work.^30^ LC analysis found that the addition of alcohol groups significantly increased the polarity of CRAM-like compounds. As a result, in natural samples alcohol-containing compounds are likely to appear as early eluting isomers for a given molecular formula. This effect was especially strong for 1,2-diol compounds, showing that replacing an acid group with two alcohol groups and adding a C–C double bond is unlikely to explain the full diversity of molecular isomers found in DOM for a given formula. Instead, functional groups like ketones, ethers, and esters provided better matches across the full range of relative retention times observed in natural DOM mixtures, with ketones likely being less significant in a marine context. Additionally, we have developed a new metric, cumulative intensity, that is useful for the comparison of retention times of isolated compounds to DOM, and we believe it should be useful for documenting and comparing retention times between individual DOM molecular formulas and between natural samples.

MS2 data revealed similar functional group fragmentation patterns between the newly synthesized compounds and representative DOM molecular formulas during low-energy HCD experiments. Critical to note is that oxygen functional group diversity and relative position were the key mediators of the MS fragmentation of these small-molecule CRAM analogues. Data from comparisons between di-, tri-, and tetracarboxylic acids suggest that for reliable functional group fragmentation, proximity between functional groups is essential. Notably, at low energies CH_3_OH and C_2_H_4_ losses were observed from an ether and an ester functionalized CRAM, respectively. Similar CH_3_OH fragment losses from the ether were also observed during the fragmentation of the same nominal mass in TRM-0522. High-energy HCD experiments caused extensive fragmentation of DOM into low *m*/*z* masses, indicative of carbon backbone breakdown. In contrast, the synthetic compounds retained fragments of some functional groups even at higher energies, and the extent of backbone fragmentation varied greatly among the synthesized compounds. Among the synthesized compounds, diols and an α-hydroxy ketone broke down into the smallest average fragment masses, most closely resembling DOM fragmentation. However, these compounds still did not fragment to the same extent as DOM under either low- or high-energy HCD conditions.

Critically, this work suggests that the commonly used trap-based collision induced dissociation experiments, especially in negative mode, are not suitable for examining carbon backbone differences between molecules in DOM and should be carefully scrutinized when performed as the only fragmentation technique.^1–3,31^ Using them in this context has the very real possibility to lead to misinformed conclusions based on the most labile functional groups that are near-ubiquitous in DOM, while ignoring the carbon backbone differences that are crucial in defining molecular origin and subsequently DOMs fluxes. As a consequence, we strongly recommend that future work includes the use of techniques like HCD, which can further break down generated fragments within one experiment.

In a wider context, this work shows that the alicyclic structures proposed as potential CRAM candidates in prior decades^17^ can no longer be considered as viable to explain any meaningful portion of the molecules present within RDOM. The incorporation of oxygen atoms to the backbones of DOM molecules is almost certainly fundamental in enabling the extent of fragmentation observed in corresponding high-energy HCD experiments. We propose that ethers and esters are excellent candidates to explain the difference in fragmentation data between the synthesized compounds and RDOM, while also having comparable LC properties. A single one of either of these functionalities incorporated into the carbon backbones of CRAM-like molecules would enable more extensive fragmentation than for all-carbon alicyclic systems at relatively low total abundance within any given DOM sample.

Recent work has highlighted the prevalence of quaternary oxygenated carbons within DOM as potential contributors to CRAM in the environment and theorized about a potential oxidative dearomatization–spirocyclization reaction cascade for their formation from phenols.^28^ In light of this, it should be noted that spirocyclic lactone **7** fragmented relatively extensively, but not as extensively as our nonspirocyclic quaternary oxygenated diols or alcohol. Importantly, our study was not designed to test for diversity in these types of spirocycles directly and, as a result, can only draw relatively limited conclusions. However, it does highlight that within the confines of MS2 fragmentation, both nonspirocyclic (i.e., alcohol) and spirocyclic (i.e., ester) oxygenated quaternary centers are excellent candidates for DOM-like chemical functionality and are a critical avenue for future synthetic design.

Outside of these oxidative dearomatization-type pathways, the degradation of lignin by reactive oxygen species^45^ and photodegradation of terpenoids/carotenoids^18,46^ are two viable and commonly invoked mechanisms for the environmental production of polycarboxylic acid molecules with backbone ethers or esters. However, it is important to note that the use of single molecules in the work we have performed here cannot alone confirm these pathways and is better suited to supporting or challenging the prevalence of structural motifs within DOM. Another key advantage of this work is its ability to provide accurate fragmentation data for library matching,^16^ as carboxylic acid rich molecules that match CRAM or other commonly invoked RDOM models remain exceptionally rare in the synthetic or isolative literature. These compounds also represent opportunities to develop the stability^47^ and response factor^48^ relationships we have begun to explore with our initial synthetic CRAM molecules. Future synthetic investigations will focus on the preparation of compounds suitable to test whether fused cyclic ethers and esters, as well as ether or ester linked carbocyclic systems, more accurately align with the HCD fragmentation data of DOM. Additional work will also seek to expand the range of these compounds to test whether other spirocyclic scaffolds behave like RDOM during LC elution and under MS2 fragmentation.

## Supplementary Material



## Notes

Description of the synthetic methods, expanded general methods, synthetic procedures, tabulated metrics for fragmentation and cumulative intensity data, extracted ion chromatograms for TRM-0522 and SRFA, tandem mass spectrometry data for final compounds, and NMR and LC-MS-CAD data for all synthetic intermediates and final compounds (PDF)

## References

[1] Zark M., Dittmar T. (2018). Universal Molecular
Structures in Natural Dissolved
Organic Matter. Nat. Commun..

[2] Witt M., Fuchser J., Koch B. P. (2009). Fragmentation
Studies of Fulvic Acids
Using Collision Induced Dissociation Fourier Transform Ion Cyclotron
Resonance Mass Spectrometry. Anal. Chem..

[3] Hawkes J. A., Patriarca C., Sjöberg P. J., Tranvik L. J., Bergquist J. (2018). Extreme Isomeric
Complexity of Dissolved Organic Matter Found across Aquatic Environments. Limnol. Oceanogr. Lett..

[4] Patriarca C., Bergquist J., Sjöberg P. J., Tranvik L., Hawkes J. A. (2018). Online
HPLC-ESI-HRMS Method for the Analysis and Comparison of Different
Dissolved Organic Matter Samples. Environ. Sci.
Technol..

[5] Han L., Kaesler J., Peng C., Reemtsma T., Lechtenfeld O. J. (2021). Online
Counter Gradient LC-FT-ICR-MS Enables Detection of Highly Polar Natural
Organic Matter Fractions. Anal. Chem..

[6] Leyva D., Jaffe R., Fernandez-Lima F. (2020). Structural Characterization of Dissolved
Organic Matter at the Chemical Formula Level Using TIMS-FT-ICR MS/MS. Anal. Chem..

[7] Leyva D., Tose L. V., Porter J., Wolff J., Jaffé R., Fernandez-Lima F. (2019). Understanding
the Structural Complexity of Dissolved
Organic Matter: Isomeric Diversity. Faraday
Discuss..

[8] Hertkorn N., Frommberger M., Witt M., Koch B. P., Schmitt-Kopplin P., Perdue E. M. (2008). Natural Organic Matter and the Event Horizon of Mass
Spectrometry. Anal. Chem..

[9] Nebbioso A., Piccolo A. (2013). Molecular Characterization
of Dissolved Organic Matter
(DOM): A Critical Review. Anal. Bioanal. Chem..

[10] Hansell D. A. (2013). Recalcitrant
Dissolved Organic Carbon Fractions. Annu. Rev.
Mar. Sci..

[11] Moran M. A., Ferrer González F., Fu H., Nowinski B., Olofsson M., Powers M., Schreier J., Schroer W., Smith C., Uchimiya M. (2022). The Ocean’s
Labile DOC Supply
Chain. Limnol. Oceanogr..

[12] Yamashita Y., Tanoue E. (2004). Chemical Characteristics of Amino Acid-Containing Dissolved
Organic Matter in Seawater. Org. Geochem..

[13] Amon R. M., Benner R. (2003). Combined Neutral Sugars as Indicators of the Diagenetic
State of Dissolved Organic Matter in the Arctic Ocean. Deep-Sea Res. I: Oceanogr. Res. Pap..

[14] Aristilde L., Guzman J. F., Klein A. R., Balkind R. J. (2017). Compound-Specific
Short-Chain Carboxylic Acids Identified in a Peat Dissolved Organic
Matter Using High-Resolution Liquid Chromatography–Mass Spectrometry. Org. Geochem..

[15] Xiao M., Wu F., Liao H., Li W., Lee X., Huang R. (2010). Characteristics
and Distribution of Low Molecular Weight Organic Acids in the Sediment
Porewaters in Bosten Lake, China. J. Environ.
Sci..

[16] Papadopoulos
Lambidis S., Schramm T., Steuer-Lodd K., Farrell S., Stincone P., Schmid R., Koester I., Torres R., Dittmar T., Aluwihare L., Simon C., Petras D. (2024). Two-Dimensional Liquid Chromatography
Tandem Mass Spectrometry Untangles the Deep Metabolome of Marine Dissolved
Organic Matter. Environ. Sci. Technol..

[17] Hertkorn N., Benner R., Frommberger M., Schmitt-Kopplin P., Witt M., Kaiser K., Kettrup A., Hedges J. I. (2006). Characterization
of a Major Refractory Component of Marine Dissolved Organic Matter. Geochim. Cosmochim. Acta.

[18] Arakawa N., Aluwihare L., Simpson A., Soong R., Stephens B., Lane-Coplen D. (2017). Carotenoids
Are the Likely Precursor of a Significant
Fraction of Marine Dissolved Organic Matter. Sci. Adv..

[19] Aluwihare L., Repeta D. (1999). A Comparison of the Chemical Characteristics of Oceanic
DOM and Extracellular DOM Produced by Marine Algae. Mar. Ecol. Prog..

[20] Aluwihare L. I., Repeta D. J., Pantoja S., Johnson C. G. (2005). Two Chemically Distinct
Pools of Organic Nitrogen Accumulate in the Ocean. Science.

[21] Dittmar T., Koch B. P. (2006). Thermogenic Organic
Matter Dissolved in the Abyssal
Ocean. Mar. Chem..

[22] Mitschke N., Vemulapalli S., Dittmar T. (2023). NMR Spectroscopy of Dissolved Organic
Matter: A Review. Environ. Chem. Lett..

[23] Mopper K., Stubbins A., Ritchie J. D., Bialk H. M., Hatcher P. G. (2007). Advanced
Instrumental Approaches for Characterization of Marine Dissolved Organic
Matter: Extraction Techniques, Mass Spectrometry, and Nuclear Magnetic
Resonance Spectroscopy. Chem. Rev..

[24] Kim S., Kim D., Jung M., Kim S. (2022). Analysis of Environmental Organic
Matters by Ultrahigh-Resolution Mass Spectrometry—A Review
on the Development of Analytical Methods. Mass
Spectrom. Rev..

[25] Hertkorn N., Harir M., Koch B. P., Michalke B., Schmitt-Kopplin P. (2013). High-Field
NMR Spectroscopy and FTICR Mass Spectrometry: Powerful Discovery Tools
for the Molecular Level Characterization of Marine Dissolved Organic
Matter. Biogeosciences.

[26] Capley E. N., Tipton J. D., Marshall A. G., Stenson A. C. (2010). Chromatographic
Reduction of Isobaric and Isomeric Complexity of Fulvic Acids to Enable
Multistage Tandem Mass Spectral Characterization. Anal. Chem..

[27] DiDonato N., Hatcher P. G. (2017). Alicyclic Carboxylic Acids in Soil Humic Acid as Detected
with Ultrahigh Resolution Mass Spectrometry and Multi-Dimensional
NMR. Org. Geochem..

[28] Li S., Harir M., Bastviken D., Schmitt-Kopplin P., Gonsior M., Enrich-Prast A., Valle J., Hertkorn N. (2024). Dearomatization
Drives Complexity Generation in Freshwater Organic Matter. Nature.

[29] Lam B., Baer A., Alaee M., Lefebvre B., Moser A., Williams A., Simpson A. (2007). Major Structural
Components in Freshwater
Dissolved Organic Matter. Environ. Sci. Technol..

[30] Craig A. J., Moodie L. W., Hawkes J. A. (2024). Preparation
of Simple Bicyclic Carboxylate-Rich
Alicyclic Molecules for the Investigation of Dissolved Organic Matter. Environ. Sci. Technol..

[31] Osterholz H., Niggemann J., Giebel H.-A., Simon M., Dittmar T. (2015). Inefficient
Microbial Production of Refractory Dissolved Organic Matter in the
Ocean. Nat. Commun..

[32] Cheng C.-R., Yang M., Wu Z.-Y., Wang Y., Zeng F., Wu W.-Y., Guan S.-H. (2011). Fragmentation
Pathways of Oxygenated
Tetracyclic Triterpenoids and Their Application in the Qualitative
Analysis of Ganoderma Lucidum by Multistage Tandem Mass Spectrometry. Rapid. Commun. Mass. Spectrom..

[33] Zhang J., Feng E., Li W., Sheng H., Milton J. R., Easterling L. F., Nash J. J., Kenttämaa H. I. (2020). Studies
of the Fragmentation Mechanisms of Deprotonated Lignin Model Compounds
in Tandem Mass Spectrometry. Anal. Chem..

[34] Demarque D. P., Crotti A. E., Vessecchi R., Lopes J. L., Lopes N. P. (2016). Fragmentation
Reactions Using Electrospray Ionization Mass Spectrometry: An Important
Tool for the Structural Elucidation and Characterization of Synthetic
and Natural Products. Nat. Prod. Rep..

[35] Felgate S.
L., Craig A. J., Moodie L. W., Hawkes J. (2023). Characterization of
a Newly Available Coastal Marine Dissolved Organic Matter Reference
Material (TRM-0522). Anal. Chem..

[36] Abdulla H. A., Minor E. C., Dias R. F., Hatcher P. G. (2010). Changes in the Compound
Classes of Dissolved Organic Matter along an Estuarine Transect: A
Study Using FTIR and 13C NMR. Geochim. Cosmochim.
Acta.

[37] Mitschke N., Vemulapalli S. P. B., Dittmar T. (2024). Dissolved Organic Matter
Contains
Ketones Across a Wide Range of Molecular Formulas. Environ. Sci. Technol..

[38] Leenheer J. A., Wershaw R. L., Reddy M. M. (1995). Strong-Acid,
Carboxyl-Group Structures
in Fulvic Acid from the Suwannee River, Georgia. 2. Major Structures. Environ. Sci. Technol..

[39] Leenheer J. A., Wershaw R. L., Reddy M. M. (1995). Strong-Acid,
Carboxyl-Group Structures
in Fulvic Acid from the Suwannee River, Georgia. 1. Minor Structures. Environ. Sci. Technol..

[40] Stenson A. C. (2008). Reversed-Phase
Chromatography Fractionation Tailored to Mass Spectral Characterization
of Humic Substances. Environ. Sci. Technol..

[41] Sleighter R. L., Caricasole P., Richards K. M., Hanson T., Hatcher P. G. (2015). Characterization
of Terrestrial Dissolved Organic Matter Fractionated by pH and Polarity
and Their Biological Effects on Plant Growth. Chem. Biol. Techn. Agric..

[42] Li Y., Harir M., Uhl J., Kanawati B., Lucio M., Smirnov K. S., Koch B. P., Schmitt-Kopplin P., Hertkorn N. (2017). How Representative Are Dissolved Organic Matter (DOM)
Extracts? A Comprehensive Study of Sorbent Selectivity for DOM Isolation. Wat. Res..

[43] Kanawati B., Schmitt-Kopplin P. (2010). Exploring Rearrangements along the
Fragmentation of
Glutaric Acid Negative Ion: A Combined Experimental and Theoretical
Study. Rapid Commun. Mass. Spectrom..

[44] Grossert J.
S., Fancy P. D., White R. L. (2005). Fragmentation Pathways of Negative
Ions Produced by Electrospray Ionization of Acyclic Dicarboxylic Acids
and Derivatives. Can. J. Chem..

[45] Waggoner D.
C., Wozniak A. S., Cory R. M., Hatcher P. G. (2017). The Role of Reactive
Oxygen Species in the Degradation of Lignin Derived Dissolved Organic
Matter. Geochim. Cosmochim. Acta.

[46] Semitsoglou-Tsiapou S., Meador T. B., Peng B., Aluwihare L. (2022). Photochemical
(UV–Vis/H2O2) Degradation of Carotenoids: Kinetics and Molecular
End Products. Chemosphere.

[47] Craig A. J., Norouzi M., Löffler P., Lai F. Y., Mtibaà R., Breyer E., Baltar F., Moodie L. W. K., Hawkes J. A. (2025). Investigating
the Stability of Individual Carboxylate Rich Alicyclic Molecules Under
Simulated Environmental Irradiation and Microbial Incubation Conditions. Environ. Sci. Technol..

[48] Craig A. J., Ganiyu M. A., Moodie L. W. K., Tshepelevitsch S., Herodes K., Simon H., Dittmar T., Hawkes J. A. (2025). Improvement
of Electrospray Ionization Response Linearity and Quantification in
Dissolved Organic Matter Using Synthetic Deuterated Internal Standards. Anal. Chem..

