# Supplementary material for: Enhanced Structural Understanding of Dissolved Organic Matter through Comparative LC/MS2 Analysis with Synthetic Carboxylate Rich Alicyclic Molecules
Source: Anal Chem. 2025 Aug 21;97(34):18612–20. doi: 10.1021/acs.analchem.5c02665 (PMC12409697; doi:10.1021/acs.analchem.5c02665)
Supplement: Supplementary file 1 [file ac5c02665_si_001.pdf]

Supporting Information

Enhanced Structural Understanding of Dissolved Organic Matter through Comparative LC/MS2 Analysis with Synthetic Carboxylate Rich Alicyclic Molecules

Jeffrey A. Hawkes,[1] Agnes D. Flygare,[1] Lindon W. K. Moodie,[2] and Alexander J. Craig[1,2]*

[1.] Department of Chemistry BMC, Uppsala University, Uppsala 752 37, Sweden

[2.] Department of Medicinal Chemistry, Uppsala University, Uppsala 752 37, Sweden

*Corresponding author: alexander.craig@uu.se

Summary: 158 pages, 147 figures, 13 tables, 9 schemes

Contents:

Abbreviations List **S2**
Synthetic Description **S3-8**
General Methods **S9-10**
Synthetic Procedures for the preparation of intermediates **18-46** **S11-21**
Synthetic Procedures for the preparation of CRAM analogues **5-16** **S22-27**
Tabulated % Cumulative Intensity Data for **5-16** **S28**
Tabulated Fragmentation Metrics for **5-16** **S29-30**
Extracted Ion Chromatograms used for % Cumulative Intensity Calculations **S31-38**
Tandem Mass Spectrometry Data for **5-16** and TRM-0522 **S39-55**
NMR Data for Intermediates **18-46** **S56-74**
NMR and LC-MS-CAD data for CRAM analogues **5-16** **S75-157**
Bibliography **S158**

Abbreviations List

Bpin	Boron pinacolate
br s	Broad Singlet
CAD	Charged aerosol detection
COSY	Correlation spectroscopy
CRAM	Carboxylate rich alicyclic molecule
d	Doublet
dd	Doublet of doublets
ddd	Doublet of doublet of doublets
dddd	Doublet of doublet of doublet of doublets
ESI	Electrospray ionization
Et_2O	Diethyl ether
EtOAc	Ethyl acetate
HCD	Higher energy collisional dissociation
HPLC	High-performance liquid chromatography
HMBC	Heteronuclear multiple bond correlation
HSQC	Heteronuclear single quantum correlation
HRMS	High-resolution mass spectrometry
LCMS	Liquid chromatography mass spectrometry
m	Multiplet
mCPBA	Meta chloroperbenzoic acid
MeOD	Deuterated methanol
NaHMDS	Sodium hexamethyldisilazane
NMR	Nuclear magnetic resonance
PET	Petroleum ether
s	Singlet
SDVP	Styrene-divinylbenzyl
SPE	Solid-phase extraction
THF	Tetrahydrofuran
TIC	Total ion chromatogram
TLC	Thin-layer chromatography
UV	Ultraviolet
XIC	Extracted Ion Chromatogram

Synthetic Description

The first compounds to be prepared (Scheme S1) focused on direct modification of alkene triester **17**.[1] Thus, diol **18** was prepared through Upjohn dihydroxylation of **17** in moderate yield as a complex diastereomeric mixture. Diol **18** was subsequently oxidized using pyridinium chlorochromate (PCC) to afford acyloin **19** in moderate yield. Preparation of an alcohol proved somewhat more challenging, with various hydroboration reagents (9-BBN, BH_3-DMS, BH_3-THF) returning only alkene starting material **17**. Instead, successful production of alcohol **21** first proceeded through epoxidation of alkene **17** using meta-chloroperbenzoic acid to afford epoxide **20** in excellent yield. Ring-opening of the epoxide was unsuccessful using either standard hydride reduction ($NaCNBH_3$, $NaBH_4$), or through attempting to form the tertiary carbocation and reacting it with a silane hydride reagent (BF_3-THF or trifluoroacetic acid, $HSiEt_3$ or $HSiPh_3$). Instead, radical mediated ring opening of epoxide using in situ generated bis(cyclopentadienyl)titanium(III) chloride proved successful, delivering the alcohol **3** in good yield. The final compound prepared directly from this scaffold was ketone **22**, provided through the oxidation of alcohol **21** using PCC in moderate yield.

***Scheme S1:** Synthetic route for compounds **18**-**22**.*

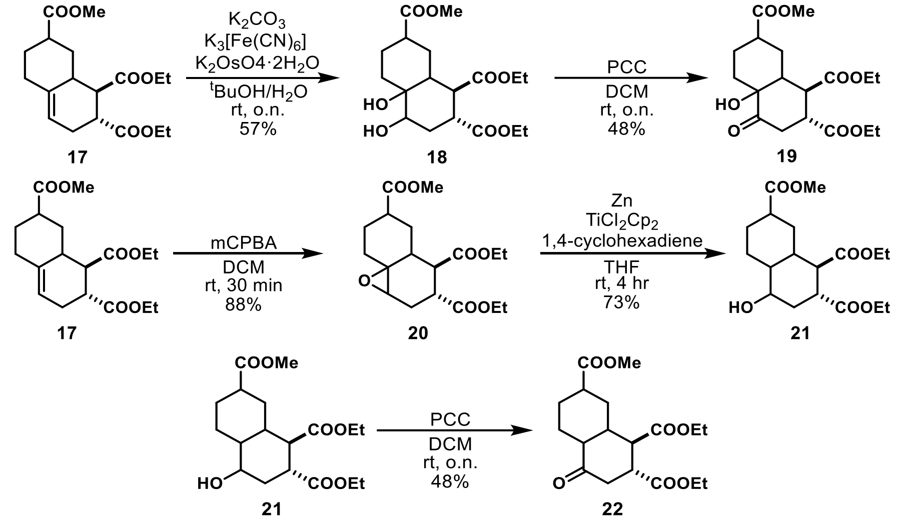

Next targeted was a methyl ether functionality, with initial attempts focusing on alkylation of alcohol **19**. Treatment with methyl iodide provided only starting material, even when a large excess of methyl iodide (10 eq.) was employed at 150 °C in dimethylformamide for extended periods of time. Similarly, the use of sodium hydride and methyl iodide at reflux in tetrahydrofuran returned only starting material. Attempts to use more aggressive methylating reagents such as methyl triflate and trimethyloxonium tetrafluroborate once again delivered only starting material. Instead, it was envisioned that the preparation of a methyl-ether diene that could undergo Diels-Alder reaction to form the desired tri-ester methyl ether scaffold would allow for the preparation of a methyl-ether CRAM analogue.

Preparing such a diene began (Scheme S2) with acid **23** being subjected to Fischer esterification to afford ester **24** in good yields. Subsequent aromatic hydrogenation using rhodium on carbon at 100 °C provided alcohol **25**, which was immediately subjected to subsequent reaction. Notably, the use of methanol and acetic acid as solvents, even at temperatures exceeding 100 °C (achievable under pressure) did not deliver complete reduction of aromatic ester **24**, while the use of ethyl acetate and acetic acid led to complete reduction of the aromatic functionality. Next, the crude mixture containing **25** was subjected to oxidation with PCC to afford *syn*-ketone **26** in 20% yield over two steps. The *anti*-compound was also delivered in a separate fraction, but was inseparable from methyl 4-

oxocyclohexanecarboxylate, presumed to result from elimination and reduction during the prior hydrogenation step, and as such was not employed in the formation of an additional *anti*-diene.

***Scheme S2:** Synthetic route for compounds **24**-**26**.*

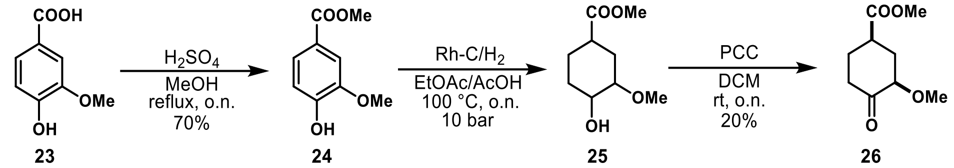

Subsequently (Scheme S3), *syn*-ketone **26** was treated with sodium hexamethyldisilazane (NaHMDS) and *N*-(5-chloropyridin-2-yl)-1,1,1-trifluoro-*N*-((trifluoromethyl)sulfonyl)methanesulfonamide (Comin's reagent) to afford vinyl triflate **27**, that was immediately subjected to palladium catalysed cross-coupling to provide diene **29**. This reaction was particularly poor yielding, with several unidentified alkenes and dienes being generated separately to the desired diene, presumably due to the lability of the vinylic methyl ether functionality. The first attempt to form diene **29** utilized Stille coupling as was employed in our prior preparation of CRAM analogues, but the instability of diene **29** meant that tributyl tin hydroxide contamination was extreme, with the sample containing less than 5% desired product by mass. Fortunately, additional method development focusing on Suzuki coupling had led to successful cross-coupling using vinyl boron pinacolate (vinyl Bpin) **28**, providing an option that avoided the use of tributylvinyl tin. Using this Suzuki reaction did provide an additional unidentified by-product that was inseparable from diene **29**, but data comparison between the Stille and Suzuki couplings identified the generation of diene **29**, albeit in extremely poor yield.

***Scheme S3:** Synthetic route to compounds **27** and **29**.*

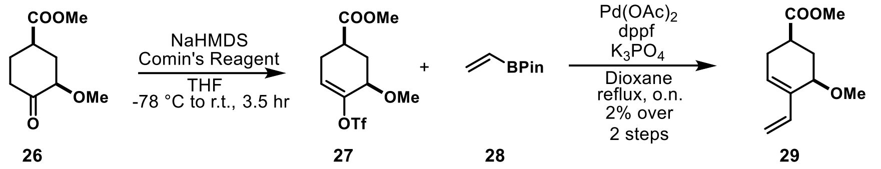

Next (Scheme S4), Diels-Alder reaction of diene **29** and diethyl fumarate **30** provided alkene **31** in moderate yield, before hydrogenation using Adam's catalyst provided alkane **32**, which was directly subjected to hydrolysis (vide infra). Once again, the lability of the vinyl methyl ether proved problematic, with approximately 20% of *des*-methyl alkene **33** being simultaneously produced as identified upon LCHRMS, MS2, and CAD investigation.

***Scheme S4:** Synthetic route to compounds **31**-**33**.*

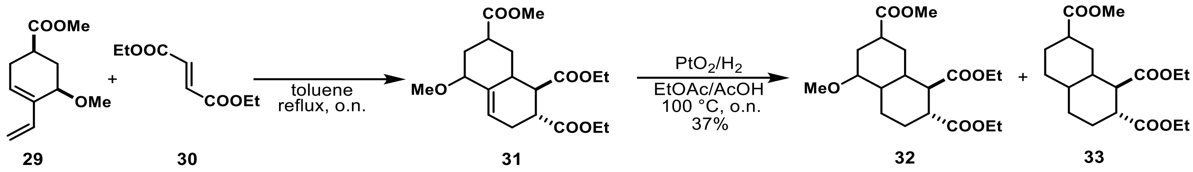

Production of a lactone functionalized CRAM, as well as the tertiary alcohol equivalent of secondary alcohol **9**, came serendipitously (Scheme S5). In a separate project, large scale preparation of alkene **34** was desired, such that after hydrolysis it could be purified by preparative high-throughput liquid chromatography (HPLC) to afford separated diastereomers. Simultaneously, we had discovered that several of our more polar CRAM analogues could not be reliably extracted into organic solvents after hydrolysis, and had turned to the use of the styrene-divinylbenzyl (SDVP) Agilent Bond Elut PPL solid-phase extraction (SPE) cartridges, commonly used in the extraction of DOM and other fulvic acids. This provided access to these materials, and also improved the yields of alcohol CRAM compounds. As such,

we decided that after hydrolysis of alkene **34**, we would extract using these PPL cartridges, to attempt to improve how much material we could recover. Surprisingly, we found near complete loss of the alkene functionality upon crude ^{1}H NMR analysis, and upon preparative HPLC, ultimately recovered lactone diacid **7**. This compound was not observed upon standard acidification and extraction into ethyl acetate, even though this extraction was at lower pH (pH 1 for organic extraction, pH 2 for SPE extraction), suggesting some catalytic capacity for this SDVP resin. In addition to this diacid lactone **7**, tertiary alcohol **9** was also provided through standard hydrolysis in good yields.

Scheme S5: *Synthetic route to compounds **7** and **9**.*

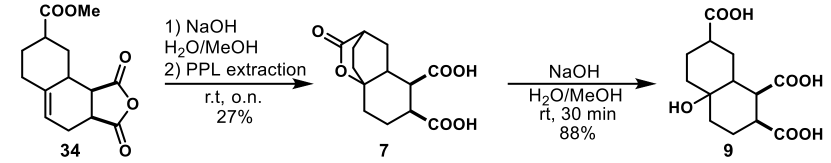

Several di-ester compounds were also produced, utilizing the optimized Suzuki cross-coupling conditions mentioned above. As part of this modified method (Scheme S6), benzyl ester **37** was initially prepared through 1-ethyl-3-(3-dimethylaminopropyl)carbodiimide mediated esterification of acid **35**, to allow for improved identification during synthetic modification. Standard triflation using NaHMDS and Comin's reagent provided vinyl triflate **38** in good yield. Finally, anhydrous Suzuki cross-coupling using tripotassium phosphate and vinyl Bpin **28** delivered benzyl-ester diene **39** in moderate yield. As a notable advantage to our previously disclosed method, vinyl Bpin could simply be removed using reduced pressure on a rotary evaporator, in comparison with Stille by-product $SnBu_3OH$, which caused significant contamination problems.

Scheme S6: *Synthetic route to compounds **37**-**39**.*

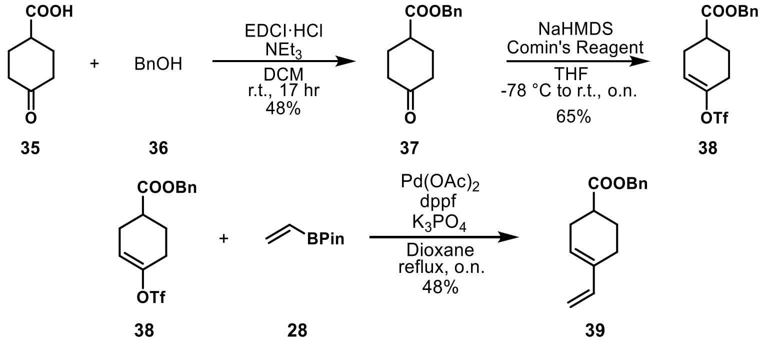

Following this (Scheme S7), diene **39** was subjected to Diels Alder reaction using both methyl acrylate **40**, and ethyl crotonate **42**. Exceedingly high temperatures were required for both reactions, with pressure vessels required to achieve 160 °C for the production of **41**, and 180 °C for **43**. In both cases, regioisomeric mixtures were delivered even after purification, with diesters **41a** and **41b** delivered from **40** and methyl diesters **43a** and **43b** from **42**. Initially, **43** was seen as a more desirable candidate for comparison with our previous compounds, as the inclusions of the methyl functionality meant that final CRAM analogues would contain thirteen carbon atoms, the same as the triacid CRAM analogues from our original method. As such, hydrogenation of **43** using Adam's catalyst delivered alkanes **44a** and **44b** with the corresponding loss of their benzyl functionalities from hydrogenative debenzylation. Next, Upjohn dihydroxylation of both diester mixtures was performed to provide again inseparable regioisomeric mixtures of diols **45a** and **45b**, and methyl diols **46a** and **46b** in moderate yields.

Scheme S7: *Synthetic route to compounds* ***41-46***.

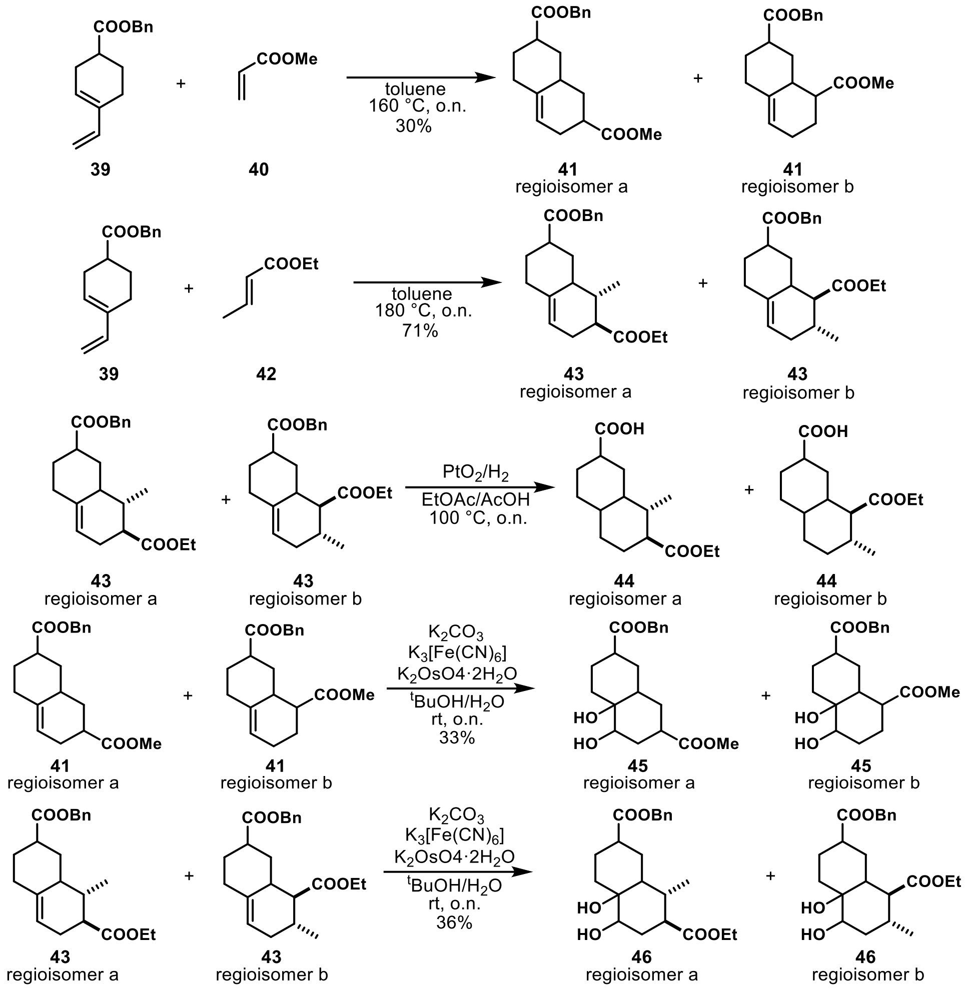

Turning to the hydrolyses of our esters to form their corresponding CRAM analogues (Scheme S8), hydrolysis of methyl diester **44** led to only partial hydrolysis according to ^{1}H NMR analysis, even at 80 °C. This same result was observed in the hydrolysis of diol diester **46**, and in this case, raising the hydrolysis temperature to 100 °C led to complete decomposition of the starting material and any product that had been generated at lower temperatures. Conversely, hydrolysis of *des*-methyl diol **45** provided their corresponding diacids **14a** and **14b** smoothly at 60 °C, with no ester functionality observed in the ^{1}H NMR spectrum. We presumed this was due to the relatively hydrophobic α-methyl functionality blocking the approach of a hydroxide anion for **44a**, **44b**, **46a**, and **46b**, perhaps with some reduced reactivity for compounds **44a** and **46a** due to the increased steric hinderance around the ethyl ester in these cases. Fortunately, the use of LCMS methods during analysis still allowed for their investigation in the context of this work, as well as of esters **16a** and **16b**, but their incomplete reaction and existence in a complex mixture does reduce the accuracy of their structural elucidation. As mentioned, the incorporation of the methyl functionality was preferred, but the combination of successful hydrolysis of **45**, and the ability to observe diols **6**, **15**, and **16** using LCMS and LCMS2 allowed for the investigation of compounds with these functional group compositions.

Scheme S8: *Synthetic route to compounds **6**, **14-16**, and **47**.*

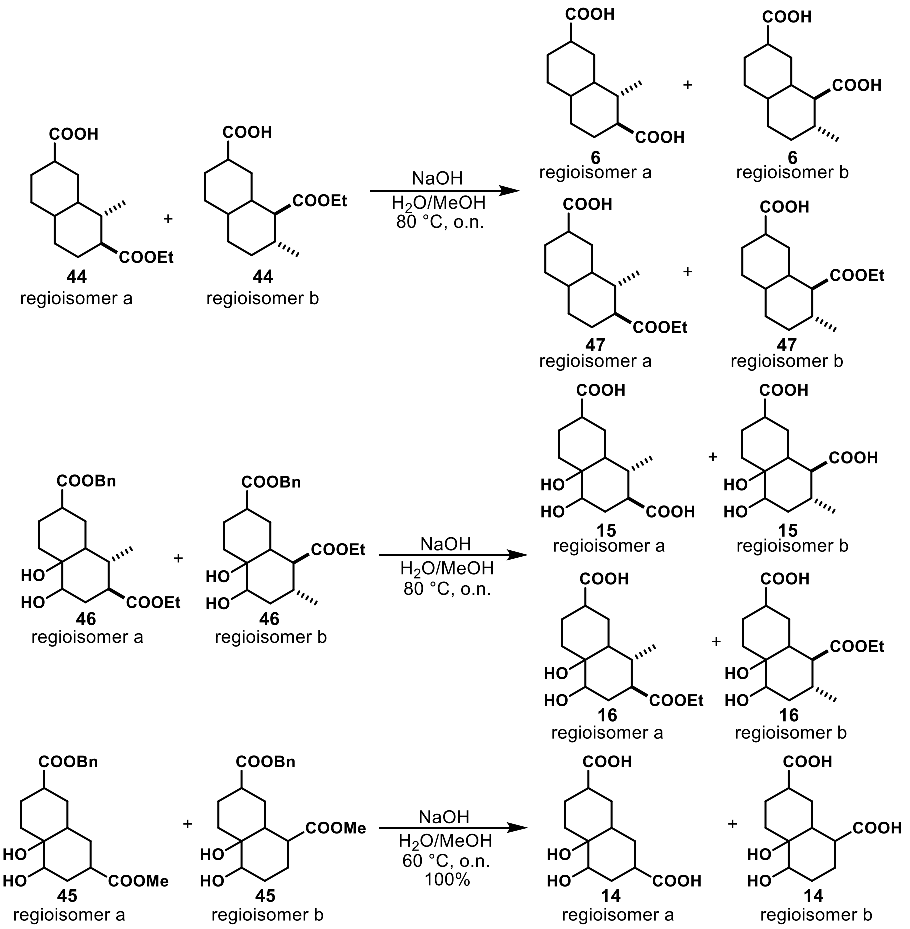

Lastly (Scheme S9), all remaining tri-esters **18**, **19**, **21**, **22** and **31**, and a commercially purchased di-ester **48** were subjected to ester hydrolysis. Alcohol **21** delivered its triacid counterpart **8** smoothly, with PPL extraction providing excellent yield. Similarly, two separately isolated diastereomeric mixtures of ketone **22** readily provided their corresponding triacids **11**, in good to excellent yield. While hydrolysis of α-hydroxy ketone **19** also provided its triacid **12** in excellent yields, it proved unstable overnight in methanol, rending full NMR analysis difficult. It was found to be similarly unstable in water for LCMS experiments over the course of a day, but could be analysed shortly after fresh preparation. Ether **10** and contaminant alkane **32** provided their counterparts **10** and **47**, with MS2 data confirming our suspicion of the methyl ether loss for around 20% of the material. Diol **18** proved somewhat problematic, as less than 1% of starting mass could be recovered by organic extraction. Utilization of PPL extraction led to transformation of a sizable (greater than half) portion of the material, and isolation of the compound **13** required preparative HPLC. This again raises the possibility for catalytic transformation of CRAM-like molecules by SDVP resins, and is something we intend to explore more generally in the future. Finally, commercial diester **48** readily delivered its corresponding diacid **5** in excellent yield.

Scheme S9: *Synthetic route to compounds* ***5***, ***8***, ***10-13***, *and* ***48***.

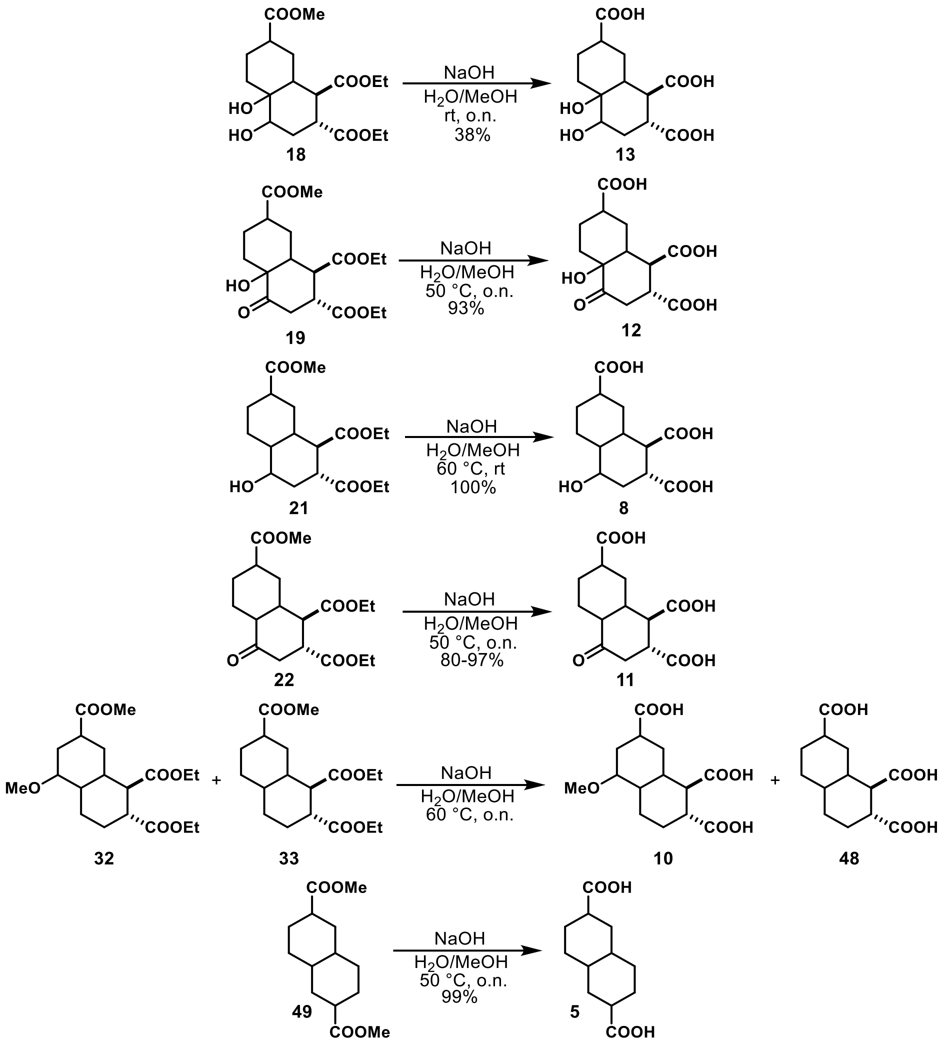

General Methods

Thin-layer chromatography (TLC) was performed on 0.2 mm aluminium plates precoated with silica gel 60 F_{254} (Merck). Compounds were visualized with an ultraviolet (UV)-light, and stained with potassium permanganate. Column chromatography was performed with silica gel (40 – 63 μM). High resolution mass-spectra to obtain accurate masses of intermediate and final compounds were recorded on a LTQ Velos Pro Orbitrap (Thermo Fisher), with samples being analysed by direct infusion with electrospray ionization in negative mode (final compounds) or by liquid chromatography mass spectrometry (LCMS) with electrospray ionisation in positive or negative mode (intermediates). Liquid chromatography was conducted at a flow rate of 350 µl/min on C18 (Phenomenex Kinetex 150x2.1 mm, 1.7 µm pore size), in a linear gradient running from 5-95% mobile phase B, where A was 0.1% formic acid in deionised water (milliQ, Millipore), and B was acetonitrile (LiChrosolv, Merck) with 0.1% formic acid. Data were collected at 100,000 resolution (at *m/z* 400) and 50 transients were co-added for direct infusion data. ^{1}H NMR spectra were recorded at 400 MHz on a Varian Mercury Plus spectrometer, or at 600 MHz on a Bruker Avance Neo spectrometer with a TCI (CRPHe TR-1H &19F/13C/15N 5mm-EZ) probe. All spectra were recorded from samples in either $CDCl_3$ or MeOD, at room temperature in 5 mm nuclear magnetic resonance (NMR) tubes. Chemical shifts are reported relative to the residual solvent peak at δ 7.26 for $CDCl_3$ or δ 3.31 for MeOD. Resonances were assigned as follows: chemical shift (multiplicity, number of protons, coupling constant(s)). Multiplicity abbreviations are reported by the conventions: s (singlet), d (doublet), dd (doublet of doublets), dt (doublet of triplets), t (triplet), app t (apparent triplet), m (multiplet). Proton decoupled ^{13}C NMR spectra were recorded at 101 MHz on a Varian Mercury Plus spectrometer under the same conditions as for the ^{1}H NMR spectra, or at 600 MHz on a Bruker Avance Neo spectrometer with a TCI (CRPHe TR-1H &19F/13C/15N 5mm-EZ) probe under the same conditions as for the ^{1}H NMR spectra. Chemical shifts have reported relative to the residual solvent peak at δ 77.16 for $CDCl_3$ or δ 49.90 for MeOD. All solvents and reagents were used as received. Compound purities are reported based on integrated peak areas from charged aerosol detection (CAD) data using a Thermo Vanquish UPLC coupled CAD analyser with the gradient LCMS method as reported in the main text (see supporting information spectral file for compound purities). Novel compounds are denoted in italics, while previously reported compounds are not.

NMR Analysis Information

The final carboxylate rich alicyclic molecule (CRAM) compounds **5**-**16** were complicated mixtures with major, minor, and trace diastereomeric peaks. For practical data reporting purposes, ^{1}H NMR integrals and peaks are largely arbitrary, aiming to define the amount of integral across a region between two relative minima, instead of integration occurring across every peak that represents a single chemical environment. Furthermore, a consistent but arbitrary manual threshold was used for ^{13}C NMR peak picking, such that anyone wishing to replicate this work can cross reference their own spectra for the highest intensity carbon NMR signals. Similarly, we are aware that different fields may be interested in using this data in different ways. Thus, for all displayed NMR data of final CRAM compounds **5**-**16**, a series of spectral windows are shown for each type of NMR experiment. NMR spectral data for all compounds can be found in the additional spectral data supplementary information file.

^{1}H NMR spectra of each compound are reported with both a broad spectral window (0–10 ppm), and a narrower spectral window (0.5-4.2 ppm).

^{13}C NMR spectra of each compound are reported with: a broad spectral window (0–200 ppm), a spectrum highlighting the carboxylate region (170–190 ppm), and a spectrum highlighting sp^3 carbon functionalities (10-80 ppm).

For correlation spectroscopy (COSY) spectra, the first spectrum shows the ^{1}H NMR range from 0-10 ppm, and the second shows the spectra from 0.5 to 4.2 ppm. For heteronuclear single quantum correlation (HSQC) spectra, the first spectrum shows the ^{1}H NMR range from 0-10 ppm, and the ^{13}C NMR range from 0-160 ppm, while the second spectrum shows ^{1}H NMR range from 0.5-4.2 ppm, and the ^{13}C NMR range from 10-80 ppm. For heteronuclear multiple bond correlation (HMBC) spectra, the first spectrum shows the ^{1}H NMR range from 0-10 ppm, and the ^{13}C NMR range from 0-210 ppm, the second spectrum shows ^{1}H NMR range from 0.5-4.2 ppm, and the ^{13}C NMR range from 170-190 ppm, and the third spectrum shows ^{1}H NMR range from 0.5-4.2 ppm, and the ^{13}C NMR range from 10-80 ppm.

Analytical Liquid Chromatography

Liquid chromatography was conducted with a Thermo Vanquish UPLC using a Phenomenex Kinetex C18 column (2.1x150 mm, 2.6 µm) at a flow rate of 0.5 ml/min. Mobile phase A was 0.1% formic acid (AnalaR Normapur, VWR) in deionized grade water (Millipore MilliQ), B was 0.1% formic acid in LCMS grade acetonitrile (Lichrosolv, Supelco, Merck). A linear gradient started at 5% B then increased at 1 min from 5 to 100% B at 10 min, followed by a 2.1 min washout phase at 100% B, a decrease to 5% B and a 2.7 min equilibration phase at 5% B (method length = 15 min). The column oven was set to 40 °C to decrease back pressure, and the autosampler chamber to 5 °C.

Analytical Mass spectrometry

Mass spectrometry was conducted with an Orbitrap Q Exactive (Thermo Fisher). Electrospray ionization was used as the ionization source, using a heated unit running at 200 °C and -3.5 kV (negative mode). Sheath gas and auxiliary gas were set to 20 and 2 units, respectively, S-Lens was set to 50, and capillary temperature was set to 300 °C. For the experiments, resolution was set to 35,000, and a maximum injection time of 200 ms was chosen, aiming to trap 3 x 10^6 ions in the orbitrap. MS2 experiments were conducted at normalized collision energies of 35 and 75 by higher energy collision dissociation (HCD) experiments using the PRM method, and 2 x 10^5 ions were targeted using automatic gain control for these experiments, with a maximum trapping time of 100 ms. An isolation window of 1 Da was selected, centered on the deprotonated mass of the compound being investigated.

Synthetic Procedures for the Preparation of Intermediates 18-46

Diol triester **18**

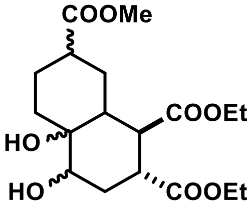

To a solution of alkene **17** (0.385 g, 1.14 mmol) in a 1:1 mixture of ^tBuOH and water (20 mL) was added potassium osmate dihydrate (0.042 g, 0.11 mmol), potassium ferricyanate (0.749 g, 2.28 mmol) and potassium carbonate (0.314 g, 2.28 mmol). The mixture was stirred overnight, before being diluted into ethyl acetate, washed with distilled water (x2), and brine (x1). The resulting solution was dried over Na_2SO_4, filtered, and concentrated in vacuo. The crude mixture was subjected to silica gel column chromatography (EtOAc: PET, 1:19 to 3:1) to afford the title compound as a mixture of diastereomers as an opaque semi-solid (0.245 g, 57%).

^1H NMR (400 MHz, $CDCl_3$) δ 4.27-4.04 (m, 4H), 4.02-3.85 (m, 0.5H), 3.72-3.61 (m, 3H), 3.51-3.34 (m, 0.5H), 3.24-2.62 (m, 2H), 2.53-2.13 (m, 3H), 2.13-1.89 (m, 2H), 1.89-1.62 (m, 4H), 1.61-1.31 (m, 3H), 1.30-1.19 (m, 6H). ^{13}C NMR (101 MHz, $CDCl_3$) δ 176.2, 175.5, 175.4, 174.6, 174.51, 174.47, 174.4, 174.0, 173.81, 173.78, 173.3, 77.5, 77.4, 77.2, 76.8, 73.8, 72.4, 72.2, 71.9, 71.2, 70.8, 65.83, 65.77, 61.1, 60.93, 60.89, 60.78, 60.76, 60.72, 60.68, 60.6, 53.5, 51.9, 51.82, 51.76, 51.7, 45.97, 45.95, 43.9, 43.8, 43.7, 43.0, 42.8, 42.7, 41.4, 40.5, 39.9, 39.1, 39.0, 38.6, 38.4, 37.1, 34.5, 32.6, 32.2, 31.9, 31.5, 31.4, 29.3, 27.3, 26.2, 26.0, 25.9, 25.6, 24.1, 23.7, 21.7, 15.1, 14.3, 14.23, 14.19, 14.17, 14.1. HRMS (ESI-MS) calculated for **18** $C_{18}H_{29}O_8^+$ $[M + H]^+$: 373.1857; found: 373.1848.

α-hydroxy ketone **19**

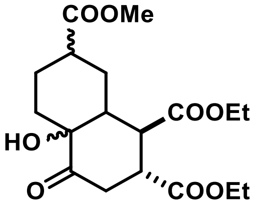

To a solution of diol **18** (0.159 g, 0.427 mmol) in DCM (15 mL) was added pyridinium chlorochromate (0.138 g, 0.640 mmol). The mixture was stirred at room temperature overnight, before being filtered and concentrated in vacuo. The crude reaction mixture was subjected to silica gel column chromatography (EtOAc:PET, 1:19 to 2:3) to afford the title compound (0.087 g, 54%) as a pale-yellow solid . ^1H NMR (400 MHz, $CDCl_3$) δ 4.28-4.09 (m, 4H), 3.86-3.80 (m, 1H), 3.74-3.61 (m, 3H), 3.23-3.07 (m, 1H), 3.07-2.94 (m, 1H), 2.86-2.63 (m, 1H), 2.63-2.41 (m, 2H), 2.41-2.01 (m, 3H), 2.00-1.59 (m, 3H), 1.51-1.32 (m, 1H), 1.30-1.22 (m, 6H). ^{13}C NMR (101 MHz, $CDCl_3$) δ 210.1, 207.3, 206.6, 175.4, 174.8, 174.3, 173.23, 173.17, 173.0, 172.8, 172.3, 171.8, 76.0, 75.6, 75.2, 61.3, 61.24, 61.15, 51.97, 51.95, 51.9, 45.8, 45.7, 45.3, 43.1, 42.6, 42.4, 42.1, 40.4, 40.2, 38.8, 38.7, 38.3, 36.6, 33.5, 31.0, 27.1, 26.6, 26.2, 25.8, 24.5, 14.31, 14.29, 14.2. HRMS (ESI-MS) calculated for **19** $C_{18}H_{27}O_8^+$ $[M + H]^+$: 371.1700; found: 371.1696.

Epoxide **20**

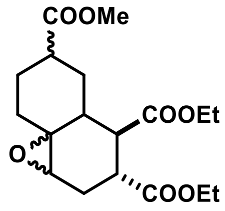

To a solution of alkene **17** (0.721 g, 2.13 mmol) in DCM (20 mL) was added mCPBA (0.552 g, 3.19 mmol) at room temperature under a nitrogen atmosphere. The mixture was stirred for 30 minutes, before being diluted into DCM and washed with 0.1 M aqueous NaOH (x2), distilled water (x1) and brine (x1). The resulting organic solution was dried over Na_2SO_4, filtered, and concentrated in vacuo to afford the title compound as a mixture of diastereomers as an opaque semi-solid (0.672 g, 88%).

^{1}H NMR (400 MHz, $CDCl_3$) δ 4.28-4.02 (m, 4H), 3.72-3.67 (m, 3H), 3.19-3.02 (m, 1H), 3.00-2.60 (m, 2H), 2.52-2.29 (m, 2H), 2.18-1.59 (m, 6H), 1.56-1.34 (m, 1H), 1.29-1.16 (m, 7H). ^{13}C NMR (101 MHz, $CDCl_3$) δ 174.97, 174.94, 174.8, 174.7, 174.6, 174.4, 174.1, 173.9, 173.7, 173.4, 173.2, 77.4, 77.2, 77.0, 76.7, 62.5, 62.2, 61.7, 60.93, 60.85, 60.83, 60.78, 60.72, 60.68, 60.66, 60.6, 59.8, 58.2, 58.1, 57.6, 57.4, 51.88, 51.85, 51.8, 45.1, 44.9, 42.5, 42.3, 41.9, 41.8, 41.63, 41.57, 39.7, 38.9, 38.5, 37.1, 36.9, 34.9, 34.7, 34.3, 33.3, 32.7, 31.0, 30.7, 30.4, 29.5, 28.2, 27.5, 27.1, 27.0, 26.9, 26.8, 26.5, 25.9, 24.3, 14.23, 14.21, 14.18, 14.10, 14.09. HRMS (ESI-MS) calculated for **20** $C_{18}H_{27}O_7^+$ $[M + H]^+$: 355.1751; found: 355.1745.

Alcohol triester **21**

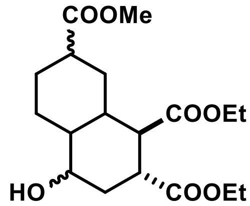

To a dry round bottom flask under a nitrogen atmosphere was added Zn powder (0.818 g, 12.5 mmol) and titanocene dichloride (1.04 g, 4.17 mmol), and the flask was pump-purged twice with nitrogen gas. Dry, degassed THF (20 mL) was added, and the suspension was stirred for half an hour, in which time a deep-green colour developed. In this time, to a dry round bottom flask under a nitrogen atmosphere was added epoxide **20** (0.672 g, 1.89 mmol), 1,4-cyclohexadiene (0.34 g, 0.40 mL, 4.2 mmol), and dry, degassed THF (10 mL). To this flask was added the deep-green solution dropwise over 10 minutes, before the combined solutions were stirred for a further 4 hours. At completion, the mixture was diluted into ethyl acetate, and washed with distilled water (x2). The aqueous portions were combined, and extracted with ethyl acetate (x1), before the organic portions were combined, washed with distilled water (x1) and brine (x1). The resulting organic solution was dried over Na_2SO_4, filtered, and concentrated in vacuo. The crude mixture was subjected to silica gel column chromatography (EtOAc: PET, 1:19 to 3:2) to afford the title compound as a mixture of diastereomers as a clear semi-solid (0.498 g, 73%).

^{1}H NMR (400 MHz, $CDCl_3$) δ 4.23-3.94 (m, 4H), 3.89-3.74 (m, 0.5H), 3.64-3.56 (m, 3H), 3.43-2.98 (m, 0.5H), 2.84-2.50 (m, 2H), 2.48-2.11 (m, 3H), 2.10-1.70 (m, 3H), 1.69-1.22 (m, 4H), 1.22-1.13 (m, 6H), 1.09-0.86 (m, 1H). ^{13}C NMR (101 MHz, $CDCl_3$) δ 176.3, 175.8, 175.7, 175.5, 175.42, 175.40, 175.38, 175.0, 174.9, 174.8, 174.7, 174.6, 174.51, 174.45, 174.3, 174.23, 174.16, 174.1, 173.74, 173.73, 173.5, 173.4, 173.3, 173.2, 173.1, 173.0, 72.5, 72.4, 70.8, 70.4, 70.1, 68.5, 68.3, 64.9, 64.8, 61.4, 61.3, 61.1,

60.87, 60.85, 60.8, 60.7, 60.61, 60.60, 60.5, 60.4, 58.0, 53.5, 52.1, 51.82, 51.78, 51.76, 51.72, 51.70, 51.64, 51.61, 51.4, 51.1, 50.7, 50.6, 48.4, 48.2, 48.1, 47.5, 45.1, 44.7, 44.5, 44.3, 44.1, 43.91, 43.85, 43.5, 43.3, 43.2, 42.93, 42.90, 42.7, 42.6, 42.39, 42.36, 41.9, 41.6, 41.3, 41.2, 40.4, 40.3, 40.1, 39.9, 39.4, 39.3, 39.03, 39.00, 38.8, 38.6, 38.5, 38.4, 38.1, 38.0, 37.9, 37.7, 37.4, 37.3, 36.9, 36.81, 36.75, 35.9, 35.8, 35.6, 34.9, 33.3, 33.1, 32.6, 31.8, 31.6, 31.4, 31.1, 30.4, 29.7, 29.4, 28.5, 28.24, 28.18, 28.0, 27.9, 27.7, 27.5, 26.8, 26.39, 26.35, 26.1, 25.9, 25.7, 25.1, 24.6, 24.4, 24.34, 24.28, 23.1, 22.4, 21.2, 18.4, 14.24, 14.22, 14.17, 14.14, 14.12, 14.10, 14.09, 14.07. HRMS (ESI-MS) calculated for **21** $C_{18}H_{29}O_7^+$ $[M + H]^+$: 357.1908; found: 357.1898.

Ketone triester **22**

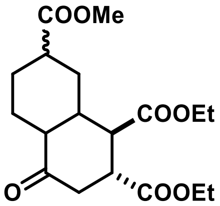

To a solution of alcohol **21** (0.188 g, 5.28 mmol) in DCM (10 mL) was added pyridinium chlorochromate (0.227 g, 1.06 mmol). The mixture was stirred overnight, and TLC indicated the presence of starting material. Additional pyridinium chlorochromate (0.113 g, 0.617 mmol) was added, and the mixture was stirred for a further 6 hours. At completion, the mixture was filtered with DCM, and the resulting solution washed with distilled water (x2), followed by brine (x1). This solution was dried over Na_2SO_4, filtered, and concentrated in vacuo. The crude mixture was subjected to silica gel column chromatography (EtOAc: PET, 1:19 to 2:3) to afford a single diastereomer (0.014 g, 7%), and a mixture of inseparable diastereomers (0.078 g, 41%).

Single isomer: ^{1}H NMR (400 MHz, $CDCl_3$) δ 4.31-4.10 (m, 4H), 3.68 (s, 3H), 3.30 (dd, 1H, *J* = 12.1, 4.3 Hz), 3.20 (ddd, 1H, *J* = 13.5, 12.2, 4.7 Hz), 2.86-2.79 (m, 1H), 2.75-2.70 (m, 1H), 2.66 (m, 2H), 2.34 (ddd, 1H, *J* = 14.5, 13.5, 1.0 Hz), 2.20-2.12 (m, 1H), 2.02-1.94 (m, 1H), 1.86-1.76 (m, 1H), 1.70-1.61 (m, 1H), 1.49-1.39 (m, 1H), 1.31-1.16 (m, 7H). Single isomer: ^{13}C NMR (101 MHz, $CDCl_3$) δ 208.0, 174.7, 173.5, 172.2, 61.3, 51.8, 48.4, 47.6, 42.6, 40.6, 38.9, 36.8, 26.3, 22.6, 21.5, 14.2. Mixture of diastereomers: ^{1}H NMR (400 MHz, $CDCl_3$) δ 4.29-4.06 (m, 4H), 3.69-3.60 (m, 3H), 3.17-3.04 (m, 1H), 2.80-2.33 (m, 4H), 2.33-2.14 (m, 1H), 2.13-1.82 (m, 3H), 1.77-1.66 (m, 1H), 1.62-1.33 (m, 2H), 1.30-1.19 (m, 6H). Mixture of diastereomers: ^{13}C NMR (101 MHz, $CDCl_3$) δ13C NMR (101 MHz, CDCl3) δ 209.5, 207.3, 207.1, 175.3, 175.2, 174.3, 173.4, 172.8, 172.32, 172.30, 172.1, 171.9, 61.4, 61.3, 61.1, 60.9, 52.2, 51.8, 51.7, 51.2, 50.70, 50.67, 45.6, 45.3, 44.7, 43.3, 43.01, 42.96, 42.5, 42.1, 42.03, 41.97, 40.40, 40.35, 38.8, 38.5, 37.4, 36.9, 33.5, 32.3, 30.4, 27.52, 27.45, 25.9, 24.3, 24.1, 21.5, 14.20, 14.17, 14.10, 14.05. HRMS (ESI-MS) calculated for **22** $C_{18}H_{27}O_7^+$ $[M + H]^+$: 355.1751; found: 355.1749.

Ester **24**

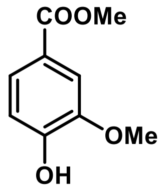

To a solution of vanillic acid (4.0 g, 24 mmol) in methanol (100 mL) was added sulfuric acid (0.5 mL) at room temperature. The mixture was heated to reflux and stirred overnight, before being cooled to room temperature, and concentrated in vacuo. The resulting mixture was diluted into ethyl acetate, washed with distilled water (x3) followed by brine (x1), dried over Na_2SO_4, and concentrated in vacuo to afford the title compound (3.1 g, 70%) as a brown oil. The ^{1}H NMR data matched that reported in the literature.[2]

^{1}H NMR (400 MHz, $CDCl_3$) δ 7.63 (dd, 1H, J = 8.3, 1.9 Hz), 7.55 (d, 1H, J = 1.9 Hz), 6.94 (d, 1H, J = 8.3 Hz), 6.02 (br s, 1H), 3.94 (s, 3H), 3.89 (s, 3H).

Alcohol **25**

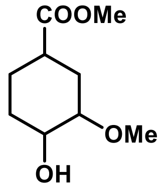

A solution of ester **24** (4.1 g) in a 4:1 mixture of EtOAc:AcOH (35 mL) was split into seven vials, before Rh-C (50 mg) was added to each vial. The vials were placed in a high-pressure vessel, and the atmosphere was replaced with hydrogen gas (10 bar). The vessel was heated to 100 °C overnight before being cooled to room temperature. The samples were combined and filtered through Celite using EtOAc, before being concentrated in vacuo to afford a pale-yellow oil that was immediately reacted in the subsequent step.

Ketone **26**

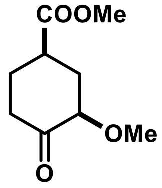

To the crude mixture from the previous step (3.15 g) in dichoromethane (100 mL) was added pyridinium chlorochromate (5.41 g, 25.1 mmol) at room temperature. The mixture was stirred overnight, before being filtered and concentrated in vacuo. The crude mixture was subjected to silica gel column chromatography (EtOAc:PET, 1:19 to 1:1) to afford the title compound as a single diastereomer (0.851 g, 20% over two steps). An additional fraction was collected containing the *anti*-diastereomer, but was contaminated with methyl 4-oxocyclohexanecarboxylate and was not fully characterized.

^{1}H NMR (400 MHz, $CDCl_3$) δ 3.80 (ddd, 1H, *J* = 11.8, 5.9, 1.1 Hz), 3.71 (s, 3H), 3.44 (s, 3H), 2.83 (dddd, 1H, *J* = 11.7, 11.7, 3.7, 3.7 Hz), 2.60-2.52 (m, 2H), 2.36 (dddd, 1H, *J* = 13.9, 13.2, 5.9, 1.3 Hz), 2.29-2.22 (m, 1H) 1.93-1.83 (m, 2H). ^{13}C NMR (101 MHz, $CDCl_3$) δ 207.8, 173.7, 82.6, 58.1, 52.2, 40.7, 39.0, 35.9, 29.5. HRMS (ESI-MS) calculated for **26** $C_9H_{15}O_4^+$ $[M + H]^+$: 187.0965; found: 187.0961.

Triflate **27**

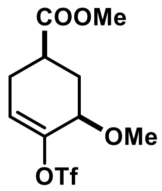

To a solution of ketone **26** (0.425 g, 2.28 mmol) and *N*-(5-Chloropyridin-2-yl)-*N*-(methanesulfonyl)methanesulfonamide (Comin's reagent, 0.986 g, 2.51 mmol) in dry THF (10 mL) was added 1M sodium hexamethyl disilazane (NaHMDS, 2.51 mmol, 2.51 mL) at – 78 °C under nitrogen. The mixture was stirred for an hour, before being warmed to room temperature and stirred for a further 2.5 hours. At completion, saturated aqueous ammonium chloride (5 mL) was added, before the mixture was concentrated in vacuo. The mixture was dissolved in ethyl acetate, washed with distilled water (x2) followed by brine (x1), before being dried over Na_2SO_4¸ and concentrated in vacuo. The crude mixture was immediately subjected to the following reaction.

Diene **29**

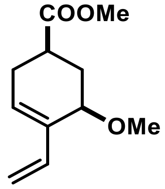

To a solution of triflate **27** (2.28 mmol, assumed from 100% conversion from previous reaction) $Pd(OAc)_2$ (0.026 g, 0.11 mmol), 1,1'-bis(diphenylphosphino)ferrocene (0.063 g, 0.11 mmol), and K_3PO_4 (1.45 g, 6.84 mmol) in dry degassed dioxane (10 mL) was added vinylboronic acid pinacol ester (0.351 g, 0.39 mL, 2.28 mmol) under a nitrogen atmosphere. The mixture was heated to reflux and stirred overnight, before being cooled to room temperature, and filtered through Celite with diethyl ether. The mixture was washed with distilled water (x2), before the aqueous portions were combined and extracted with diethyl ether (x2). The organic portions were combined, washed with distilled water (x1) followed by brine (x1), before being dried over Na_2SO_4, and concentrated in vacuo. The crude mixture was subjected silica gel chromatography (Et_2O: PET, 1:19 to 1:1) to afford a fraction that contained the title product and an unidentified impurity (57 mg). Previous Stille coupling had afforded a mixture after column chromatography (4 mg) which contained a small amount of the diene contaminated with approximately 1.5 equivalents of $SnBu_3OH$. The ^{1}H NMR spectra of both the fraction collected here and from that Stille coupling (both provided below) have been used to infer ^{1}H NMR data of diene **27**.

^{1}H NMR (400 MHz, $CDCl_3$) δ 6.26 (dd, 1H, *J* = 17.6, 11.1 Hz), 5.90 (dd, 1H, *J* = 4.1, 4.1 Hz), 5.23 (d, 1H, *J* = 17.6 Hz), 5.02 (d, 1H, *J* = 11.1 Hz), 4.10 (dd, 1H, *J* = 6.3, 4.9 Hz), 3.68 (s, 3H), 3.33 (s, 3H), 2.69-2.53 (m, 2H), 2.32-2.22 (m, 2H), 2.13-2.05 (m, 1H).

Ether alkene triester **31**

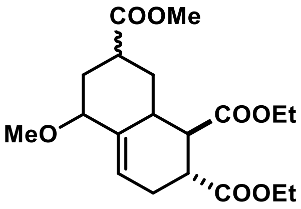

To a solution of diene **29** (0.023 g, 0.12 mmol, calculated assuming the impurity mentioned in the previous step had an identical molecular weight) in toluene (3 mL) was added diethyl fumarate (0.040 g, 0.039 mL, 2.3 mmol). The solution was degassed before being heated to reflux overnight. At completion, the mixture was cooled to room temperature, before the solvent was removed in vacuo. The crude mixture was subjected to silica gel column chromatography to afford the title compound (0.014 g, 32%) as an opaque semi-solid as a mixture of diastereomers.

^{1}H NMR (400 MHz, $CDCl_3$) δ 5.65-5.60 (m, 1H), 4.34-4.07 (m, 4H), 3.71-3.61 (m, 4H), 3.12-3.08 (m, 3H), 2.91-2.70 (m, 2H), 2.68-2.56 (m, 2H), 2.55-2.33 (m, 3H), 2.21-1.92 (m, 2H), 1.76-1.66 (m, 1H), 1.36-1.20 (m, 6H). ^{13}C NMR (101 MHz, $CDCl_3$) δ 175.5, 175.1, 175.0, 174.5, 174.3, 173.5, 139.9, 137.0, 123.3, 120.6, 81.2, 80.7, 60.81, 60.76, 60.7, 55.8, 55.4, 51.7, 51.6, 49.6, 45.3, 42.0, 37.9, 36.9, 36.2, 34.7, 33.5, 33.0, 32.7, 30.29, 30.25, 28.8, 28.0, 14.44, 14.42, 14.28, 14.27. HRMS (ESI-MS) calculated for **31** $C_{19}H_{29}O_7^+$ $[M + H]^+$: 369.1908; found: 369.1901. Additionally, calculated for $C_{19}H_{29}O_7^+$ $[M - OMe]^+$: 337.1646; found: 337.1641.

Ether alkane triester **32**, *alkane triester* **33**

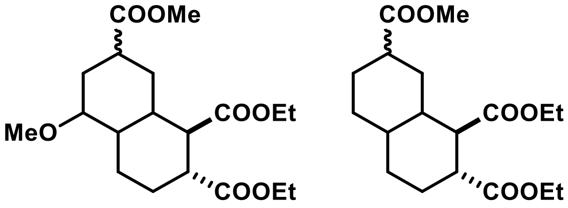

To a solution of alkene **31** (0.016 g, 0.043 mmol) in 4:1 methanol:AcOH (2 mL) was added PtO_2 (7 mg). The mixture was pump purged with hydrogen gas (10 bar), heated to 50 °C, and stirred for 2 hours, before being filtered through Celite using methanol to afford the title compound contaminated with the de-methoxylated compound **33** (17 mg total).

HRMS (ESI-MS) calculated for **32** $C_{19}H_{31}O_7^+$ $[M + H]^+$: 371.2064; found: 371.2060.

Ester **37**

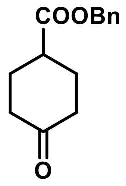

The synthesis of ester **37** followed the modified procedure of Wang et al. (2024).[3] To a stirred solution of triethylamine (8.0 g, 79 mmol, 11.0 mL) in dry DCM (120 mL) was added benzyl alcohol (7.0 g, 64.7 mmol, 6.7 mL), DMAP (1.305 g, 12.9 mmol, 0.2 equiv), 4-oxocyclohexane carboxylic acid (10.092 g, 70.1 mmol, 1.1 equiv.) and EDC hydrochloride (13.265 g, 69.2 mmol) while the solution was stirring. The mixture was stirred at room temperature overnight, before the solution was diluted into DCM, and washed with sat. aq. NH_4Cl. The aqueous phase was extracted with DCM (x2), before the organic portions were combined and washed with distilled water (x1), brine (x2), dried over Na_2SO_4 and concentrated in vacuo. The crude mixture was purified by silica gel column chromatography (1:20 to 1:1, EtOAc: toluene) to afford the title compound (7.27 g, 48%) as a clear oil. A further 3 g of ester **37** was obtained containing approximately 15% benzyl alcohol.

^{1}H NMR (400 MHz, $CDCl_3$) δ 7.40-7.31 (m, 5H), 5.16 (s, 2H), 2.85-2.77 (m, 1H), 2.51-2.43 (m, 2H), 2.39-2.30 (m, 2H), 2.27-2.19 (m, 2H), 2.10-1.99 (m, 2H). ^{13}C NMR (101 MHz, $CDCl_3$) δ 210.1, 174.1, 135.9, 129.2, 128.8, 128.5, 128.4, 128.3, 125.4, 66.7, 40.8, 39.8, 28.6, 21.6. HRMS (ESI-MS) calculated for **37** $C_{14}H_{17}O_3^+$ $[M + H]^+$: 233.1172; found: 223.1169.

Triflate **38**

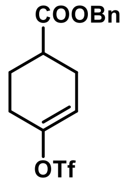

To a stirred solution of ester **37** (1.044 g, 4.49 mmol) and *N*-(5-chloropyridin-2-yl)-N-(methanesulfonyl)methanesulfonamide (Comin's reagent, 1.959 g, 4.99 mmol) in dry THF (20 mL) at -78°C was added NaHMDS (4.9 mL, 4.9 mmol) dropwise. The mixture stirred at -78 °C for 1 hr, before being warmed to room temperature and stirred overnight. The reaction was quenched with saturated aqueous NH_4Cl solution (20 mL), before the solvent was removed *in vacuo*. Et_2O (30 mL) and aqueous 2M NaOH (10 mL) were added, and the mixture was stirred vigorously for 15 min. The organic phase was set aside, and the aqueous phase extracted with Et_2O (x2). The organic fractions were combined and washed with distilled water (x1) and brine (x1), before being dried over Na_2SO_4, filtered, and concentrated *in vacuo*. The crude mixture was purified with silica gel column chromatography (1:9 to 1:4, EtOAc: PET), to afford the title compound (1.07 g, 65%) as a slightly yellow oil.

^{1}H NMR (400 MHz, $CDCl_3$) δ 7.42-7.30 (m, 5H), 5.80-5.74 (m, 1H), 5.15 (s 2H), 2.70-2.63 (m, 1H), 2.53-2.46 (m, 2H), 2.45-2.37 (m, 2H), 2.22-2.11 (m, 1H), 2.00-1.88 (m, 1H). ^{13}C NMR (101 MHz, $CDCl_3$) δ 173.9, 148.5, 135.9, 128.8, 128.5, 128.4, 120.2, 117.0, 66.8, 38.0, 26.7, 26.2, 25.1. HRMS (ESI-MS) calculated for **38** $C_{15}H_{16}F_3SO_3^+$ $[M + H]^+$: 365.0665; not found.

Diene **39**

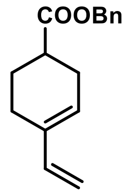

A stirred solution of compound **38** (4.92 g, 13.5 mmol), dppf (0.375 g, 0.688 mmol), $Pd(OAc)_2$ (0.156 g, 0.699 mmol), and K_3PO_4 (6.96 g, 32.8 mmol) in dry dioxane (40 mL) was degassed with nitrogen, before vinyl boronic acid pinacol ester (3.2 mL, 3.5 g, 23 mmol) was added. The reaction was heated to 100°C for 22 hr, before being cooled to room temperature, diluted with Et_2O and washed with water (x1). The aqueous layer was extracted with Et_2O (x3), before the organic layers were combined and washed with distilled water (x1) and brine (x1), then dried over Na_2SO_4 before the solvent was reduced *in vacuo.* The crude mixture was purified with silica gel column chromatograph (0:1 to 1:9, Et_2O: petroleum ether), to afford the title compound (1.58 g, 48%) as a clear liquid.

^{1}H NMR (400 MHz, $CDCl_3$) δ 7.41-7.29 (m, 5H), 6.34 (dd, 1H, *J* = 17.5, 10.8 Hz), 5.77-5.69 (dd, 1H, *J* = 4.2, 4.2 Hz), 5.14 (s, 2H), 5.07 (d, 1H, *J* = 17.6 Hz), 4.94 (d, 1H, *J* = 10.9 Hz), 2.65-2.58 (m, 1H), 2.49-2.44 (m, 2H), 2.37-2.26 (m, 1H), 2.22-2.09 (m, 2H), 1.81-1.71 (m, 1H). ^{13}C NMR (101 MHz, $CDCl_3$) δ 175.6, 139.5, 136.3, 135.8, 128.7, 128.3, 128.2, 127.4, 110.8, 77.4, 66.3, 39.6, 28.2, 25.1, 24.9, 23.3. HRMS (ESI-MS) calculated for **39** $C_{16}H_{19}O_2^+$ $[M + H]^+$: 243.1380, found; 243.1381.

Alkenes **41a** and **41b**

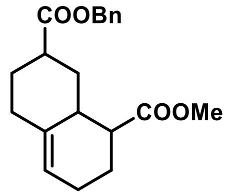

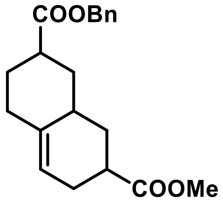

A solution of diene **39** (0.551 g, 2.27 mmol) in toluene (5 mL) in a sealed pressure vessel was degassed with nitrogen, and methyl acrylate (0.57g, 0.60 mL, 6.6 mmol) was added, before the solution was heated to 160°C for 22 hr. At completion, it was cooled to room temperature, and the solvent was removed *in vacuo.* The crude mixture was subjected to silica gel column chromatography (0:1 to 1:9, EtOAc: petroleum ether), to afford the title compound (0.229 g, 30%) as a clear liquid.

^{1}H NMR (400 MHz, $CDCl_3$) δ 7.39-7.28 (m, 5H), 5.47-5.34 (m, 1H), 5.21-5.08 (m, 2H), 3.71-3.53 (m, 3H), 2.88-2.66 (m, 1H), 2.65-2.36 (m, 2H), 2.34-2.07 (m, 4H), 2.06-1.78 (m, 3H), 1.78-1.38 (m, 3H), 1.26-1.11 (m, 1H). ^{13}C NMR (101 MHz, $CDCl_3$) δ 176.4, 176.3, 176.2, 176.1, 175.3, 175.13, 175.10, 174.92, 174.87, 174.8, 174.7, 174.6, 174.3, 140.2, 139.8, 139.2, 138.9, 138.0, 136.9, 136.8, 136.34, 136.32, 136.29, 136.26, 136.2, 128.7, 128.63, 128.61, 128.24, 128.19, 128.17, 128.11, 128.09, 128.06, 120.3, 119.6, 118.8, 118.3, 117.2, 66.4, 66.34, 66.28, 66.24, 66.21, 66.20, 51.8, 51.73, 51.69, 51.66, 51.5, 51.3, 48.0, 47.6, 43.84, 43.80, 43.5, 43.1, 40.0, 39.52, 39.45, 39.44, 39.41, 38.3, 38.1, 36.6, 36.0, 35.8, 35.6, 35.4, 35.3, 35.1, 34.18, 34.16, 34.0, 33.6, 33.1, 32.4, 32.3, 32.2, 31.8, 31.6, 31.2, 31.1, 31.0, 30.0, 29.7, 29.2, 28.4, 28.22, 28.21, 28.17, 27.5, 25.9, 25.7, 25.1, 24.87, 24.85, 24.7, 24.6, 24.5, 23.2, 20.32, 20.29. HRMS (ESI-MS) calculated for **41** $C_{20}H_{25}O_4^+$ $[M + H]^+$: 329.1747, found; 329.1741.

Alkenes **43a** and **43b**

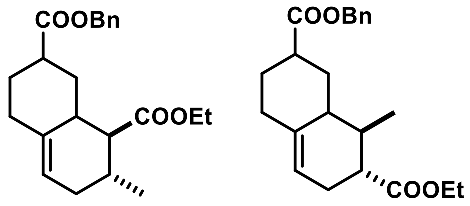

A stirred solution of diene **39** (0.368 g, 1.52 mmol) in toluene (3 mL) in a sealed pressure vessel was degassed with nitrogen, before ethyl crotonate (0.918 g, 8.04 mmol, 1.00 mL) was added. The reaction was stirred at 180°C for 22 hr, before it was cooled to room temperature, and the solvent was removed *in vacuo*. The crude mixture was subjected to silica gel column chromatography (1:49 to 1:9, EtOAc: petroleum ether), to afford the title compound (0.386 g, 71%) as a clear liquid.

^{1}H NMR (400 MHz, CDCl3) δ 7.41-7.27 (m, 5H), 5.43-5.33 (m, 1H), 5.27-5.05 (m, 2H), 4.22-4.01 (m, 2H), 2.90-2.76 (m, 1H), 2.59-2.36 (m, 2H), 2.33-1.97 (m, 5H), 1.94-1.76 (m, 2H), 1.75-1.59 (m, 1H), 1.53-1.36 (m, 1H), 1.31-1.07 (m, 3H), 1.01-0.85 (m, 3H). ^{13}C NMR (101 MHz, $CDCl_3$) δ 176.0, 175.6, 175.1, 174.9, 174.8, 174.7, 174.2, 173.8, 173.7, 139.3, 139.0, 138.2, 137.5, 136.6, 136.33, 136.30, 136.25, 136.2, 128.62, 128.61, 128.3, 128.23, 128.22, 128.20, 128.19, 128.17, 128.14, 128.09, 128.07, 128.05, 120.1, 119.4, 119.0, 118.7, 118.1, 117.8, 117.3, 116.3, 66.4, 66.3, 66.22, 66.18, 65.6, 60.33, 60.25, 60.1, 60.04, 59.97, 56.1, 55.5, 53.6, 51.3, 50.9, 47.34, 47.26, 44.0, 43.4, 43.0, 40.7, 40.1, 40.0, 39.9, 39.8, 39.4, 38.7, 38.4, 37.8, 37.6, 37.5, 36.0, 35.2, 34.9, 33.9, 33.8, 33.74, 33.71, 33.6, 33.4, 33.3, 33.1, 32.99, 32.95, 31.8, 31.6, 31.5, 31.3, 31.1, 30.9, 29.9, 29.8, 29.7, 29.6, 28.8, 28.2, 28.0, 26.0, 25.8, 24.8, 19.98, 19.95, 19.53, 19.47, 17.7, 17.5, 14.5, 14.43, 14.41, 14.39. HRMS (ESI-MS) calculated for **43** $C_{22}H_{29}O_4^+$ $[M + H]^+$: calculated; 357.2060, found; 357.2053.

Alkanes **44a** and **44b**

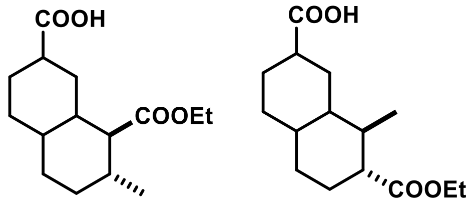

To a solution of alkenes **43a** and **43b** (0.062 g, 0.17 mmol) in EtOAc was added PtO_2 (0.016 g, 0.07 mmol) and three drops of acetic acid. The solution was pump purged with hydrogen gas, before being heated to 50 °C under hydrogen at 10 atmospheres of pressure and stirred for 16 hr. At completion, the mixture was filtered through celite with MeOH before the solvent was removed *in vacuo* to afford a white solid which was immediately subjected to the next reaction.

Diols **45a** and **45b**

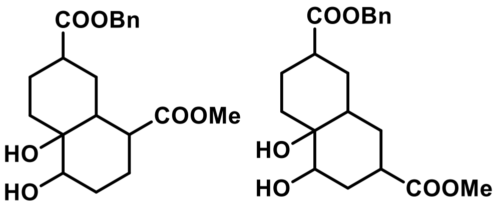

To a solution of compounds **41a** and **41b** (0.17 g, 0.52 mmol) in *t*BuOH (10 mL) and distilled water (10 mL) was added potassium osmate dihydrate (0.022 g, 0.059 mmol), potassium ferricyanide (0.347 g, 1.08 mmol) and K_2CO_3 (0.174 g, 1.26 mmol), before he solution was stirred at room temperature for 16 hr. At completion, the mixture was diluted into EtOAc, and the aqueous portion set aside. The organic phase was washed with distilled water (x2) followed by brine (10 mL), before being dried over Na_2SO_4, filtered and the solvent removed *in vacuo*. The crude mixture was subjected to silica gel column chromatography (1:9 to 4:1, EtOAc: petroleum ether), to afford the title compound (0.063 g, 33%) as a clear liquid.

^{1}H NMR (400 MHz, $CDCl_3$) δ 7.40-7.28 (m, 5H), 5.20-5.07 (m, 2H), 3.93-3.45 (m, 4H), 3.15-3.04 (m, 1H), 2.81-2.52 (m, 1H), 2.51-2.15 (m, 3H), 1.90-1.50 (m, 9H), 1.42-1.22 (m, 1H). ^{13}C NMR (101 MHz, $CDCl_3$) δ 175.4, 175.11, 175.07, 174.1, 136.1, 136.0, 128.71, 128.67, 128.40, 128.38, 128.3, 128.2, 72.8, 72.5, 66.69, 66.66, 66.56, 66.5, 51.8, 51.5, 44.3, 43.0, 42.0, 40.9, 40.7, 38.7, 35.0, 32.4, 29.54, 29.49, 26.5, 26.1, 25.0, 24.2, 21.3, 21.2. HRMS (ESI-MS) calculated for **45** $C_{20}H_{27}O_6^+$ [M + H]$^+$: 363.1802, found; 363.1794.

Diols **46a** and **46b**

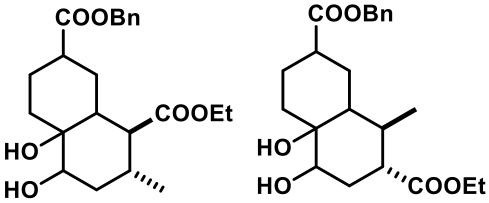

To a solution of alkenes **43a** and **43b** (0.087 mg, 0.24 mmol) in ^{t}BuOH (5 mL) and distilled water (5 mL) was added potassium osmate dihydrate (0.010 g, 0.027 mmol), potassium ferricyanide (0.145 g, 0.440 mmol) and K_2CO_3 (0.066 g, 0.48 mmol), before the solution was stirred at room temperature for 16 hr. At completion, the mixture was diluted into EtOAc, and the aqueous portion set aside. The organic phase was washed with distilled water (x2) followed by brine (x1), before being dried over Na_2SO_4, filtered and the solvent removed *in vacuo*. The crude mixture was subjected to silica gel column chromatography (1:19 to 3:2, EtOAc: petroleum ether), to afford the title compound (0.034 g, 36%) as a clear liquid.

^{1}H NMR (400 MHz, $CDCl_3$) δ 7.46-7.20 (m, 5H), 5.20-4.98 (m, 2H), 4.21-3.38 (m, 3H), 2.83-2.69 (m, 1H), 2.69-2.08 (m, 4H), 2.08-1.30 (m, 7H), 1.28-1.12 (m, 3H), 0.98-0.81 (m, 3H). ^{13}C NMR (126 MHz, 298K MeOD) δ 178.2, 177.6, 177.4, 177.3, 177.2, 177.02, 176.95, 176.7, 176.6, 176.2, 176.12, 176.11, 176.09, 175.8, 175.6, 137.8, 137.73, 137.72, 137.69, 129.59, 129.55, 129.53, 129.52, 129.3, 129.24, 129.17, 129.15, 129.11, 129.07, 129.0, 75.7, 75.6, 74.7, 74.5, 74.0, 73.7, 73.4, 73.2, 72.9, 72.6, 72.4, 72.2, 72.0, 71.6, 67.6, 67.5, 67.34, 67.28, 67.2, 67.10, 67.08, 66.9, 66.7, 66.5, 61.6, 61.5, 61.43, 61.39, 61.34, 61.31, 61.25, 54.8, 53.7, 53.1, 53.0, 52.9, 49.9, 49.7, 49.3, 49.1, 46.8, 46.4, 46.1, 45.9, 45.2, 44.3, 44.1, 44.0, 43.0, 42.3, 40.2, 40.1, 39.8, 38.9, 38.7, 38.63, 38.60, 38.5, 38.2, 38.3, 35.1, 34.8, 34.6, 34.4, 34.0, 33.9,

32.8, 32.6, 32.2, 31.8, 30.4, 30.3, 29.8, 28.9, 28.6, 28.5, 28.3, 28.1, 27.4, 26.6, 26.5, 25.1, 24.80, 24.75, 24.6, 24.2, 22.7, 22.0, 21.0, 20.9, 20.4, 20.3, 20.2, 20.0, 17.7, 16.3, 14.7, 14.64, 14.60, 14.56. HRMS (ESI-MS) calculated for **46** $C_{22}H_{31}O_6^+$ $[M + H]^+$: 391.2115, found; 391.2110.

Synthetic Procedures the Preparation of CRAM Analogues 5-16

Diacid **5**

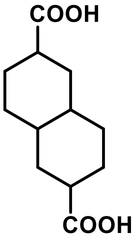

A solution of decahydro-2,6-napthalenedicarboxylic acid dimethyl ester (0.336 g, 1.32 mmol) in methanol (1 mL) and 6M aq. NaOH (9 mL), was heated to 50 °C and stirred overnight. At completion, the mixture was washed with Et_2O (x2), before being acidified to pH 2, and extracted with EtOAc (x3). The EtOAc portions were combined and reduced *in vacuo* to afford the title compound (0.297 g, 99%) as a 6:1 mixture of diastereomers.

^{1}H NMR *major* (400 MHz, MeOD) δ 2.39-2.22 (m, 2H), 1.94-1.60 (m, 10H), 1.60-1.38 (m, 4H). ^{1}H NMR *minor* (400 MHz, MeOD) δ 2.70-2.61 (m, 2H), 1.94-1.60 (m, 10H), 1.60-1.38 (m, 4H). ^{13}C NMR *major* (101 MHz, MeOD) δ 179.9, 45.1, 35.9, 32.3, 29.3, 24.6. ^{13}C NMR *minor* (101 MHz, MeOD) δ 180.1. 49.3, 40.5, 36.3, 29.1, 24.9. HRMS (ESI-MS) calculated for **5** $C_{12}H_{17}O_4^-$ [M - H]: calculated; 225.1132, found; 225.1132.

Diacids **6a** and **6b**, *diesters* **47a** and **47b**

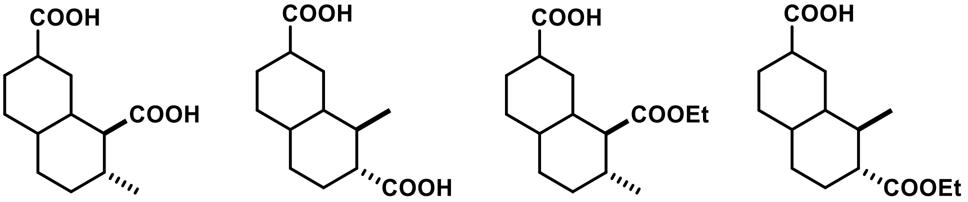

A solution of alkanes **44a** and **44b** (0.015 g) in methanol (0.5 mL) and 6M aq. NaOH (4 mL) was heated to 80 °C and stirred overnight. At completion, the mixture was washed with Et_2O (x2), before being acidified to pH 2, and extracted with EtOAc (x2). The EtOAc portions were combined and reduced *in vacuo* to afford the title compounds **6a** and **6b** contaminated with mono-ester compounds **47a** and **47b**.

Lactone **7**

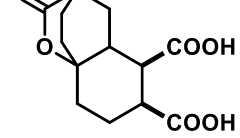

To a solution of ester **34** (0.431 g, 1.63 mmol mmol) in methanol (2 mL) was added 25% aqueous NaOH solution (8 mL), before being stirred overnight at room temperature. At completion, the mixture was acidified to pH 2, and extracted using a 5g Agilent Bond Elut PPL cartridge. The resulting solution was concentrated in vacuo, before the remaining liquid was frozen and freeze-dried. The resulting crude

mixture was subjected to preparative HPLC (H_2O:MeCN with 0.5% HCOOH, 100:0 to 60:40) to afford the title compound as a 9:1 mixture of diastereomers as a white powder (0.120 g, 27%).

^{1}H NMR (400 MHz, MeOD) δ 2.90-2.80 (m, 2H), 2.77-2.71 (m, 1H), 2.40-2.24 (m, 1H), 2.22-2.13 (m, 1H), 2.13-1.98 (m, 2H), 1.94-1.84 (m, 2H), 1.83-1.67 (m, 3H), 1.65-1.56 (m, 2H), 1.32-1.10 (m, 1H).^{13}C NMR (126 MHz, MeOD) δ 178.9, 178.7, 178.6, 177.7, 175.4, 175.3, 86.0, 85.5, 51.9, 51.7, 47.5, 44.9, 43.3, 43.2, 41.8, 38.6, 35.6, 35.3, 32.6, 30.4, 29.4, 28.4, 24.8, 23.11, 23.09, 22.8. HRMS (ESI-MS) calculated for **7** $C_{13}H_{15}O_6^-$ [M - H]$^-$: 267.0874; found: 267.0874. 100% purity by CAD.

Triacid alcohol **8**

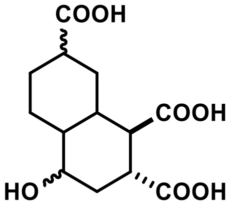

To a solution of alcohol **21** (0.051 g, 0.18 mmol) in methanol (1 mL) was added 25% aqueous NaOH solution (9 mL), before the mixture was heated to 60 °C and stirred overnight. At completion, the mixture was extracted with Et_2O (x2), before being acidified to pH 2, and extracted using a 5g Agilent Bond Elut PPL cartridge. The resulting solution was concentrated in vacuo, before the remaining liquid was frozen and freeze-dried to afford the title compound as a white powder (0.041 g, 100%).

^{1}H NMR (400 MHz, MeOD) δ 3.95-3.08 (m, 1H), 2.93-2.33 (m, 2H), 2.31-2.05 (m, 2H), 2.02-1.42 (m, 4H), 1.41-0.87 (m, 2H). ^{13}C NMR (101 MHz, MeOD) 179.6, 179.5, 178.7, 178.52, 178.45, 178.42, 178.39, 178.37, 178.36, 178.32, 178.29, 178.26, 178.22, 178.18, 178.12, 178.10, 178.03, 178.00, 177.98, 177.93, 177.90, 177.88, 177.8, 177.74, 177.69, 177.6, 177.53, 177.50, 177.3, 177.24, 177.18, 177.01, 177.00, 176.8, 176.7, 175.8, 175.7, 175.6, 73.6, 73.51, 73.46, 73.4, 73.3, 71.9, 71.7, 71.3, 70.9, 69.5, 69.2, 65.7, 65.52, 65.48, 53.3, 53.0, 52.64, 52.62, 52.55, 52.5, 52.4, 46.8, 46.03, 45.96, 45.6, 45.5, 44.8, 44.7, 44.5, 44.3, 44.18, 44.16, 44.1, 44.0, 43.94, 43.87, 43.1, 43.0, 42.5, 42.4, 41.9, 41.5, 41.4, 41.1, 40.8, 40.69, 40.65, 40.4, 40.34, 40.30, 40.2, 40.1, 40.01, 39.99, 39.8, 39.74, 39.68, 39.4, 39.22, 39.18, 39.1, 38.7, 38.5, 38.4, 38.21, 38.20, 38.16, 38.0, 37.2, 36.34, 36.28, 34.64, 34.55, 34.24, 34.20, 33.2, 32.8, 32.7, 32.6, 32.2, 31.9, 30.5, 30.3, 29.84, 29.81, 29.7, 29.4, 29.3, 29.1, 29.0, 28.9, 28.6, 27.9, 27.5, 27.4, 27.3, 27.10, 27.05, 26.44, 26.39, 26.3, 25.8, 25.7, 25.38, 25.36, 24.2, 23.6, 22.0, 19.67, 19.65. HRMS (ESI-MS) calculated for **8** $C_{13}H_{17}O_7^-$ [M - H]$^-$: 285.0980; found: 285.0978. 91% purity by CAD.

Tertiary alcohol **9**

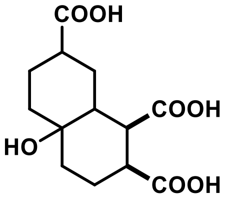

A solution of lactone **7** (0.025 g) in methanol (0.5 mL) and 6M aq. NaOH (4 mL), was heated to 50 °C and stirred overnight. At completion, the mixture was washed with Et_2O (x2), before being acidified to pH 3, and extracted with EtOAc (x3). The EtOAc portions were combined and reduced *in vacuo* to afford the title compound (0.024 mg, 88%).

^{1}H NMR (400 MHz, MeOD) δ 3.01 (dd, 1H, *J* = 5.0, 5.0 Hz), 2.78-2.72 (m, 1H), 2.64 (ddd, 1H, *J* = 12.8, 4.1, 4.1 Hz), 2.27-2.19 (m, 1H), 2.05-1.78 (m, 6H), 1.74-1.67 (m, 1H), 1.51-1.38 (m, 3H). ^{13}C NMR (101 MHz, MeOD) δ 179.1, 178.7, 177.1, 70.2, 47.3, 46.4, 42.0, 40.4, 40.3, 37.3, 28.3, 23.3, 21.1. HRMS (ESI-MS) calculated for **9** $C_{13}H_{17}O_7^-$ [M - H]$^-$: 285.0980; found: 285.0984. 100% purity by CAD.

Ether **10**

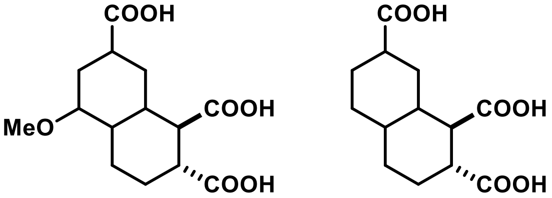

To a solution of methyl ether **32** and tri-ester **33** (0.016 g) in methanol (0.5 mL) was added 25% NaOH solution (2 mL). The mixture was heated to 60 °C and stirred overnight, before being cooled to room temperature and extracted with diethyl ether (x2). The aqueous portion was acidified to pH 2, and extracted with ethyl acetate (x2), before these organic portions were combined and dried in vacuo to afford the title compound **10** contaminated with triester **48**.

^{1}H NMR (500 MHz, $CDCl_3$) δ 4.22-3.19 (m, 4H), 2.91-2.51 (m, 2H), 2.50-1.90 (m, 3H), 1.90-1.36 (m, 5H), 1.34-0.81 (m, 3H). ^{13}C NMR (126 MHz, MeOD) δ 179.9, 179.5, 179.44, 179.41, 179.3, 178.71, 178.67, 178.6, 178.5, 178.44, 178.37, 178.34, 178.29, 178.27, 178.23, 178.20, 178.1, 178.0, 177.8, 177.7, 177.3, 176.3, 176.2, 81.7, 81.5, 81.3, 80.9, 80.8, 80.5, 80.4, 79.9, 79.82, 79.76, 79.7, 68.9, 61.61, 61.55, 57.4, 57.3, 57.2, 56.6, 56.4, 53.5, 53.4, 53.2, 53.11, 53.09, 53.0, 52.9, 47.9, 47.70, 47.68, 47.65, 47.6, 47.5, 47.42, 47.39, 47.3, 47.2, 46.5, 46.4, 46.02, 45.97, 44.73, 44.68, 44.66, 44.6, 44.3, 43.6, 42.6, 42.5, 42.3, 42.2, 42.01, 41.94, 41.7, 41.5, 41.2, 41.0, 40.8, 40.5, 40.42, 40.36, 40.35, 40.1, 39.5, 39.1, 38.9, 38.6, 38.5, 38.41, 38.35, 38.3, 38.20, 38.18, 38.1, 37.6, 37.22, 37.19, 37.1, 36.43, 36.39, 35.3, 35.1, 34.4, 34.30, 34.25, 33.9, 33.8, 33.1, 33.0, 32.92, 32.86, 32.0, 31.9, 31.82, 31.80, 31.7, 31.1, 31.0, 30.9, 30.8, 30.61, 30.57, 30.5, 30.3, 30.13, 30.09, 29.87, 29.85, 29.79, 29.77, 29.7, 29.6, 29.4, 29.3, 29.12, 29.10, 29.0, 28.3, 27.9, 27.8, 27.7, 26.5, 26.33, 26.25, 26.1, 25.7, 25.5, 25.2, 24.91, 24.87, 24.8, 24.7, 24.1, 22.6, 14.52, 14.49, 14.45. HRMS (ESI-MS) calculated for **10** $C_{14}H_{19}O_7^-$ [M - H]$^-$: 299.1136; found: 299.1144. 61% purity of **10** by CAD

Triacid ketone **11a** (from single diastereomer)

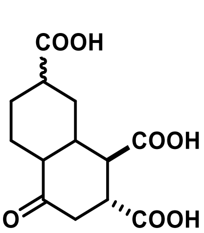

To a solution of ketone triester **22** single diastereomer (0.014 g, 0.04 mmol) in methanol (0.5 mL) was added 25% aqueous NaOH solution (2 mL), before being heated to 50 °C and stirred overnight. At completion, the mixture was acidified to pH 2, and extracted using a 1g Agilent Bond Elut PPL cartridge. The resulting solution was concentrated in vacuo, before the remaining liquid was frozen and freeze-dried to afford the title compound as an off-white powder (0.09 g, 80%).

^{1}H NMR (500 MHz, MeOD) δ 3.24-3.04 (m, 1H), 3.03-2.47 (m, 4H), 2.45-2.21 (m, 1H), 2.17-1.79 (m, 3H), 1.79-1.09 (m, 4H). ^{13}C NMR (126 MHz, MeOD) δ 212.0, 210.3, 178.0, 177.9, 176.8, 176.0, 49.4, 49.3, 44.1, 43.5, 41.9, 40.1, 40.0, 39.9, 38.1, 33.1, 32.5, 28.9, 27.3, 25.8, 24.9, 23.4, 23.2, 22.4. HRMS (ESI-MS) calculated for **11** $C_{13}H_{15}O_7^-$ [M - H]$^-$: 283.0823; found: 283.0832. 64% purity by CAD.

Triacid ketone **11b** (mixture of diastereomers)

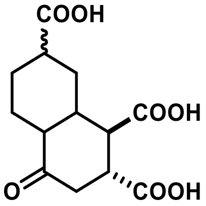

To a solution of ketone **22** mixture of diastereomers (0.078 g, 0.22 mmol) in methanol (1 mL) was added 25% aqueous NaOH solution (4 mL), before being stirred overnight at room temperature. At completion, the mixture was extracted with Et_2O (x2) before being acidified to pH 2, and extracted using a 5g Agilent Bond Elut PPL cartridge. The resulting solution was concentrated in vacuo, before the remaining liquid was frozen and freeze-dried to afford the title compound as an off-white powder (0.061 g, 97%).

^{1}H NMR (400 MHz, MeOD) δ 3.22-2.87 (m, 1H), 2.86-2.51 (m, 3H), 2.50-1.89 (m, 4H), 1.87-1.57 (m, 2H), 1.56-1.20 (m, 3H)^{13}C NMR (101 MHz, MeOD) 210.2, 209.8, 178.8, 177.8, 176.83, 176.75, 175.6, 175.5, 53.0, 52.6, 52.2, 52.0, 47.1, 44.7, 43.5, 43.4, 43.1, 42.0, 39.7, 35.0, 34.9, 33.4, 28.7, 28.6, 26.8, 25.4, 22.7, 22.6. HRMS (ESI-MS) calculated for **11** $C_{13}H_{15}O_7^-$ [M - H]$^-$: 283.0823; found: 283.0829. 75% purity by CAD.

Acyloin **12**

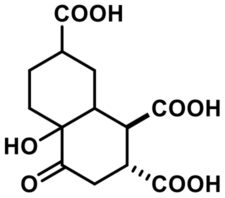

A solution of acyloin ester **19** (0.045 g) in methanol (0.5 mL) and 6M aq. NaOH (4 mL), was heated to 50 °C and stirred overnight. At completion, the mixture was washed with Et_2O (x2), before being acidified to pH 2, and extracted with EtOAc (x2). The EtOAc portions were combined and reduced *in vacuo* to afford the title compound (0.034 g, 93%). Attempting to acquire NMR spectra other than ^{1}H NMR data led to degradation of the compound. Reported first is the ^{1}H NMR spectra of the main isomer of title compound **19**, followed by the ^{1}H NMR and ^{13}C NMR data of the degradation compound, which was unassigned.

^{1}H NMR (400 MHz, MeOD) δ 3.79 (dd, 1H, *J* = 11.9, 4.4 Hz), 3.09 (ddd, 1H, *J* = 13.8, 12.1, 4.5 Hz), 2.98 (dd, 1H, *J* = 13.4, 13.4 Hz), 2.71-2.65 (m, 1H), 2.56 (ddd, 1H, *J* = 13.9, 3.8, 3.8 Hz), 2.42 (dd, 1H, *J* = 13.3, 4.4 Hz), 2.15-1.96 (m, 3H), 1.67 (dddd, 1H, *J* = 13.6, 13.6, 4.9, 3.8 Hz), 1.47 (ddd, 1H, *J* = 13.4, 13.4. 4.4 Hz), 1.29 (ddd, 1H, *J* = 13.8, 12.7, 4.8 Hz). HRMS (ESI-MS) calculated for **12** $C_{13}H_{15}O_8^-$ [M - H]$^-$: 299.0772; found: 299.0780. 71% purity by CAD.

Triacid diol **13**

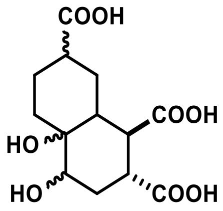

To a solution of diol **18** (0.052 g, 0.14 mmol) in methanol (1 mL) was added 25% aqueous NaOH solution (4 mL), before being stirred overnight at room temperature. At completion, the mixture was acidified to pH 2, and extracted using a 5g Agilent Bond Elut PPL cartridge. The resulting solution was concentrated in vacuo, before the remaining liquid was frozen and freeze-dried. The resulting crude mixture was subjected to preparative HPLC (H_2O:MeCN with 0.5% HCOOH, 100:0 to 80:20), from which three peaks were collected to afford three separate fractions (f1 = 2.3 mg, 5%, single diastereomer by NMR, f2 = 0.9 mg, 2%, insufficient mass for NMR acquisition; f3 = 13 mg, 31%, 4:1 ratio of two diastereomers).

f1 ^{1}H NMR (400 MHz, MeOD) δ 3.94 (dd, 1H, *J* = 11.5, 4.8 Hz), 3.37 (dd, 1H, *J* = 12.1, 4.8 Hz), 2.81 (ddd, 1H, *J* = 9.2, 8.3, 4.0 Hz), 2.42-2.29 (m, 2H), 2.20 (ddd, 1H, *J* = 13.6, 13.6, 4.5 Hz), 2.01 (ddd, 1H, *J* = 12.1, 12.1, 4.3 Hz), 1.94-1.77 (m, 2H), 1.69-1.45 (m, 3H), 1.31 (ddd, 1H, *J* = 13.6, 13.6, 4.1 Hz). f1 ^{13}C NMR (101 MHz, MeOD) δ 178.9, 178.5, 177.7, 72.8, 66.8, 45.7, 44.3, 44.2, 40.6, 35.2, 33.5, 28.6, 26.9. f3 *major* ^{1}H NMR (400 MHz, MeOD) δ 3.97 (dd, 1H, *J* = 11.5, 4.8 Hz), 3.35 (dd, 1H, *J* = 12.1, 4.8 Hz), 2.86-2.78 (m, 1H), 2.75-2.62 (m, 1H), 2.53-2.27 (m, 1H), 2.20-1.98 (m, 3H), 1.92-1.76 (m, 3H), 1.72-1.55 (m, 2H), 1.51-1.35 (m, 1H). f3 *minor* ^{1}H NMR (400 MHz, MeOD) δ 3.53 (dd, 1H, *J* = 2.3, 2.3 Hz), 3.16-3.09 (m, 1H), 2.86-2.78 (m, 1H), 2.75-2.62 (m, 1H), 2.53-2.27 (m, 1H), 2.20-1.98 (m, 3H), 1.92-1.76 (m, 2H), 1.72-1.55 (m, 2H), 1.51-1.35 (m, 1H). f3 ^{13}C NMR (101 MHz, MeOD) 178.7, 178.0, 73.7, 73.1, 72.1, 71.5, 66.6, 64.7, 57.2, 54.8, 52.3, 46.1, 45.7, 44.3, 43.3, 43.1, 42.9, 40.6, 40.5, 39.8, 39.1, 33.7, 33.5, 33.2, 33.0, 32.6, 30.4, 27.2, 26.8, 25.3, 24.8, 23.4, 23.1, 22.4, 22.2, 22.0. HRMS (ESI-MS) calculated for **13** $C_{13}H_{15}O_8^-$ [M - H]$^-$: 301.0929; found: 301.0929. CAD purities: f1 = 85%, f2 = 100%, f3 = 100%.

Diacids **14a** and **14b**

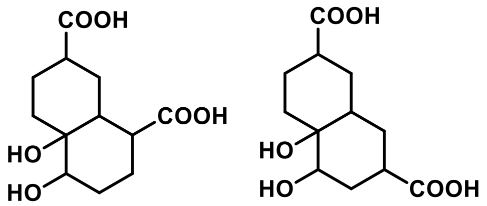

A solution of diols **45a** and **45b** (0.049 g, 0.14 mmol) in methanol (0.5 mL) and 6M aq. NaOH (4 mL), was heated to 60 °C and stirred overnight. At completion, the mixture was washed with Et_2O (x2), before being acidified to pH 2, and extracted with EtOAc (x3). The EtOAc portions were combined and reduced *in vacuo* to afford the title compounds **14a** and **14b** (0.005 g, 14%).

^{1}H NMR (500 MHz, MeOD) δ 3.92-3.01 (m, 1H), 2.76-2.26 (m, 2H), 2.22-1.99 (m, 2H), 1.97-1.55 (m, 7H), 1.54-1.14 (m, 2H). ^{13}C NMR (126 MHz, MeOD) δ 179.8, 179.2, 179.0, 178.8, 178.7, 178.2, 76.1, 76.0, 75.9, 73.7, 73.39, 73.38, 72.5, 72.3, 72.1, 68.9, 67.3, 67.0, 46.1, 45.5, 45.4, 44.1, 43.4, 42.1, 42.0, 41.9, 39.8, 39.7, 35.5, 32.8, 32.3, 29.94, 29.91, 29.7, 28.8, 27.8, 27.3, 27.0, 26.5, 25.9, 25.1, 24.9, 22.8, 22.6, 22.5. HRMS (ESI-MS) calculated for **14** $C_{12}H_{17}O_6^-$ [M - H]: calculated; 257.1031, found; 257.1031. 70% purity by CAD.

Diol diacids **15a** and **15b**, *esters* **16a** and **16b**

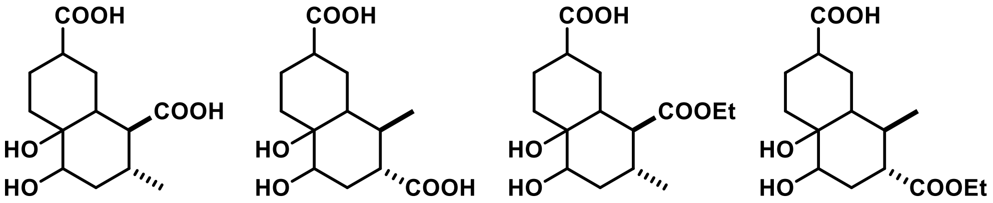

A solution of diols **46a** and **46b** (0.023 g) was dissolved in methanol (0.5 mL) and 6M aq. NaOH (4 mL), before being heated to 80 °C and stirred overnight. At completion, the mixture was washed with Et_2O (x2), before being acidified to pH 2, and extracted with EtOAc (x2). The EtOAc portions were combined and reduced *in vacuo* to afford the title compounds **15a** and **15b** contaminated with mono-ester compounds **16a** and **16b**.

Table S1: *%CI data for compounds* ***1-16*** *for both SRFA and TRM, including %CI values for every measured isomer, and mean value for all isomers of each compound.*

Compound Number	*SRFA %CI Isomers*	*SRFA %CI Average*	*TRM %CI Isomers*	*TRM %CI Isomers*
**1** alkene triacid	63, 72, 74, 78, 79, 80, 81	75	54, 65, 68, 74, 75, 76, 77	70
**2** alkane triacid	74, 74, 75, 78, 82	77	68, 69, 70, 73, 79	72
**3** alkene tetraacid	85, 92, 93	87	76, 87, 90	84
**4** alkane tetraacid	90, 90, 91, 92, 92, 93, 94	92	83, 83, 85, 87, 87, 90, 91	87
**5** diacid	45, 54	50	46, 58	52
**6** methyl diacid	39, 49, 52, 57	49	45, 56, 59, 63	56
**7** lactone	55, 57	56	45, 47	46
**8** sec alcohol	1, 1, 1, 2, 3, 14, 50, 62	17	1, 1, 1, 3, 4, 13, 43, 55	15
**9** tert alcohol	10, 18, 61	30	9, 16, 53	26
**10** ether	26, 39, 49, 56, 62, 65, 70, 79, 86	59	24, 35, 45, 51, 57, 61, 66, 76, 84	55
**11** ketone	43, 57, 67, 68	59	31, 43, 52, 54	45
**12** acyloin	4, 50	27	3, 34	19
**13** triacid diol	1, 1, 1	1	1, 1, 1	1
**14** diacid diol	1, 1, 1, 1, 7, 14	4	1, 1, 1, 1, 5, 12	4
**15** methyl diacid diol	1, 1, 1, 1, 1, 4, 17, 20	6	1, 1, 1, 1, 2, 4, 18, 22	6
**16** acid ester diol	19, 23	21	20, 24	22

Table S2: *HCD 35V average fragment mass, HCD 75V average fragment mass, and HCD 75V average fragment mass divided by parent ion mass, as well as the HCD 35V average fragment mass and HCD 75V average fragment mass for the same nominal mass in TRM. Final columns are the number of peaks observable in the isolated compounds that are also observable in TRM fragmentations above 1% intensity of the highest fragment mass at both 35V and 75V.*

Compound	HCD35 average	HCD75 average	HCD75 per parent mass	TRM HCD35 average	TRM HCD75 average	%HCD35 Overlap	%HCD75 Overlap
Triacid alkene **1**a	238.36	174.76	0.65	160.18	105.54	100	36
Triacid alkene **1**b	240.39	180.57	0.68	160.18	105.54	100	39
Triacid alkane **2**a	238.35	174.76	0.65	155.94	106.24	100	59
Triacid alkane **2**b	240.39	180.57	0.67	155.94	106.24	100	50
Tetraacid alkene **3**a	202.64	125.99	0.41	188.05	112.77	100	79
Tetraacid alkene **3**b	195.43	128.04	0.41	188.05	112.77	100	68
Tetraacid alkane **4**a	236.02	167.01	0.53	176.41	105.87	100	69
Tetraacid alkane **4**b	240.72	178.54	0.57	176.41	105.87	100	65
Diacid **5**	218.14	167.04	0.74	144.91	101.39	67	8
Methyl diacid **6**	220.18	167.37	0.70	155.42	103.13	75	47
Lactone **7**	205.54	128.75	0.48	184.31	102.70	100	58
Sec alcohol **8**	251.88	154.50	0.54	173.07	104.17	89	65
Tert Alcohol **9**	225.23	141.85	0.50	173.07	104.17	88	58
Ether **10**	250.11	154.20	0.52	182.54	105.34	86	50
Ketone f1 **11**	230.37	123.01	0.43	178.14	105.95	100	81
Ketone f2 **11**	236.69	126.86	0.45	178.14	105.95	100	81
Acyloin **12**	215.84	116.26	0.39	182.54	105.34	89	62
Triacid diol isomer 1 **13**	240.72	128.89	0.43	162.04	97.45	88	65
Triacid diol isomer 2 **13**	245.38	128.74	0.43	162.04	97.45	75	64
Triacid diol isomer 3 **13**	253.85	132.08	0.44	162.04	97.45	82	55
Diacid diol **14**	235.45	136.53	0.53	164.51	105.69	72	22
Methyl diacid diol **15**	243.03	130.52	0.48	167.93	108.57	82	48
Acid ester diol **16**	235.08	125.59	0.42	182.54	105.34	72	63

Comparisons between isolated compounds and TRM focused on two metrics; the weighted average fragment mass of all peaks in a given HCD spectrum (HCD35 and HCD75 average), and the number of peaks in isolated fragmentation spectra that were also observable in fragmentations of the same nominal mass within TRM (%HCD35 and %HCD75 overlap). For average fragment masses for HCD 75V fragmentations, these were also normalized for isolated compounds by their parent ion mass for all compounds (HCD75 per parent mass). This same metric was not generated for TRM, as all HCD 75V weighted average fragment masses were close to 100 m/z, and as such the number would only inversely represent the m/z of the parent mass.

For isolated compounds, HCD75 per parent mass generally decreased as %HCD75 overlap increased (Figure S1). Essentially, this shows that the more a compound breaks down, the more its fragments appear in TRM. It is important to note that some compounds with the best %HCD75 overlaps such as ketone **9** still bear strong functional group losses corresponding to the breakdown of their carboxylic acids, not observed in the comparable experiments with TRM. As such, these metrics are general indicators that should be checked against their actual fragmentation spectra (vide infra) for accurate analysis.

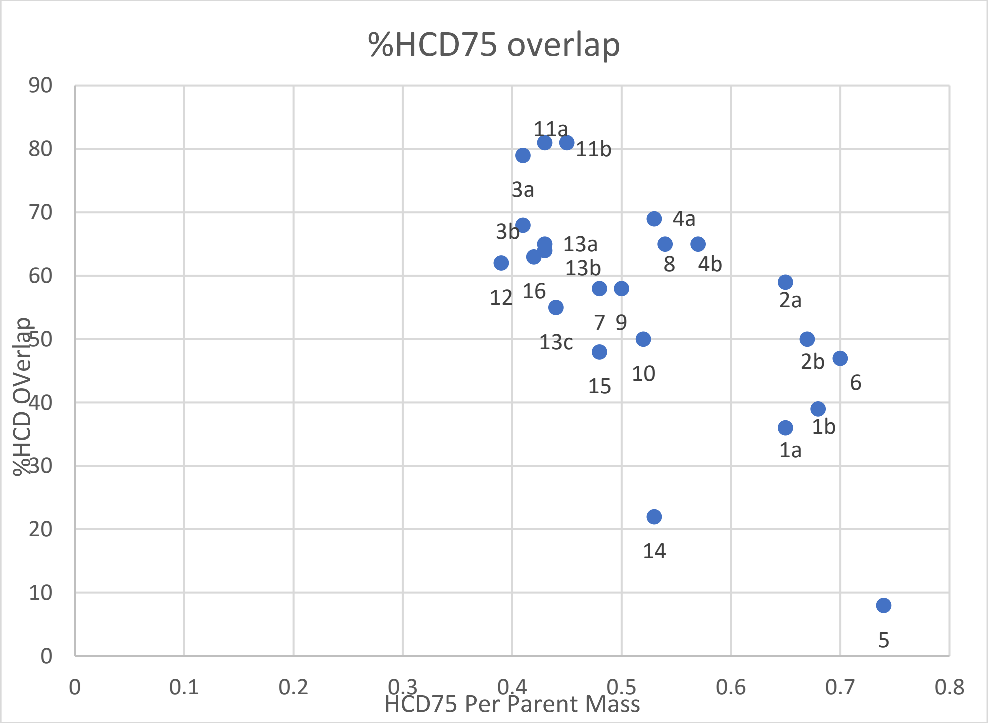

Figure S1: *HCD75 per parent mass vs %HCD75 overlap.*

Extracted Ion Chromatogram Data Used to Calculate % Cumulative Intensity

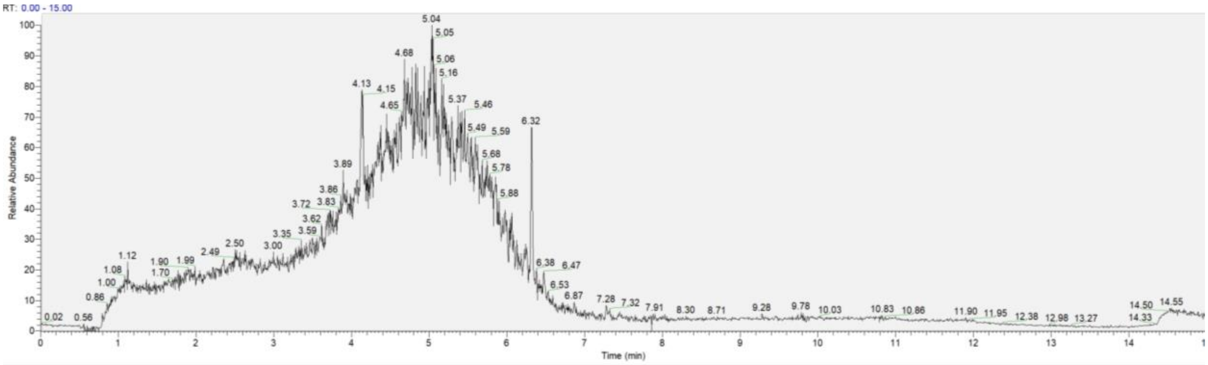

Figure S2: *XIC of TRM for formula $C_{13}H_{15}O_6^-$ (triacid alkene **1** and lactone **7** molecular formula).*

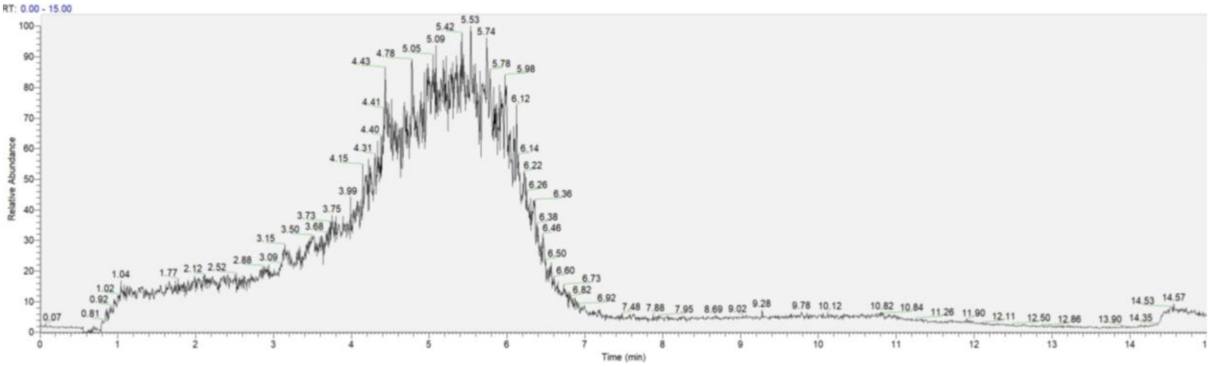

Figure S3: *XIC of TRM for formula $C_{13}H_{17}O_6^-$ (triacid alkane **2** molecular formula).*

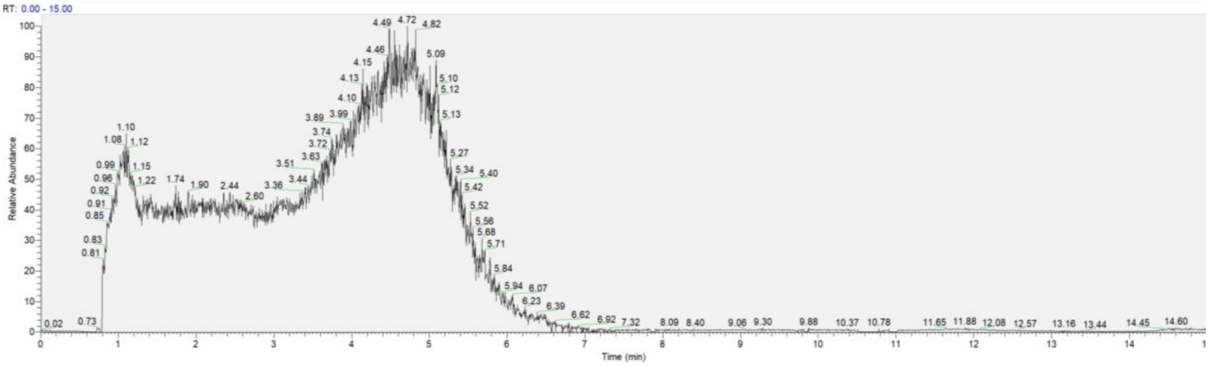

Figure S4: *XIC of TRM for formula $C_{14}H_{15}O_8^-$ (tetraacid alkene **3** molecular formula).*

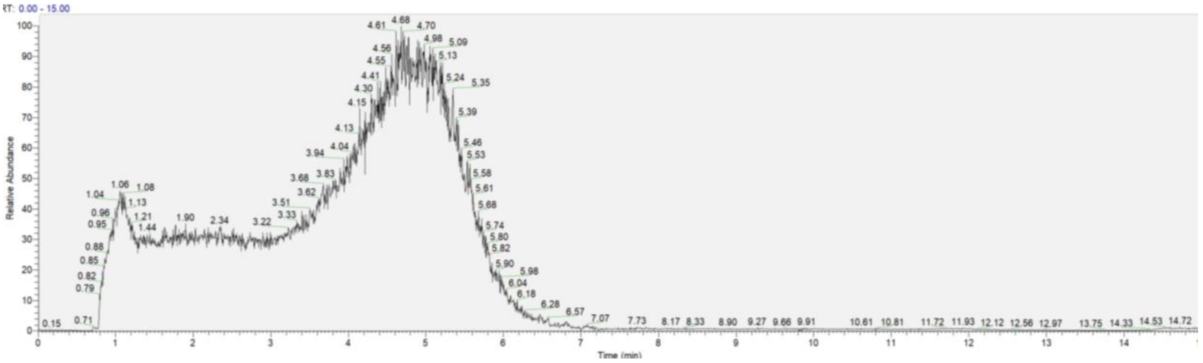

Figure S5: *XIC of TRM for formula $C_{14}H_{17}O_8^-$ (tetraacid alkane **4** molecular formula).*

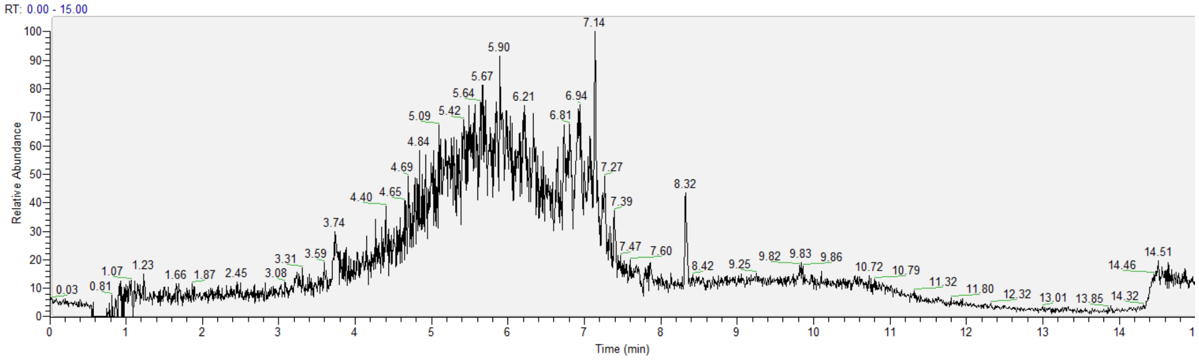

Figure S6: *XIC of TRM for formula $C_{12}H_{17}O_4^-$ (diacid **5** molecular formula).*

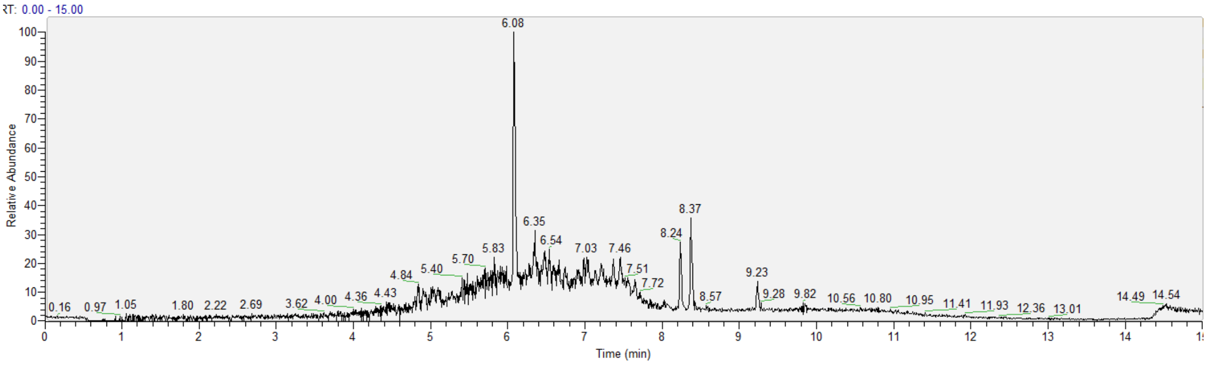

Figure S7: *XIC of TRM for formula $C_{13}H_{19}O_4^-$ (methyl diacid **6** molecular formula).*

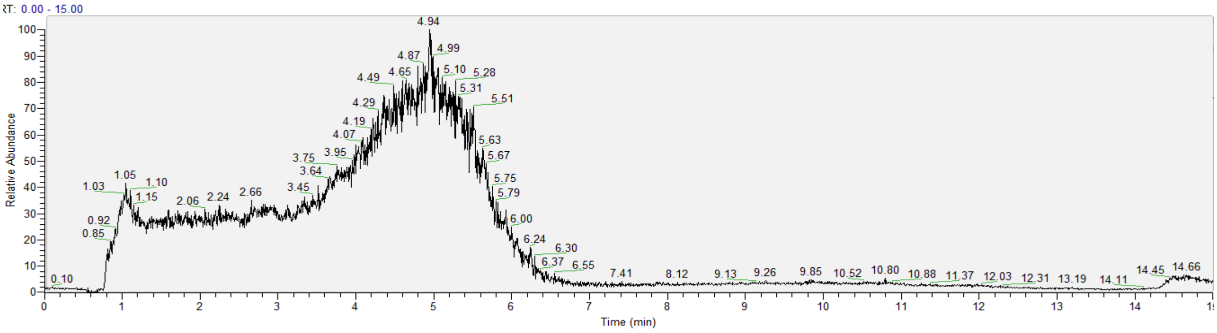

Figure S8: *XIC of TRM for formula $C_{13}H_{17}O_7^-$ (sec alcohol **8** and tert alcohol **9** molecular formula).*

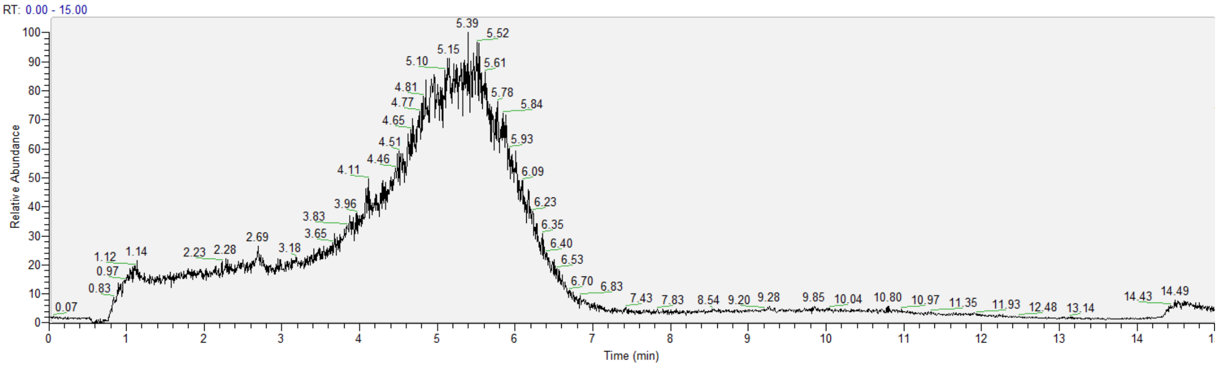

Figure S9: *XIC of TRM for formula $C_{14}H_{19}O_7^-$ (ether **10** molecular formula).*

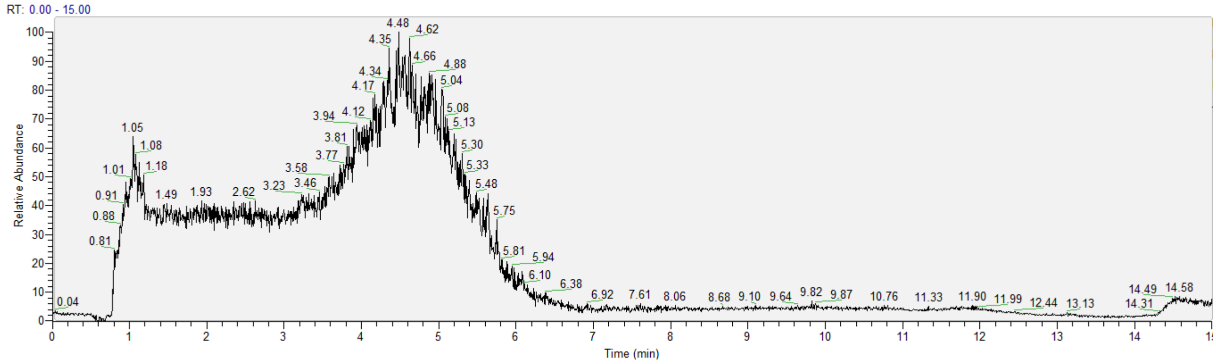

Figure S10: *XIC of TRM for formula* $C_{13}H_{15}O_7^-$ *(ketone* ***11*** *molecular formula).*

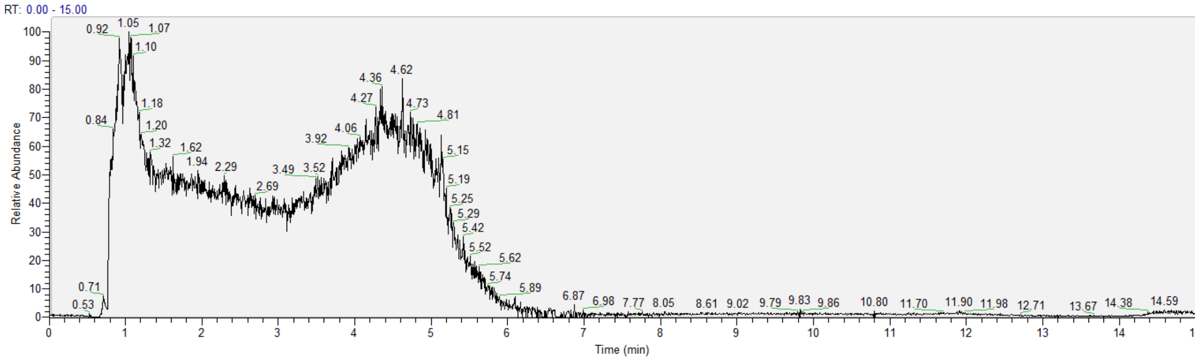

Figure S11: *XIC of TRM for formula* $C_{13}H_{15}O_8^-$ *(acyloin* ***12*** *molecular formula).*

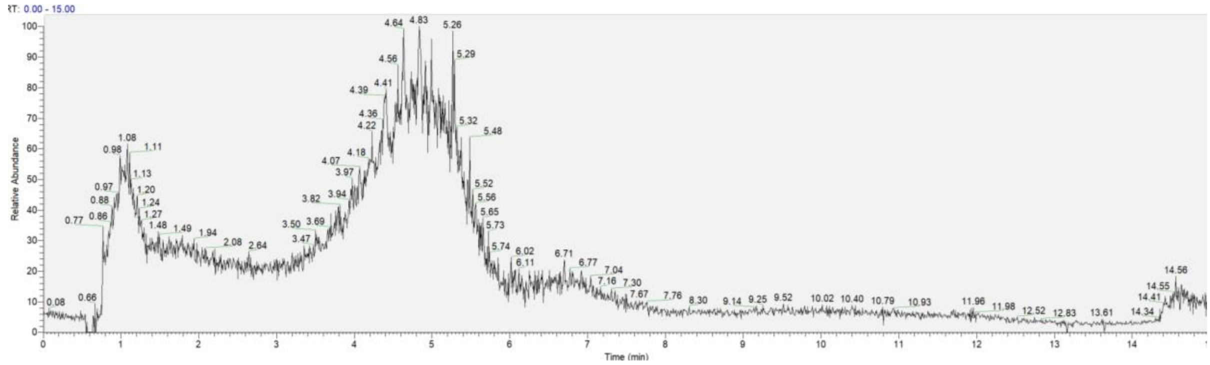

Figure S12: *XIC of TRM for formula* $C_{13}H_{17}O_8^-$ *(triacid diol* ***13*** *molecular formula).*

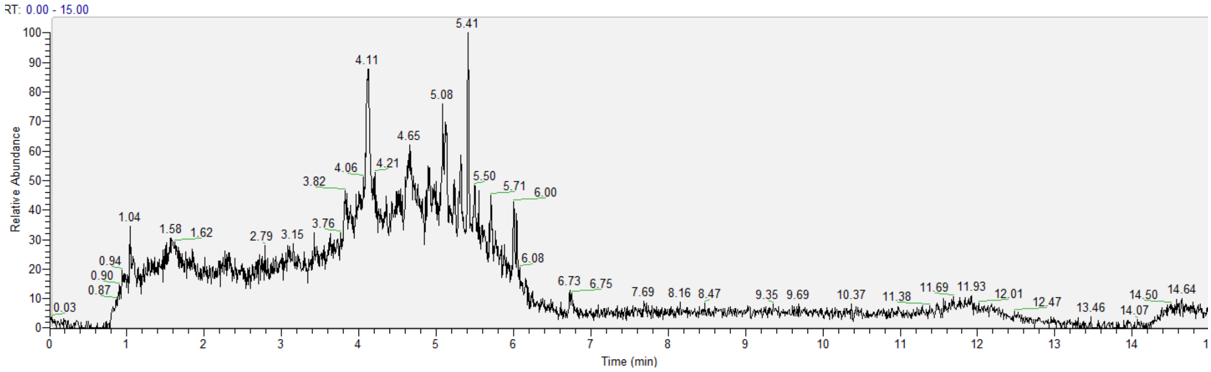

Figure S13: *XIC of TRM for formula* $C_{12}H_{17}O_6^-$ *(diacid diol* ***14*** *molecular formula).*

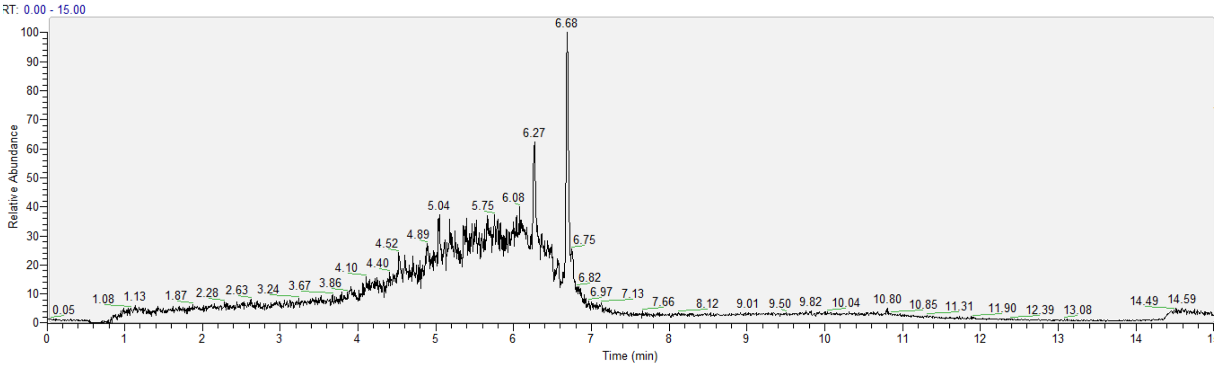

Figure S14: *XIC of TRM for formula $C_{13}H_{19}O_6^-$ (methyl diacid diol **15** molecular formula).*

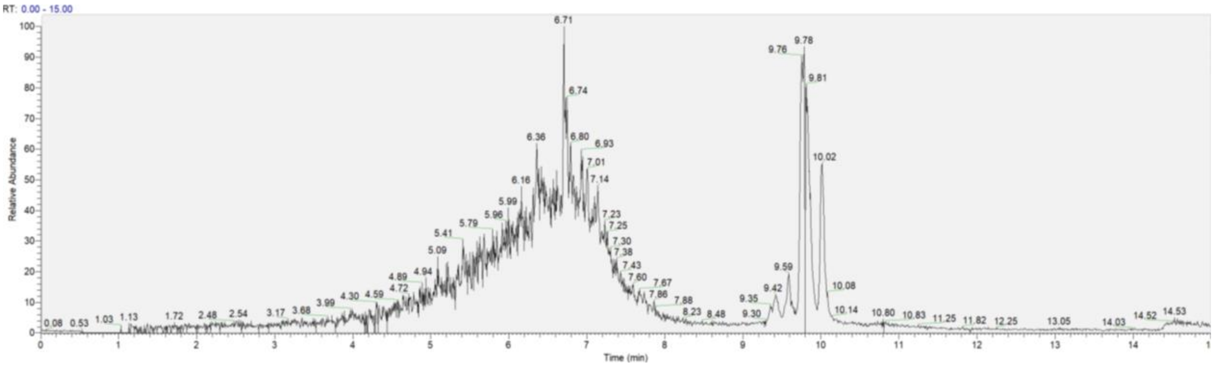

Figure S15: *XIC of TRM for formula $C_{15}H_{23}O_6^-$ (acid ester diol **16** molecular formula).*

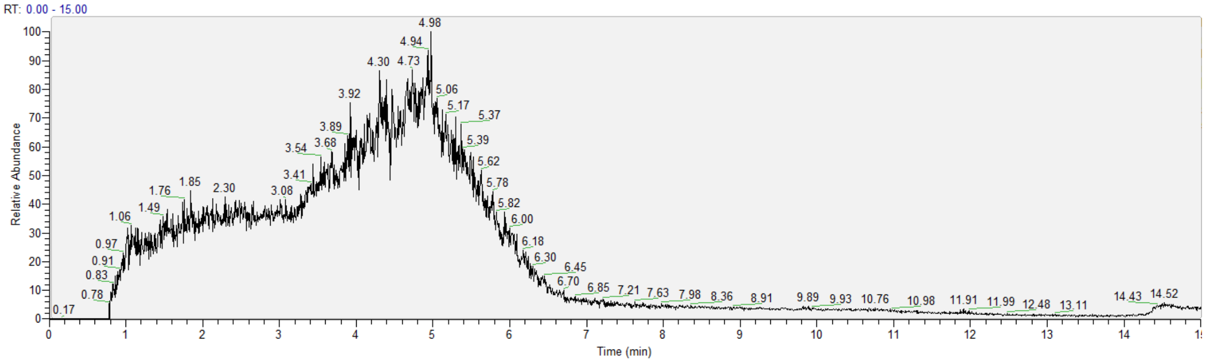

Figure S16: *XIC of SRFA for formula* $C_{13}H_{15}O_6^-$ *(triacid alkene* ***1*** *and lactone* ***7*** *molecular formula).*

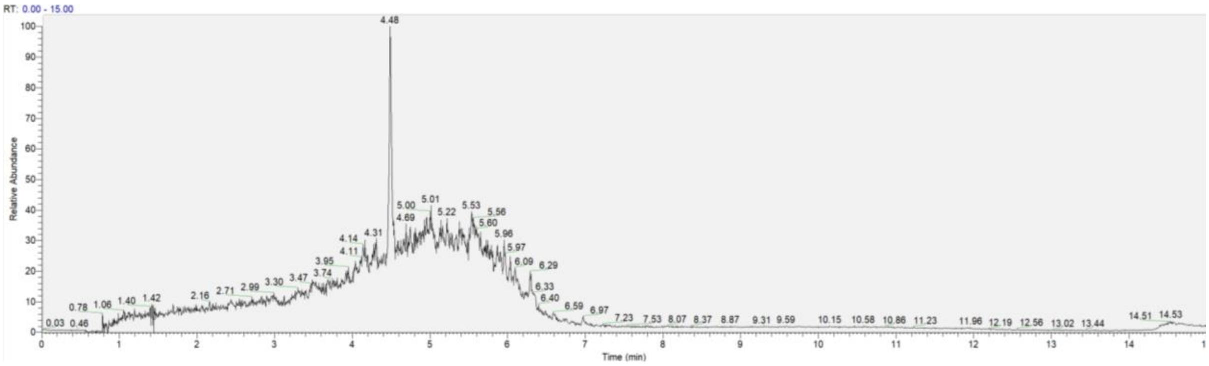

Figure S17: *XIC of SRFA for formula* $C_{13}H_{17}O_6^-$ *(triacid alkane* ***2*** *molecular formula).*

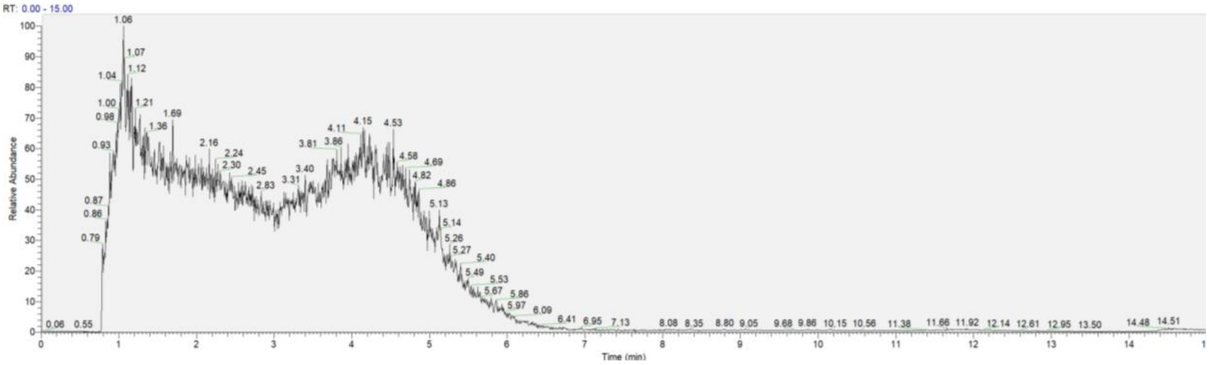

Figure S18: *XIC of SRFA for formula* $C_{14}H_{15}O_8^-$ *(tetraacid alkene* ***3*** *molecular formula).*

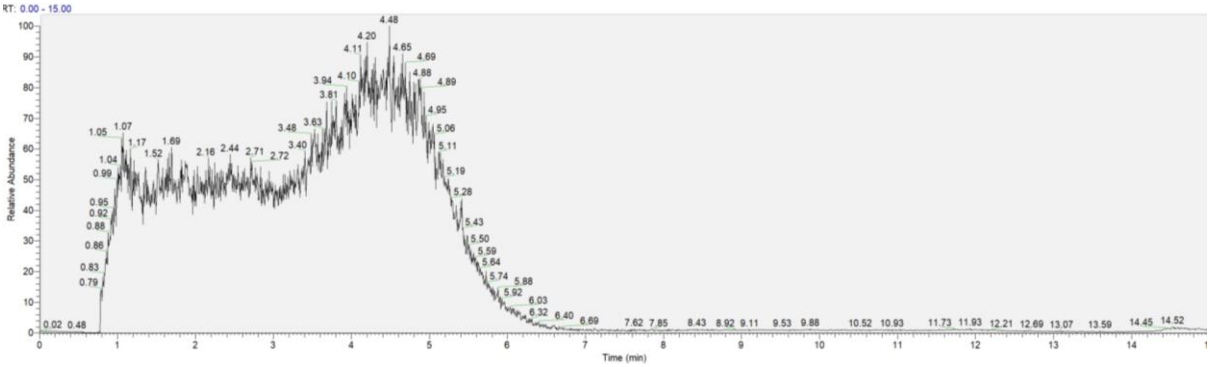

Figure S19: *XIC of SRFA for formula* $C_{14}H_{17}O_8^-$ *(tetraacid alkene* ***4*** *molecular formula).*

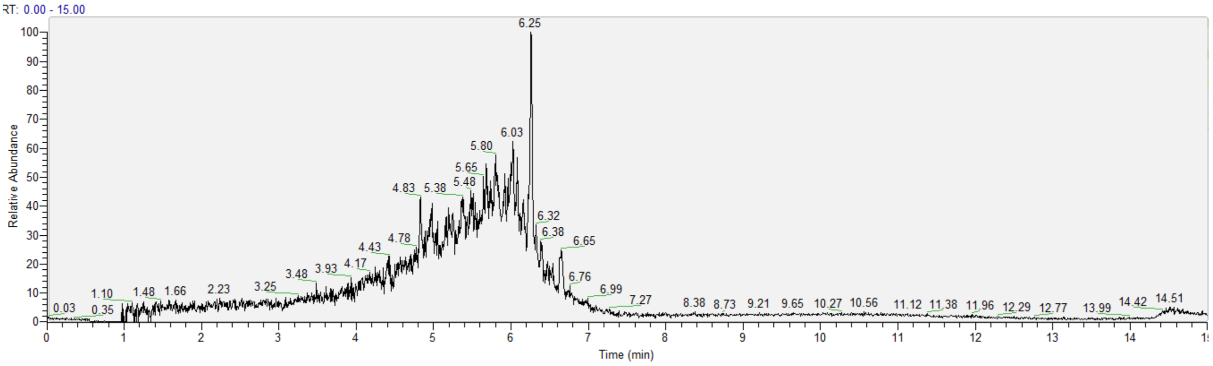

Figure S20: *XIC of SRFA for formula* $C_{12}H_{17}O_4^-$ *(diacid* ***5*** *molecular formula).*

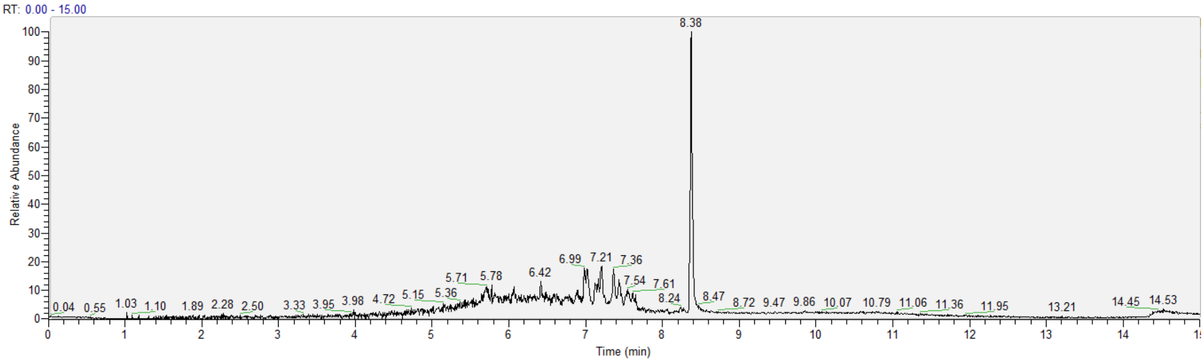

Figure S21: *XIC of SRFA for formula* $C_{13}H_{19}O_4^-$ *(methyl diacid* ***6*** *molecular formula).*

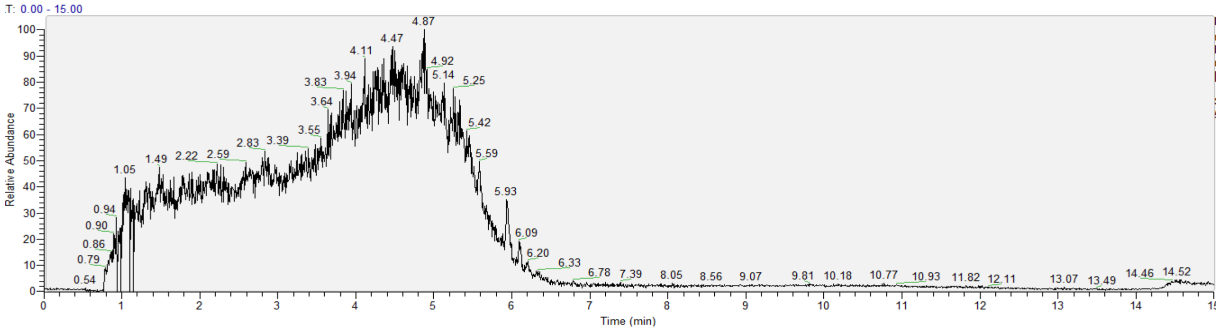

Figure S22: *XIC of SRFA for formula* $C_{13}H_{17}O_7^-$ *(sec alcohol* ***8*** *and tert alcohol* ***9*** *molecular formula).*

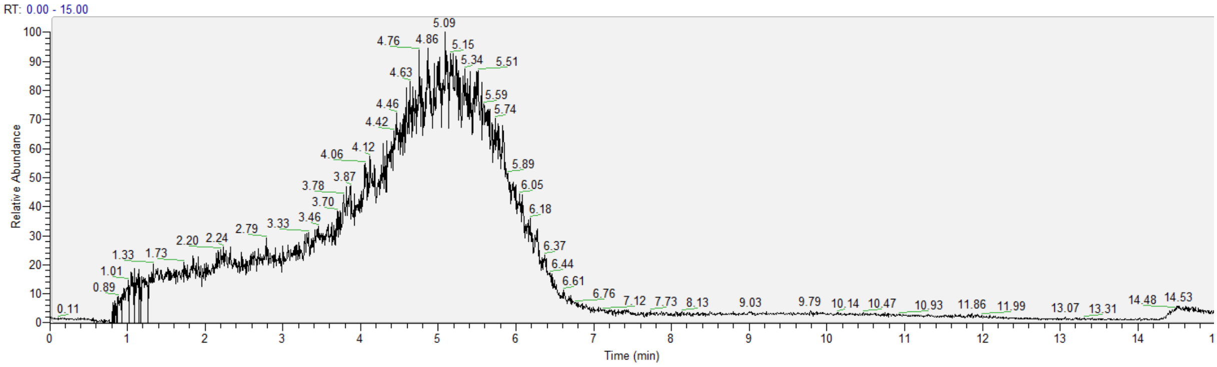

Figure S23: *XIC of SRFA for formula* $C_{14}H_{19}O_7^-$ *(ether* ***10*** *molecular formula).*

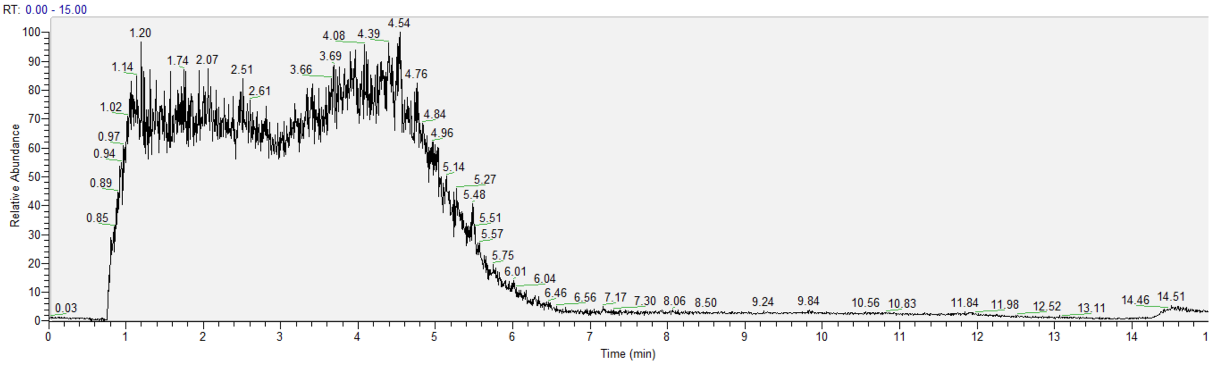

Figure S24: *XIC of SRFA for formula* $C_{13}H_{15}O_7^-$ *(ketone* ***11*** *molecular formula).*

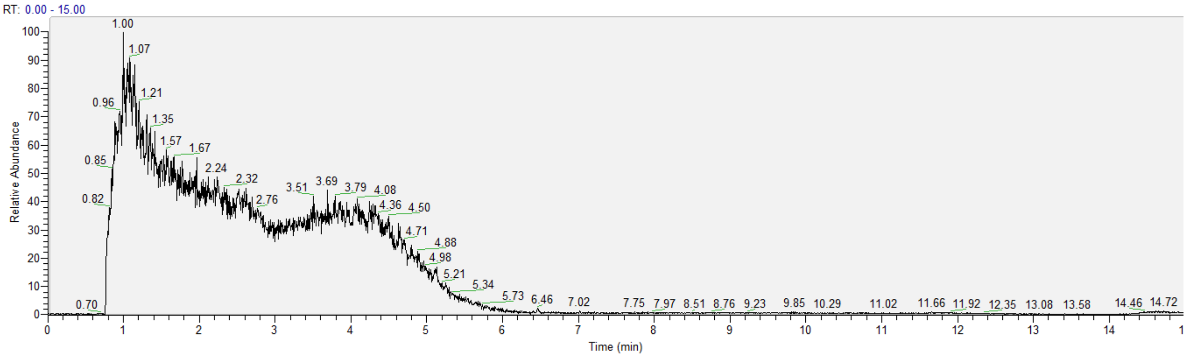

Figure S25: *XIC of SRFA for formula* $C_{13}H_{15}O_8^-$ *(acyloin* ***12*** *molecular formula).*

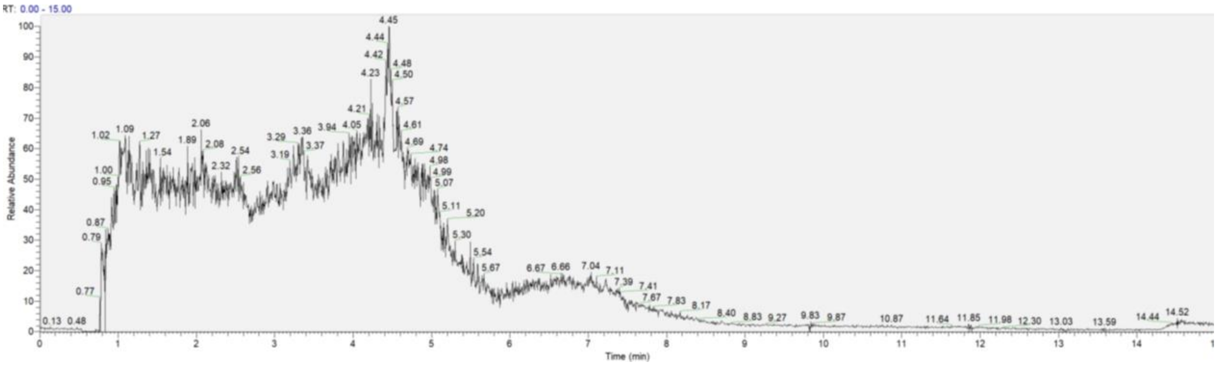

Figure S26: *XIC of SRFA for formula* $C_{13}H_{17}O_8^-$ *(triacid diol* ***13*** *molecular formula).*

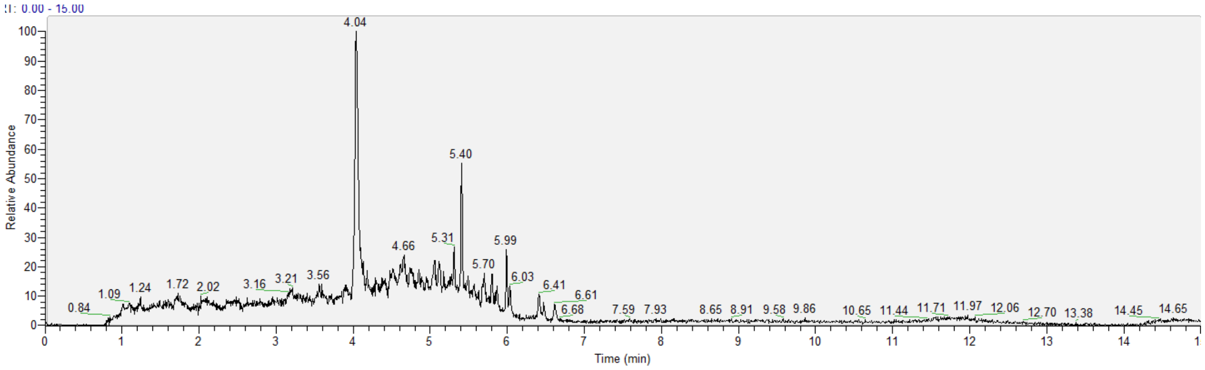

Figure S27: *XIC of SRFA for formula* $C_{12}H_{17}O_6^-$ *(diacid diol* ***14*** *molecular formula).*

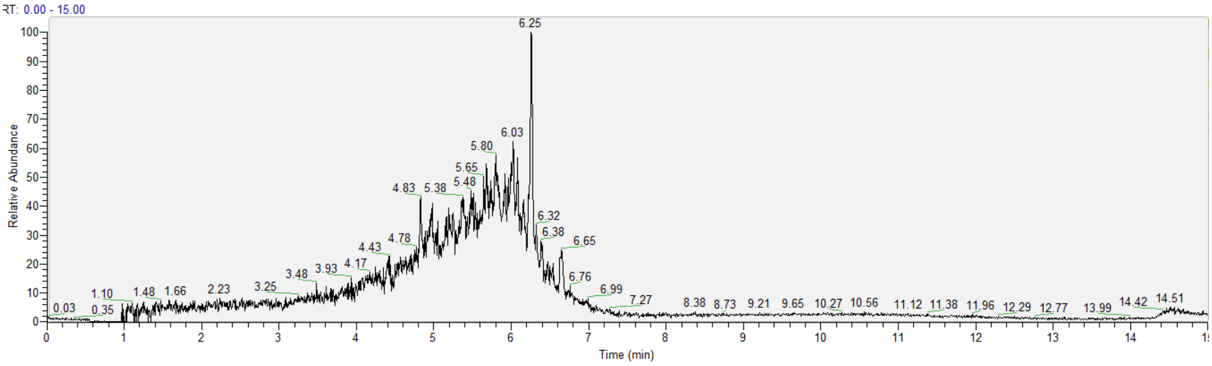

Figure S28: XIC of SRFA for formula $C_{13}H_{19}O_6^-$ (methyl diacid diol 15 molecular formula).

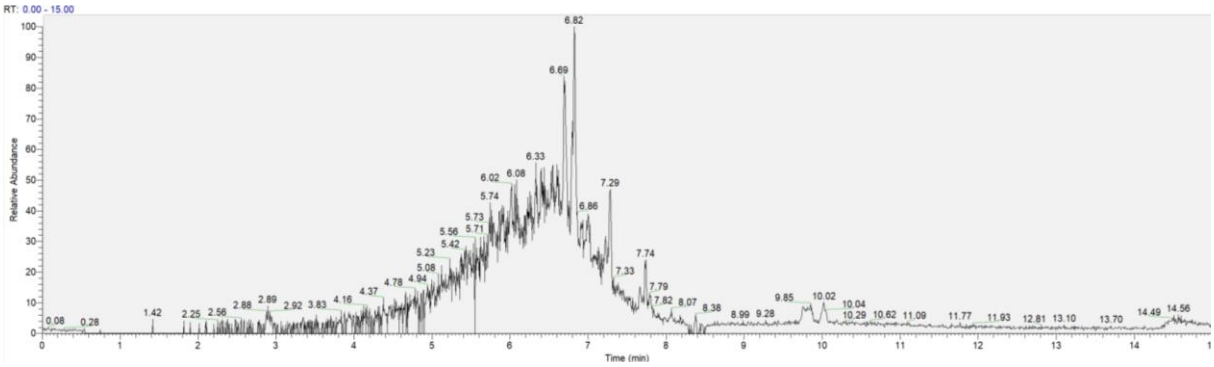

Figure S29: XIC of SRFA for formula $C_{15}H_{23}O_6^-$ (acid ester diol 16 molecular formula).

Fragmentation Data for CRAM Analogues 5-16

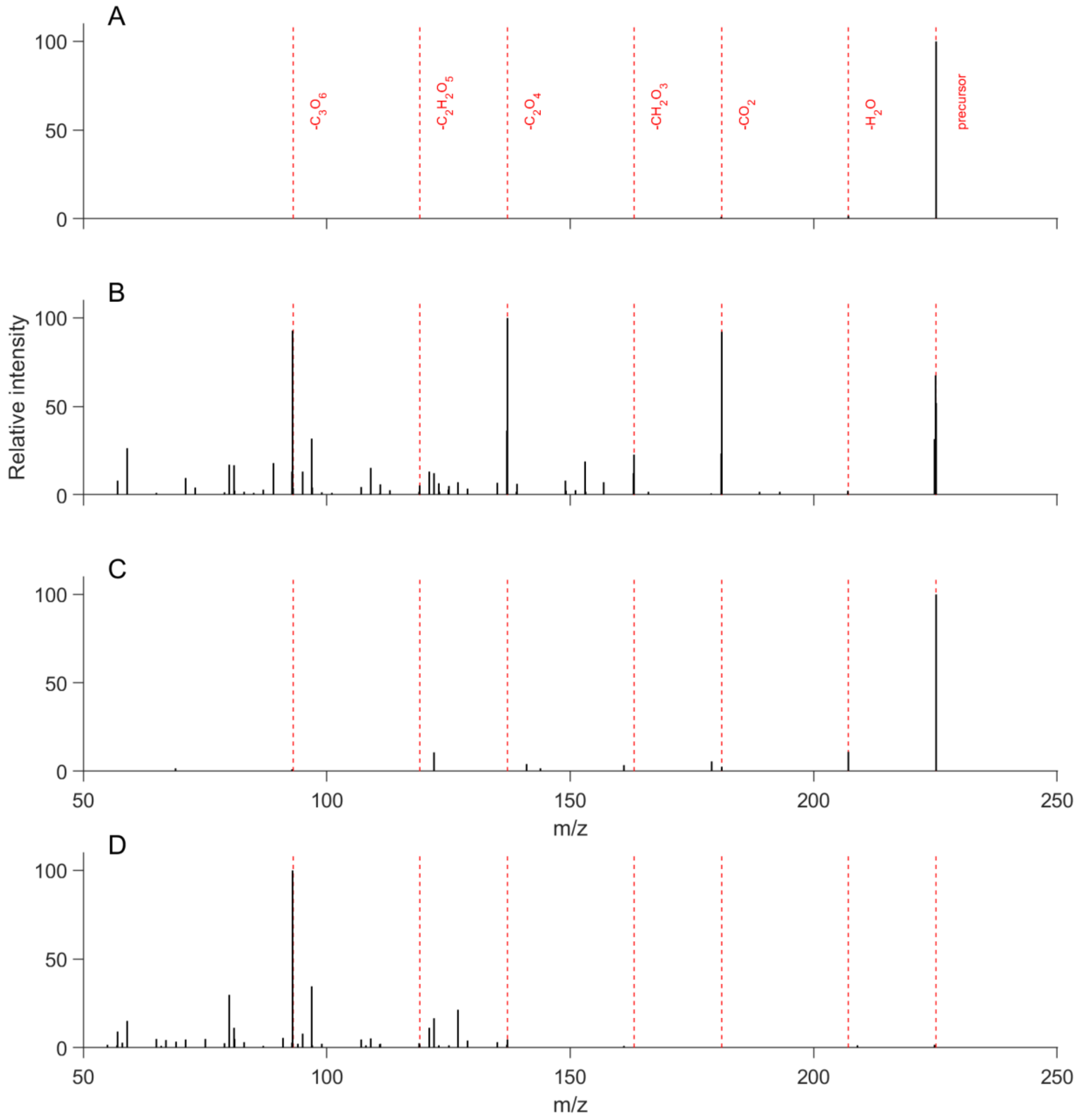

Figure S30: *a) HCD35 of diacid **5**, b) HCD35 of TRM225, c) HCD75 of diacid diol **5**, d) HCD75 of TRM225.*

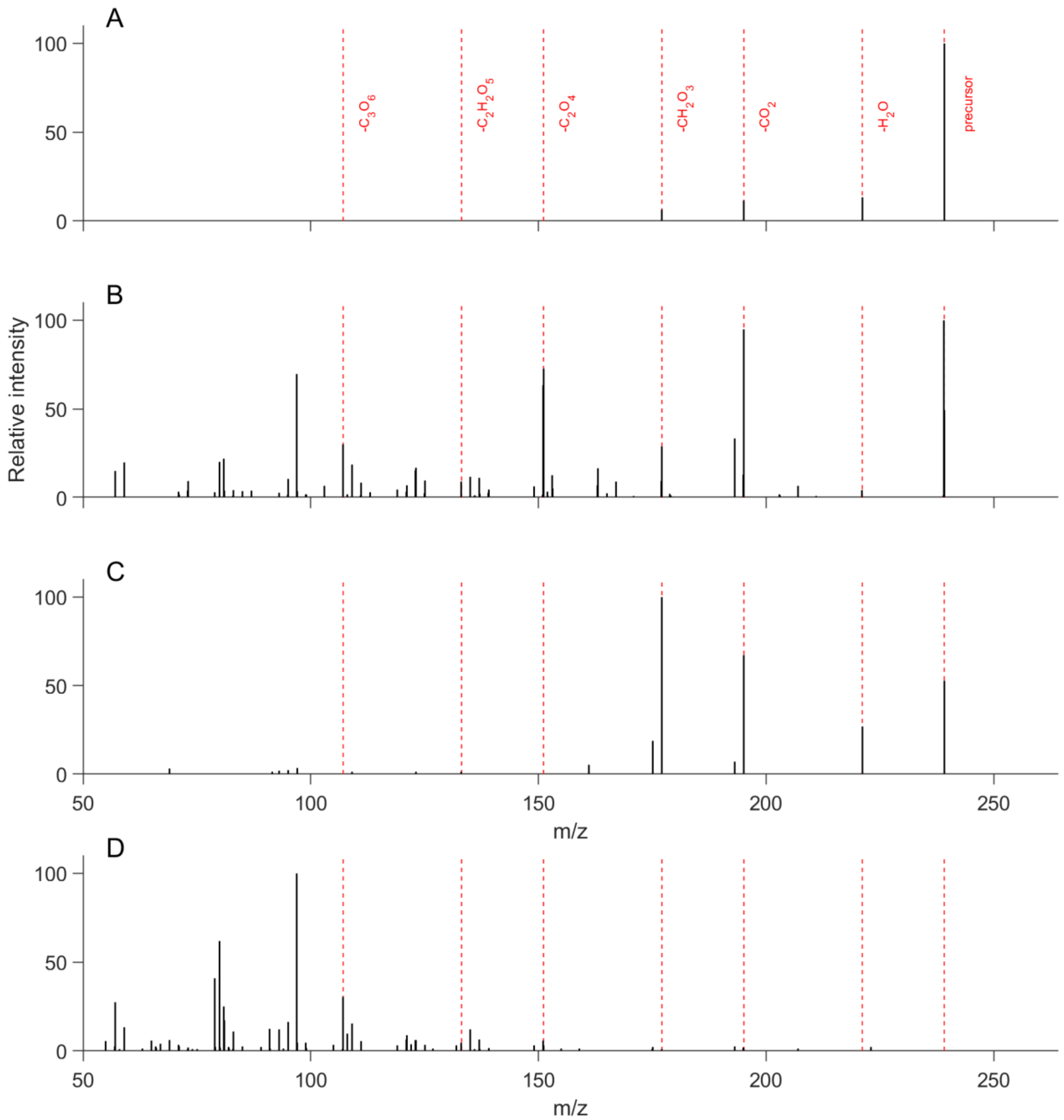

Figure S31: *a) HCD35 of methyl diacid **6**, b) HCD35 of TRM239, c) HCD75 of methyl diacid **6**, d) HCD75 of TRM239.*

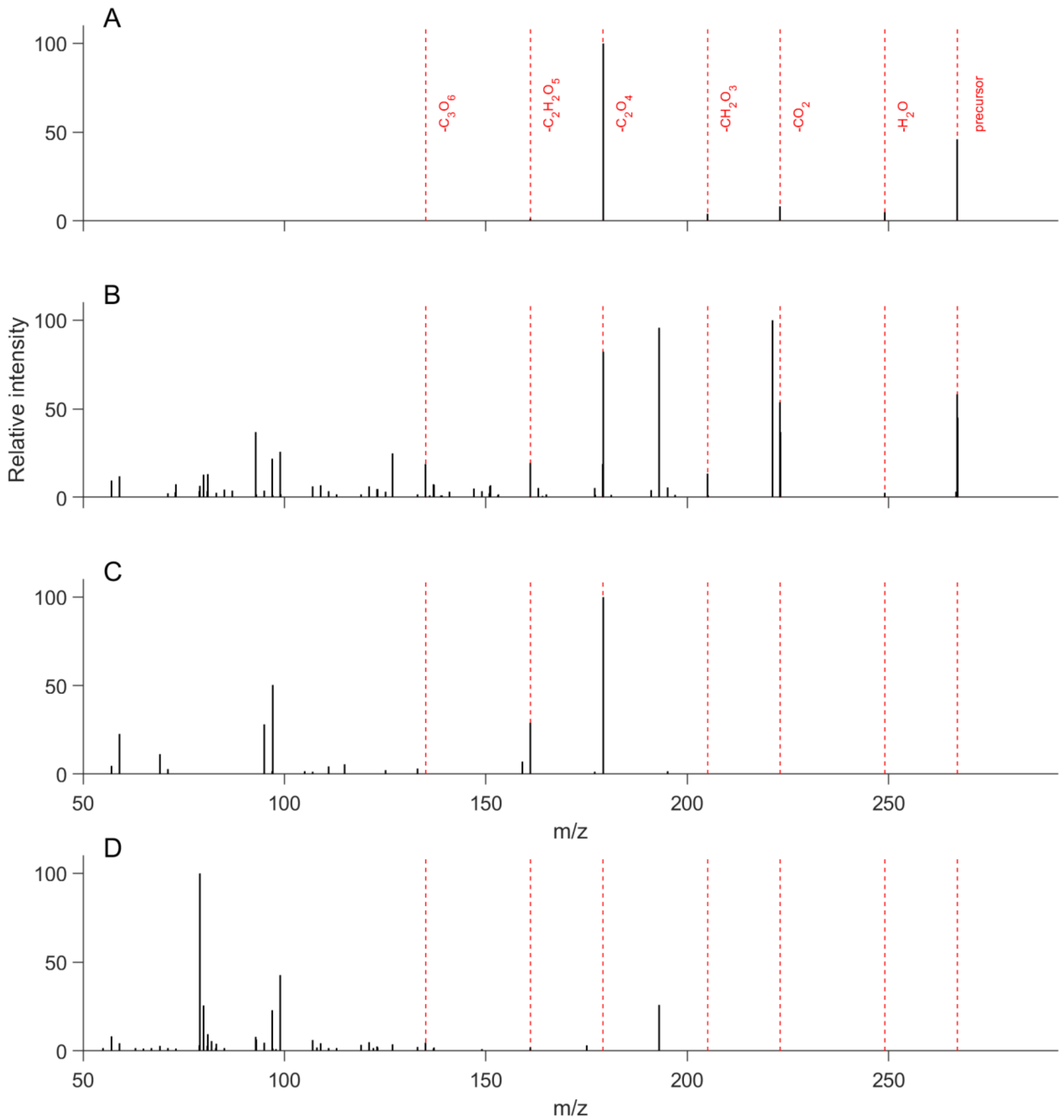

Figure S32: *a) HCD35 of lactone **7**, b) HCD35 of TRM267, c) HCD75 of lactone **7**, d) HCD75 of TRM267.*

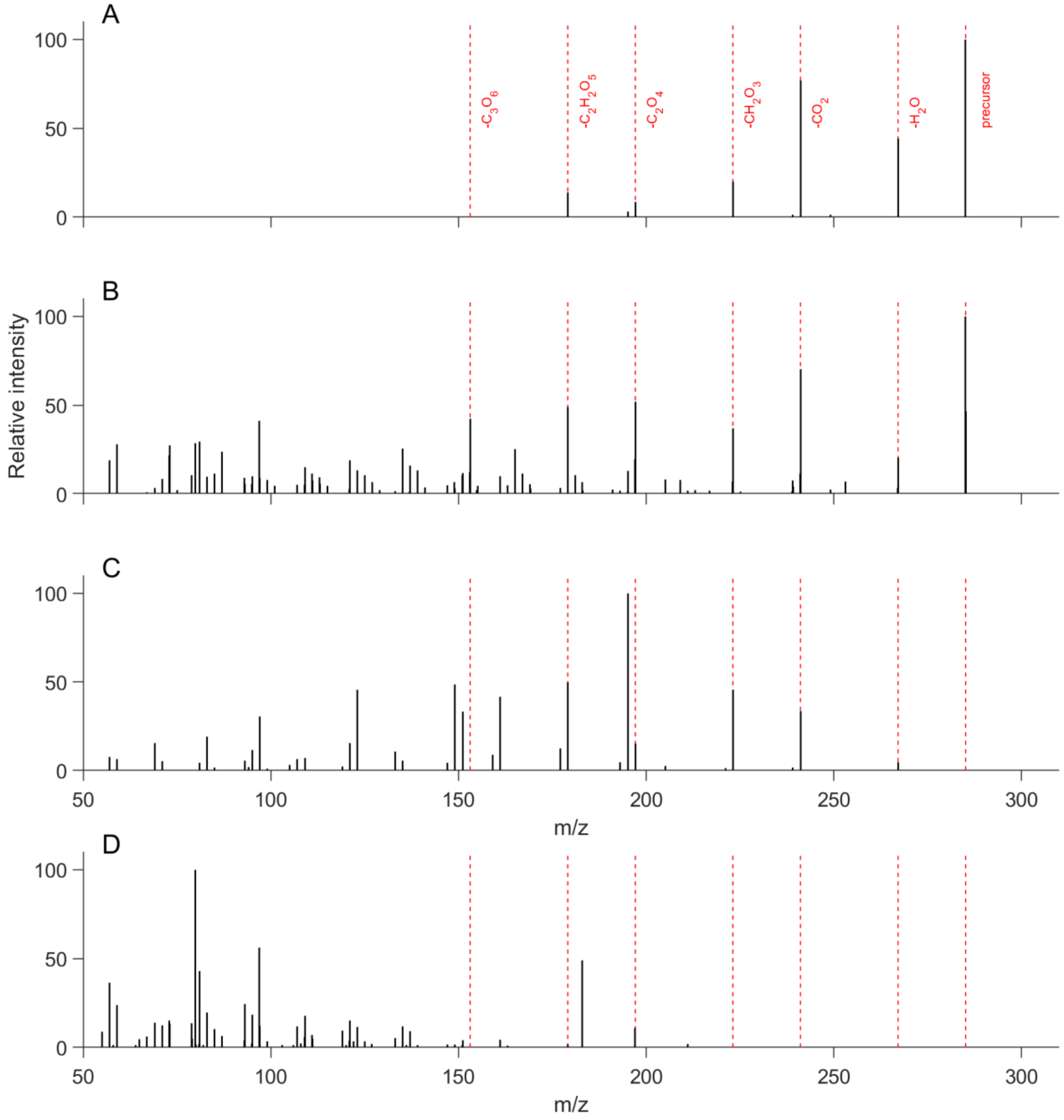

Figure S33: *a) HCD35 of sec alcohol **8**, b) HCD35 of TRM285, c) HCD75 of sec alcohol **8**, d) HCD75 of TRM285.*

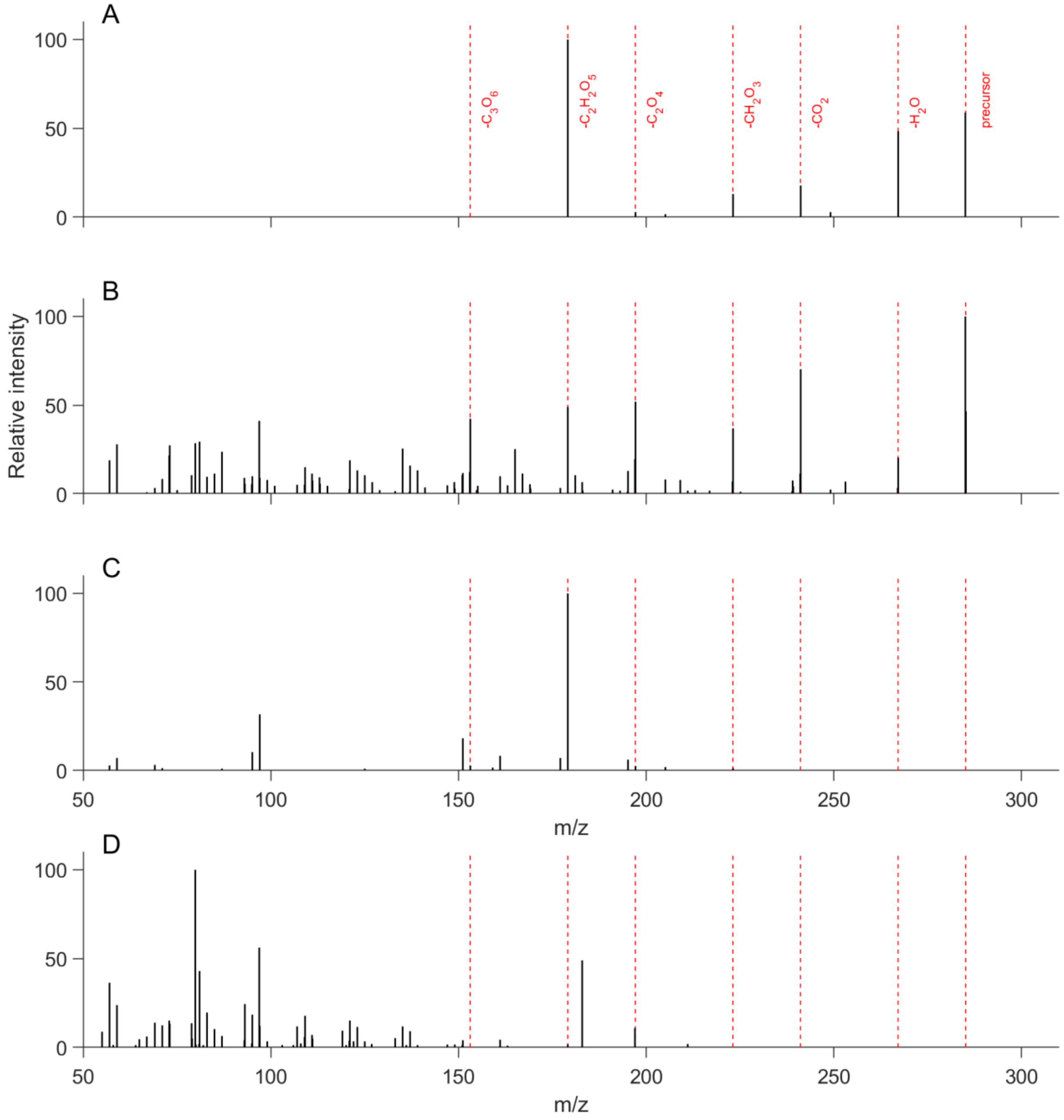

Figure S34: *a) HCD35 of tert alcohol **9**, b) HCD35 of TRM285, c) HCD75 of tert alcohol **9**, d) HCD75 of TRM285.*

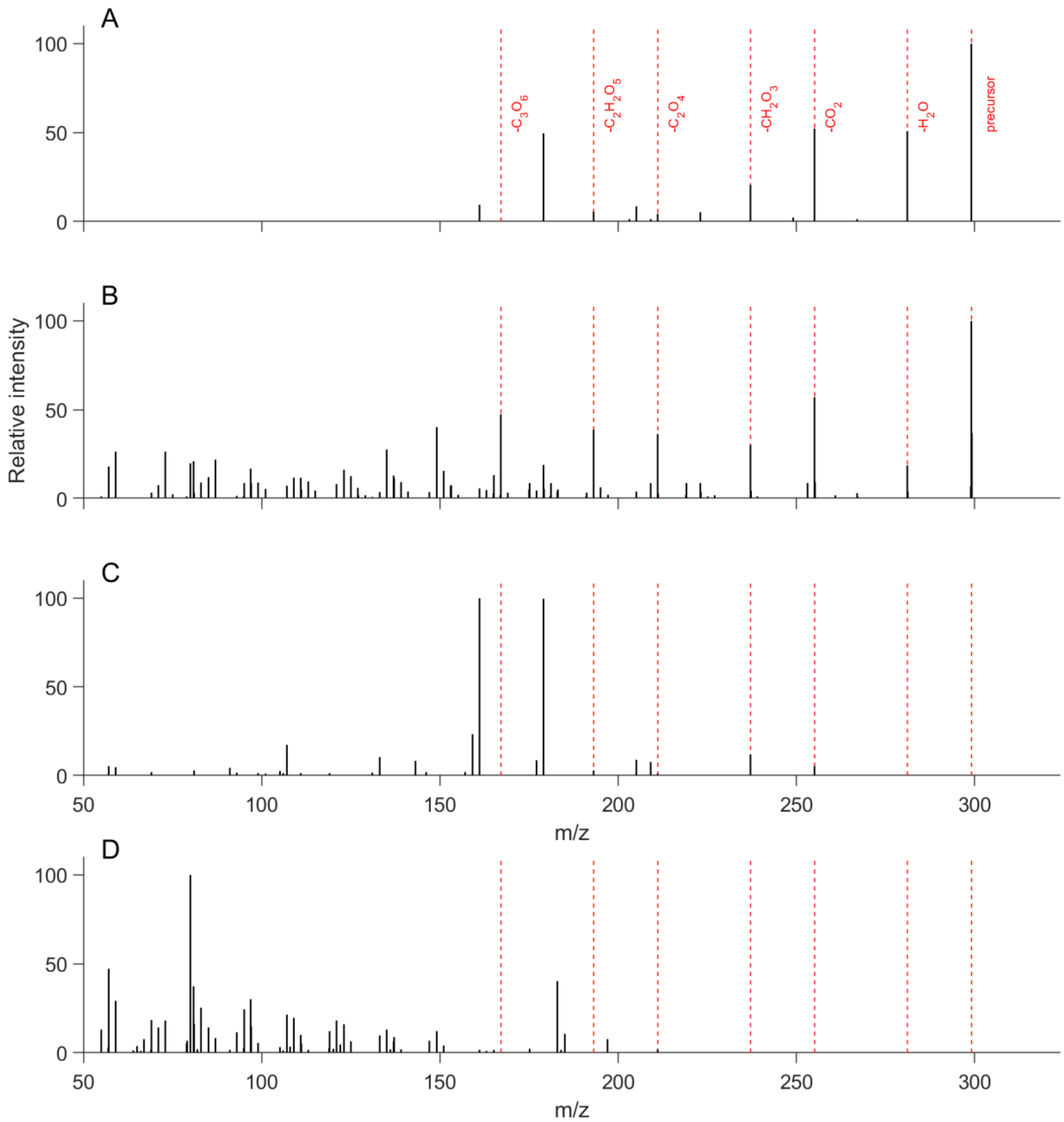

Figure S35: *a) HCD35 of ether **10**, b) HCD35 of TRM299, c) HCD75 of ether **10**, d) HCD75 of TRM299.*

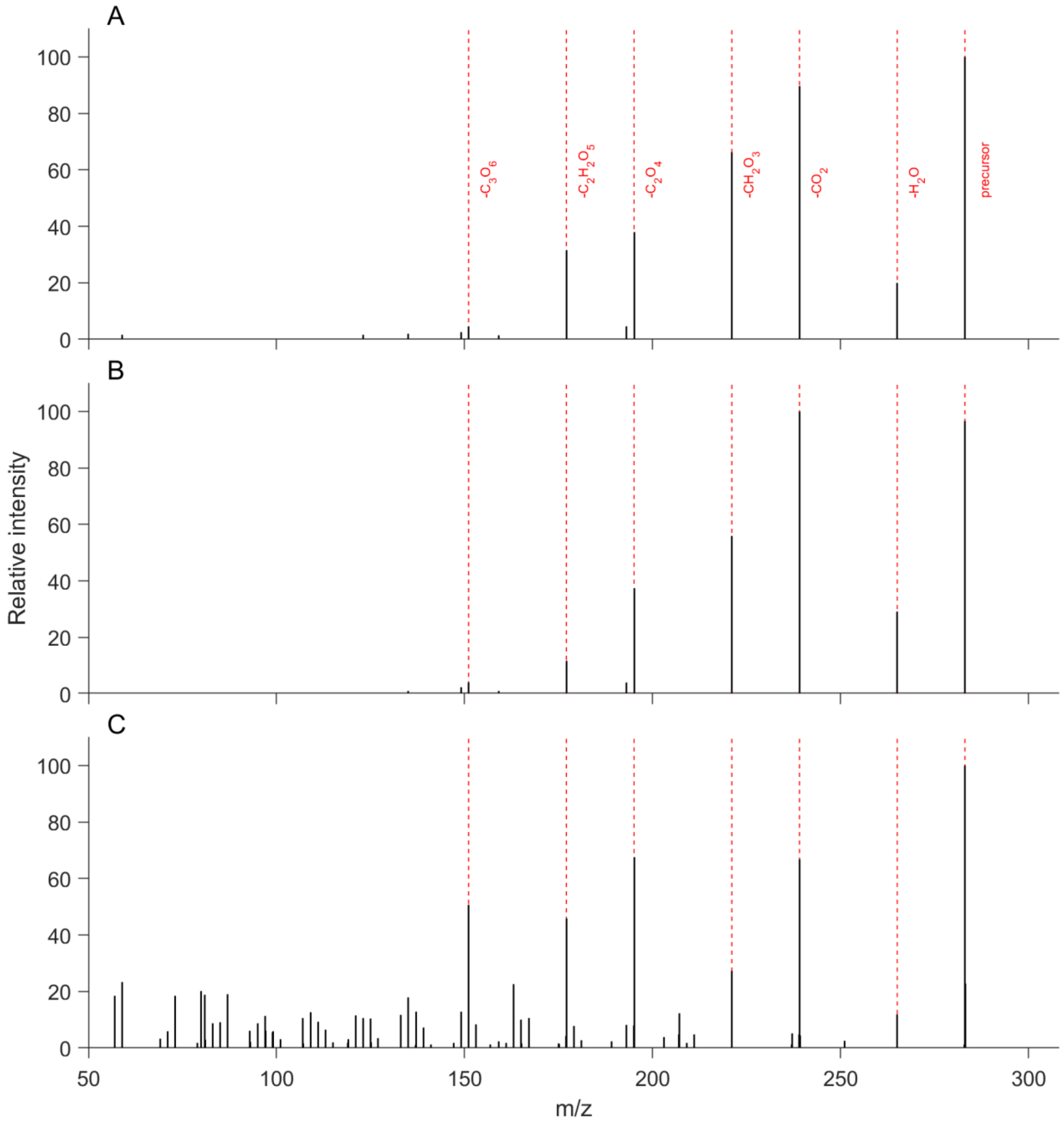

Figure S36: *a) HCD35 of ketone* ***11*** *isomer mix 1, b) HCD35 of ketone* ***11*** *isomer mix 2, c) HCD35 of TRM283.*

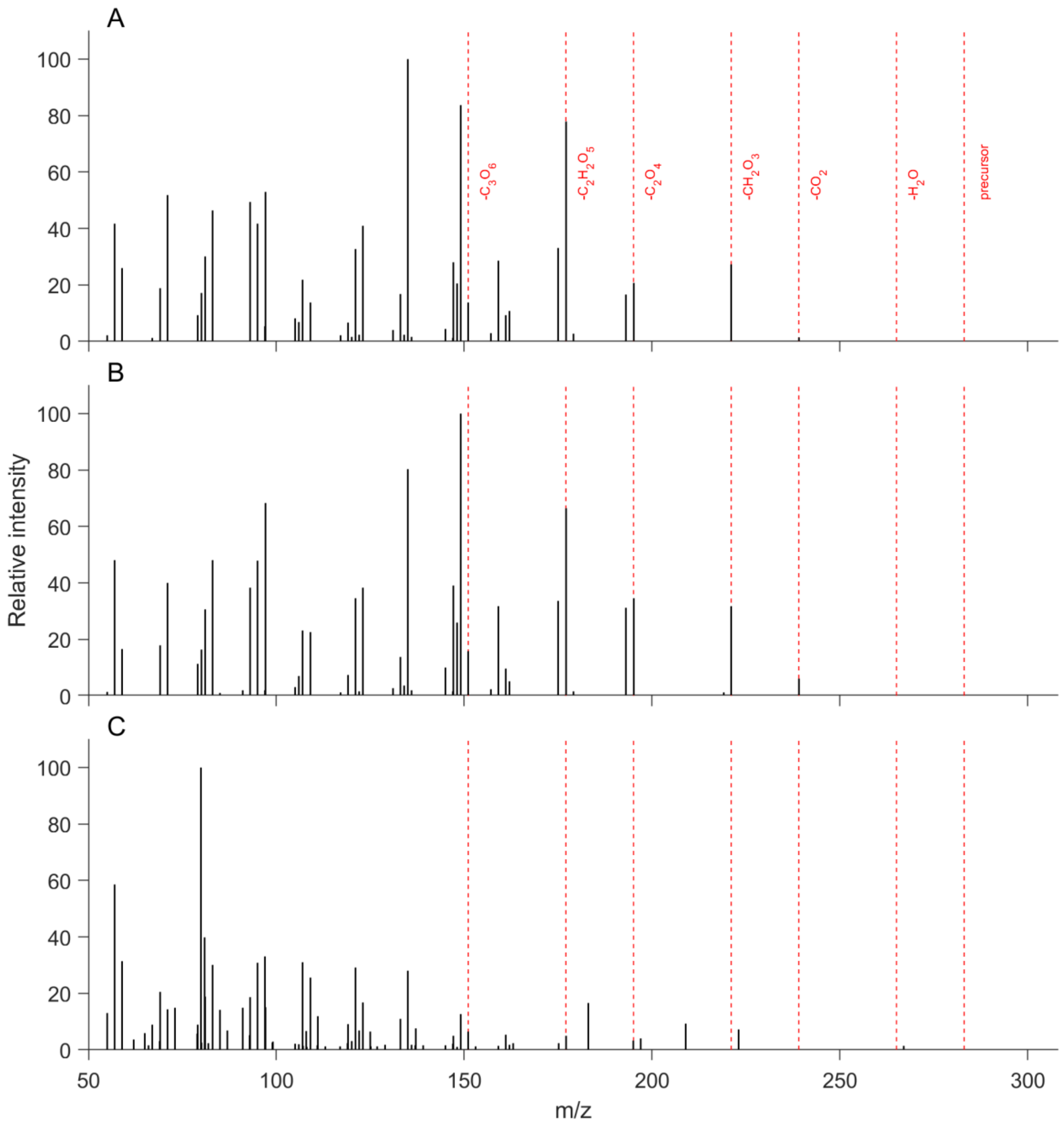

Figure S37: *a) HCD75 of ketone **11** isomer mix 1, b) HCD75 of ketone **11** isomer mix 2, c) HCD75 of TRM283.*

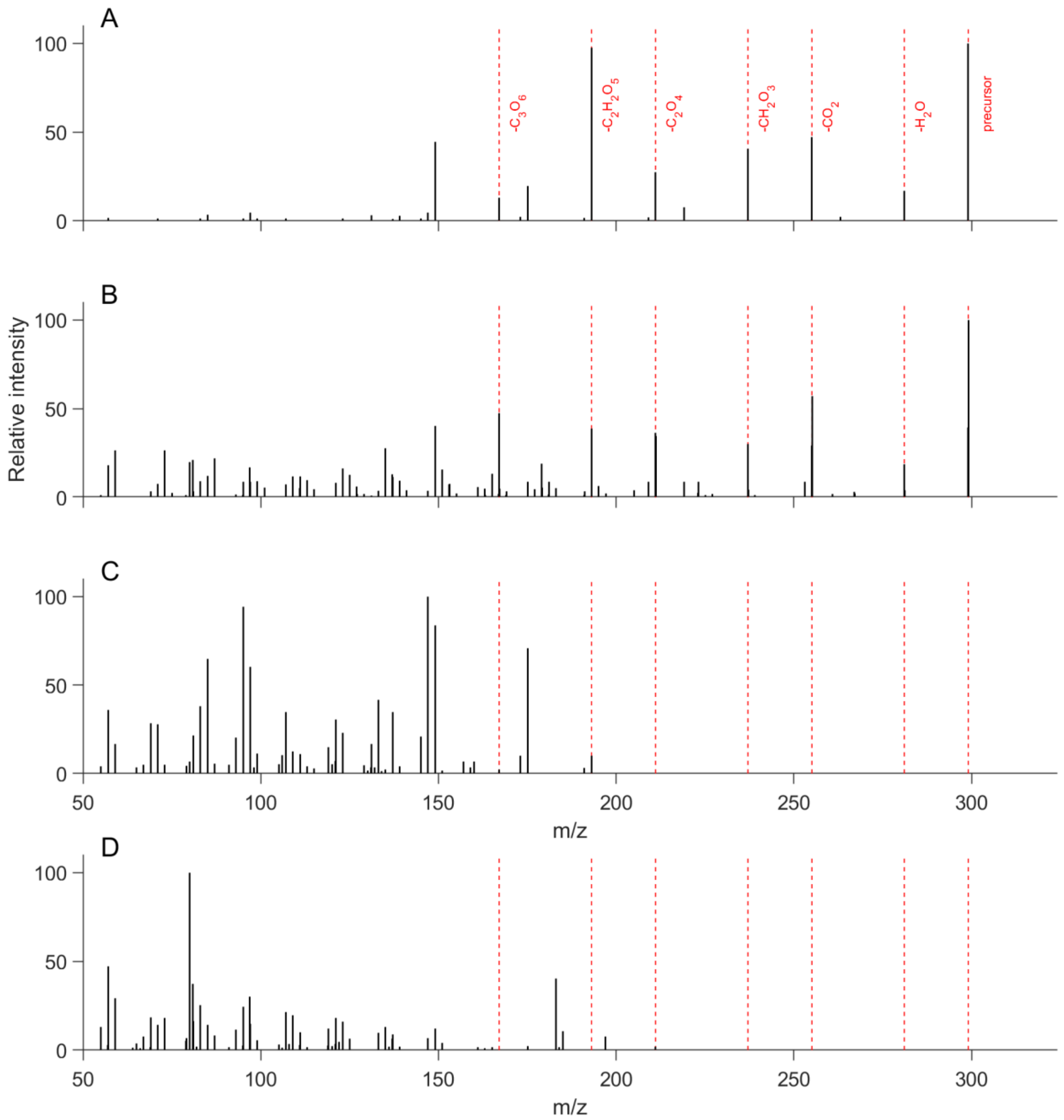

Figure S38: *a) HCD35 of acyloin **12**, b) HCD35 of TRM299, c) HCD75 of acyloin **12**, d) HCD75 of TRM299.*

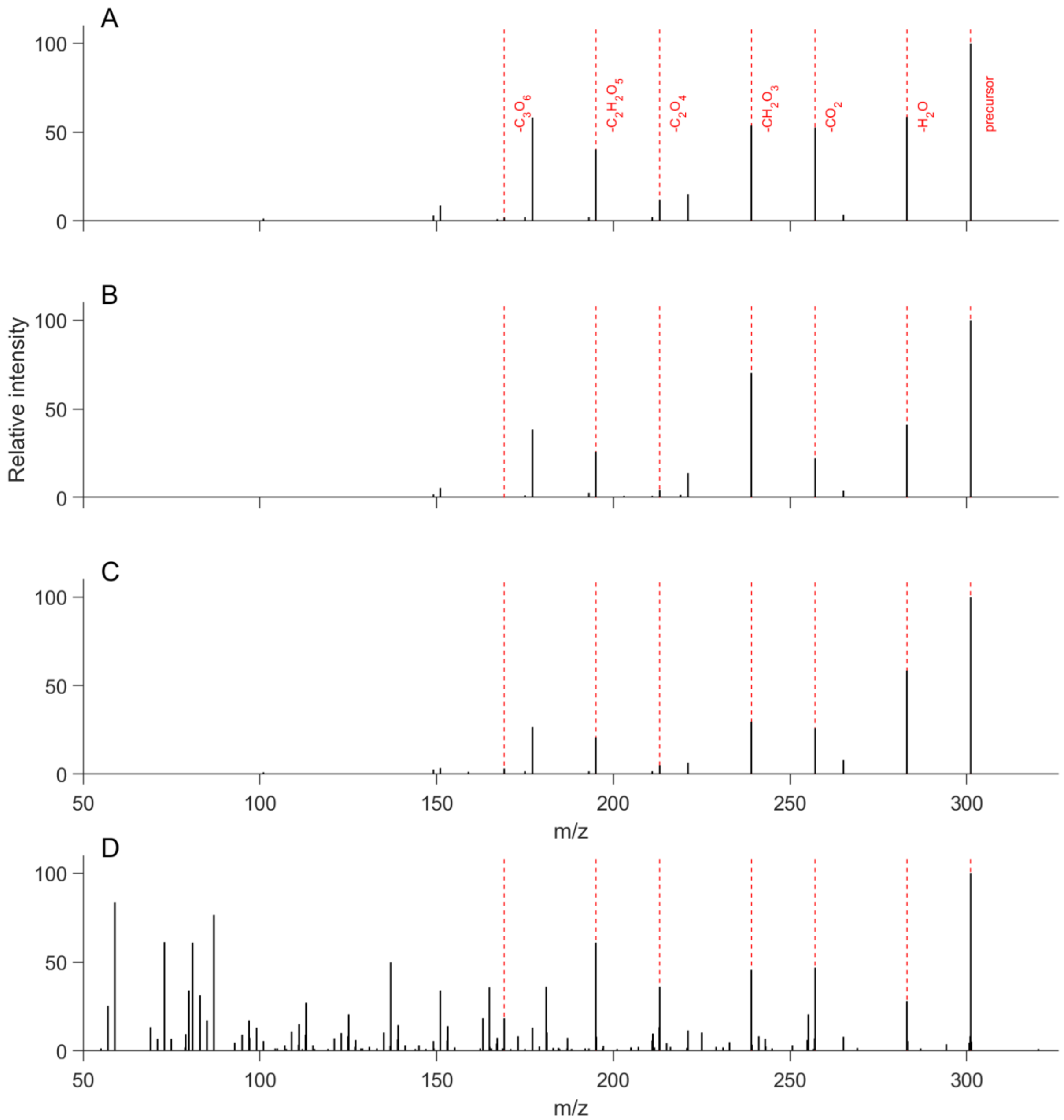

Figure S39: *a) HCD35 of triacid diol* ***13*** *isomer 1, b) HCD35 of triacid diol* ***13*** *isomer 2, c) HCD35 of triacid diol* ***13*** *isomer 3, d) HCD35 of TRM301.*

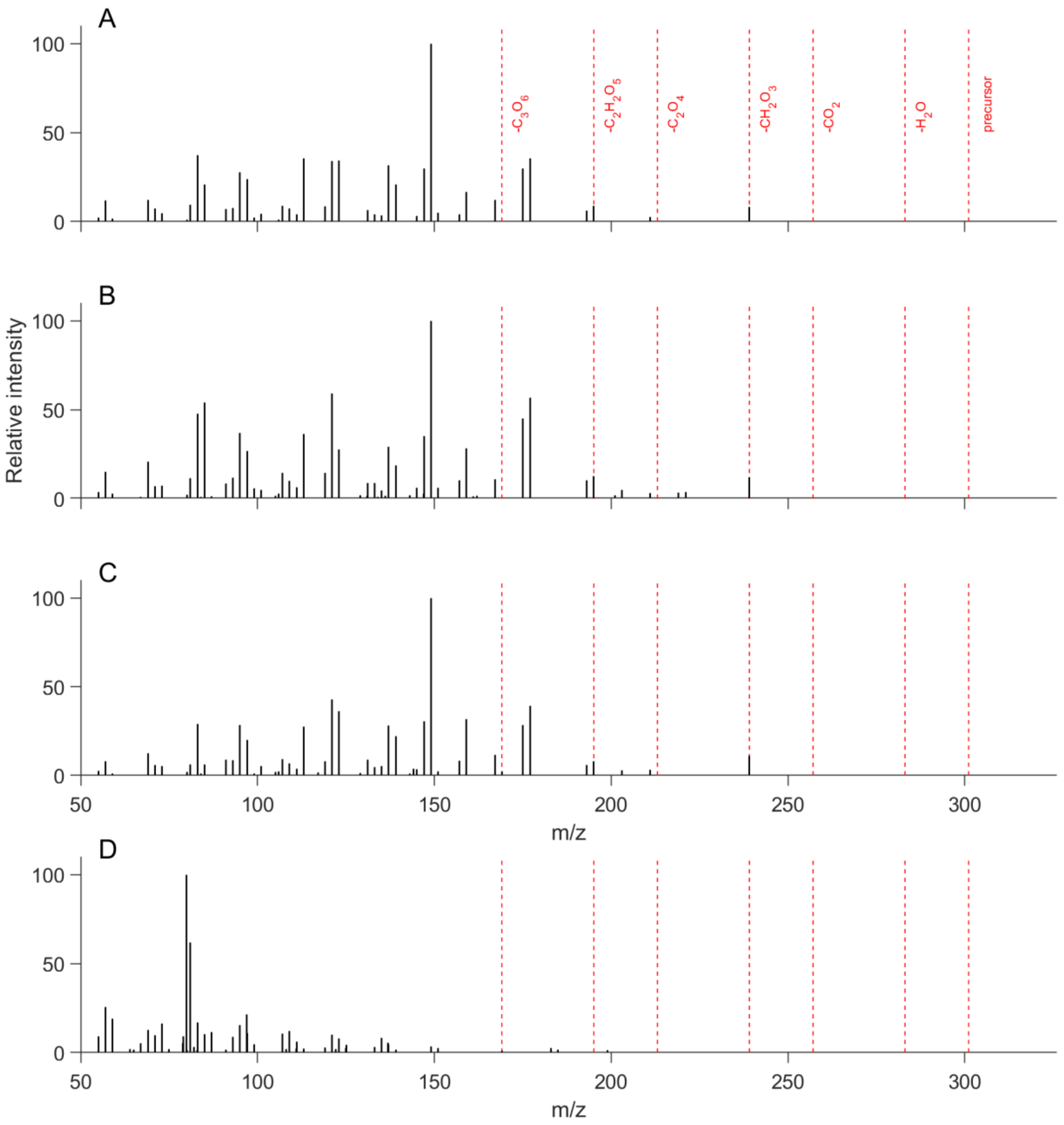

***Figure S40:** a) HCD75 of triacid diol **13** isomer 1, b) HCD75 of triacid diol **13** isomer 2, c) HCD75 of triacid diol **13** isomer 3, d) HCD75 of TRM301.*

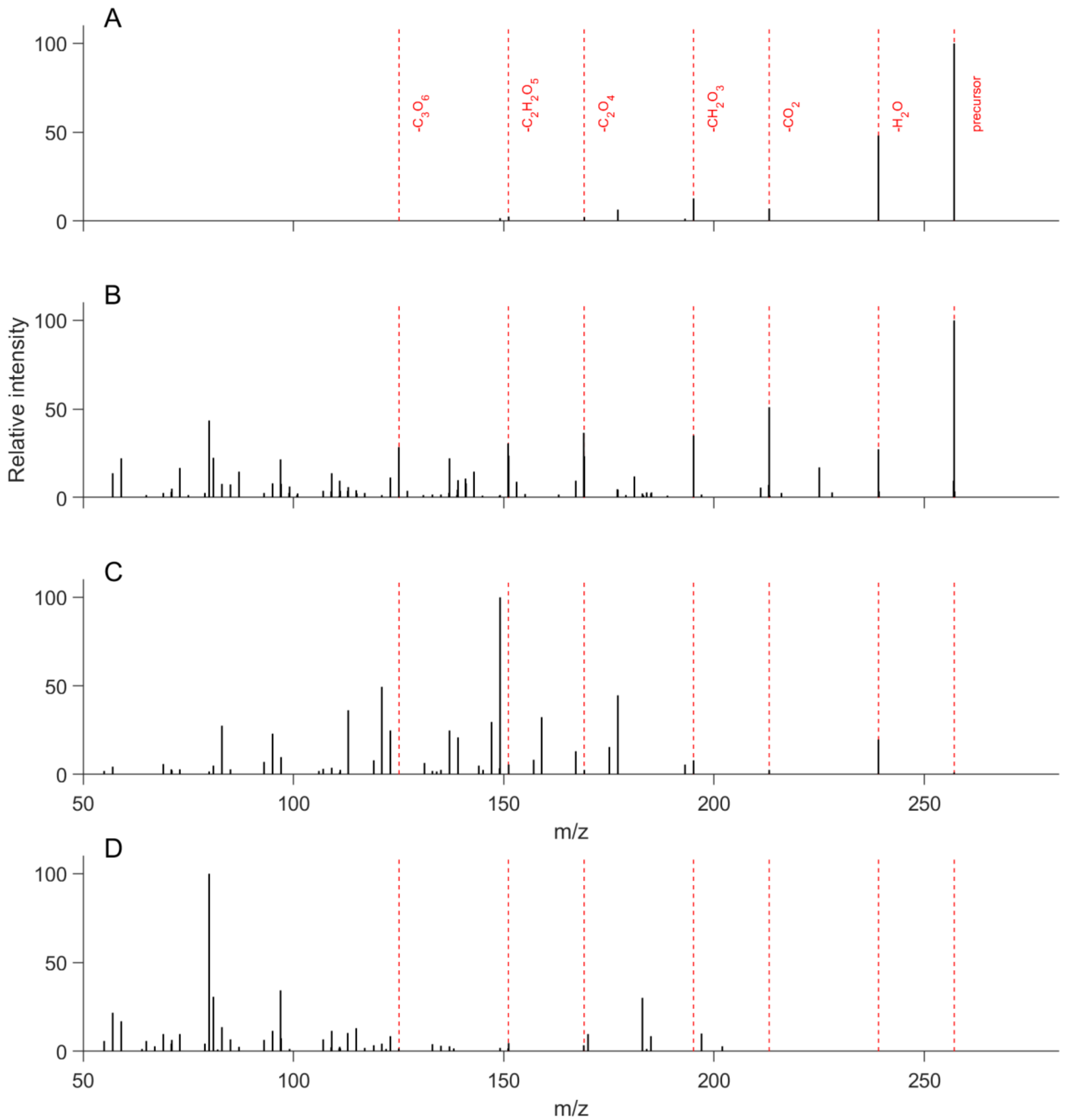

Figure S41: *a) HCD35 of diacid diol **14**, b) HCD35 of TRM257, c) HCD75 of diacid diol **14**, d) HCD75 of TRM257.*

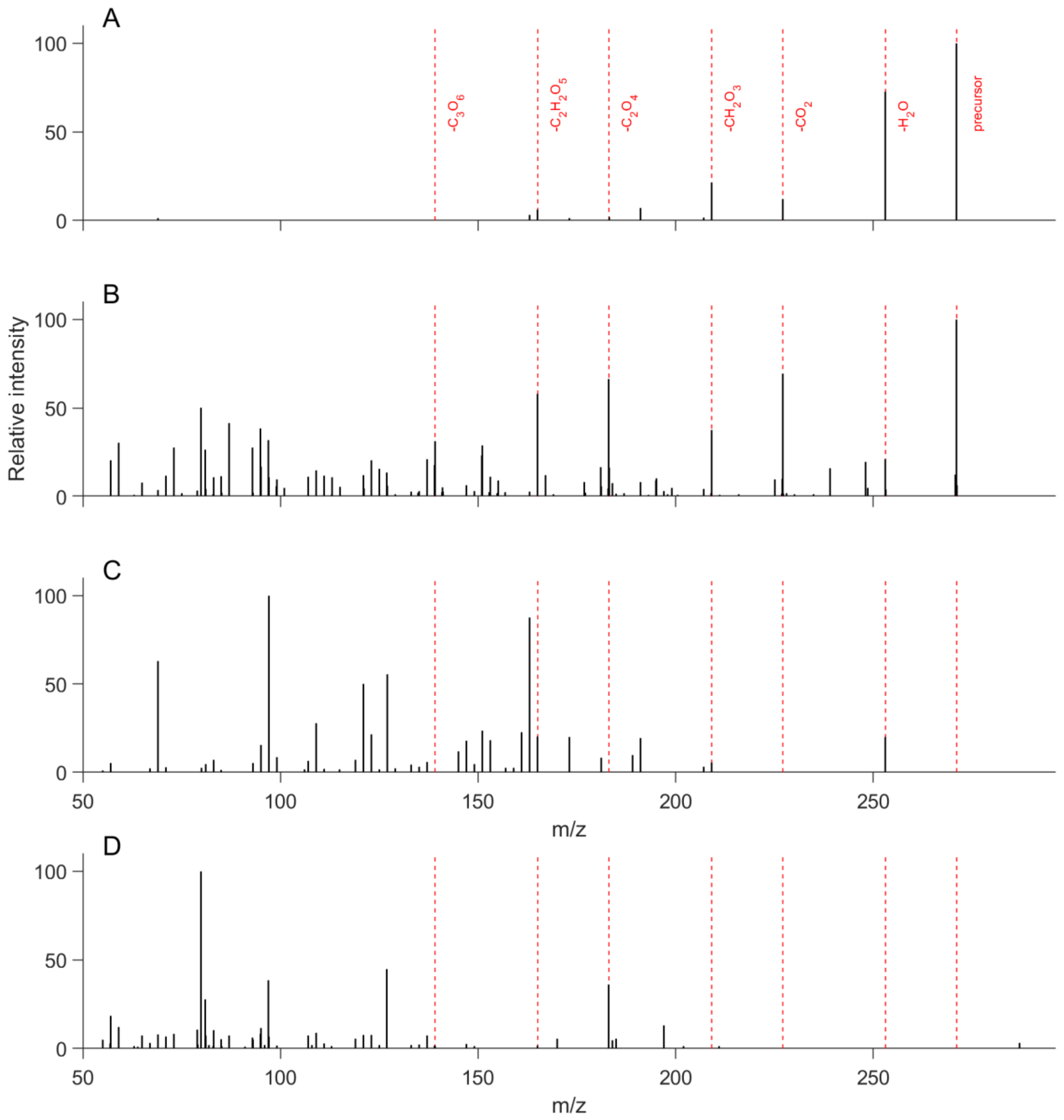

***Figure S42:** a) HCD35 of methyl diacid diol **15**, b) HCD35 of TRM271, c) HCD75 of methyl diacid diol **15**, d) HCD75 of TRM271.*

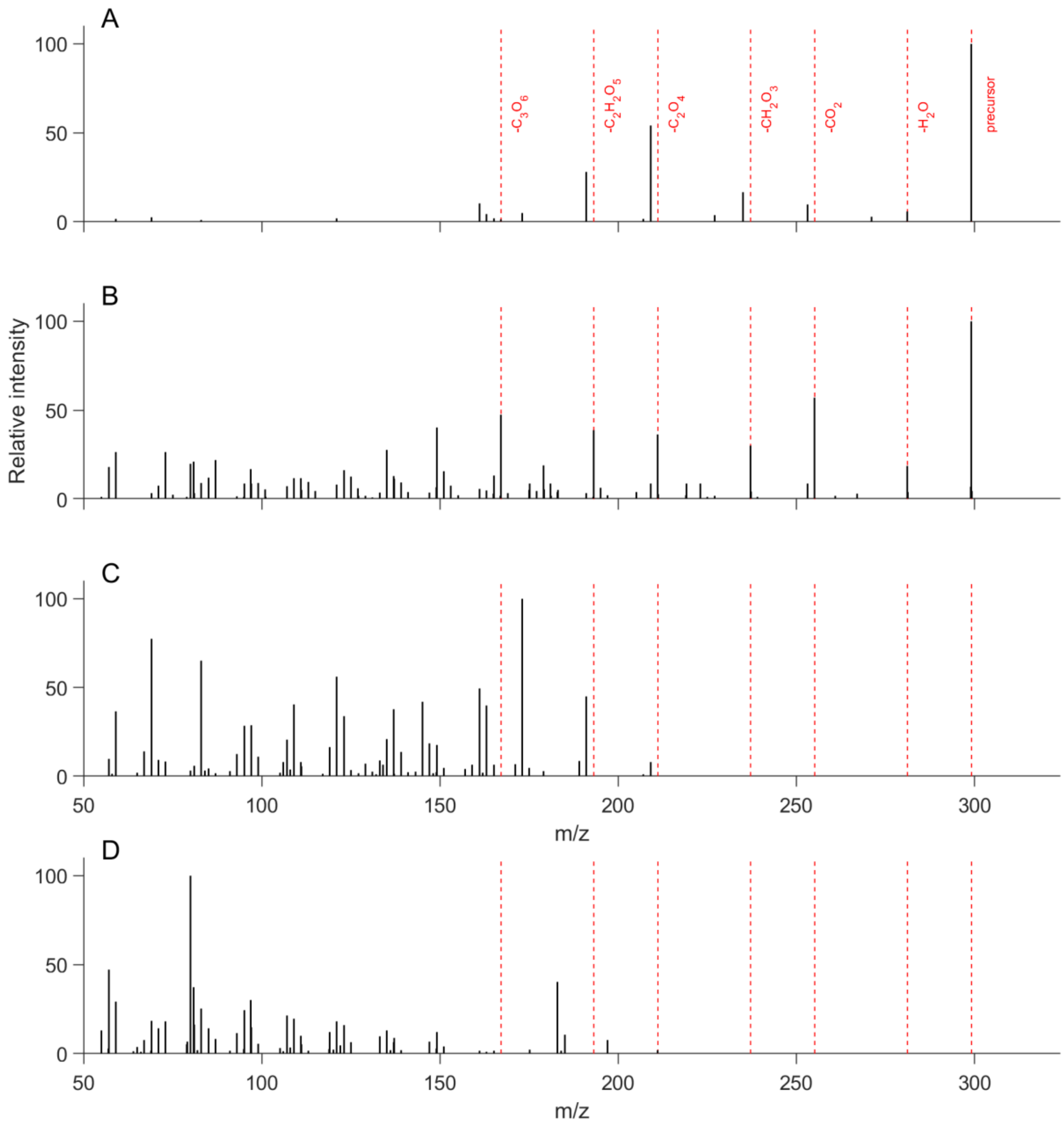

Figure S43: *a) HCD35 of ester* ***16****, b) HCD35 of TRM299, c) HCD75 of ester* ***16****, d) HCD75 of TRM299.*

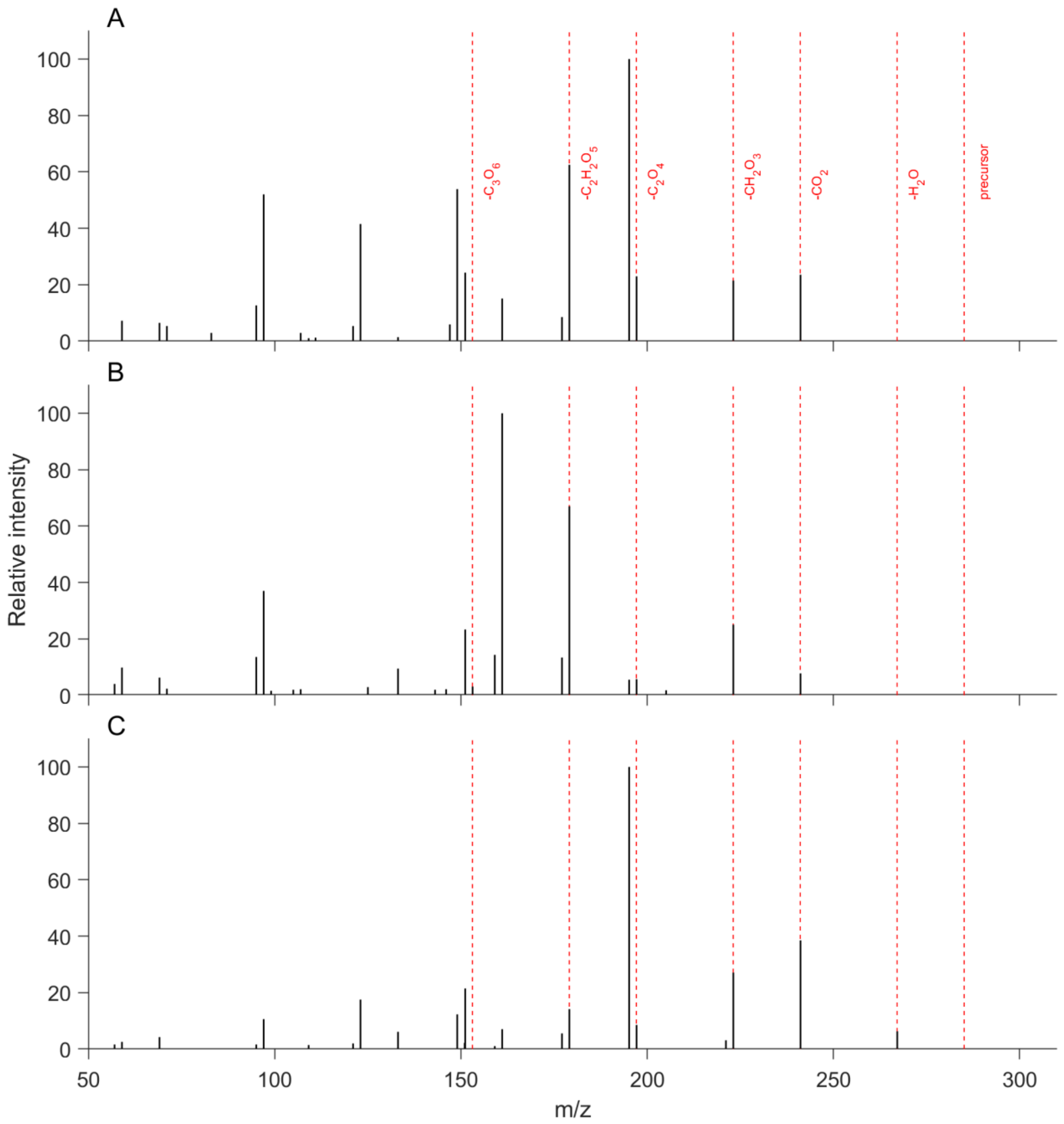

Figure S44: *a) HCD75 of sec alcohol **8** peak with retention time 2.16 minutes, b) HCD75 of sec alcohol **8** peak with retention time 4.20 minutes, c) HCD75 of sec alcohol **8** peak with retention time 4.63 minutes.*

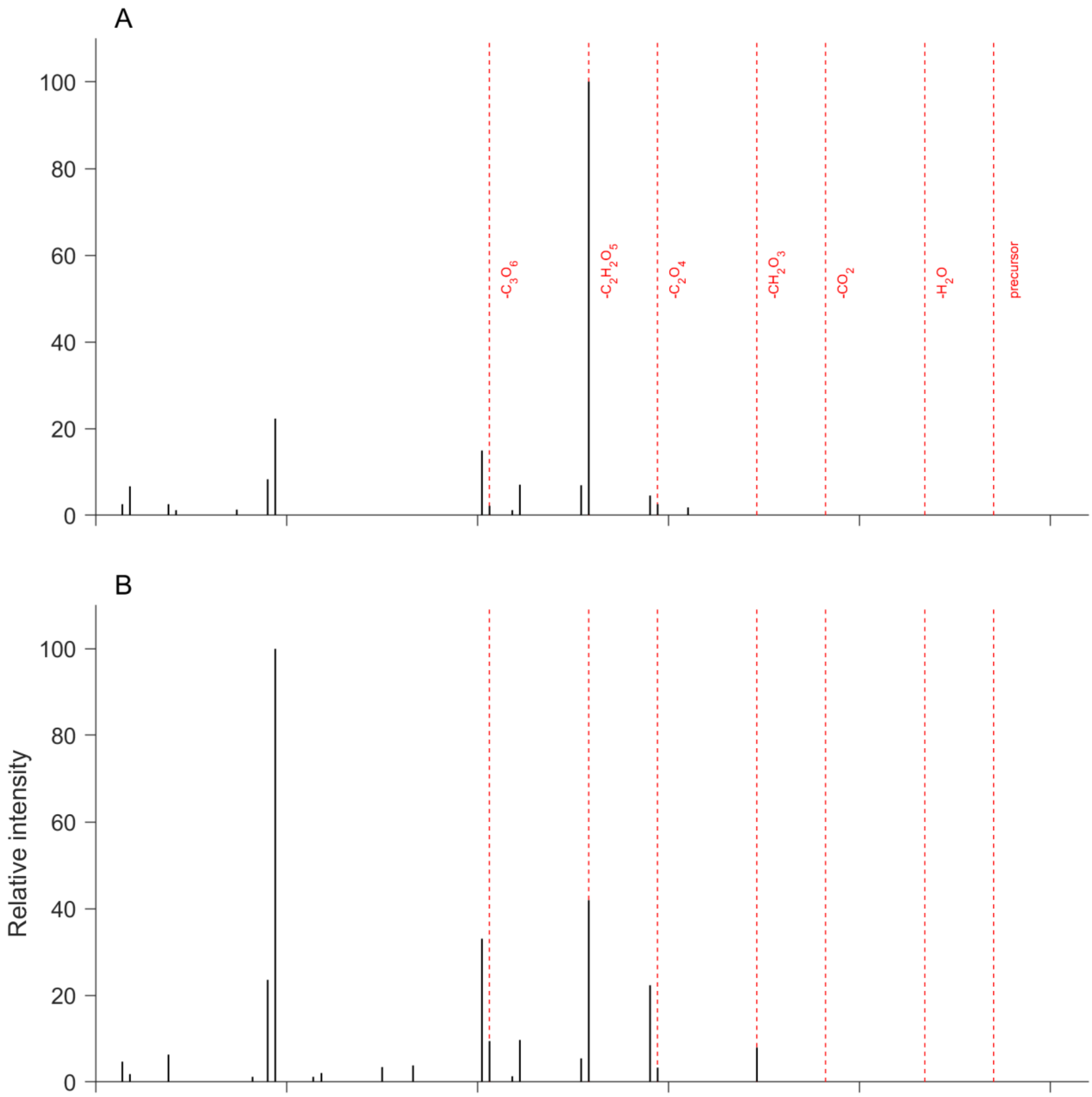

Figure S45: *a) HCD75 of tert alcohol* ***9*** *peak with retention time 1.80 minutes, b) HCD75 of tert alcohol* ***9*** *peak with retention time 4.59 minutes.*

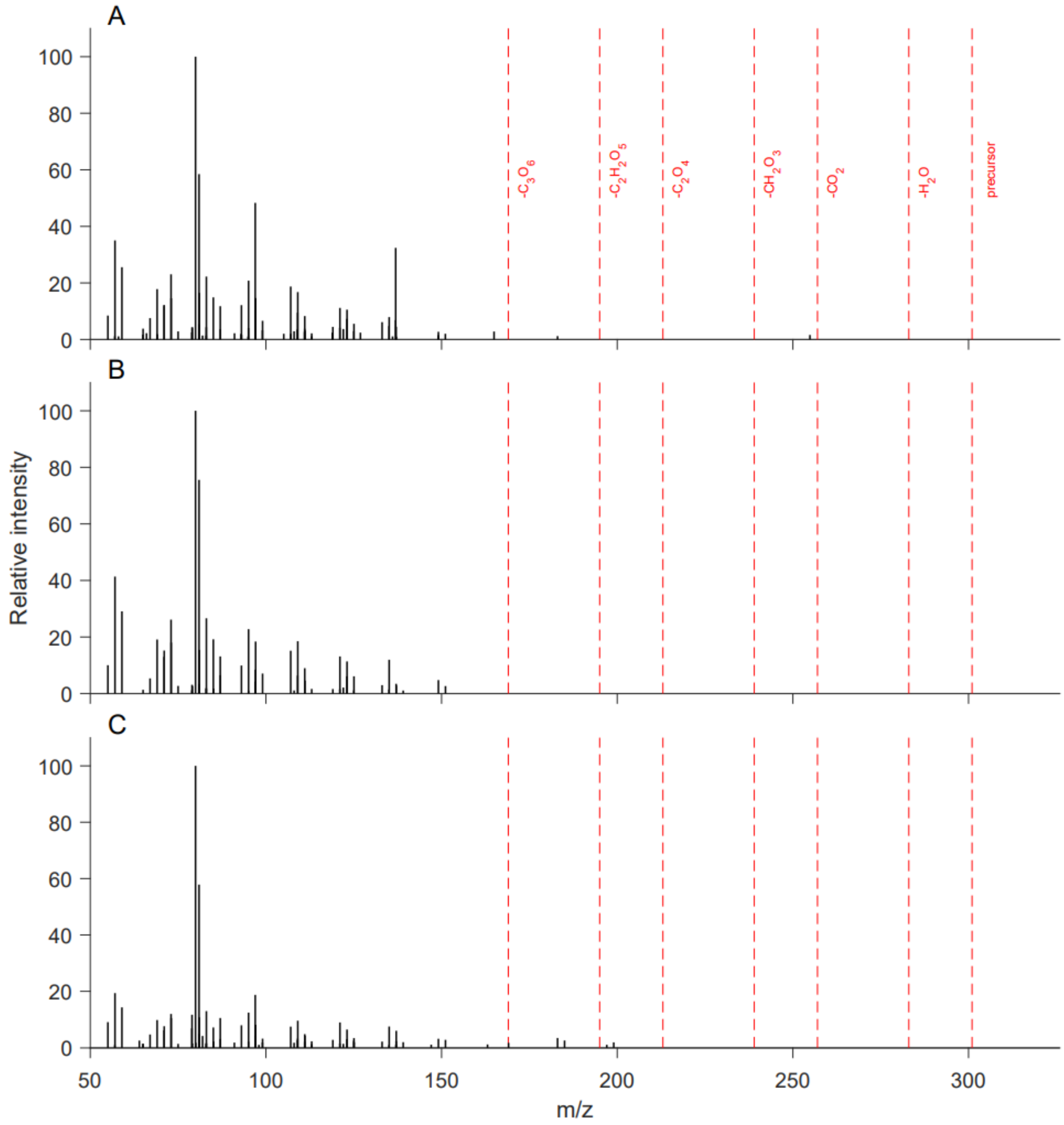

Figure S46: *a) HCD75 of TRM301 from 0.5 to 2 minutes, b) HCD75 of TRM301 from 2 to 4 minutes, c) HCD75 of TRM301 from 4 to 10 minutes.*

NMR Data of Synthetic Intermediates

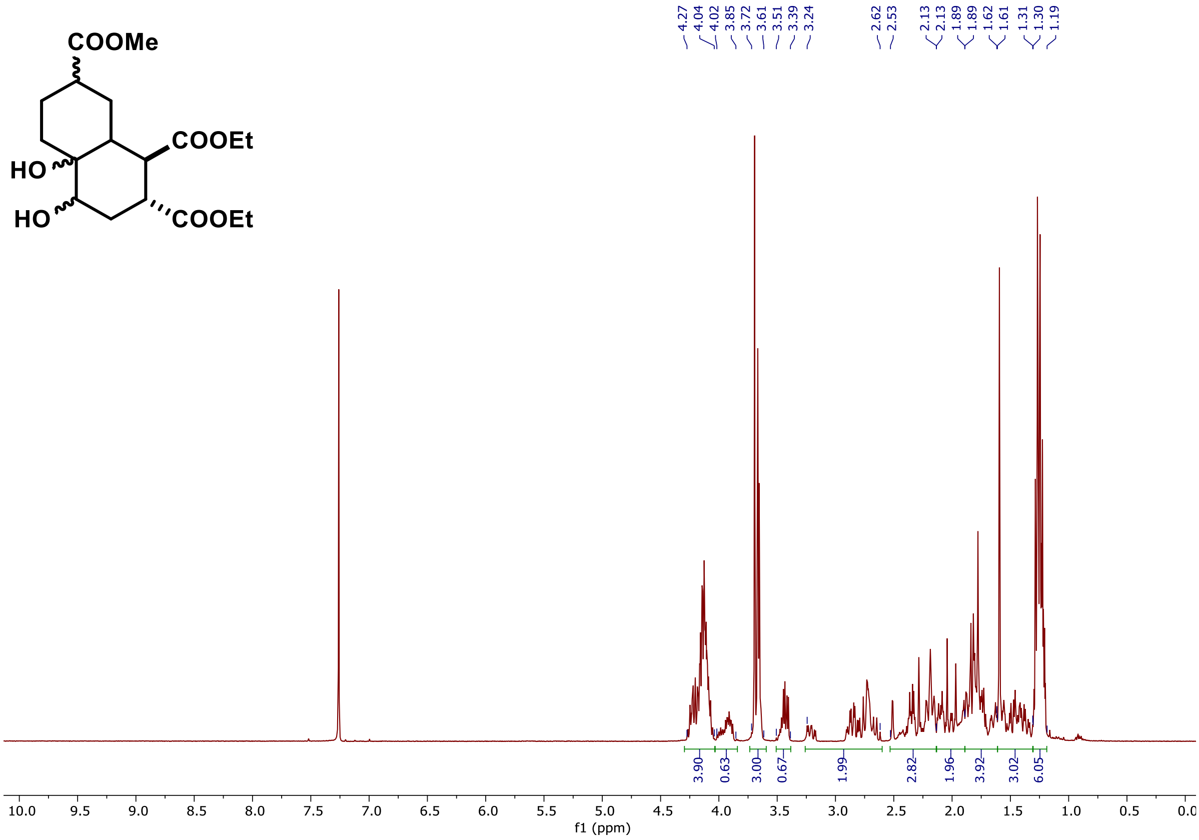

Figure S47: *^{1}H NMR spectrum of diol triester **18** (400 MHz, $CDCl_3$).*

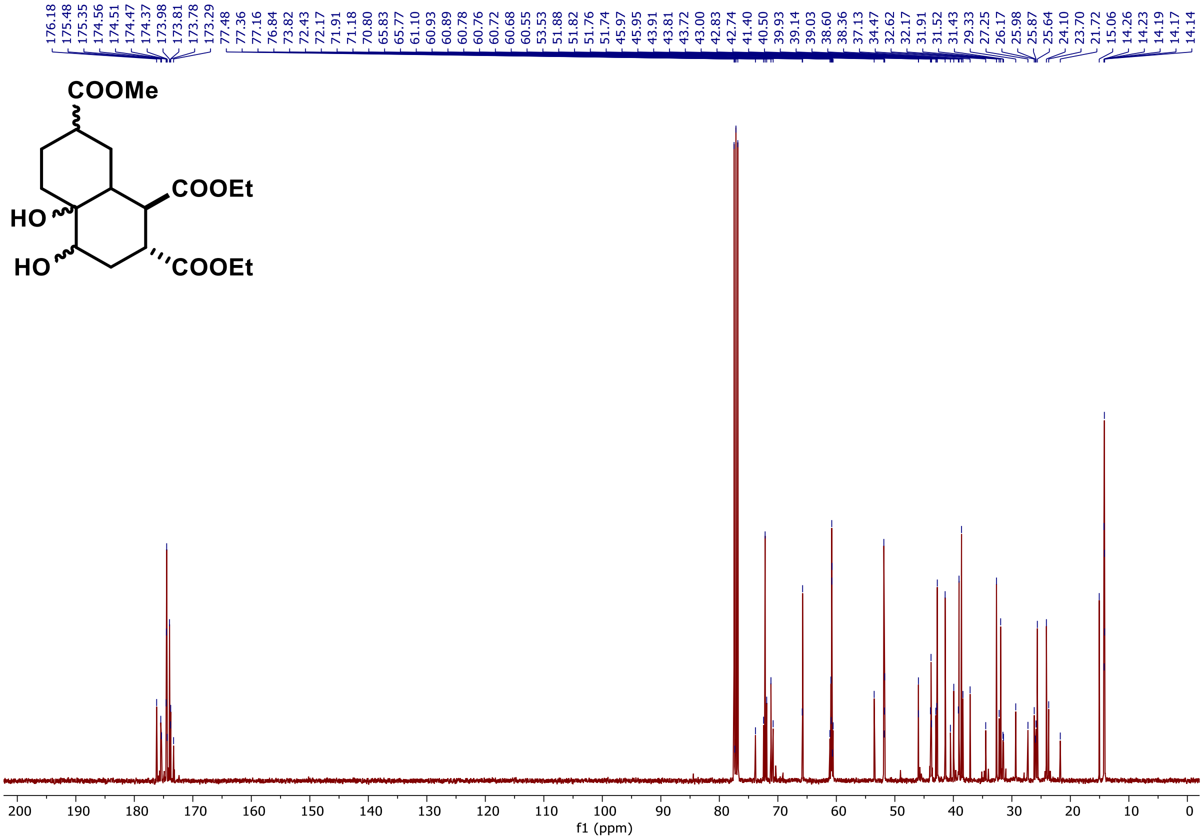

Figure S48: *^{13}C NMR spectrum of diol triester **18** (101 MHz, $CDCl_3$).*

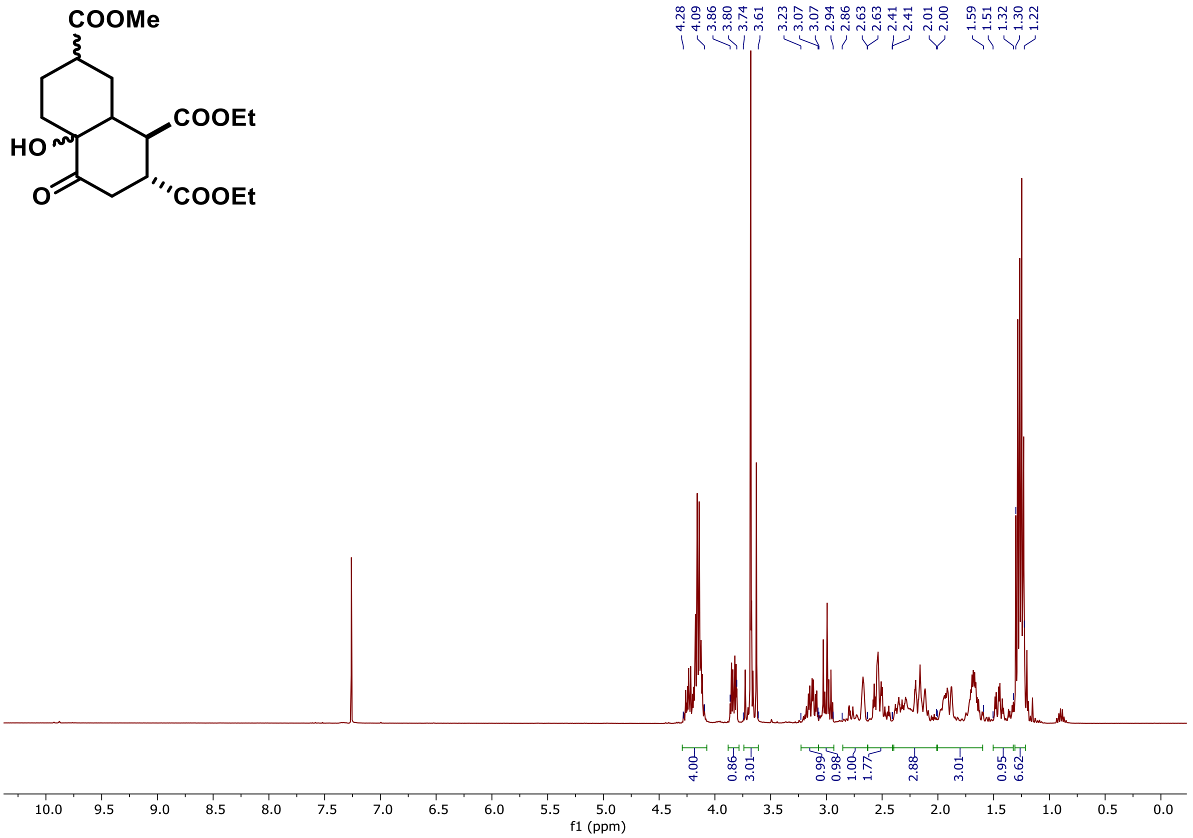

Figure S49: *^{1}H NMR spectrum of α-hydroxy ketone triester **19** (400 MHz, $CDCl_3$).*

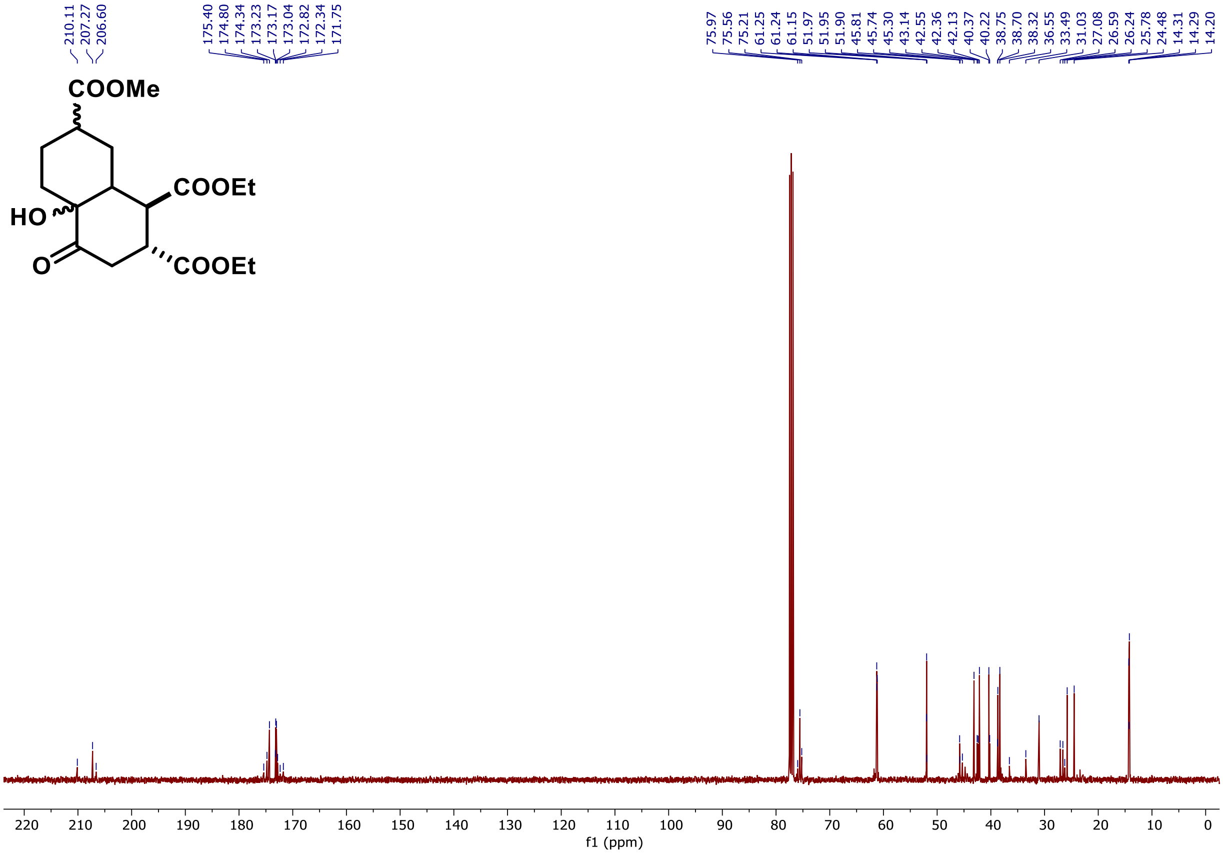

Figure S50: *^{13}C NMR spectrum of α-hydroxy ketone triester **19** (101 MHz, $CDCl_3$).*

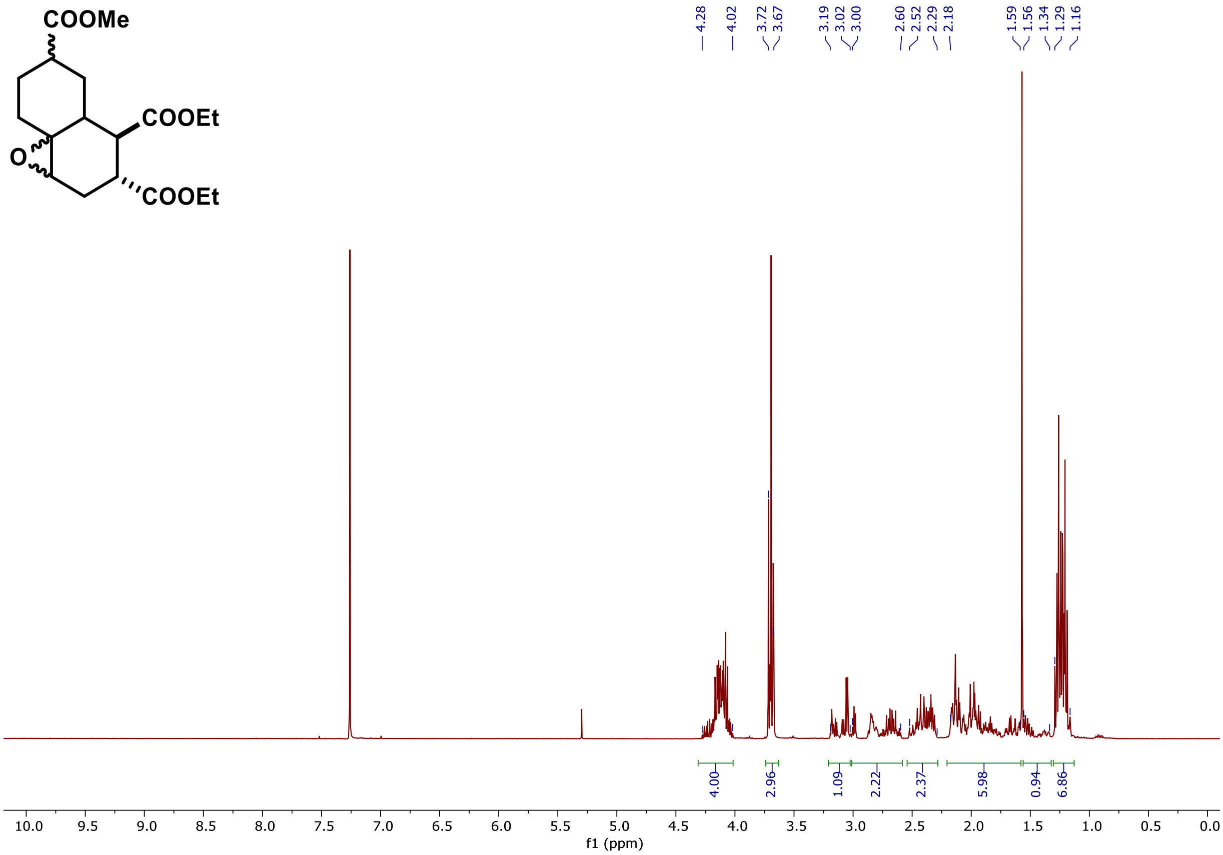

Figure S51: *^{1}H NMR spectrum of epoxide **20** (400 MHz, $CDCl_3$).*

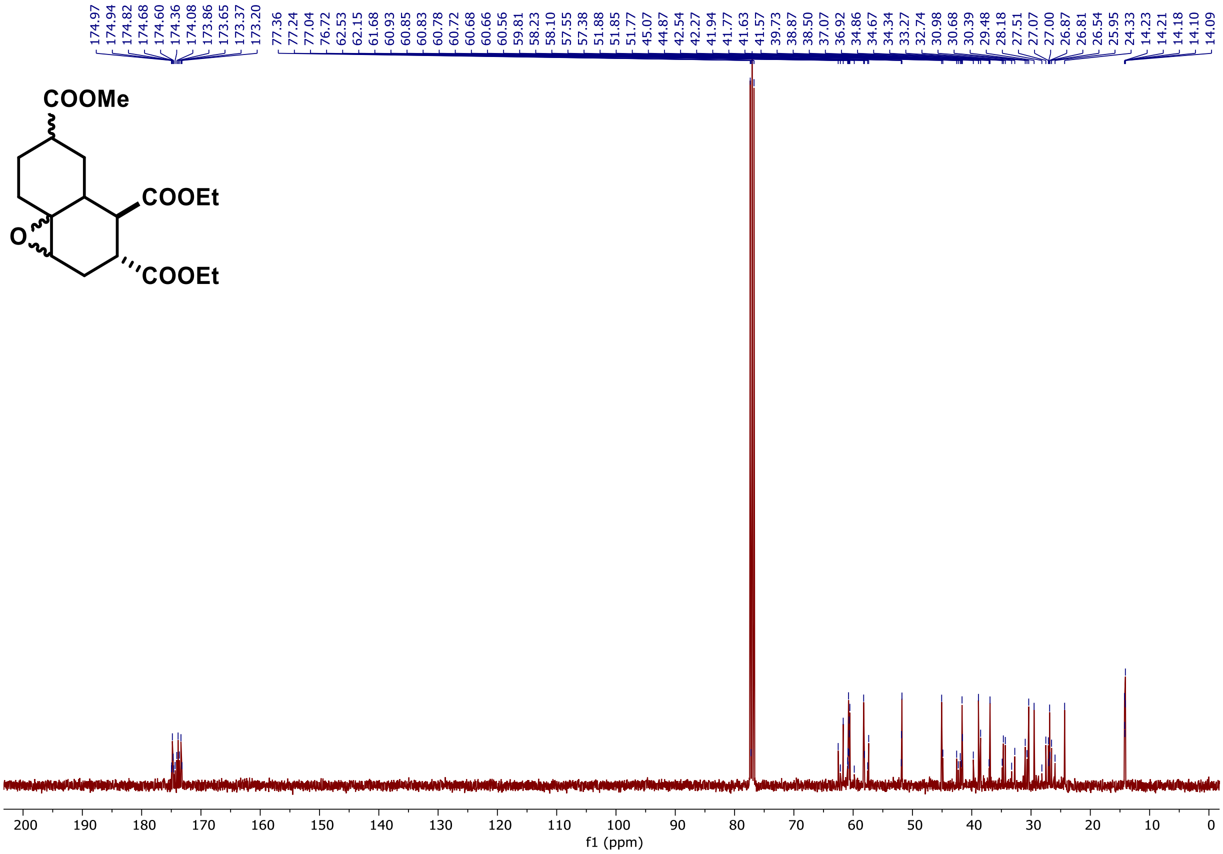

Figure S52: *^{13}C NMR spectrum of epoxide **20** (101 MHz, $CDCl_3$).*

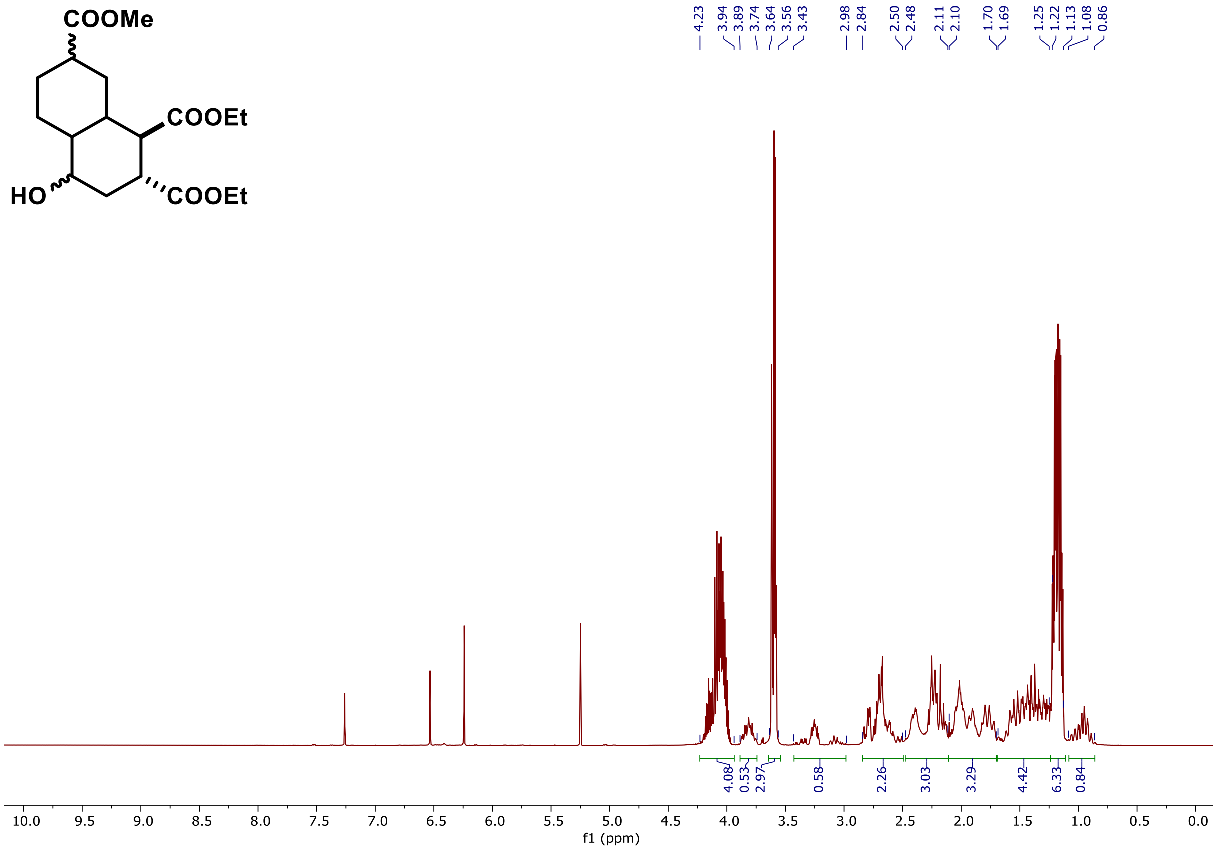

Figure S53: *^{1}H NMR spectrum of alcohol triester **21** (400 MHz, $CDCl_3$).*

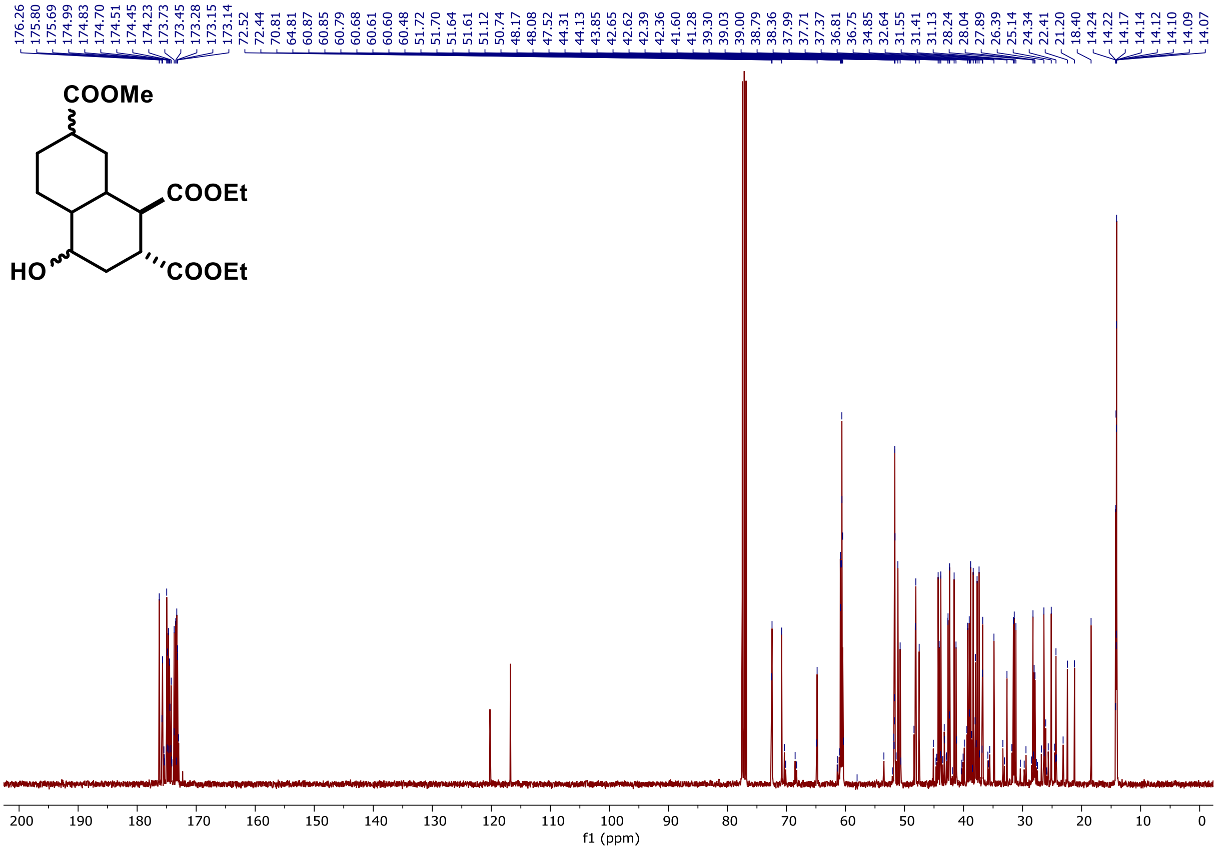

Figure S54: *^{13}C NMR spectrum of alcohol triester **21** (101 MHz, $CDCl_3$).*

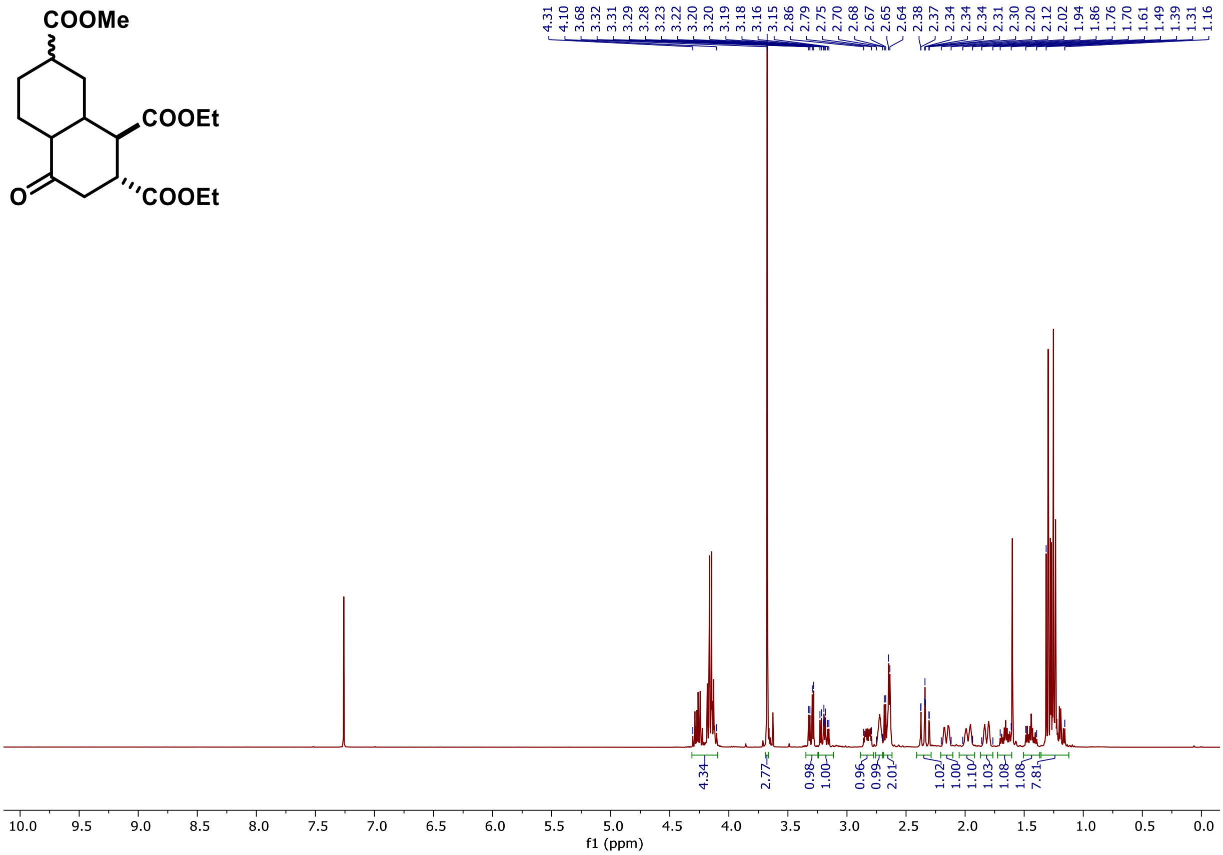

Figure S55: [1]*H NMR spectrum of ketone triester* ***22*** *single isomer (400 MHz, $CDCl_3$).*

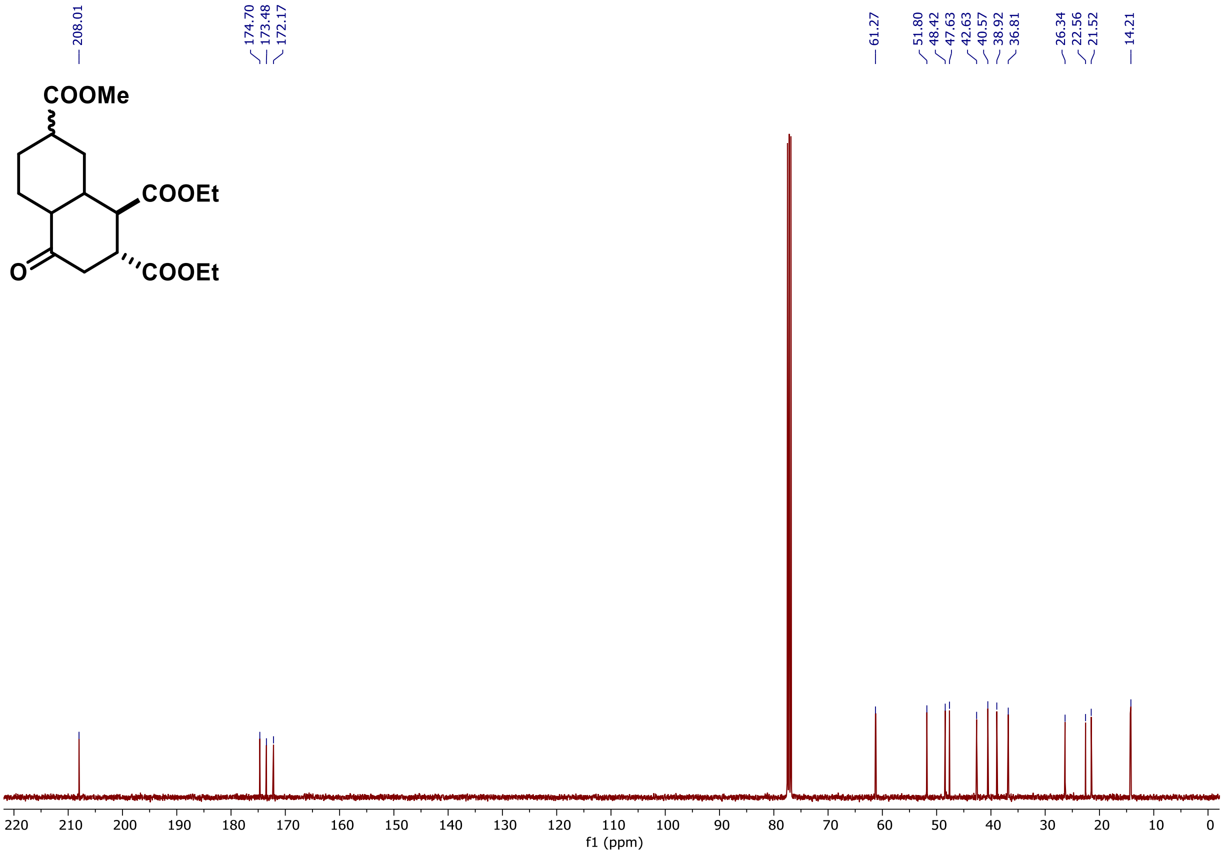

Figure S56: [13]*C NMR spectrum of ketone triester* ***22*** *single isomer (101 MHz, $CDCl_3$).*

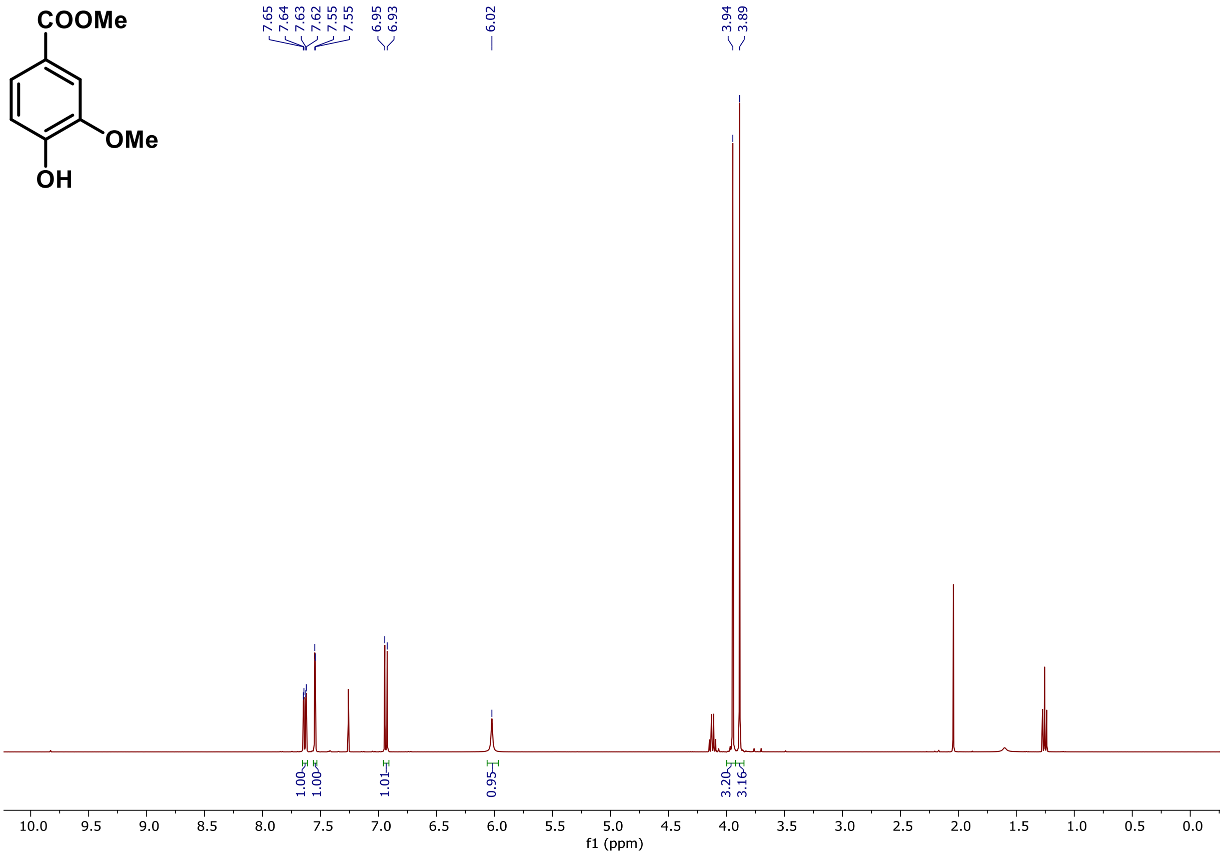

Figure S57: *^{1}H NMR spectrum of ester **24** (400 MHz, $CDCl_3$).*

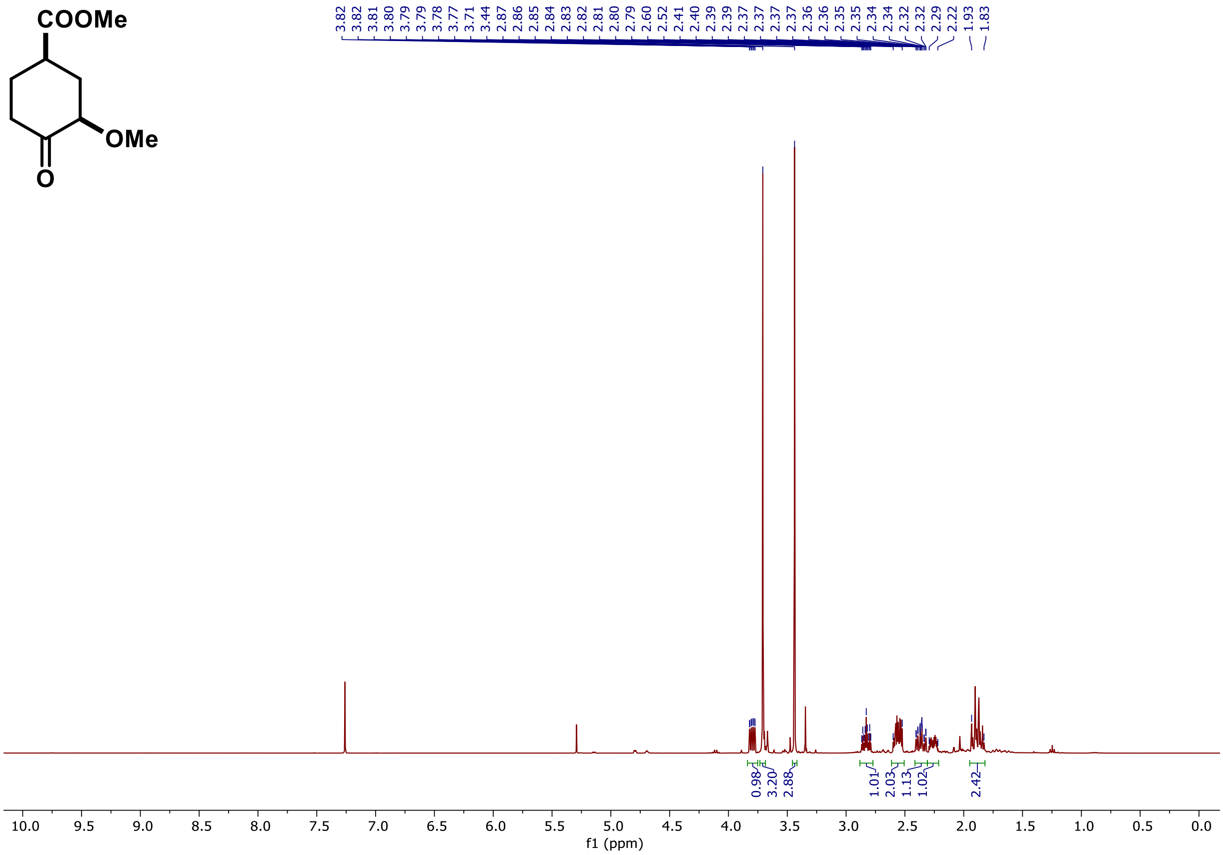

Figure S58: *^{1}H NMR spectrum of α-methoxy ketone* ***26*** *(400 MHz, $CDCl_3$).*

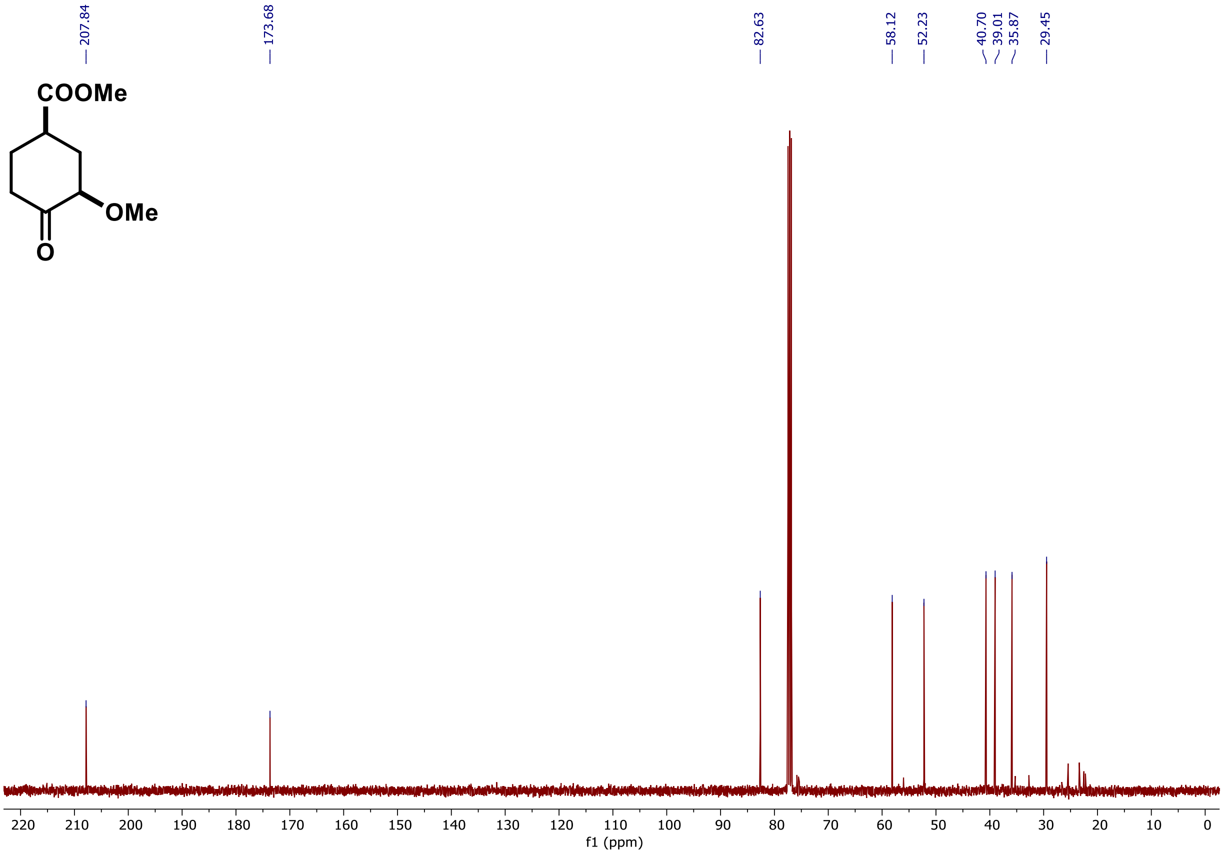

Figure S59: *^{13}C NMR spectrum of α-methoxy ketone* ***26*** *(101 MHz, $CDCl_3$).*

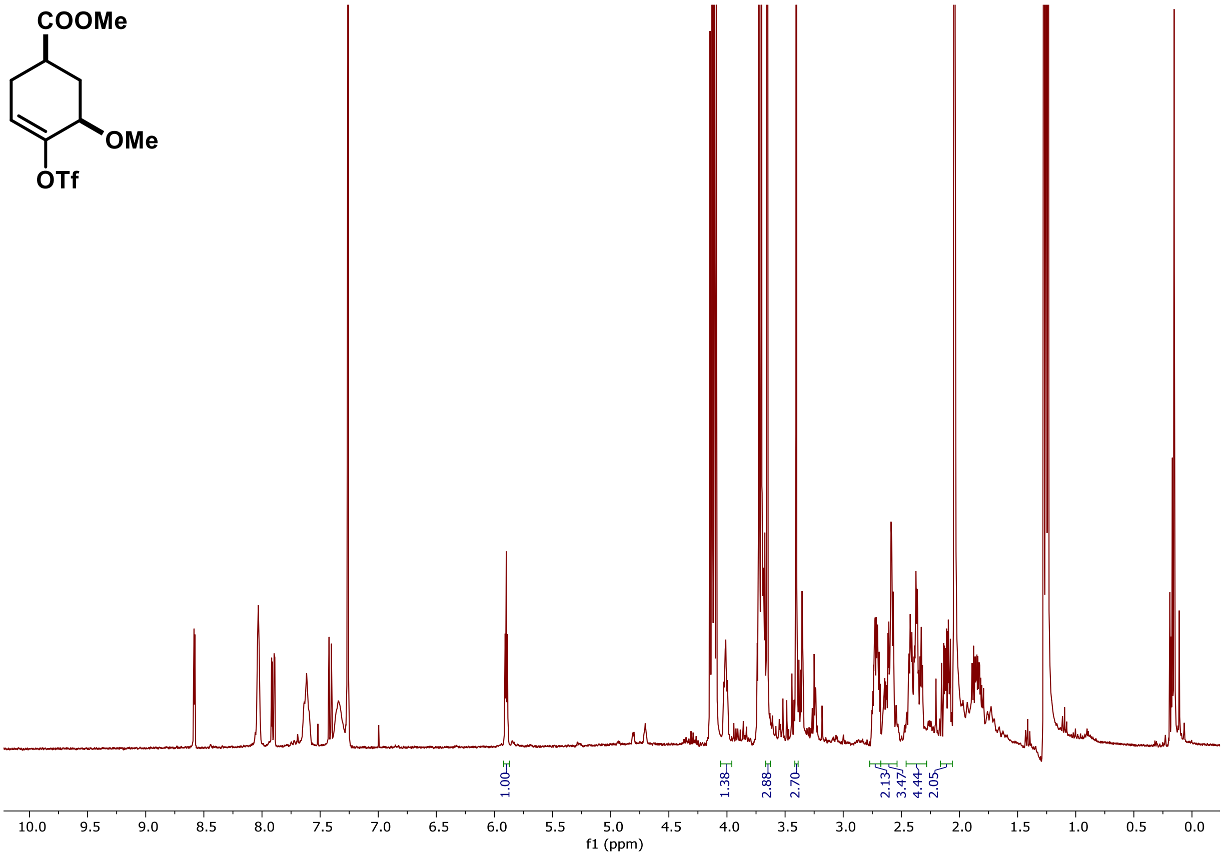

Figure S60: *^{1}H NMR spectrum of crude mixture of triflate* ***27*** *(400 MHz, $CDCl_3$).*

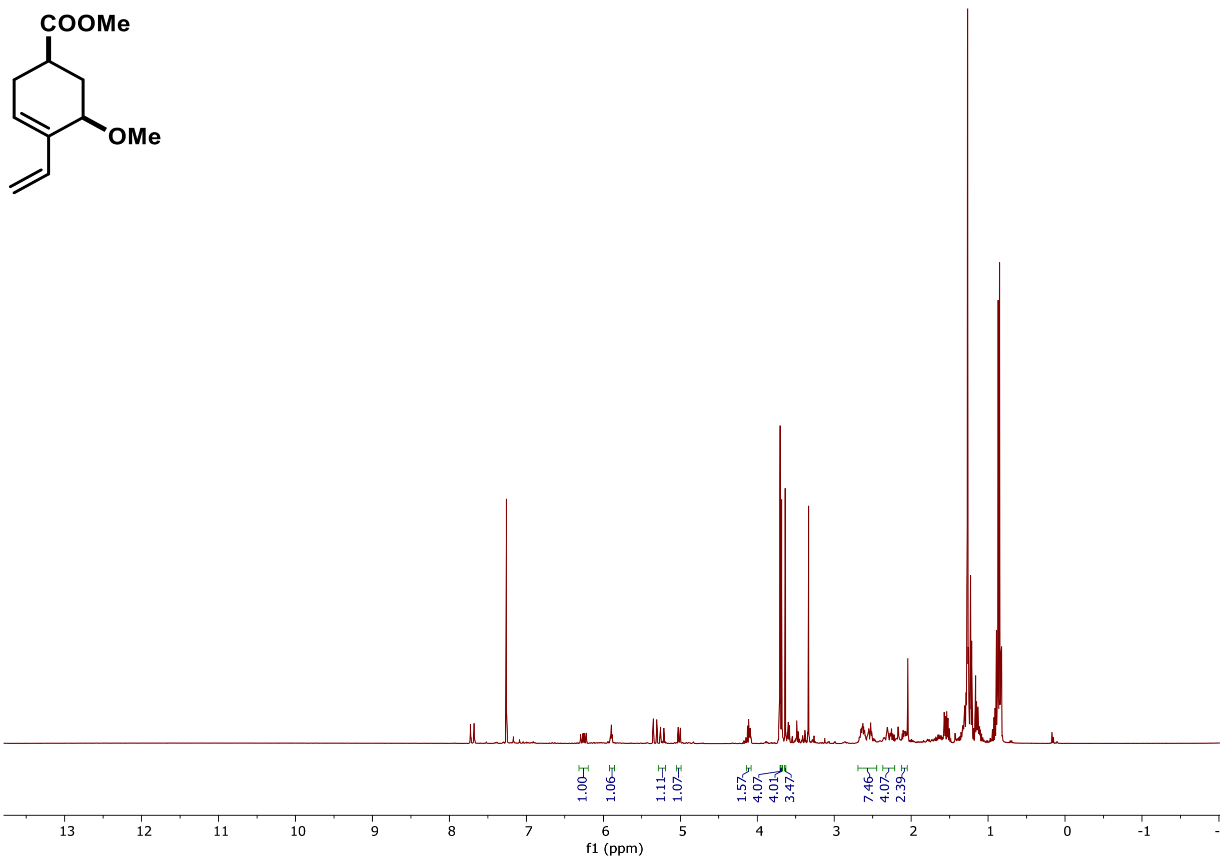

Figure S61: *^{1}H NMR spectrum of fractions containing diene* ***29*** *from Suzuki reaction (400 MHz, $CDCl_3$).*

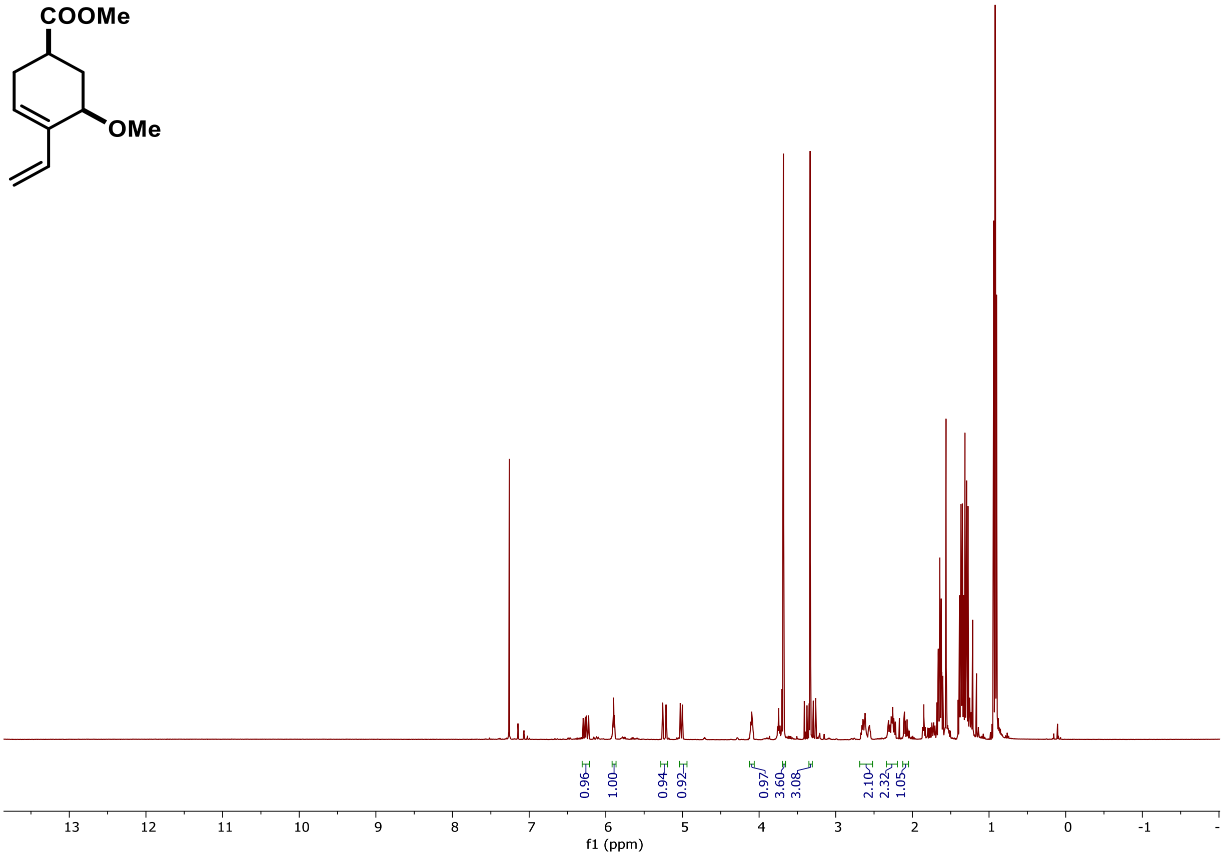

Figure S62: *^{1}H NMR spectrum of fractions containing diene* ***29*** *from Stille reaction (400 MHz, $CDCl_3$).*

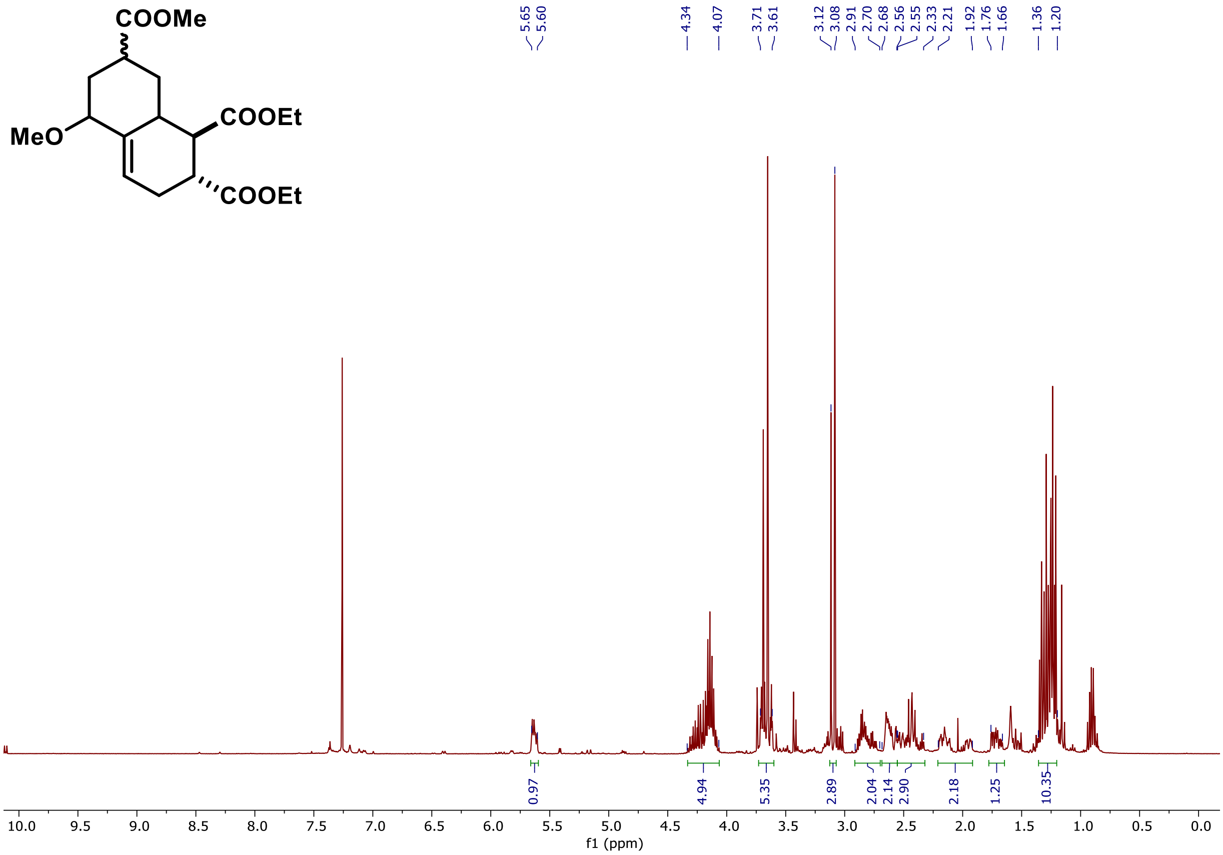

Figure S63: *^{1}H NMR spectrum of ether alkene triester* ***31*** *(400 MHz, $CDCl_3$).*

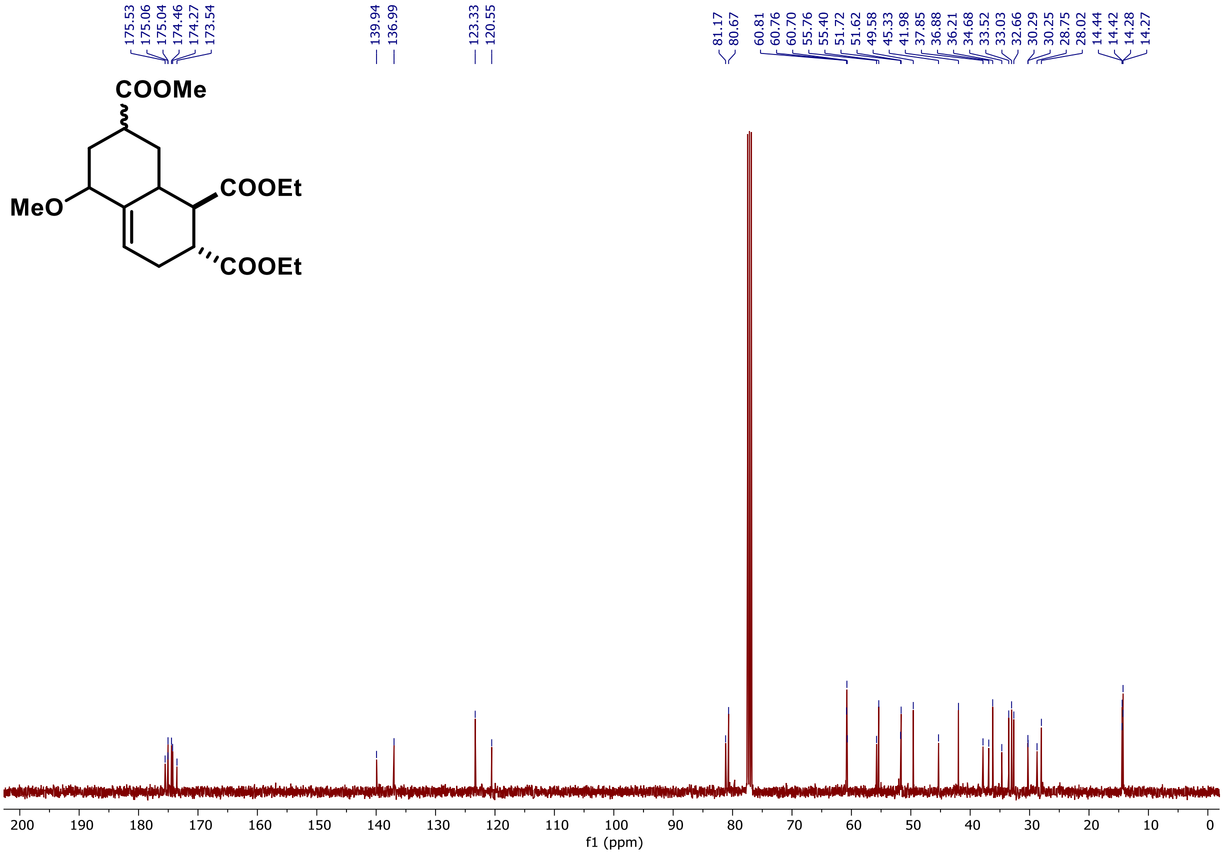

Figure S64: *^{13}C NMR spectrum of ether alkene triester* ***31*** *(101 MHz, $CDCl_3$).*

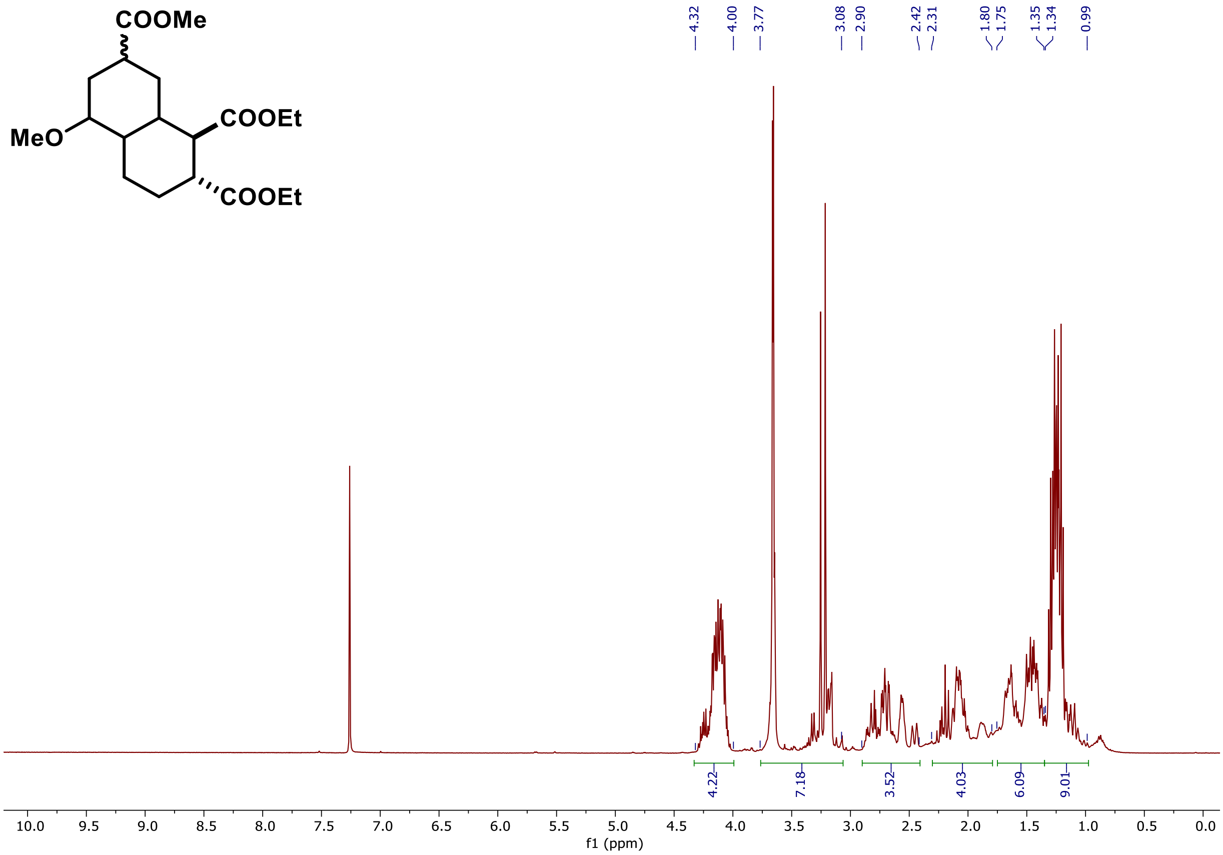

Figure S65: *^{1}H NMR spectrum of ether alkane triester* ***32****, contaminated with triester alkane* ***33*** *(400 MHz, $CDCl_3$).*

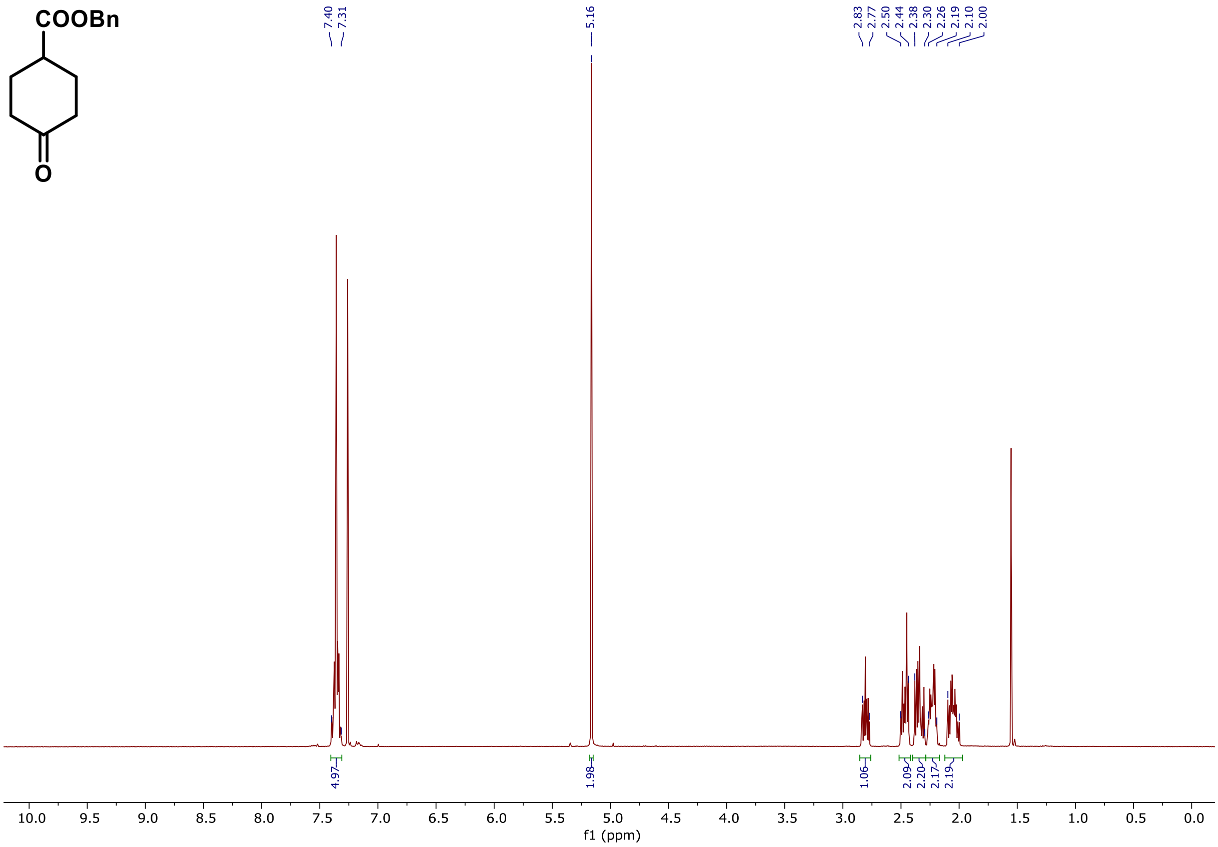

Figure S66: *^{1}H NMR spectrum of benzyl ester* ***37*** *(400 MHz, $CDCl_3$).*

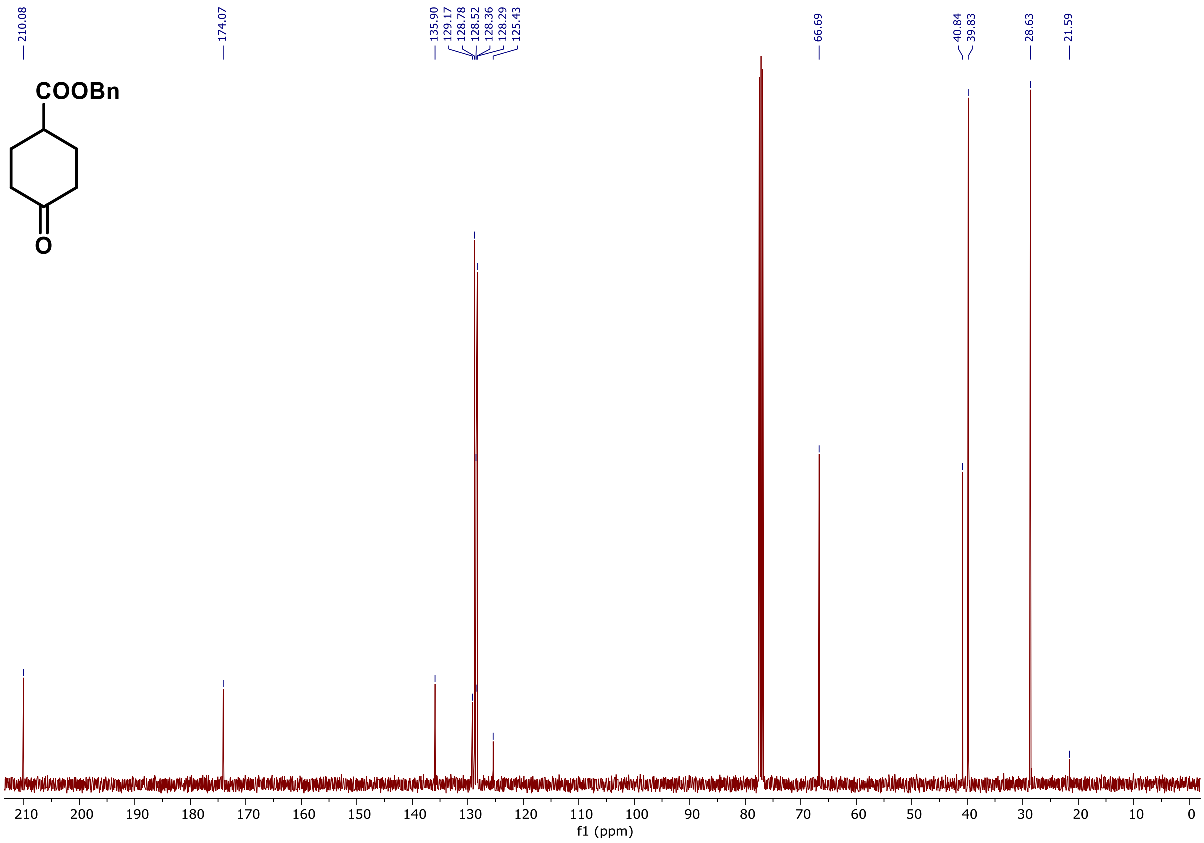

Figure S67: *^{13}C NMR spectrum of benzyl ester* ***37*** *(101 MHz, $CDCl_3$)*

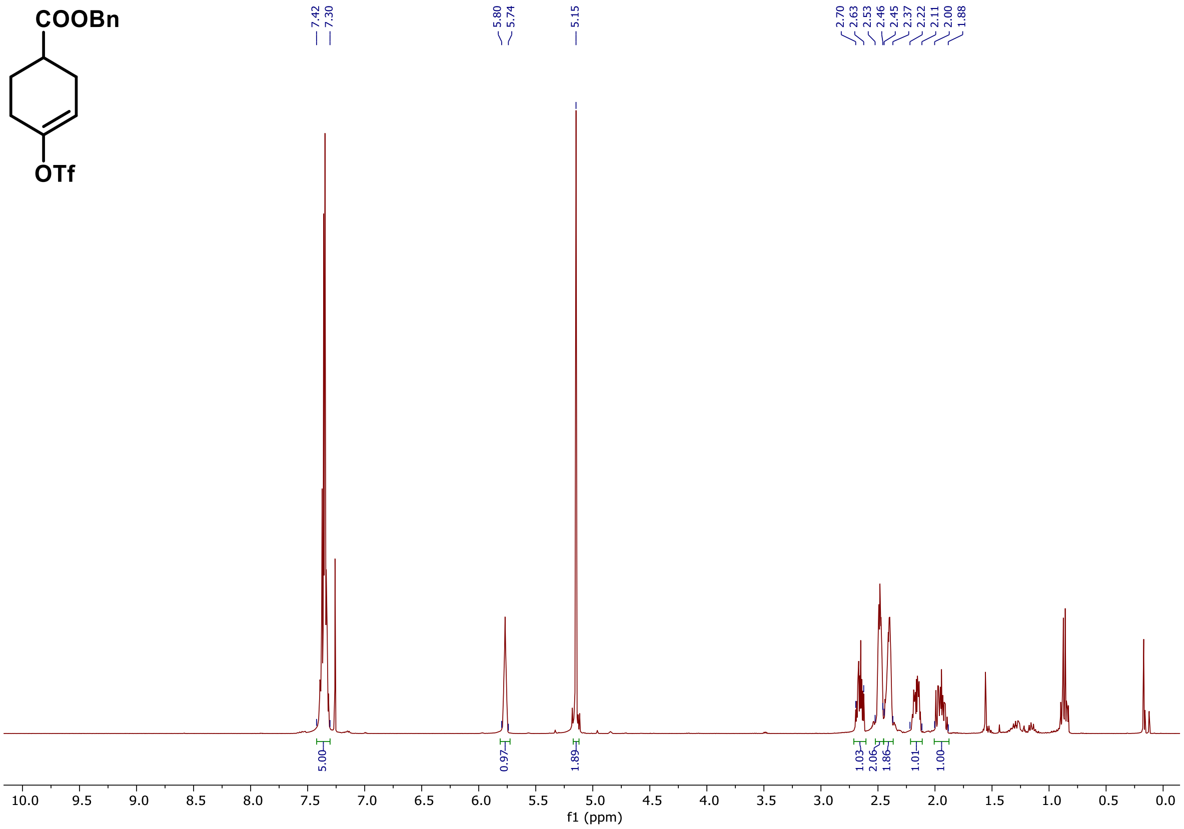

Figure S68: *^{1}H NMR spectrum of triflate* ***38*** *(400 MHz, $CDCl_3$).*

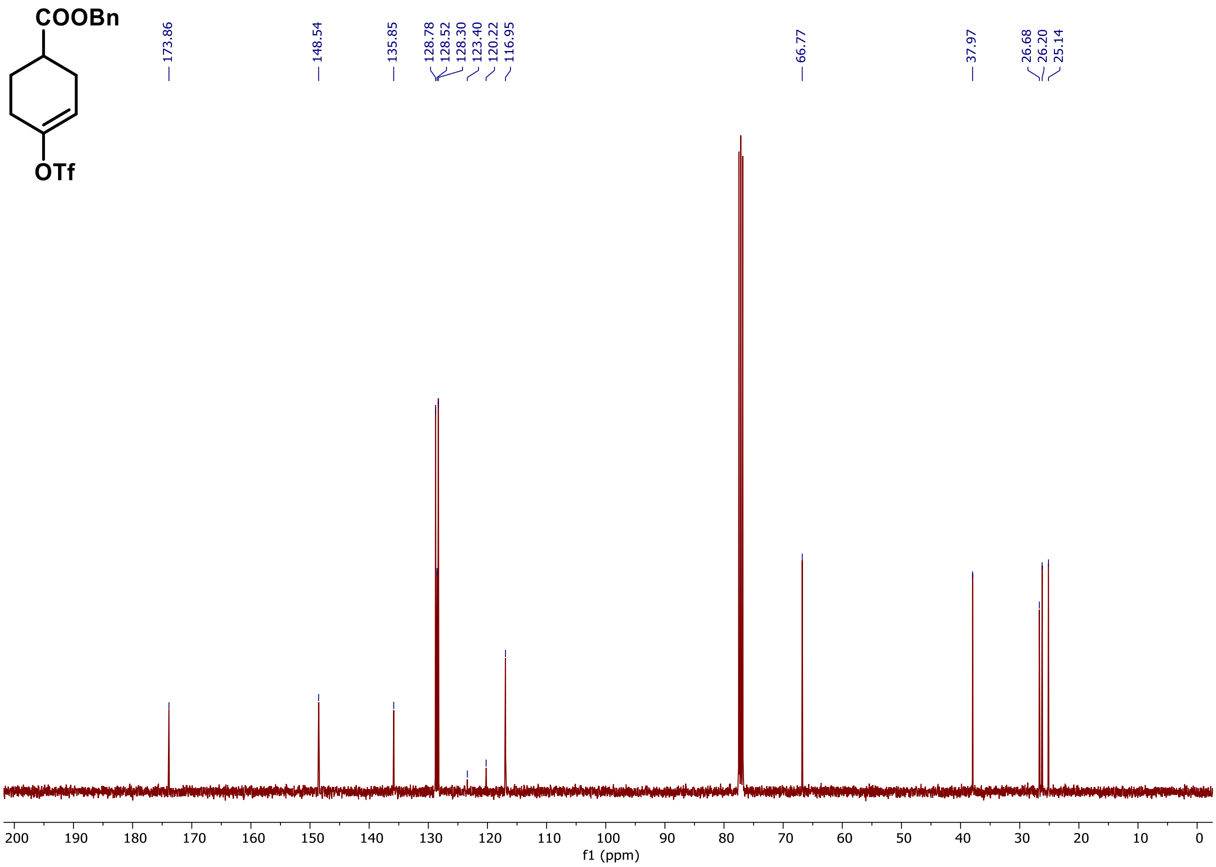

Figure S69: *^{13}C NMR spectrum of triflate* ***38*** *(101 MHz, $CDCl_3$)*

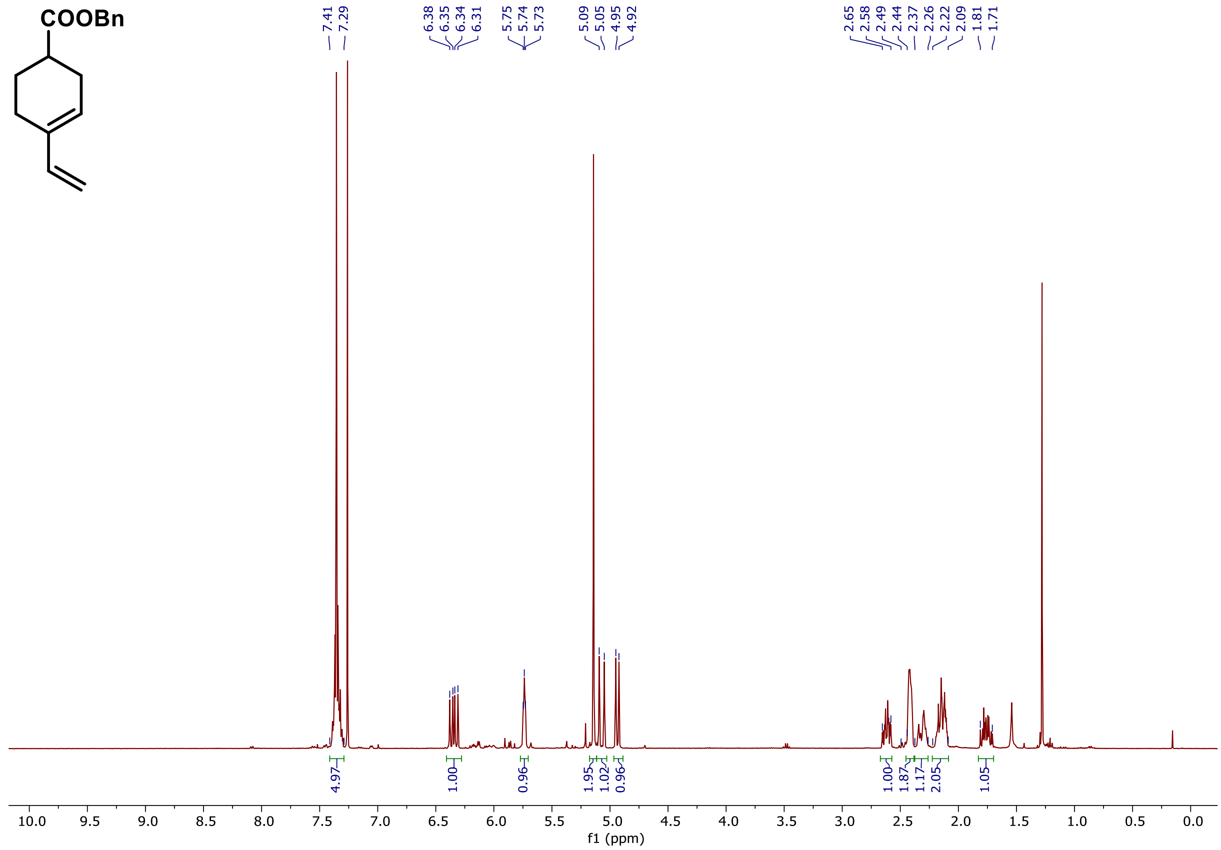

Figure S70: *^{1}H NMR spectrum of diene* ***39*** *(400 MHz, $CDCl_3$).*

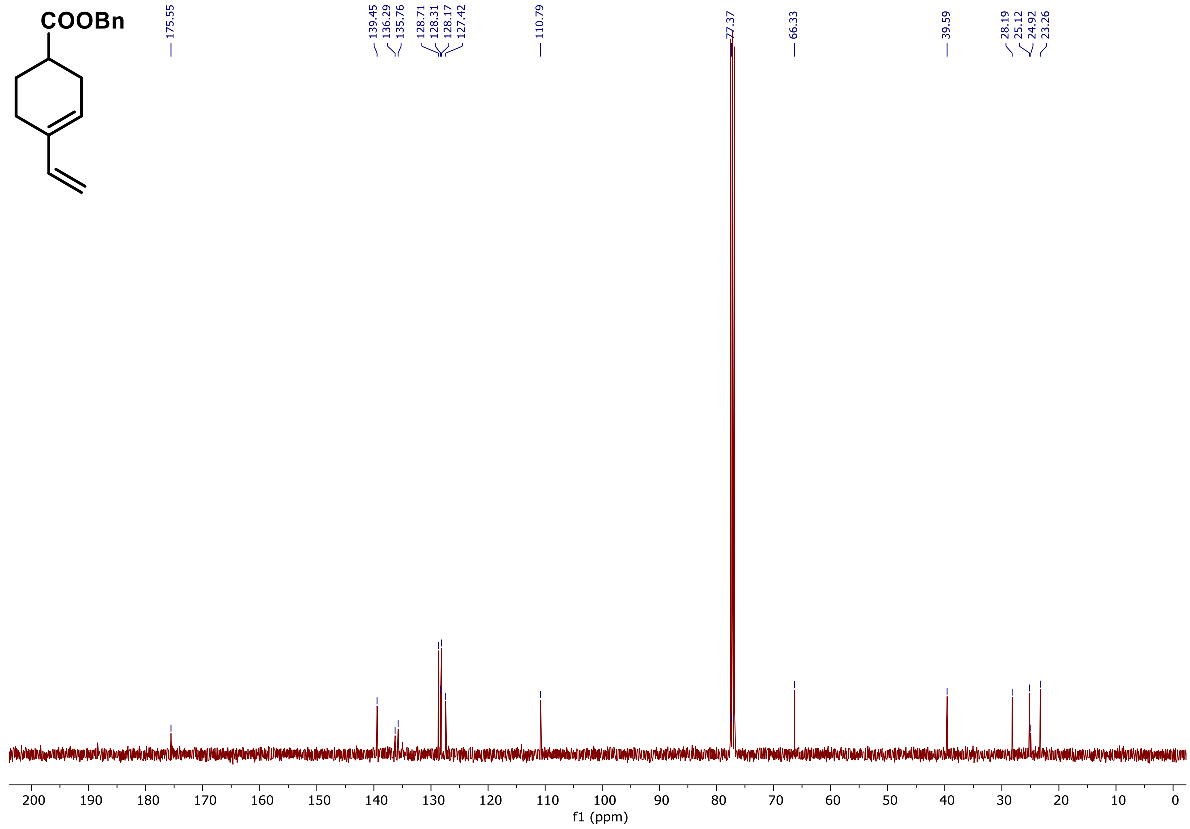

Figure S71: *^{13}C NMR spectrum of diene* ***39*** *(101 MHz, $CDCl_3$)*

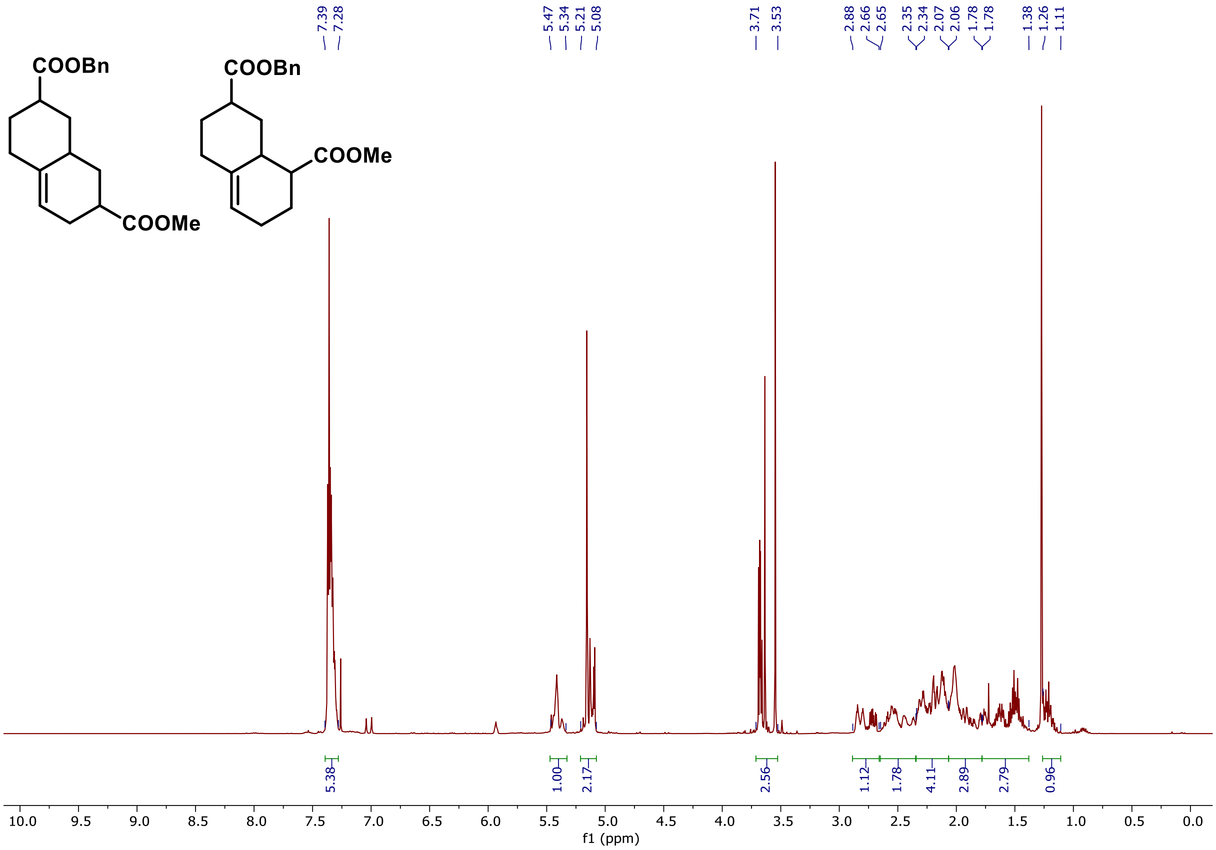

Figure S72: *^{1}H NMR spectrum of diesters **41a** and **41b** (400 MHz, $CDCl_3$).*

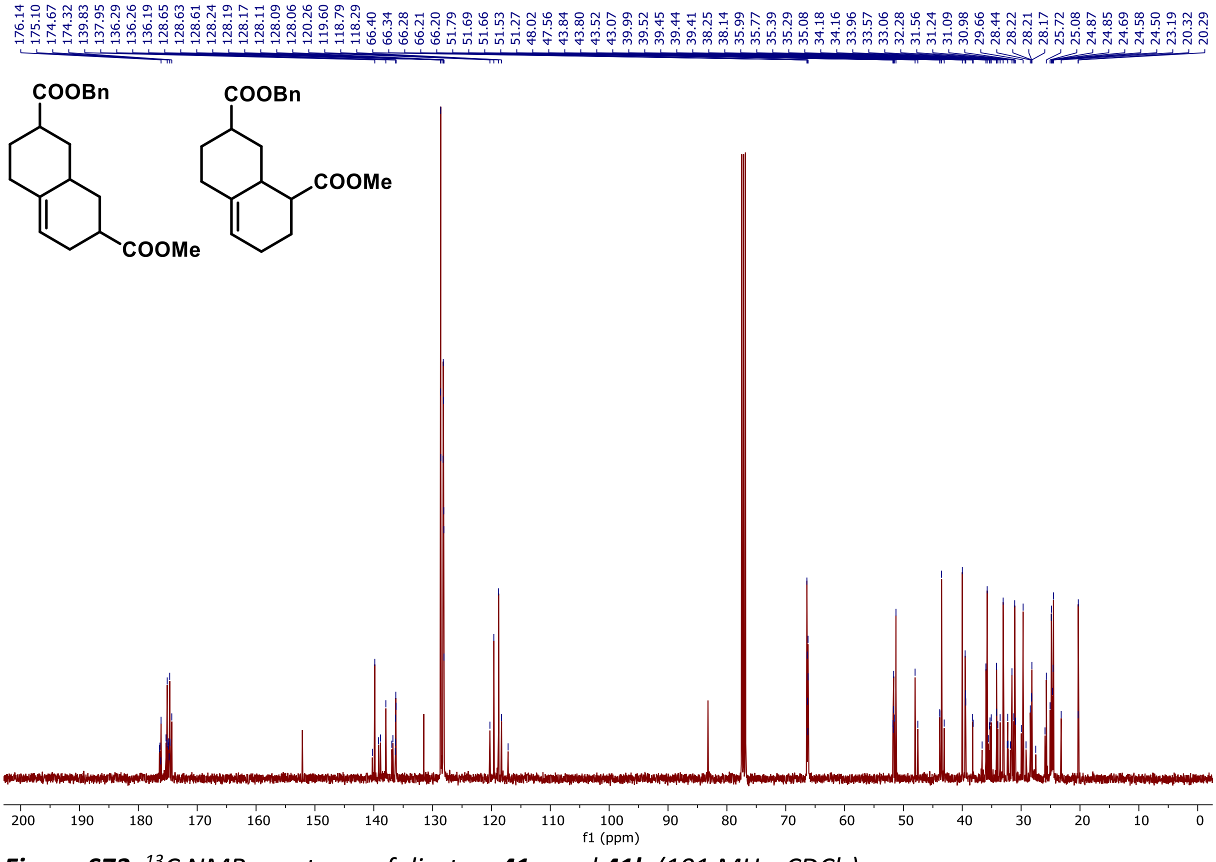

Figure S73: *^{13}C NMR spectrum of diesters **41a** and **41b** (101 MHz, $CDCl_3$).*

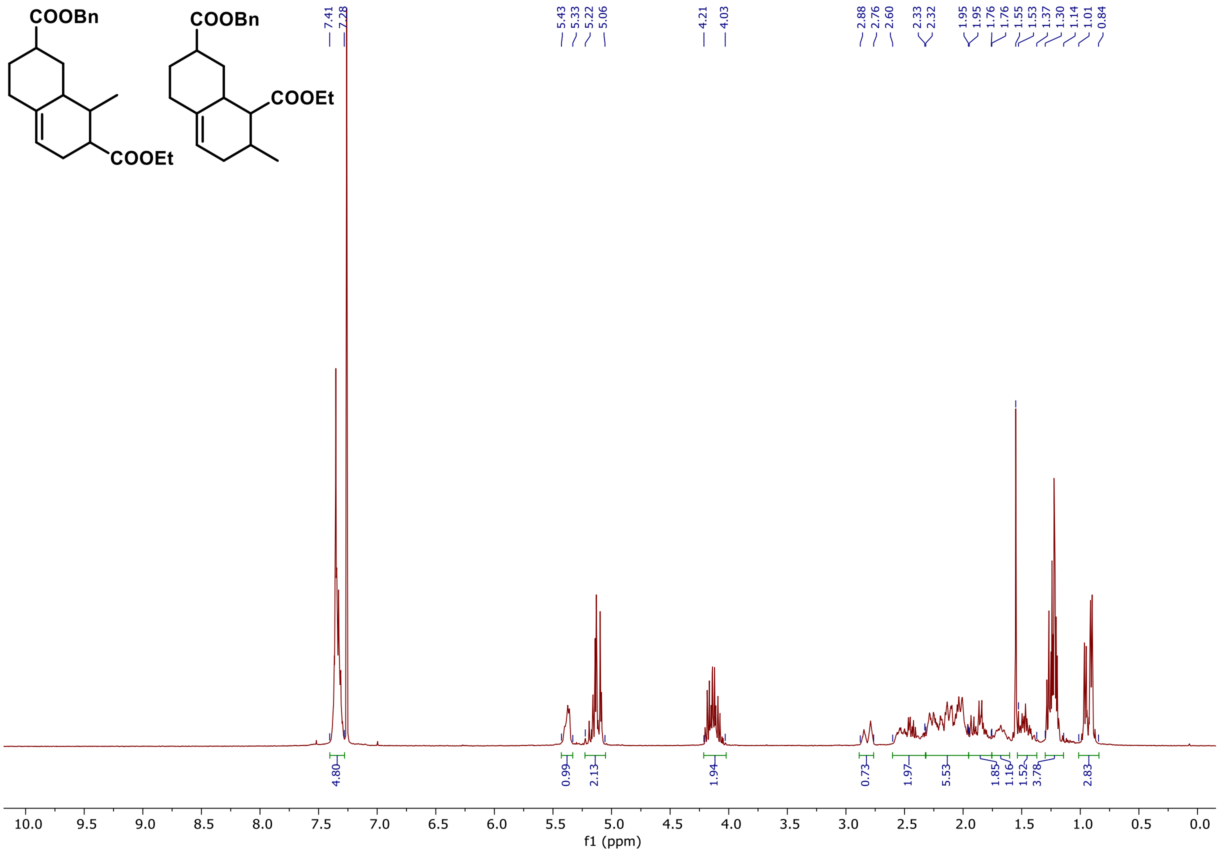

Figure S74: *^{1}H NMR spectrum of methyl diesters **43a** and **43b** (400 MHz, $CDCl_3$).*

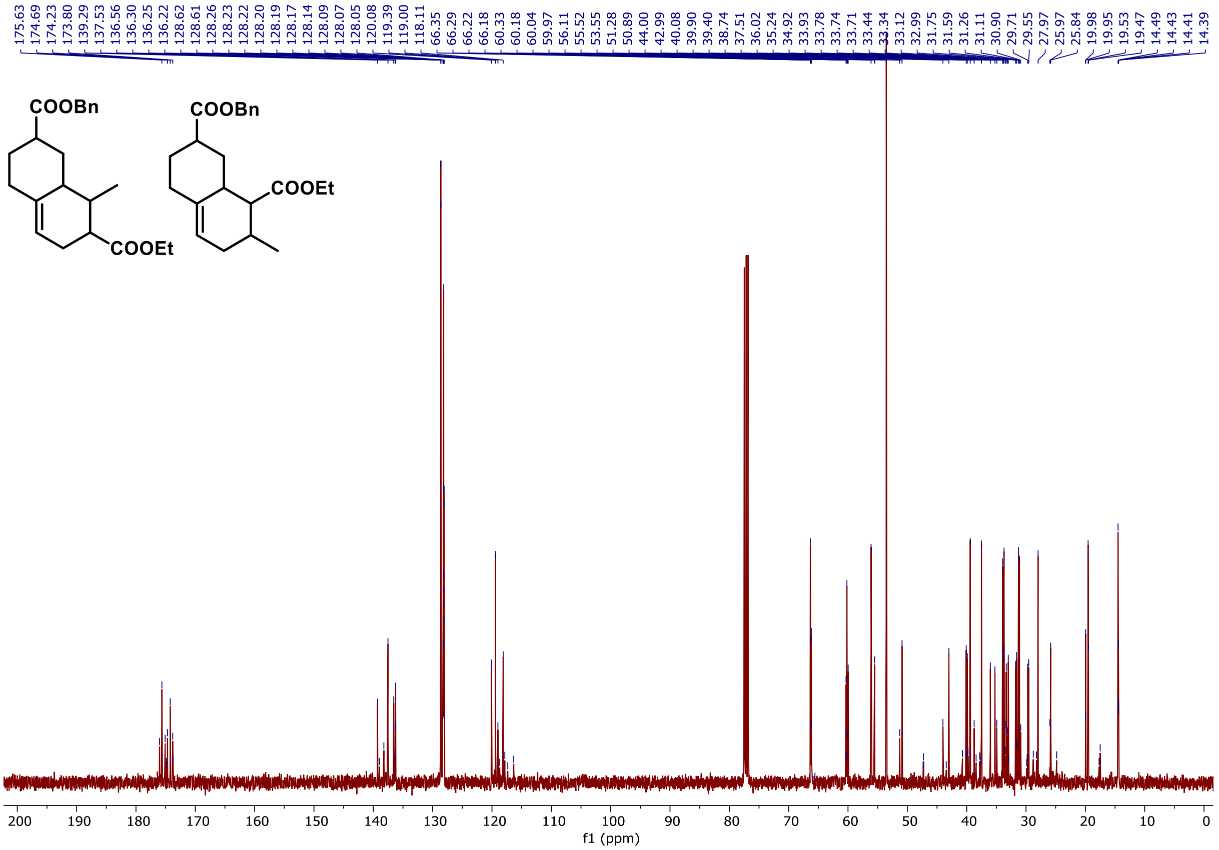

Figure S75: *^{13}C NMR spectrum of methyl diesters **43a** and **43b** (101 MHz, $CDCl_3$).*

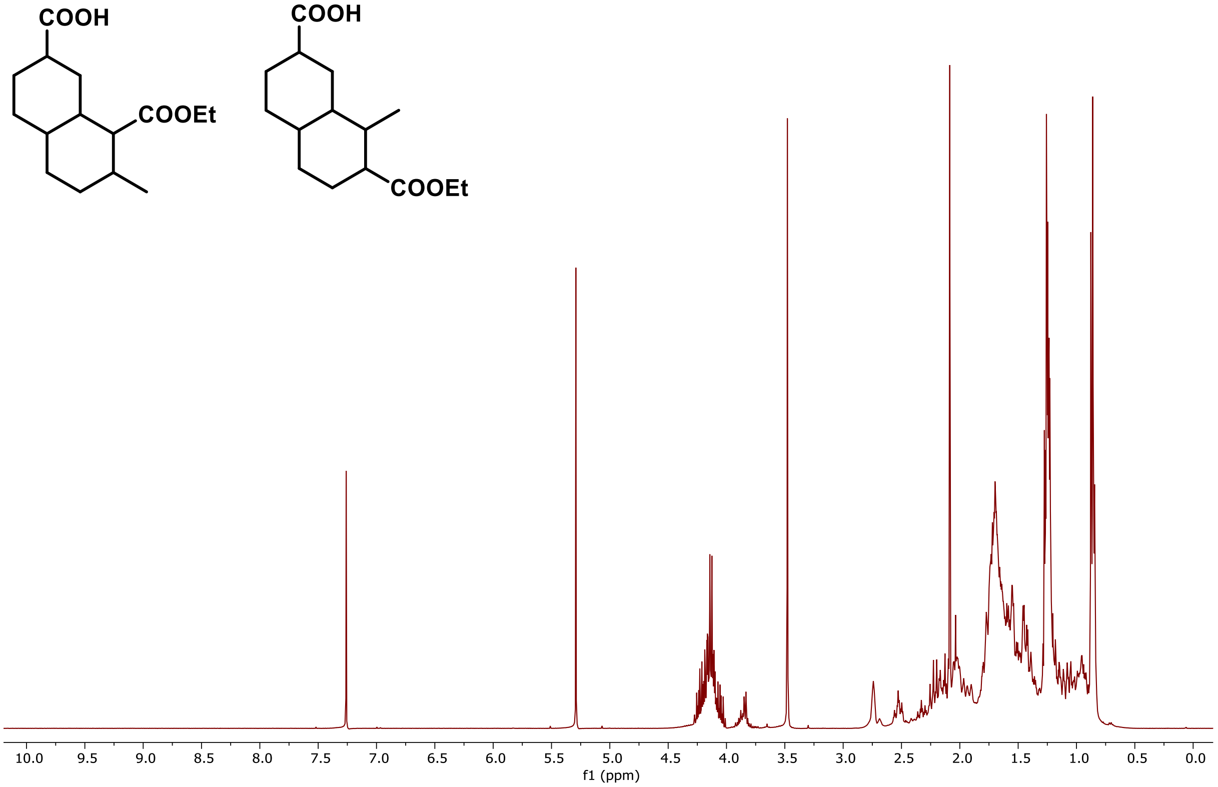

Figure S76: *Crude* ^{1}*H NMR spectrum of alkane esters* ***44a*** *and* ***44b*** *(400 MHz,* $CDCl_3$*).*

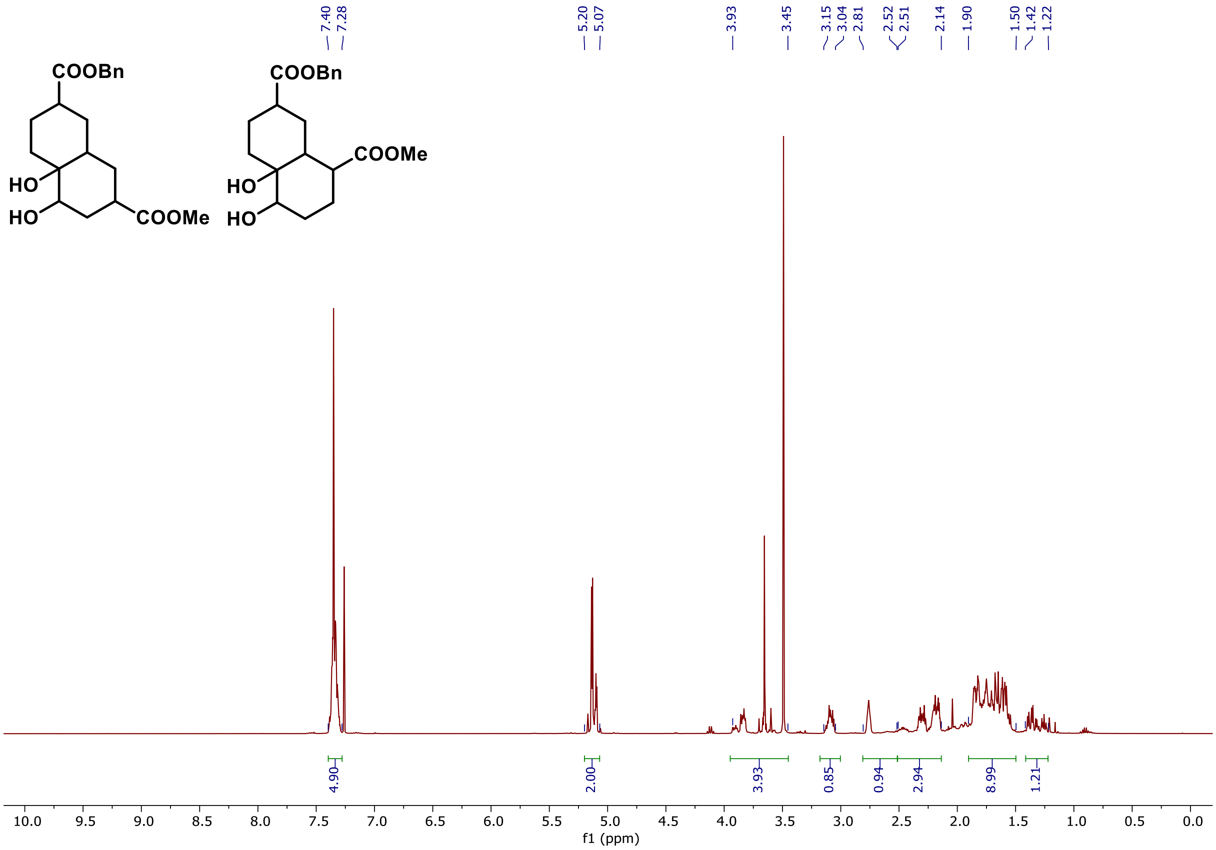

Figure S77: *^{1}H NMR spectrum of diols* ***45a*** *and* ***45b*** *(400 MHz, CDCl$_3$).*

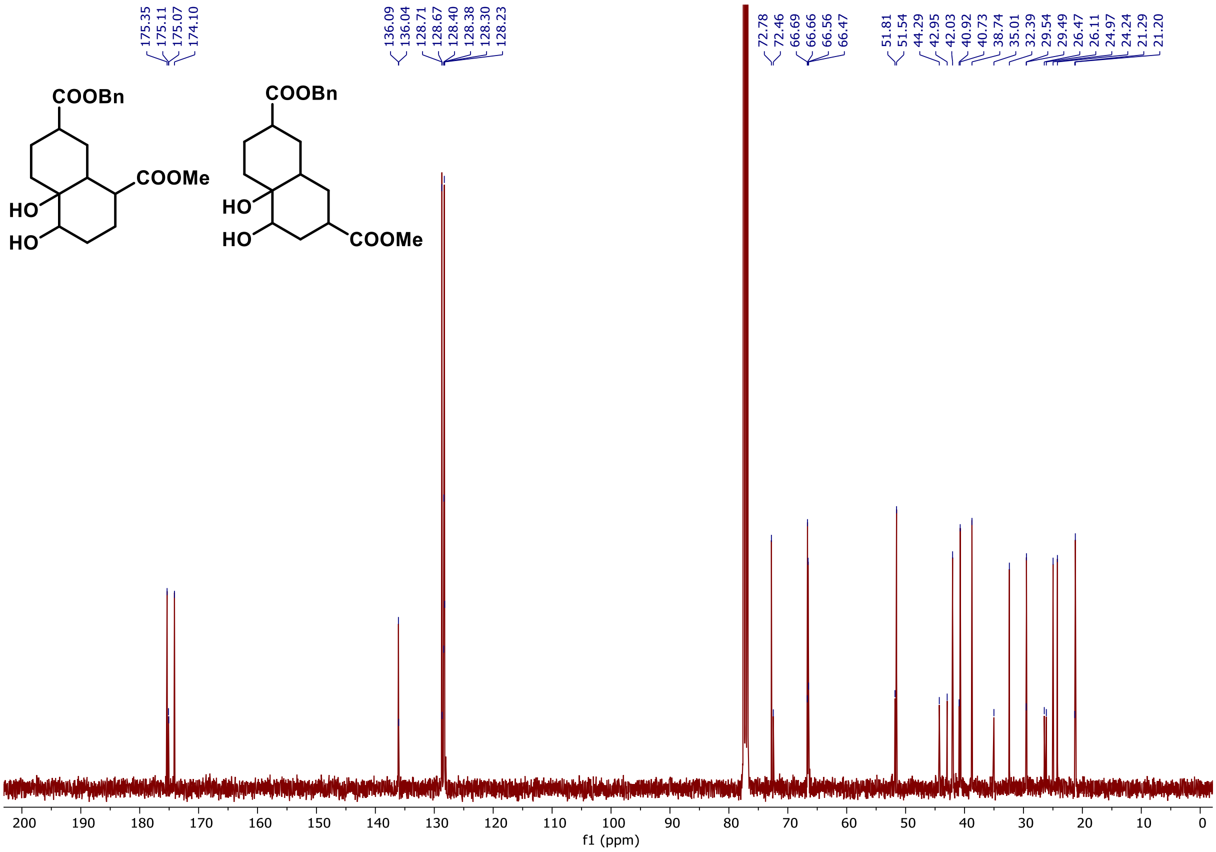

Figure 78: *^{13}C NMR spectrum of diols* ***45a*** *and* ***45b*** *(101 MHz, CDCl$_3$).*

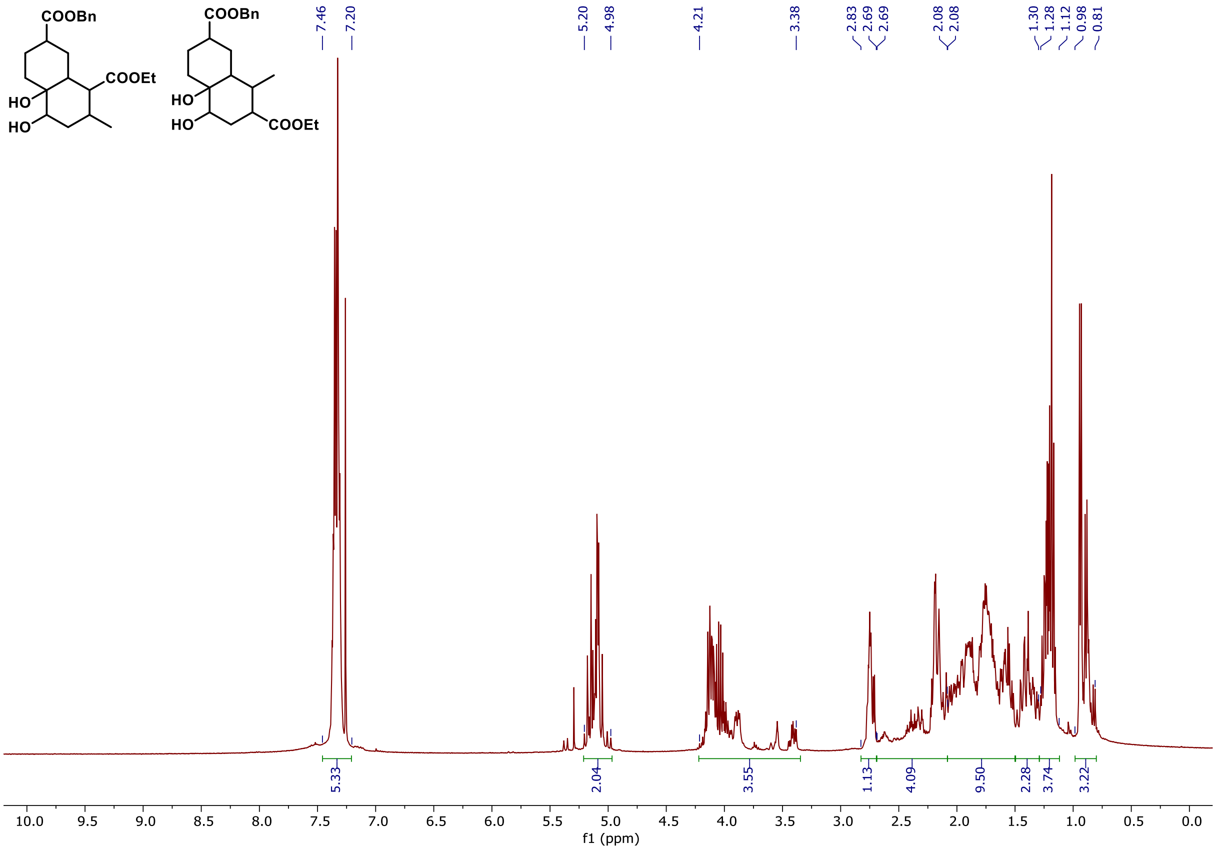

Figure S79: *^{1}H NMR spectrum of diols **46a** and **46b** (400 MHz, $CDCl_3$).*

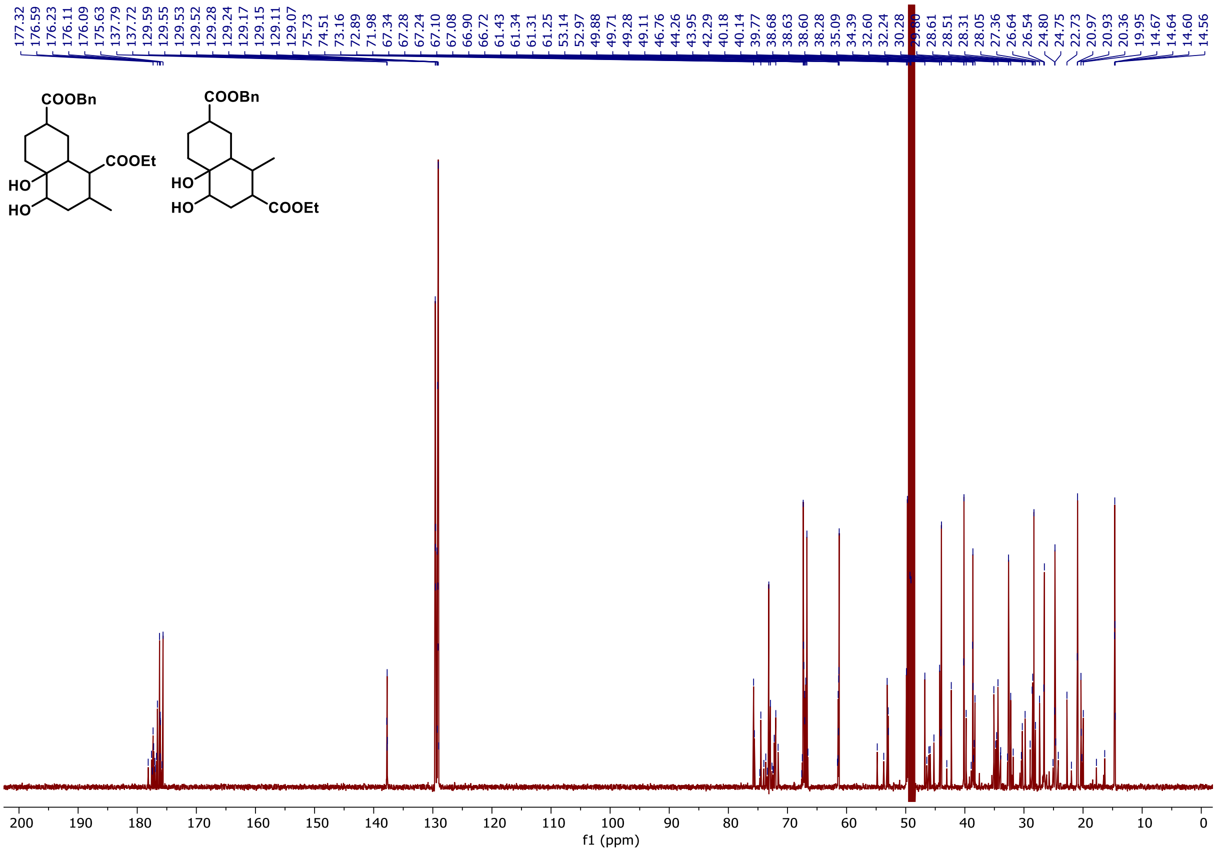

Figure S80: *^{13}C NMR spectrum of diols **46a** and **46b** (126 MHz, MeOD).*

NMR, MS1, and CAD Data for Compounds 5-16

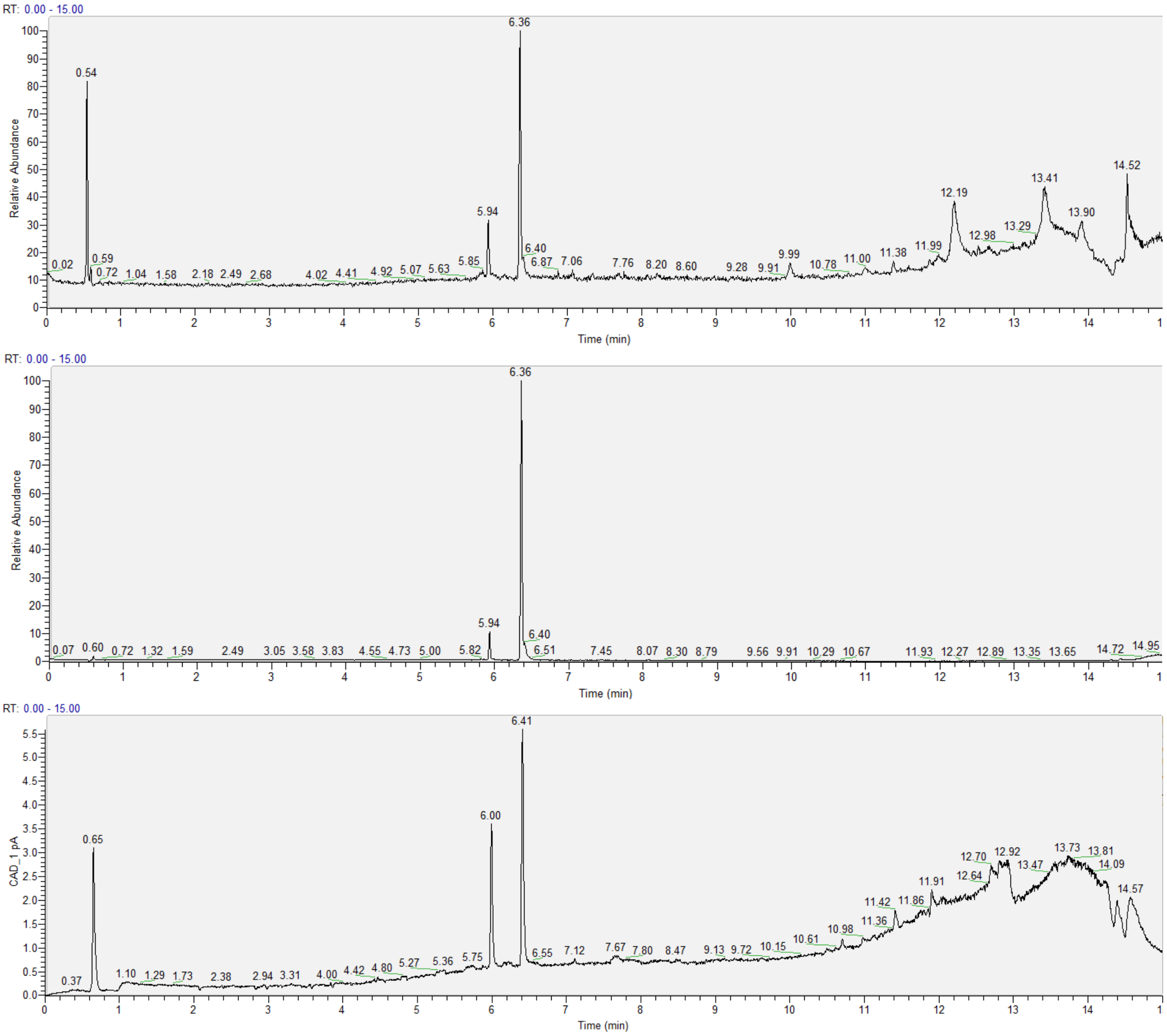

Figure S81: *TIC trace (top), XIC of title compound parent ion (middle), and CAD trace (bottom) of CRAM diacid* ***5****.*

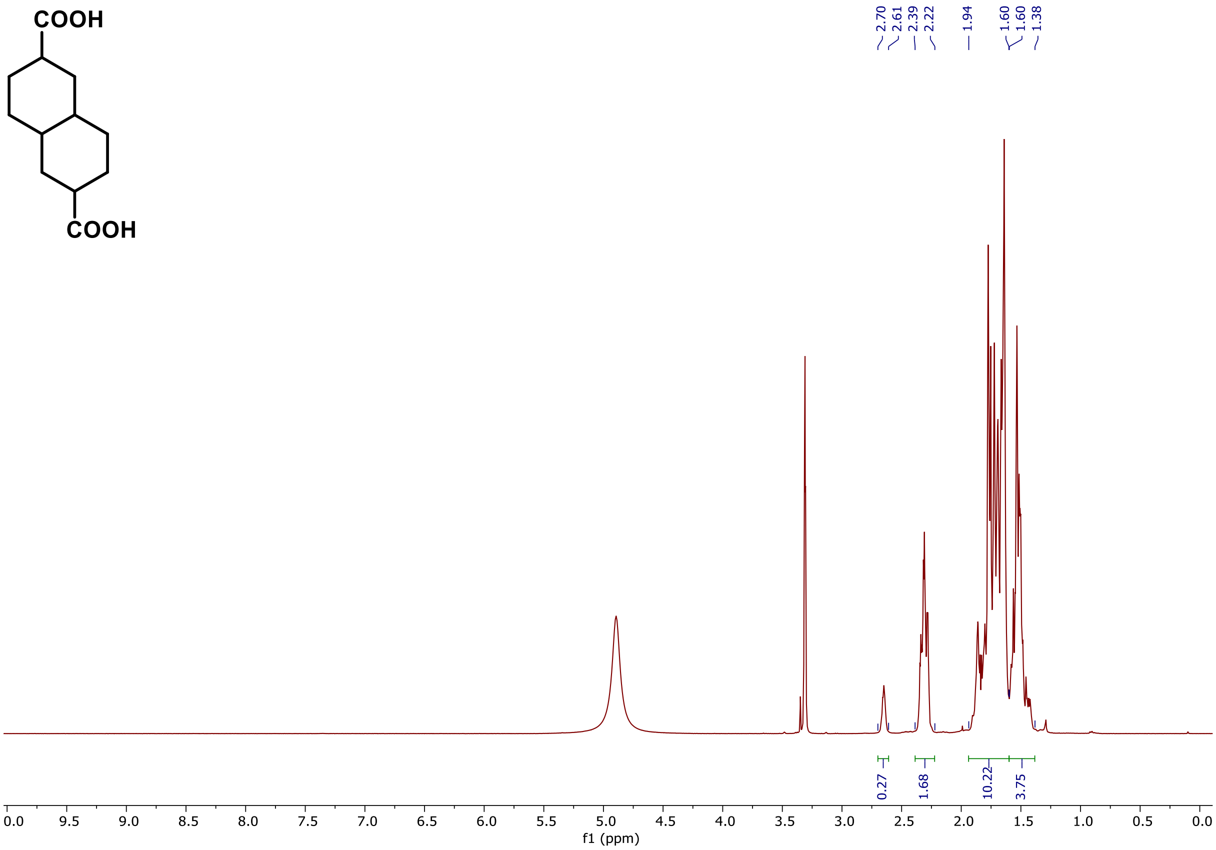

Figure S82: *¹H NMR spectrum of diacid* ***5*** *(400 MHz, MeOD).*

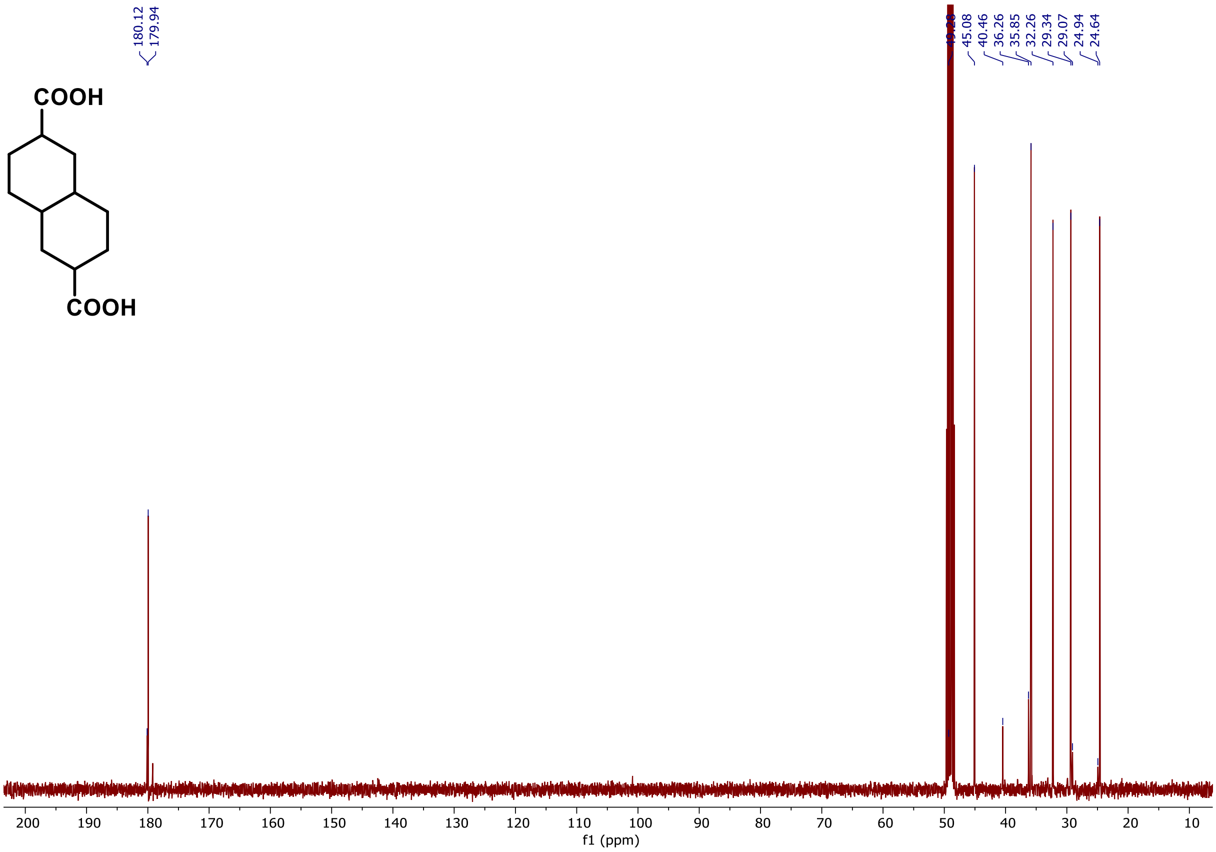

Figure S83: *¹³C NMR spectrum of diacid* ***5*** *(101 MHz, MeOD).*

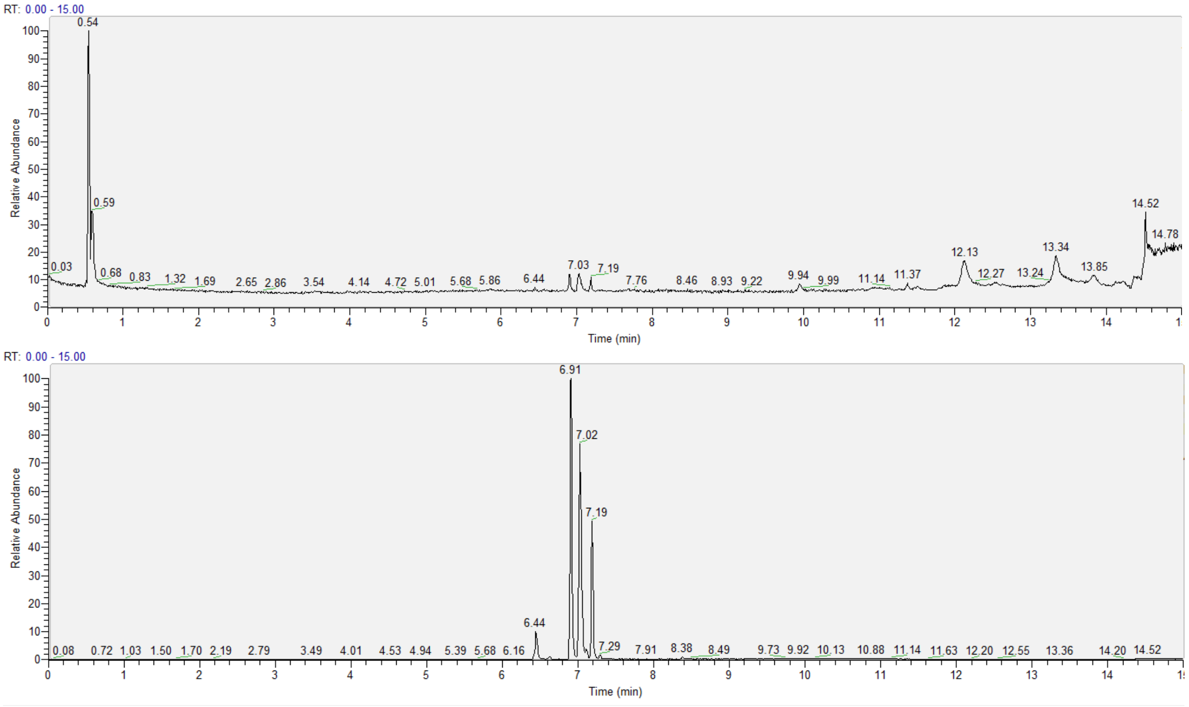

Figure S84: *TIC trace (top) and XIC of diacids **6a** and **6b** (bottom).*

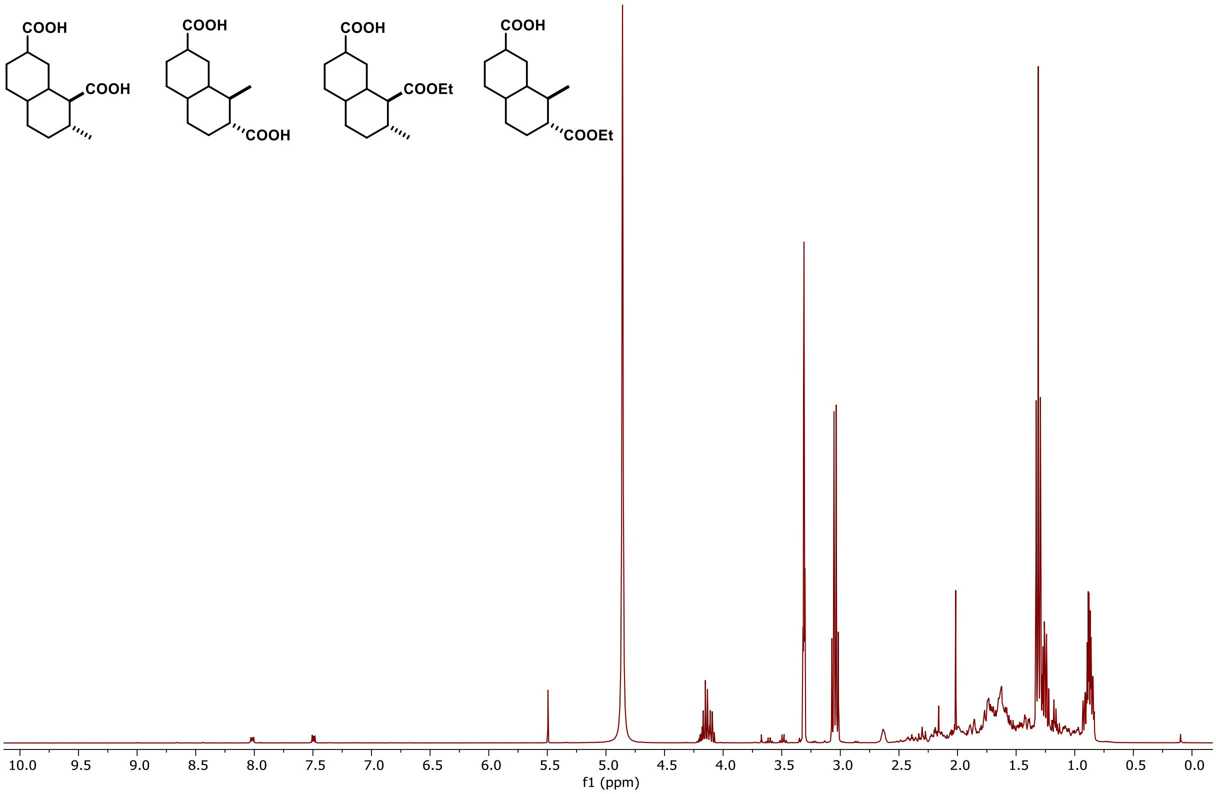

Figure S85: *Crude ^{1}H NMR spectrum of di-acids **6a** and **6b** and mono-esters **47a** and **47b** (400 MHz, $CDCl_3$).*

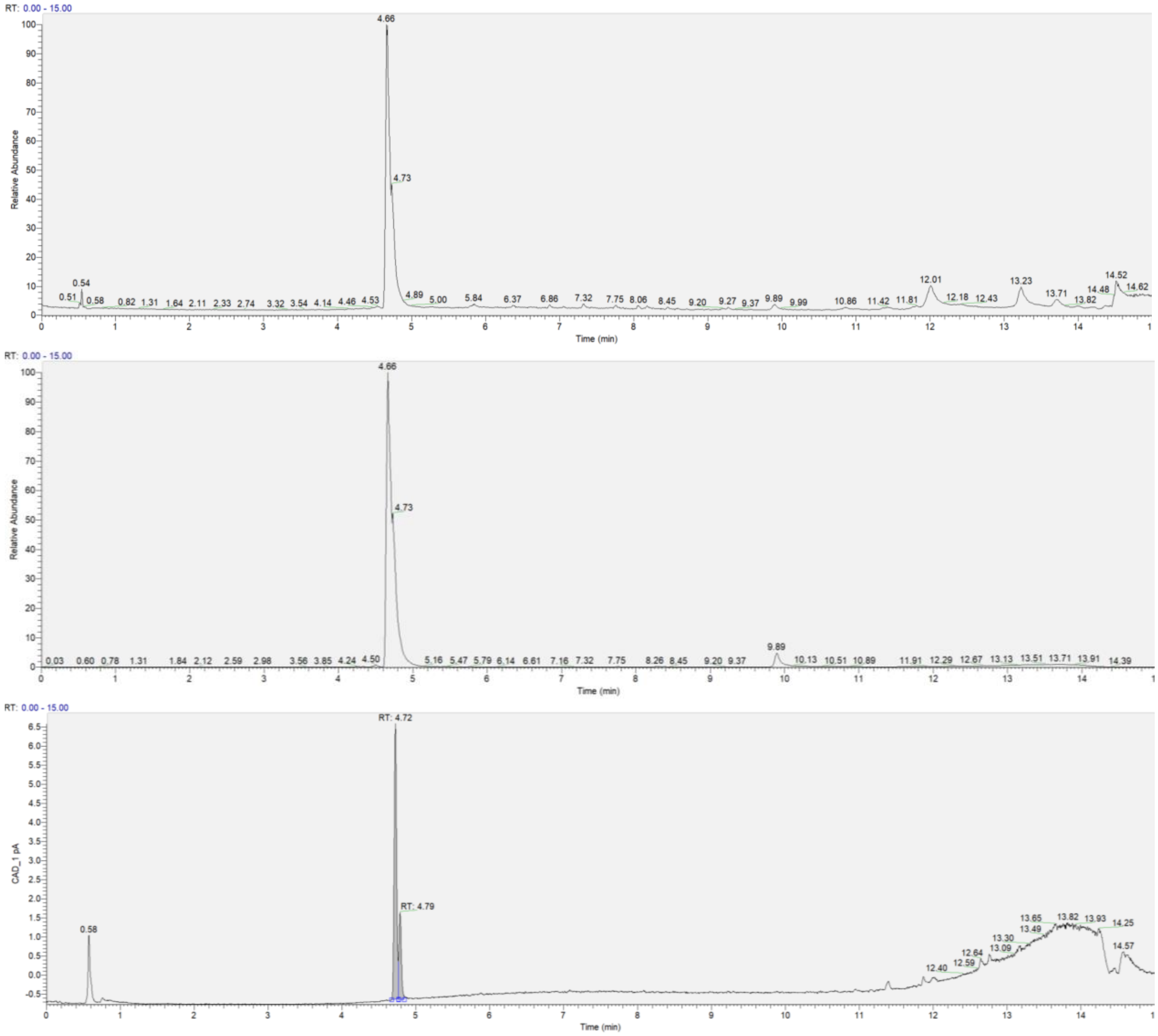

Figure S86: *TIC trace (top), XIC of title compound parent ion (middle), and CAD trace (bottom) of CRAM lactone **7**.*

Table S3: *LC-MS data and peak identities for CRAM lactone **7**.*

Apex RT	Start RT	End RT	Area	%Area	*m/z*	Identity
4.72	4.67	4.76	16.618	76.54	267.0875	Title compound **7** isomer
4.79	4.76	4.84	5.095	23.46	267.0875	Title compound **7** isomer

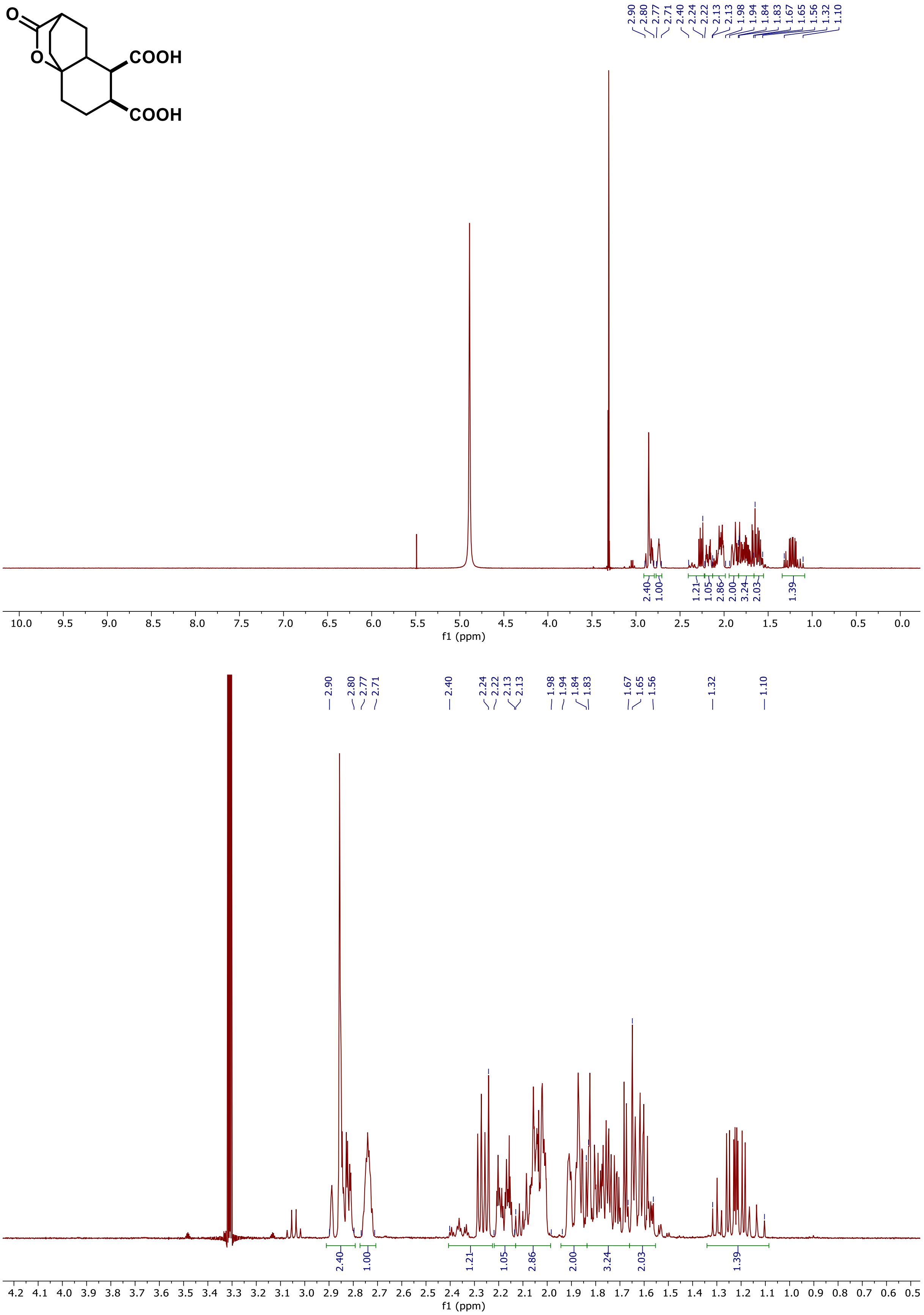

Figure S87: *^{1}H NMR spectra of lactone* ***7*** *(400 MHz, MeOD).*

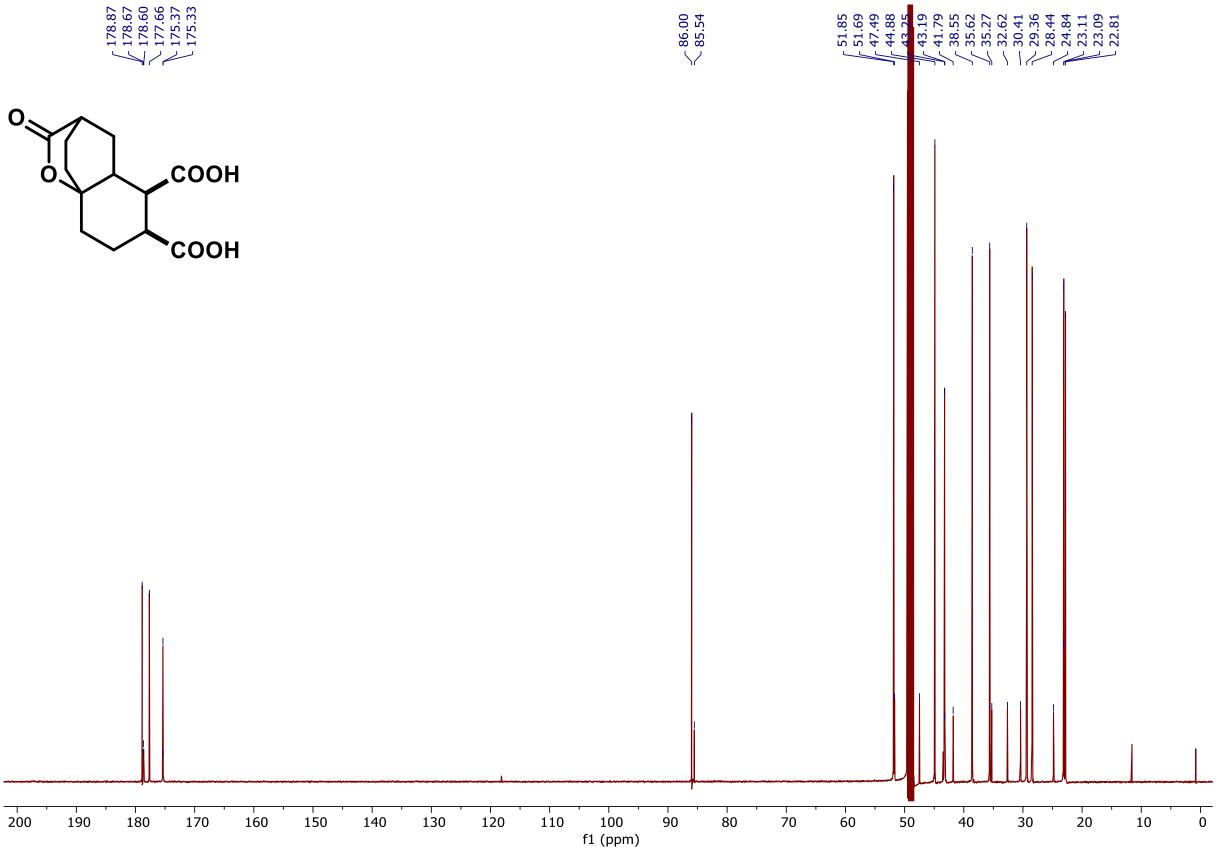
178.87
178.67
178.60
177.66
175.37
175.33
86.00
85.54
51.85
51.69
47.49
44.88
43.25
43.19
41.79
38.55
35.62
35.27
32.62
30.41
29.36
28.44
24.84
23.11
23.09
22.81
O
O
COOH
COOH
200 190 180 170 160 150 140 130 120 110 100 90 80 70 60 50 40 30 20 10 0
f1 (ppm)

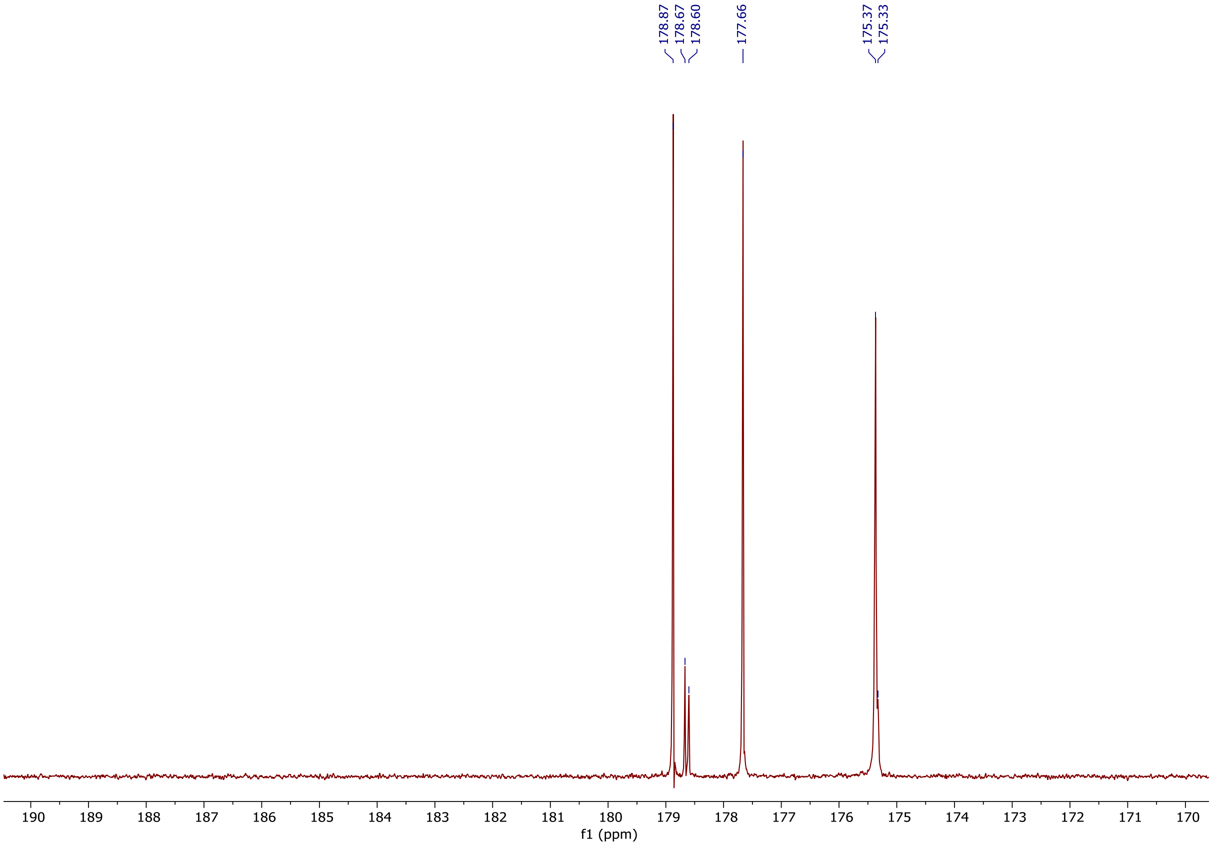
178.87
178.67
178.60
177.66
175.37
175.33
190 189 188 187 186 185 184 183 182 181 180 179 178 177 176 175 174 173 172 171 170
f1 (ppm)

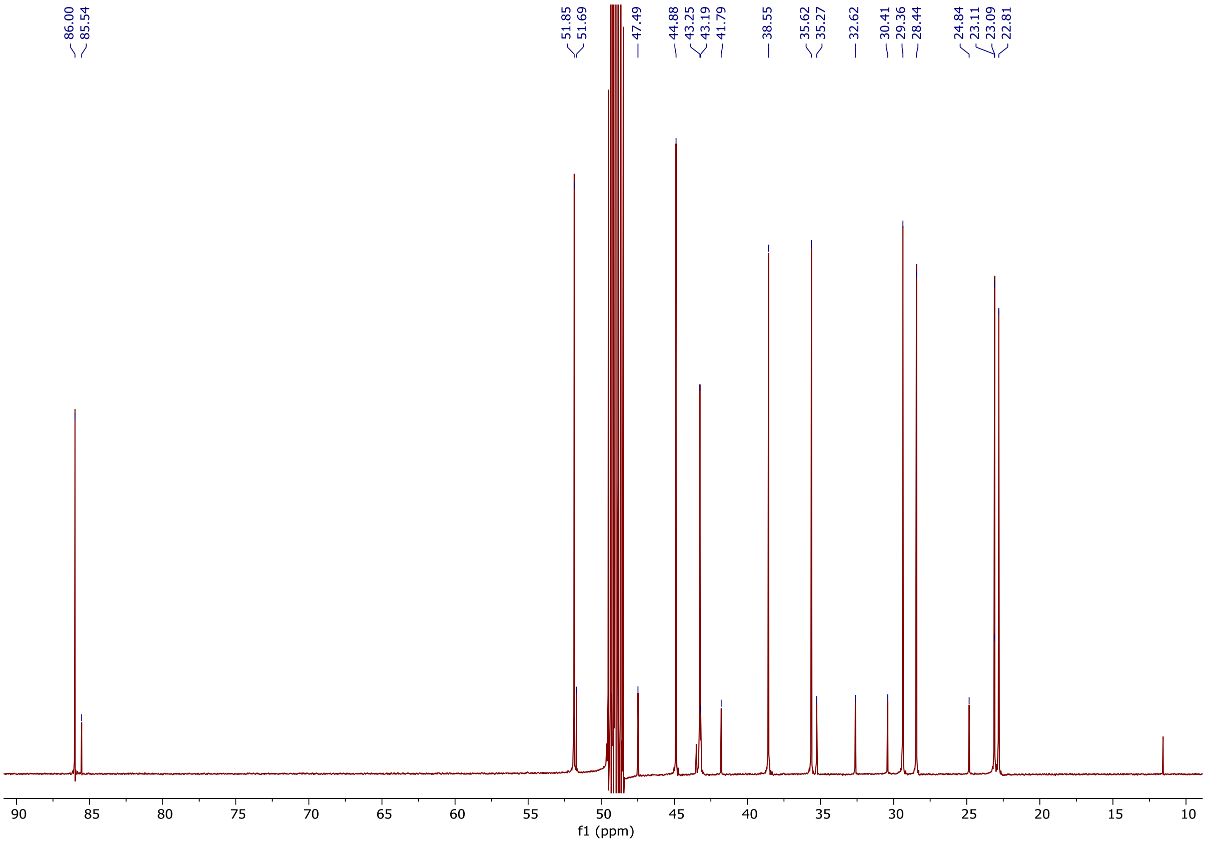

Figure S88: *^{13}C NMR spectra of compound **7** (126 MHz, MeOD).*

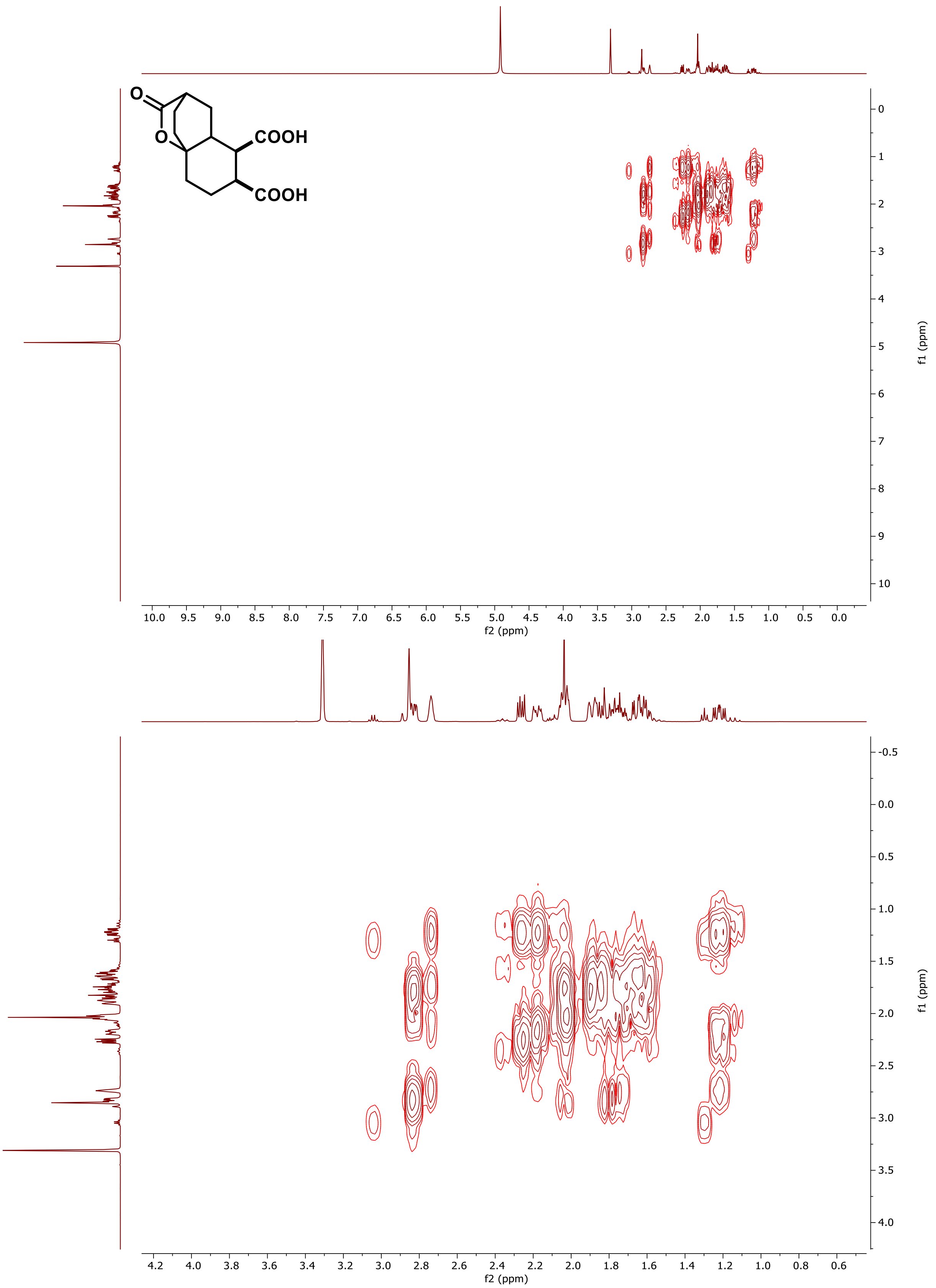

Figure S89: *COSY spectra of CRAM lactone **7**.*

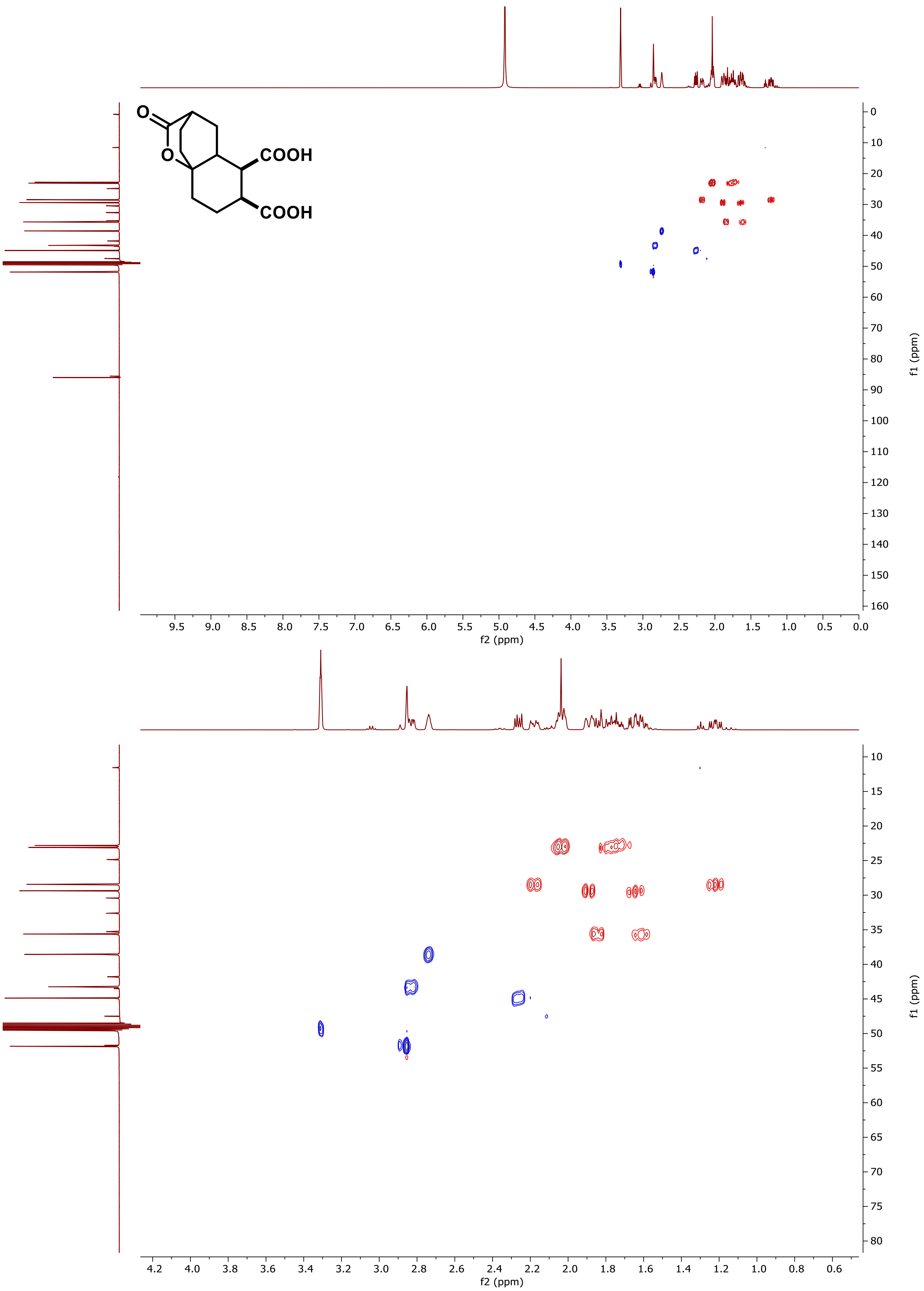

Figure S90: *HSQC spectra of CRAM lactone **7**.*

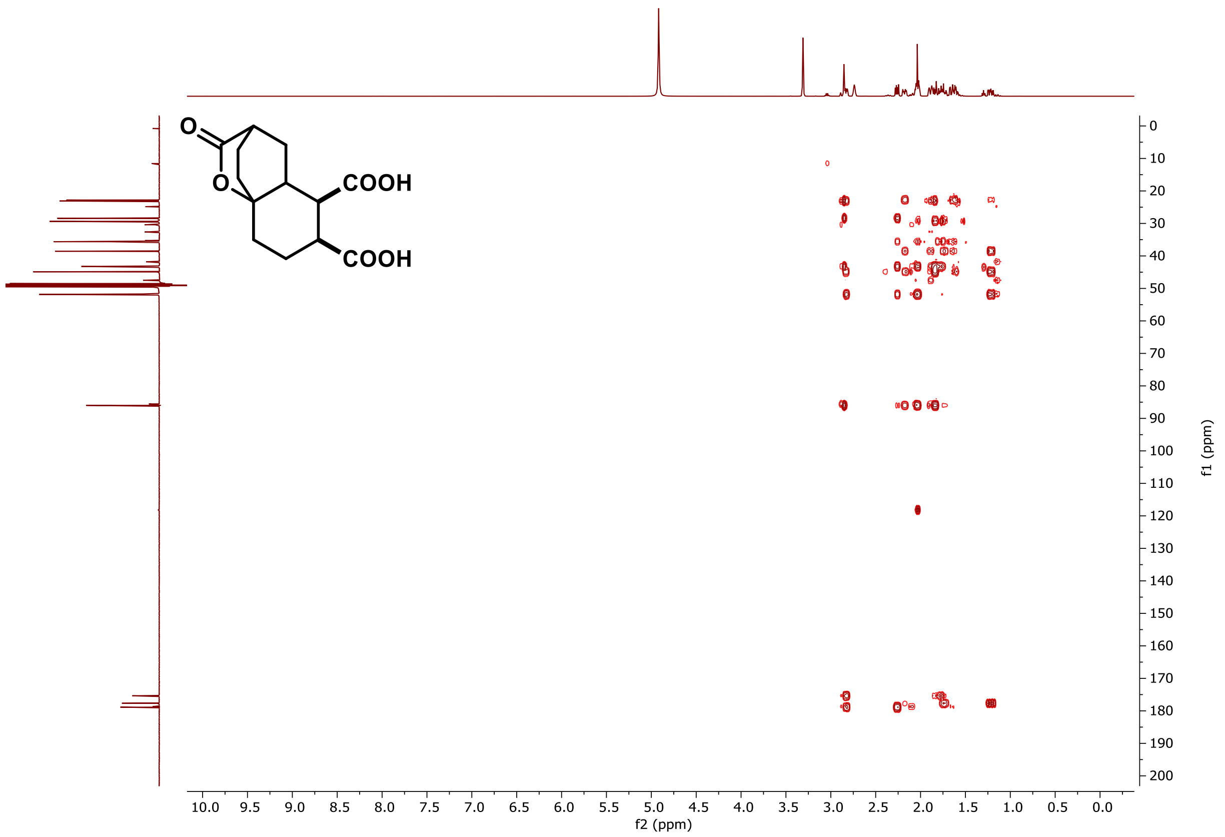
COOH
COOH
f2 (ppm)
f1 (ppm)

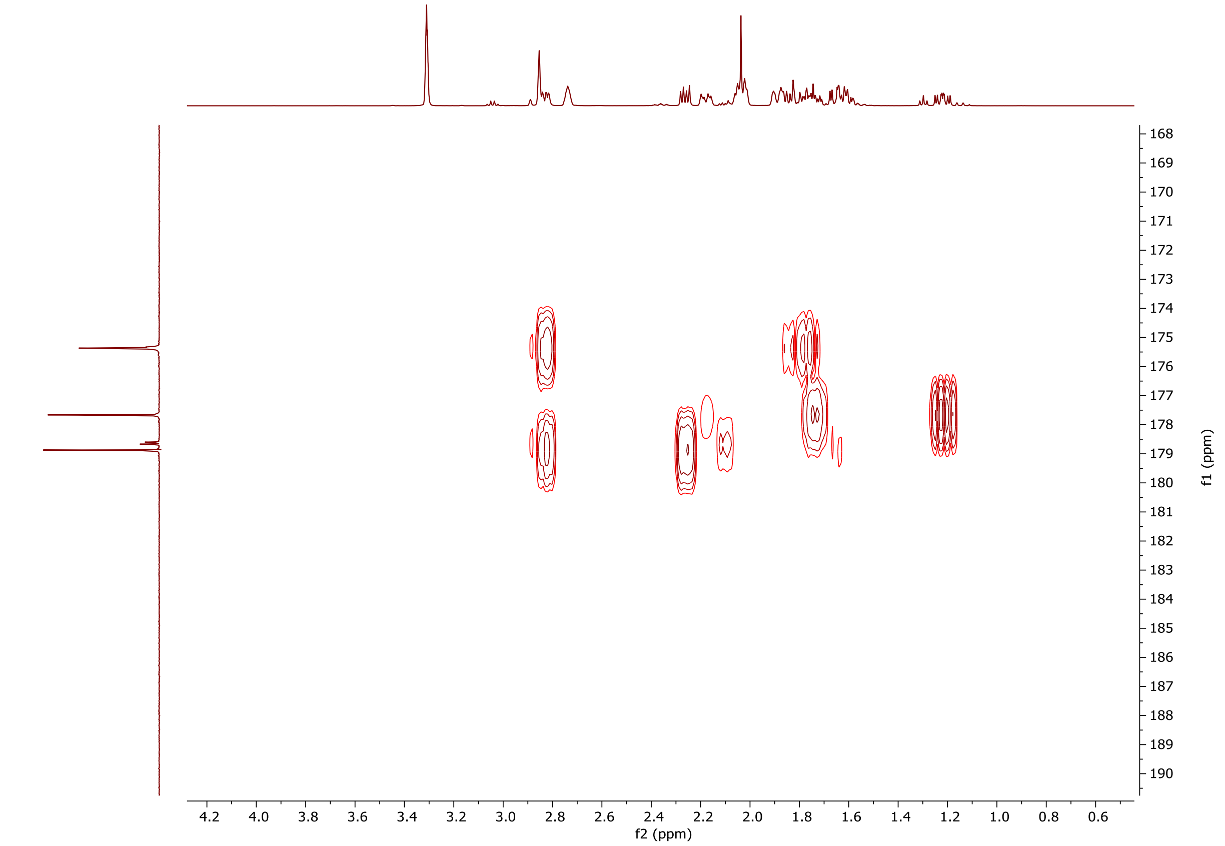
f2 (ppm)
f1 (ppm)

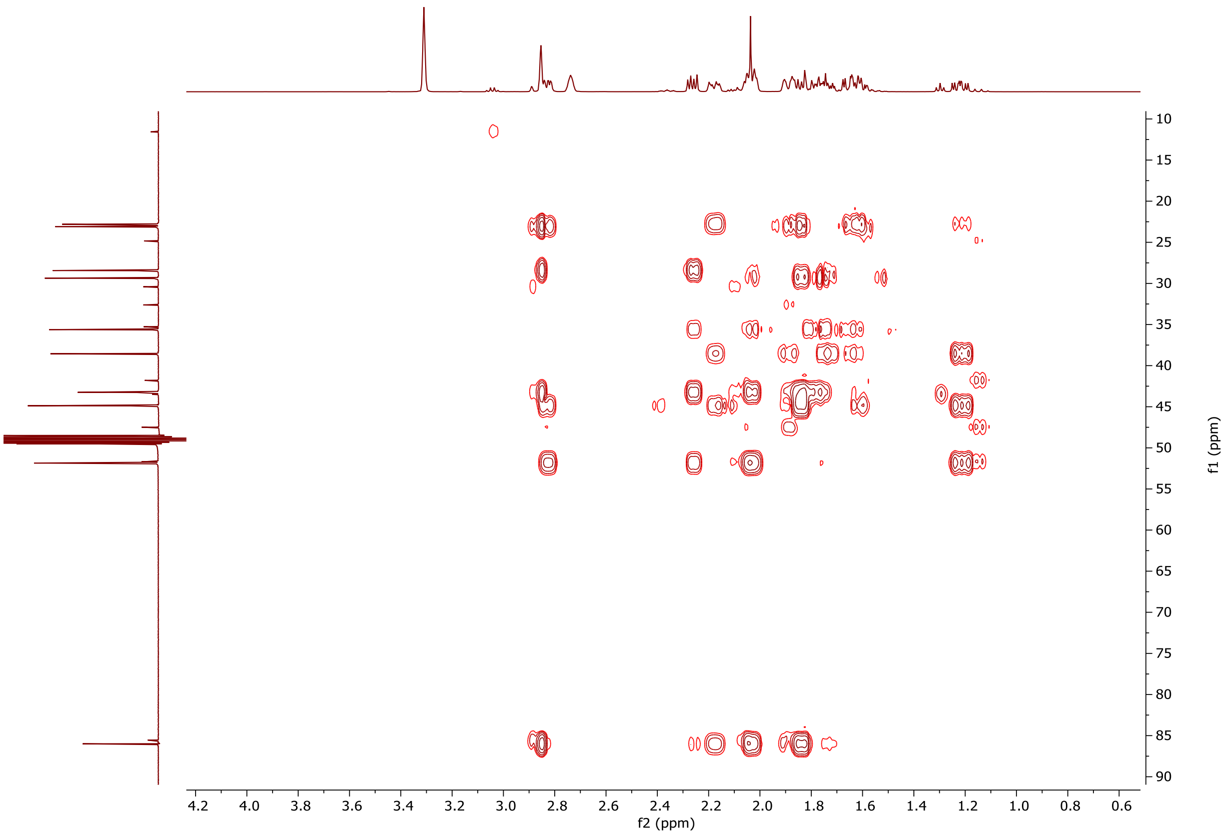

Figure S91: *HMBC spectra of CRAM lactone 7.*

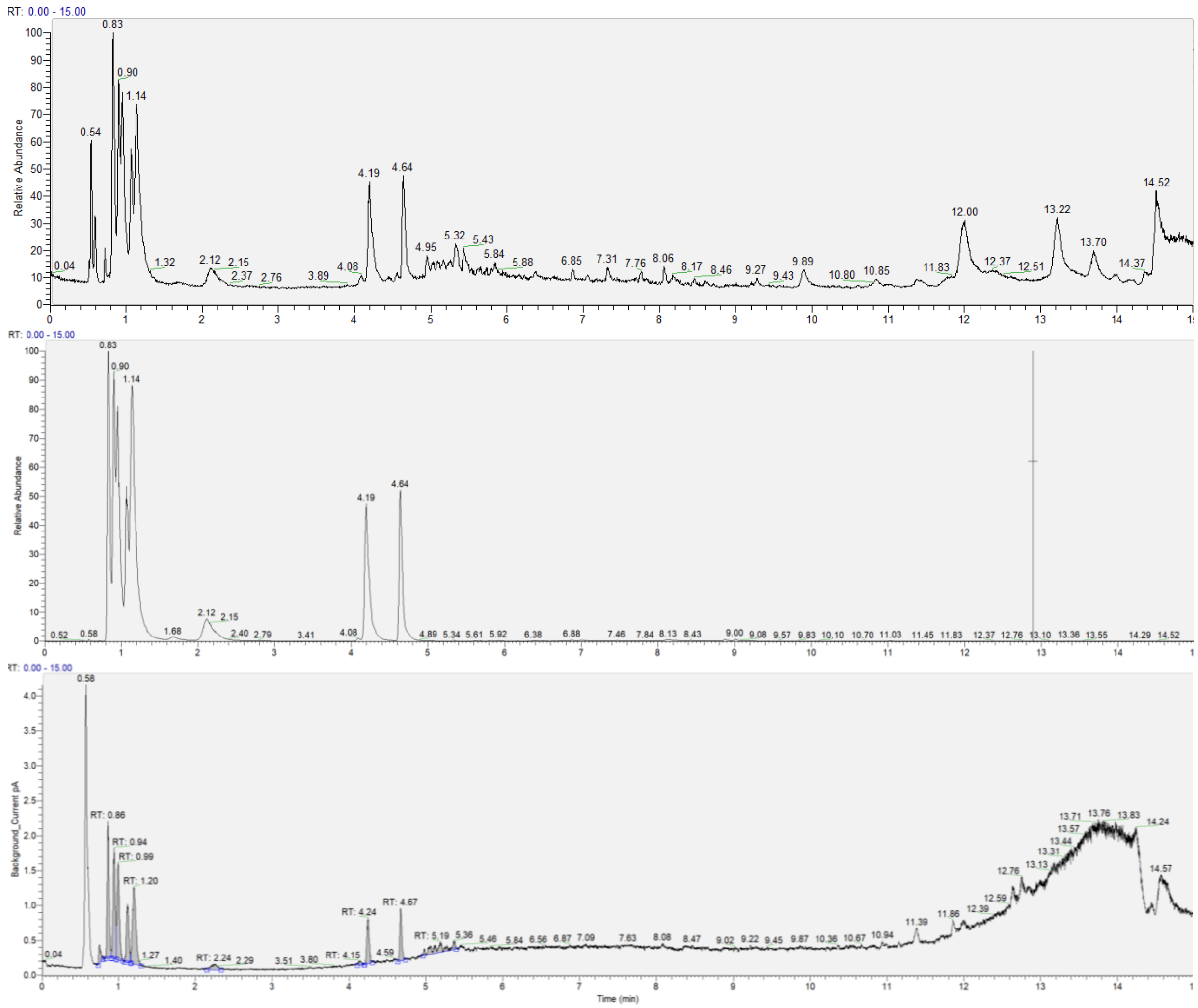

Figure S92: *TIC trace (top), XIC of title compound parent ion (middle), and CAD trace (bottom) of CRAM secondary alcohol* ***8****.*

Table S4: *LC-CAD-MS data and peak identities for CRAM secondary alcohol* ***8****.*

Apex RT	Start RT	End RT	Area	%Area	*m/z*	Identity
0.75	0.73	0.79	0.523	2.48	301.0941	Unknown
0.86	0.83	0.91	3.727	17.67	285.0984	Title compound **8** isomer
0.94	0.91	0.97	3.100	14.70	285.0984	Title compound **8** isomer
0.99	0.97	1.05	2.982	14.13	285.0984	Title compound **8** isomer
1.11	1.08	1.15	1.871	8.87	285.0984	Title compound **8** isomer
1.20	1.15	1.29	3.711	17.59	285.0984	Title compound **8** isomer
2.24	2.15	2.33	0.505	2.40	285.0984	Title compound **8** isomer
4.15	4.11	4.19	0.175	0.83	299.1147	Title compound **8** isomer
4.24	4.20	4.30	1.429	6.78	285.0984	Title compound **8** isomer
4.67	4.63	4.73	1.396	6.62	285.0984	Title compound **8** isomer
5.19	4.96	5.39	1.675	7.94	243.0701<br>267.0879<br>553.1939	Unknown

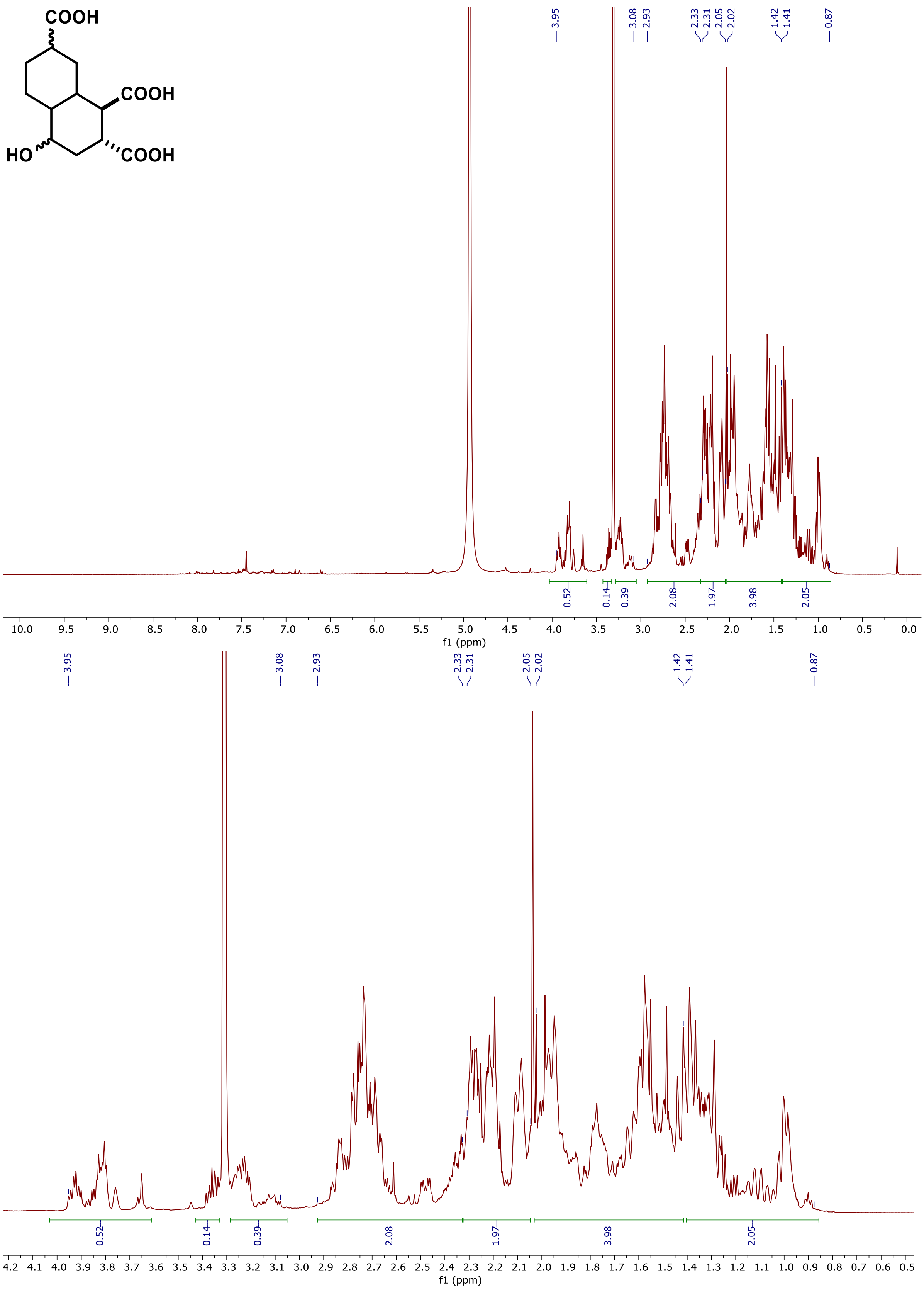

Figure S93: *^{1}H NMR spectra of CRAM secondary alcohol **8** (400 MHz, MeOD).*

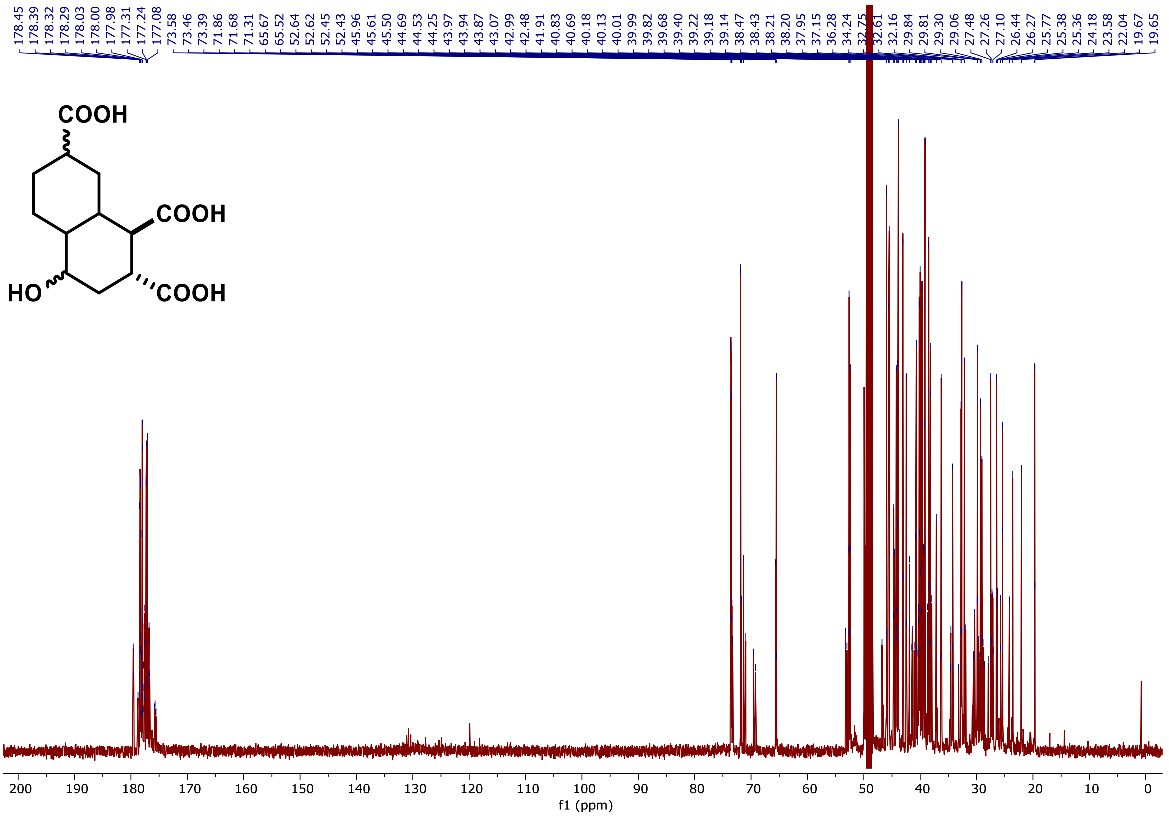
178.45
178.39
178.32
178.29
178.03
178.00
177.98
177.31
177.24
177.08
73.58
73.46
73.39
71.86
71.68
71.31
65.67
65.52
52.64
52.62
52.45
52.43
45.96
45.61
45.50
44.69
44.53
44.25
43.97
43.94
43.87
43.07
42.99
42.48
41.91
40.83
40.69
40.18
40.13
40.01
39.99
39.82
39.68
39.40
39.22
39.18
39.14
38.47
38.43
38.21
38.20
37.95
37.15
36.28
34.24
32.75
32.61
32.16
29.84
29.81
29.30
29.06
27.48
27.26
27.10
26.44
26.27
25.77
25.38
25.36
24.18
23.58
22.04
19.67
19.65
200 190 180 170 160 150 140 130 120 110 100 90 80 70 60 50 40 30 20 10 0
f1 (ppm)
COOH
COOH
HO
COOH

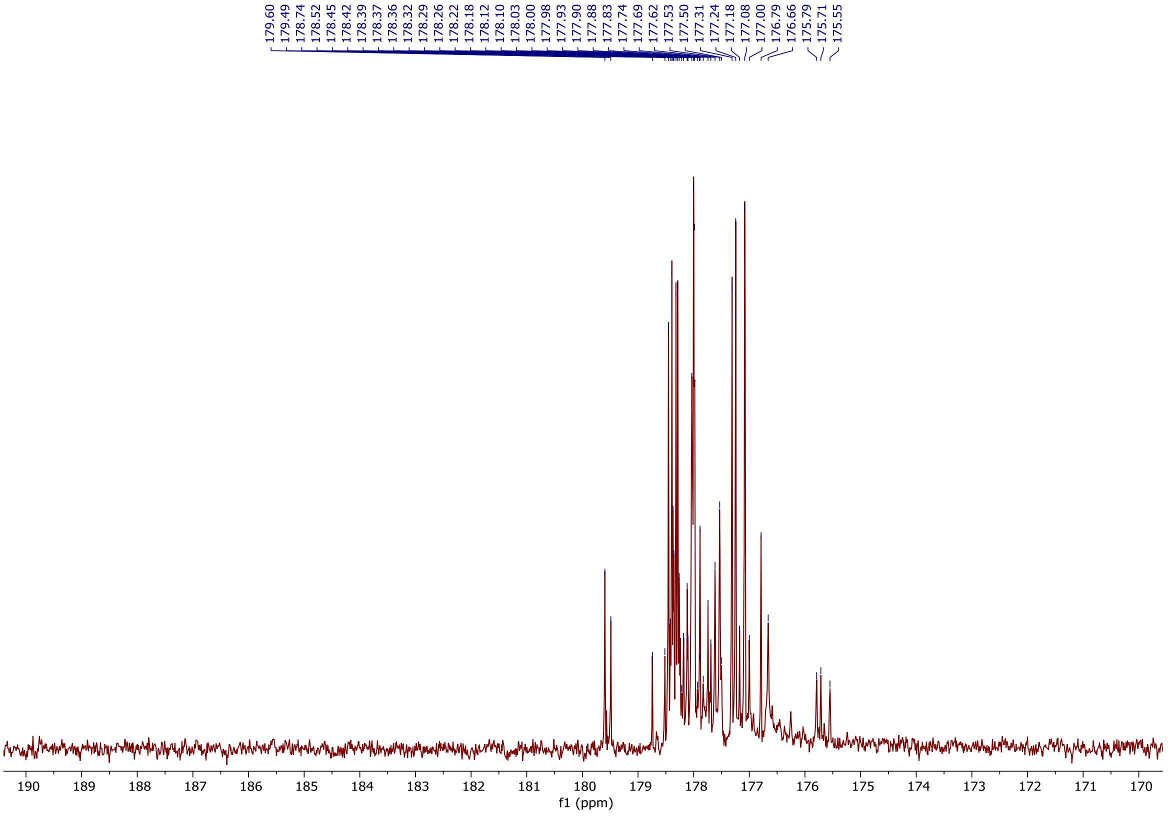
179.60
179.49
178.74
178.52
178.45
178.42
178.39
178.37
178.36
178.32
178.29
178.26
178.22
178.18
178.12
178.10
178.03
178.00
177.98
177.93
177.90
177.88
177.83
177.74
177.69
177.62
177.53
177.50
177.31
177.24
177.18
177.08
177.00
176.79
176.66
175.79
175.71
175.55
190 189 188 187 186 185 184 183 182 181 180 179 178 177 176 175 174 173 172 171 170
f1 (ppm)

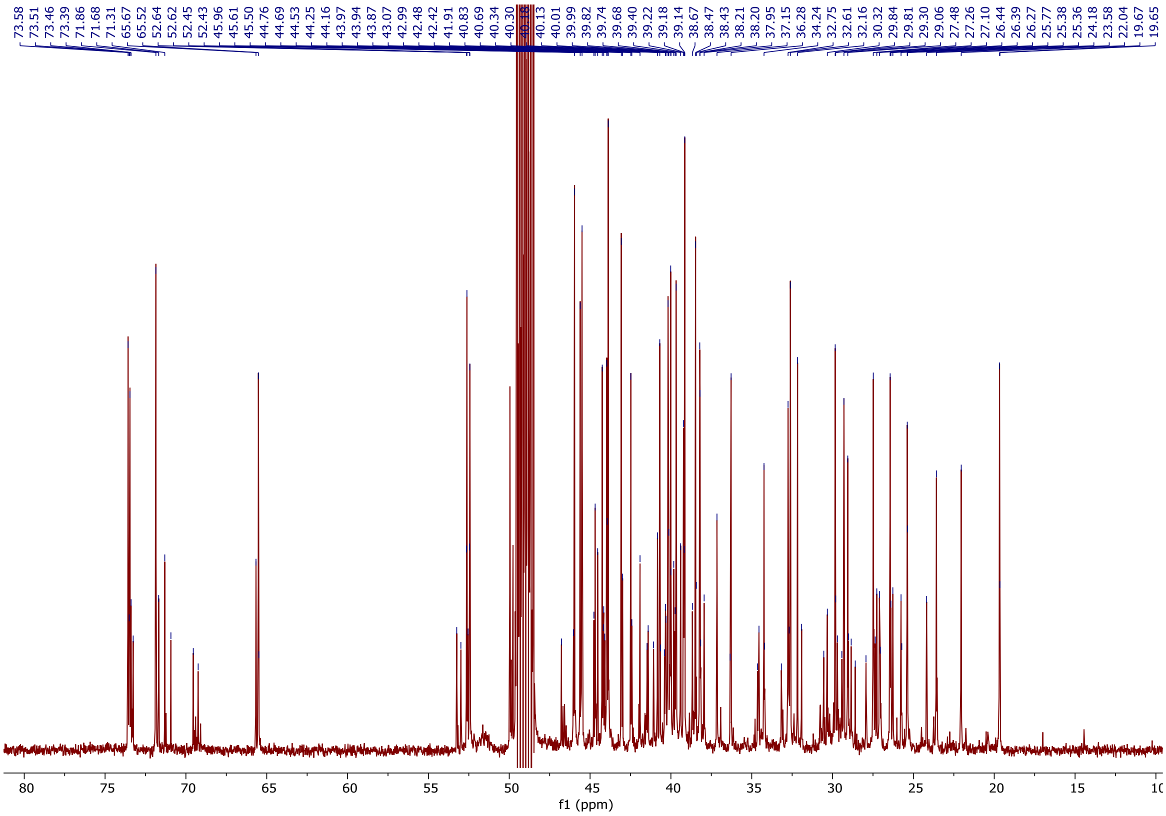

Figure S94: *^{13}C NMR spectra of CRAM secondary alcohol **8** (101 MHz, MeOD).*

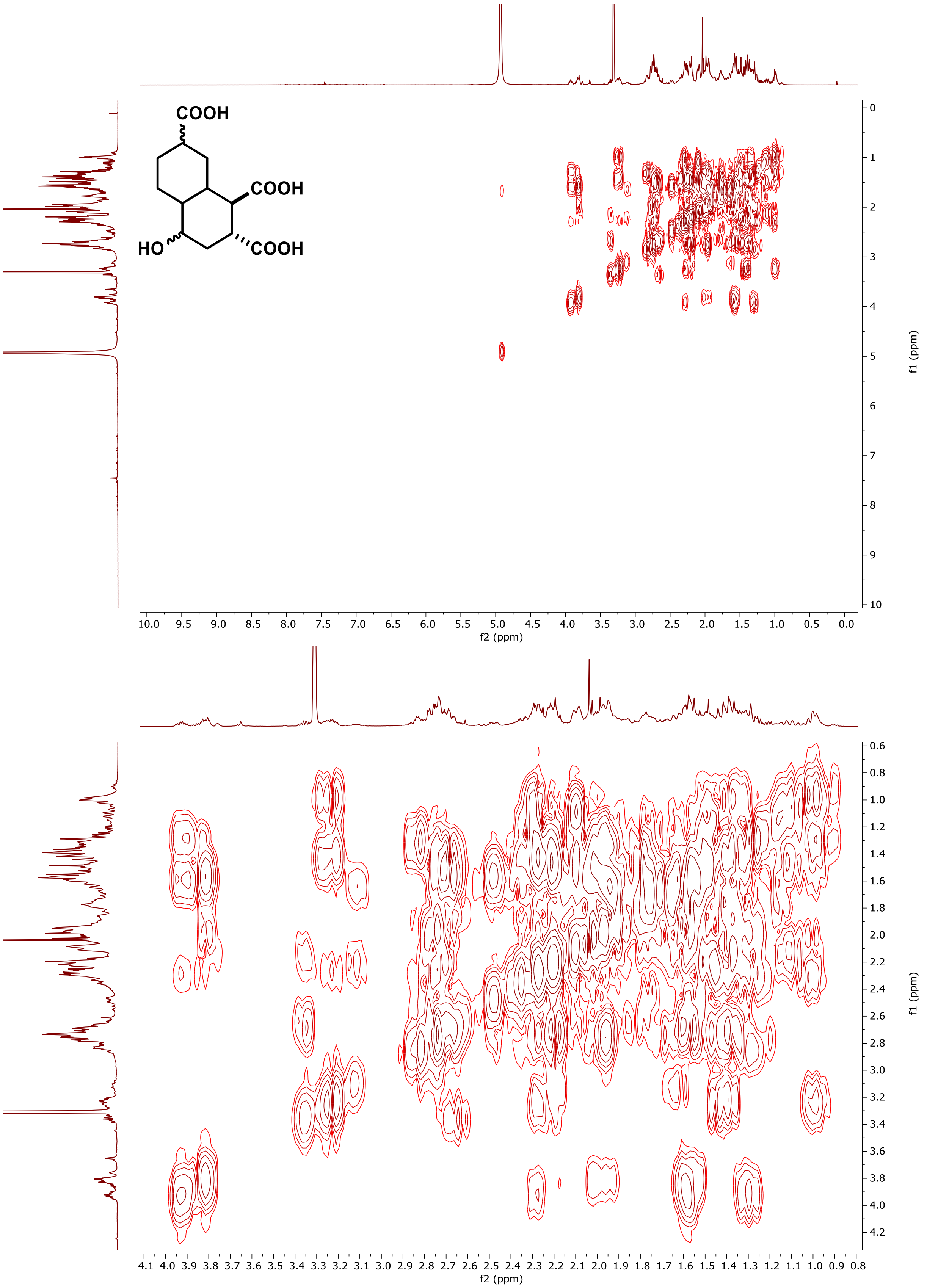

Figure S95: *COSY spectra of CRAM secondary alcohol* ***8****.*

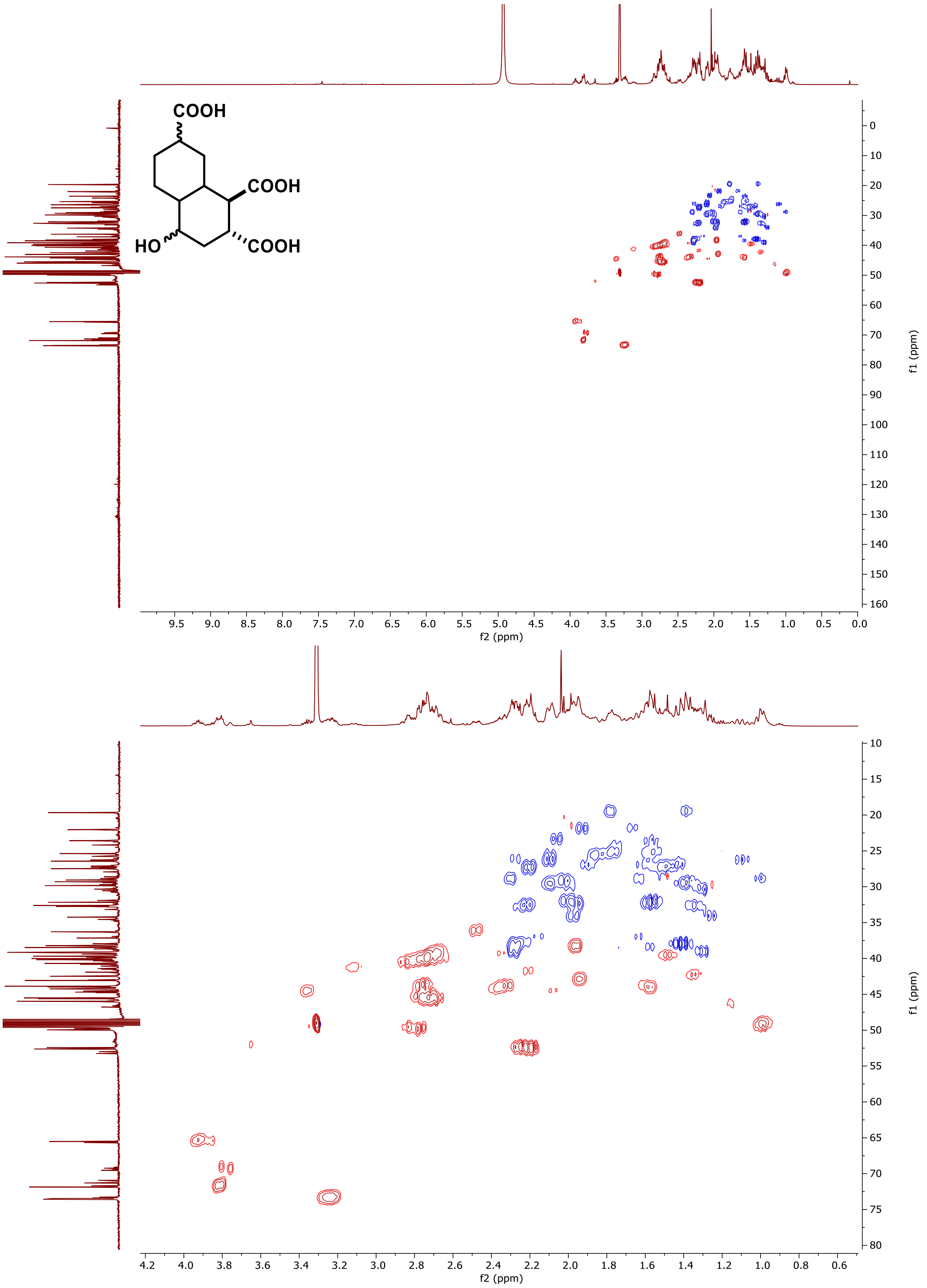

Figure S96: *HSQC spectra of CRAM secondary alcohol **8**.*

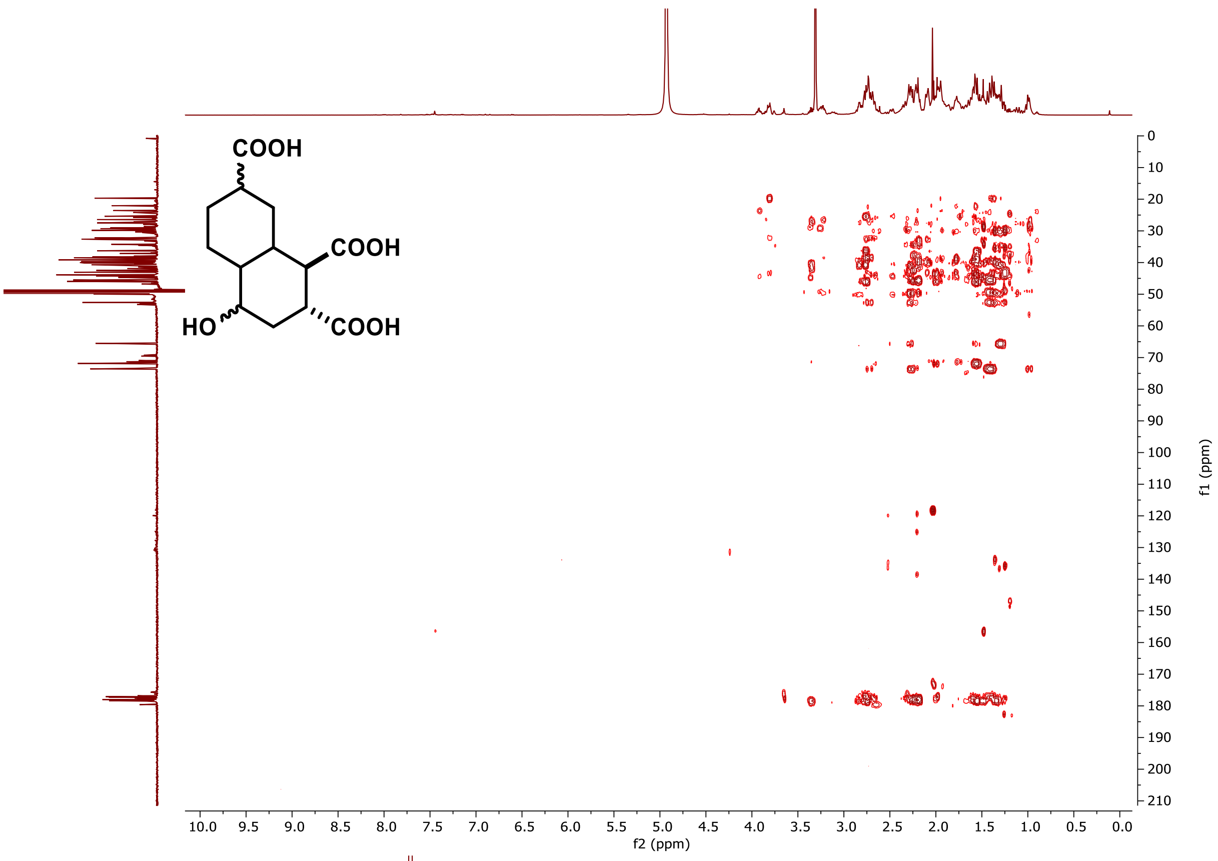
COOH
COOH
HO
COOH
f2 (ppm)
f1 (ppm)

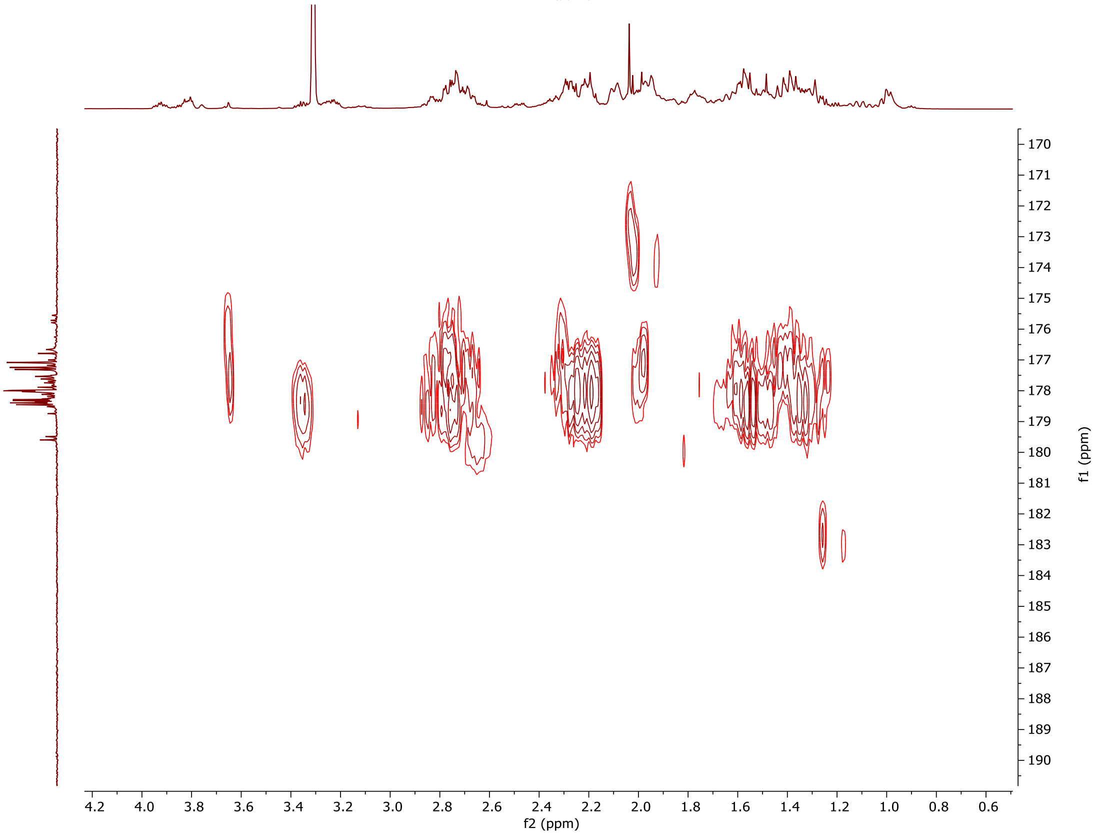
f2 (ppm)
f1 (ppm)

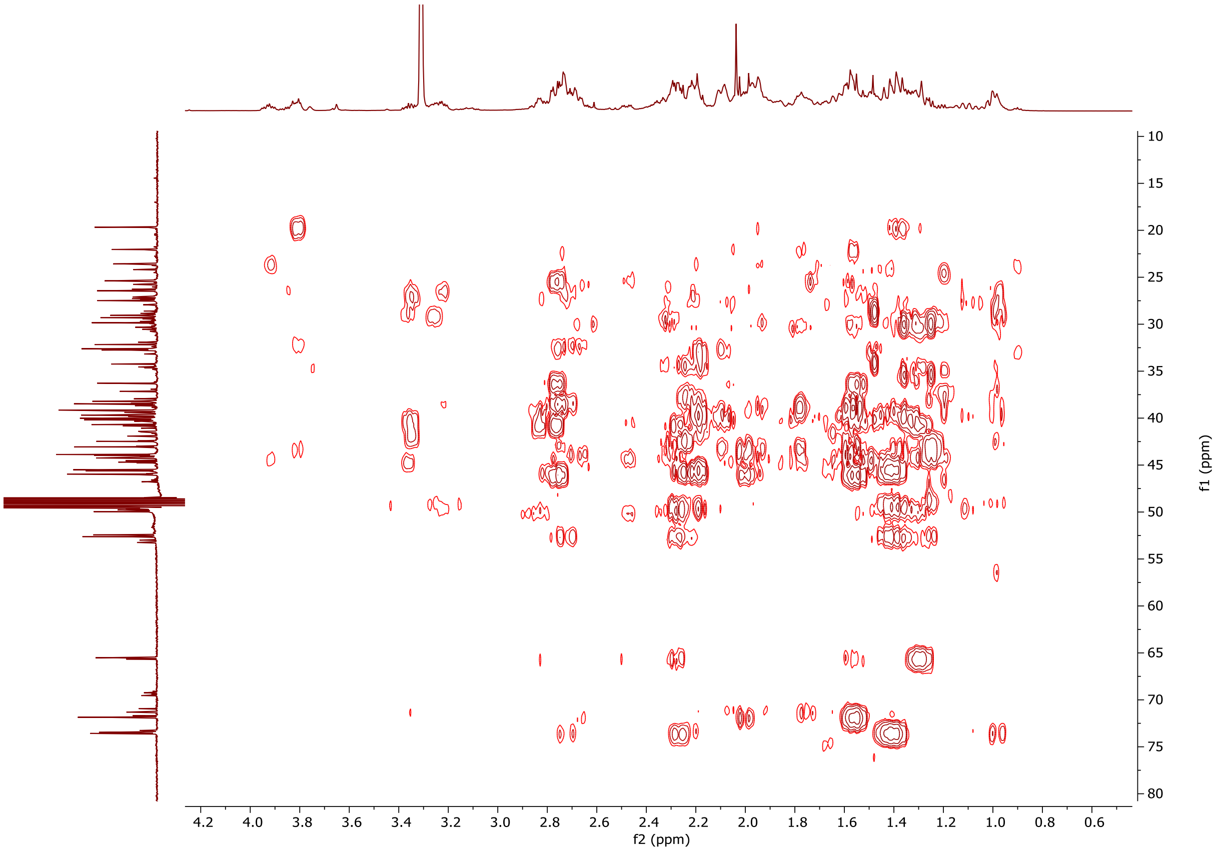

Figure S97: *HMBC spectra of CRAM secondary alcohol* ***8****.*

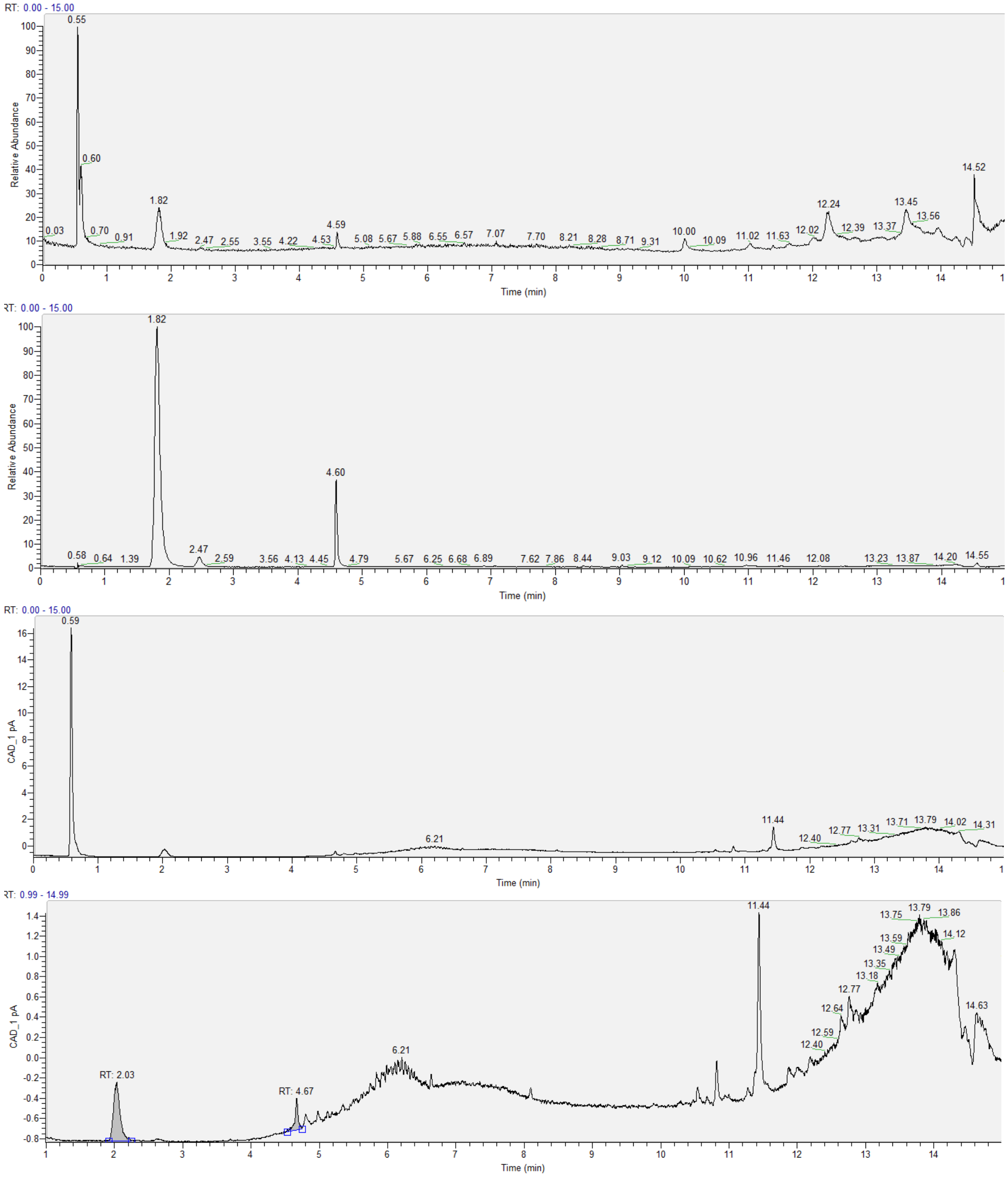

Figure S98: *TIC trace (top), XIC of title compound parent ion (middle-top), CAD trace (middle-bottom), and CAD trace from 1-15 minutes (bottom) of CRAM tertiary alcohol **9**.*

Table S5: *LC-MS data and peak identities for CRAM tertiary alcohol **9**.*

Apex RT	Start RT	End RT	Area	%Area	*m/z*	Identity
2.03	1.91	2.25	3.851	78.85	285.0983	Title compound **9** isomer
4.67	4.53	4.75	1.033	21.15	285.0984	Title compound **9** isomer

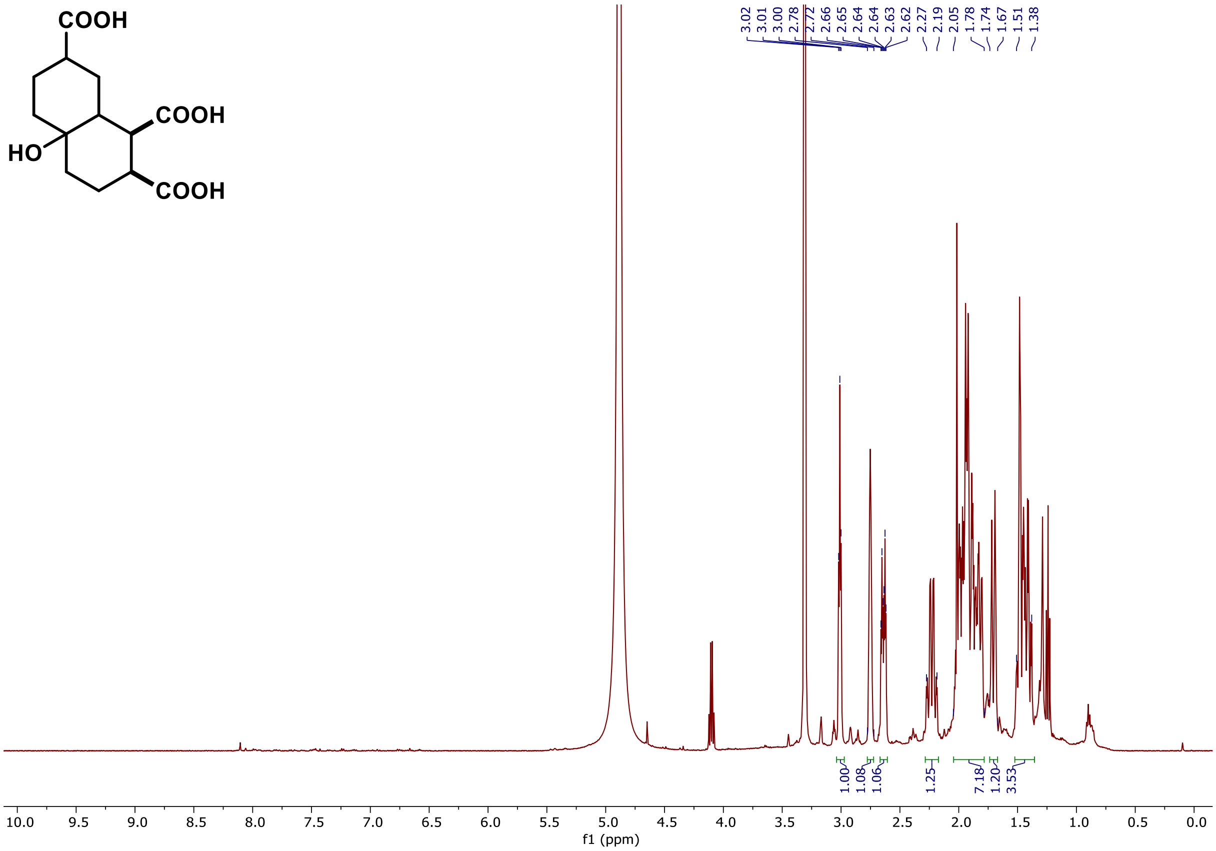

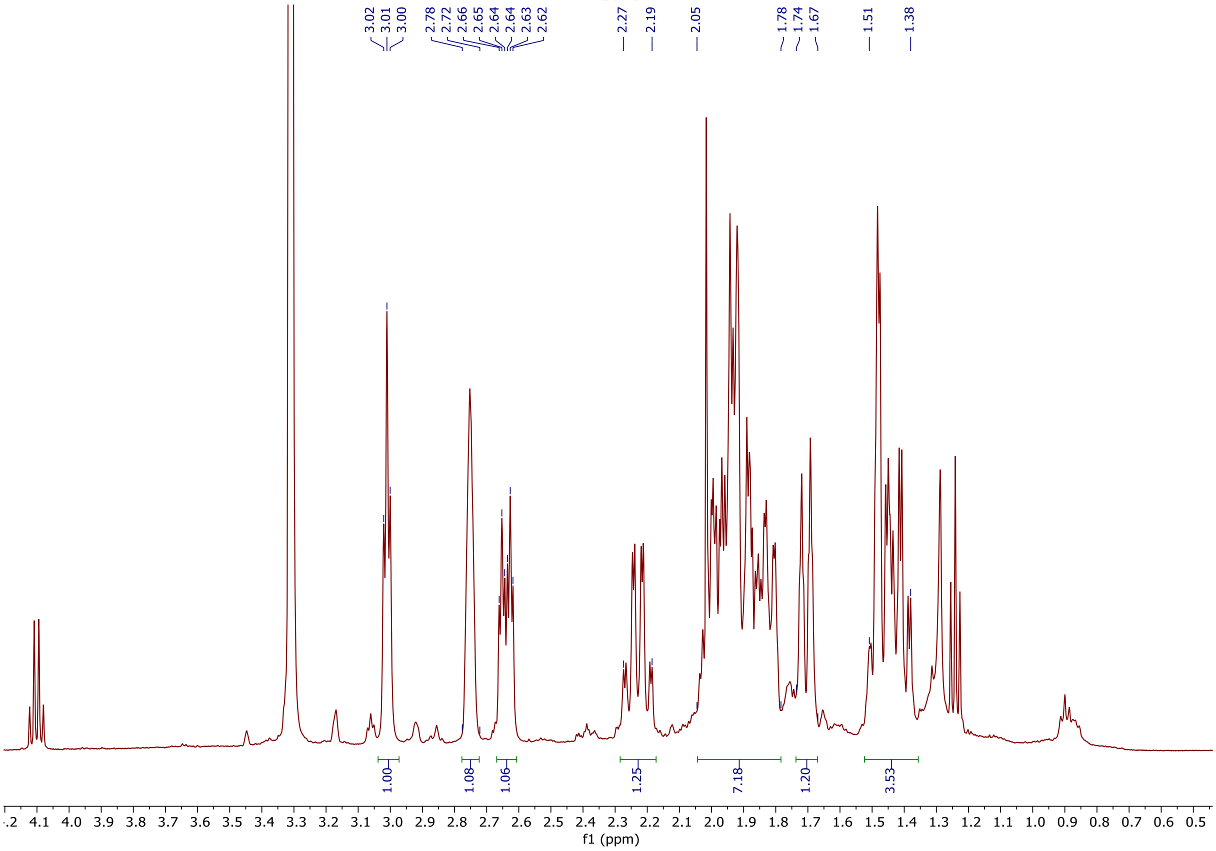

Figure S99: [1]*H NMR spectra of CRAM tertiary alcohol* ***9*** *(500 MHz, MeOD).*

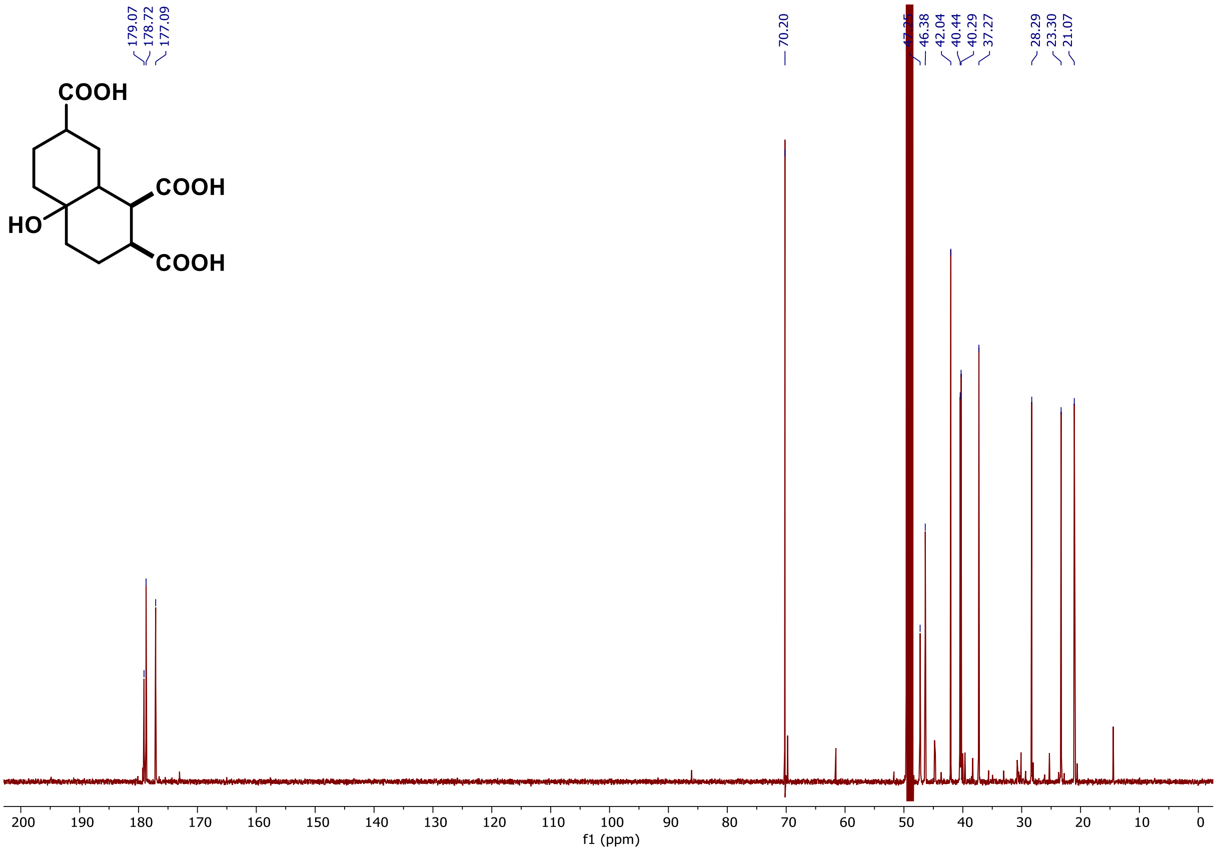
COOH
COOH
COOH
HO
179.07
178.72
177.09
70.20
46.38
42.04
40.44
40.29
37.27
28.29
23.30
21.07
f1 (ppm)

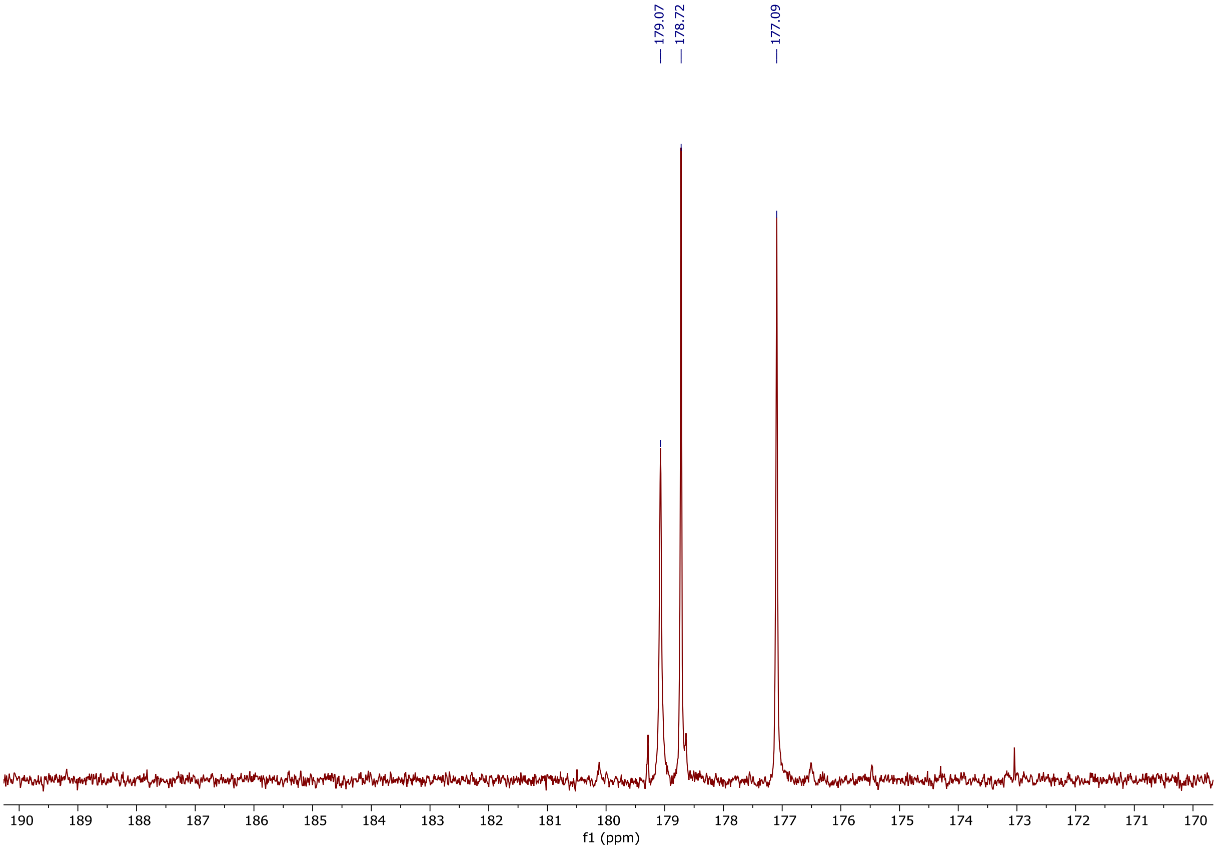
179.07
178.72
177.09
f1 (ppm)

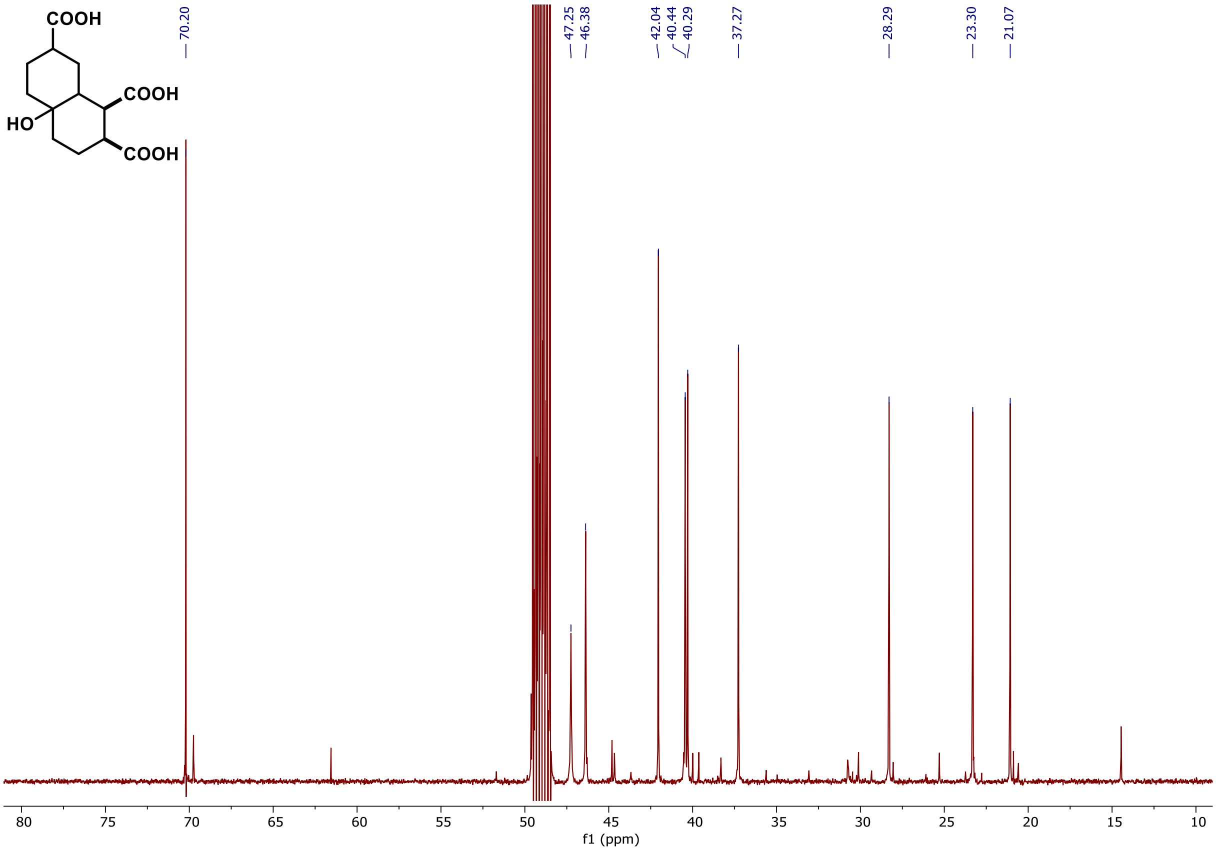

Figure S100: *^{13}C NMR spectra of CRAM tertiary alcohol* ***9*** *(126 MHz, MeOD).*

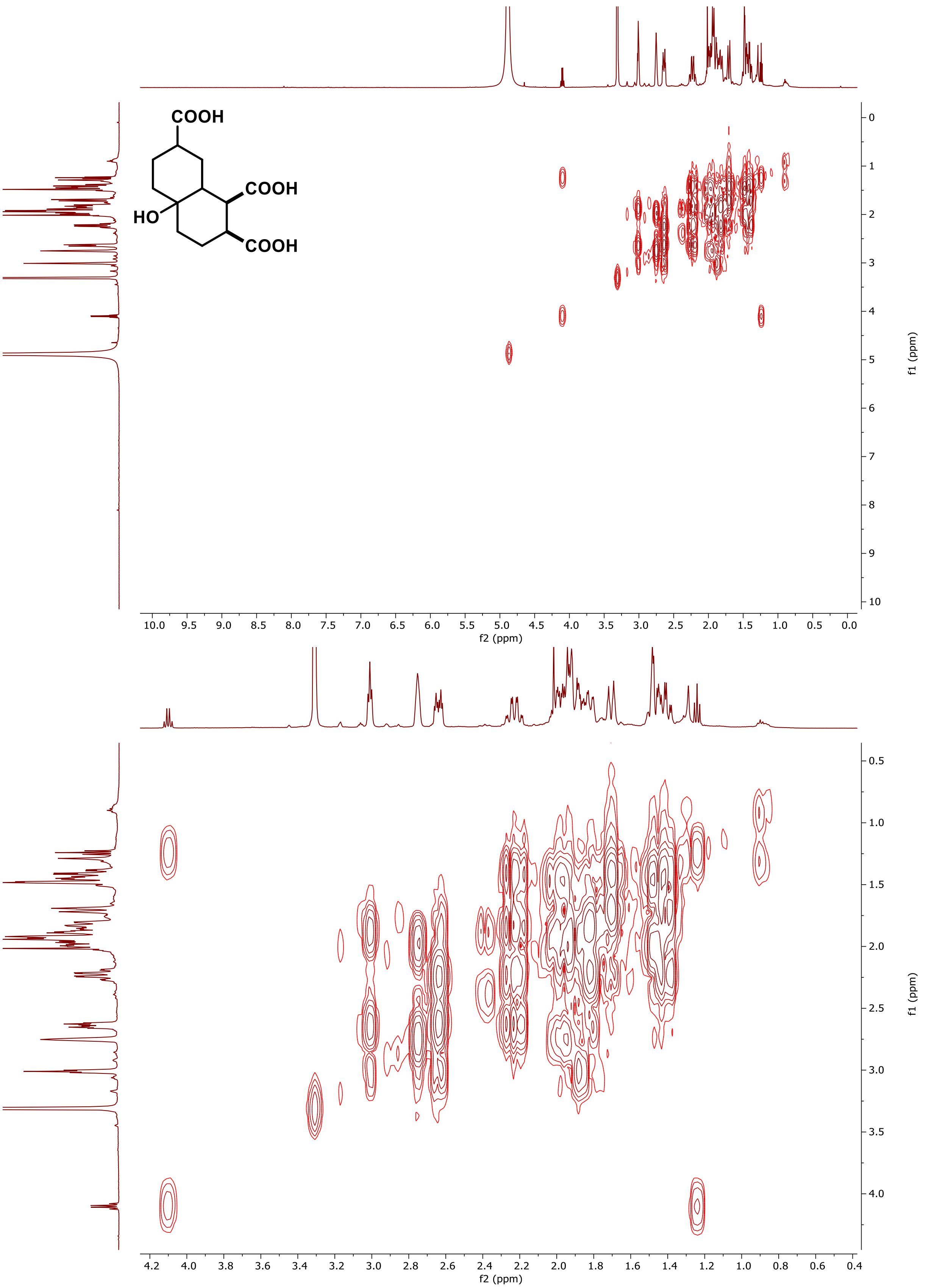

Figure S101: *COSY spectra of CRAM tertiary alcohol **9**.*

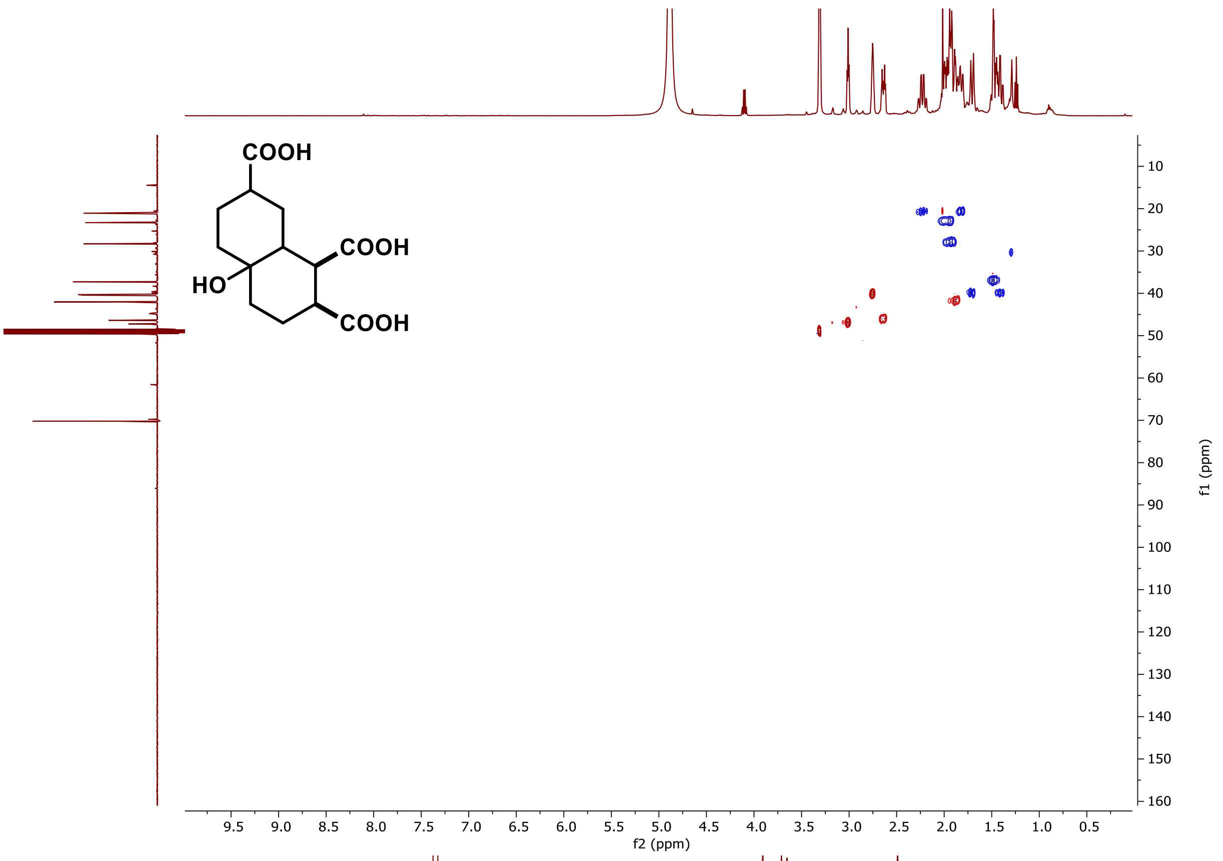

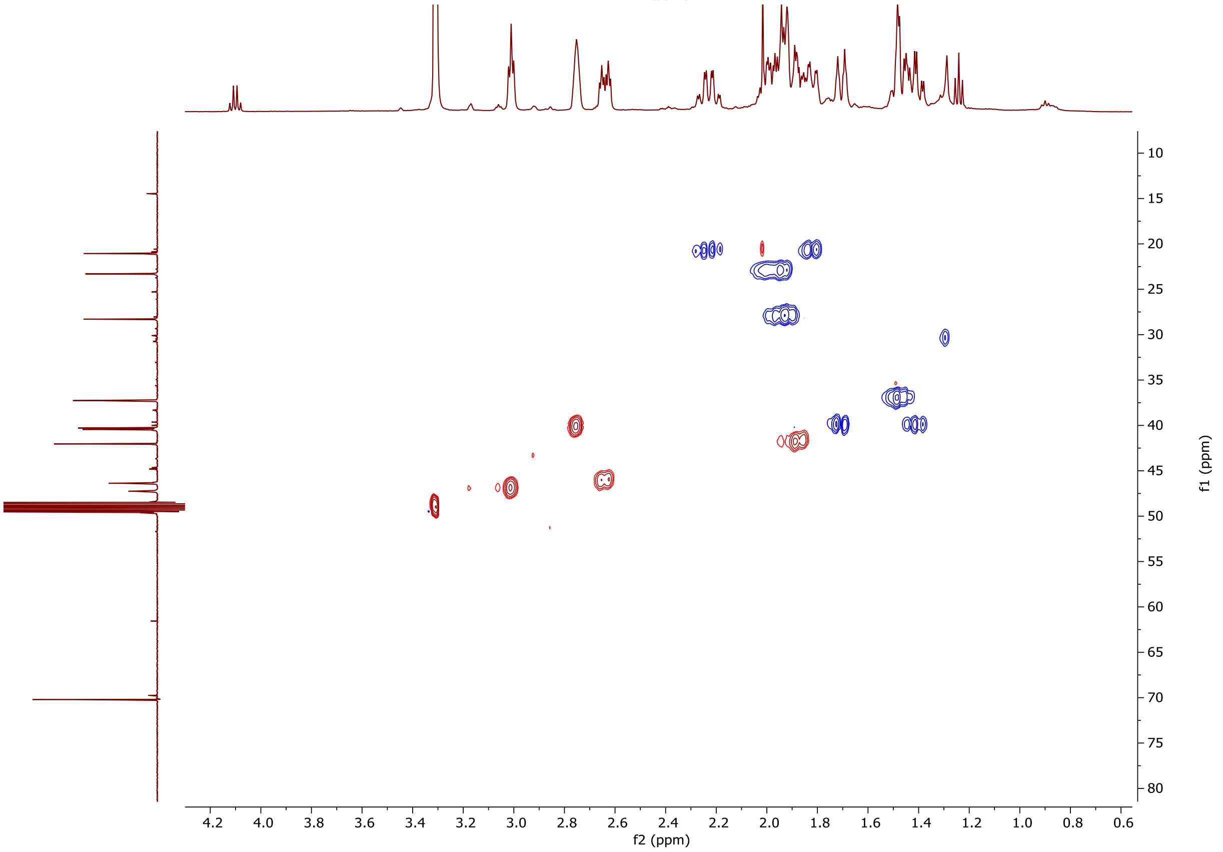

Figure S102: *HSQC spectra of CRAM tertiary alcohol **9**.*

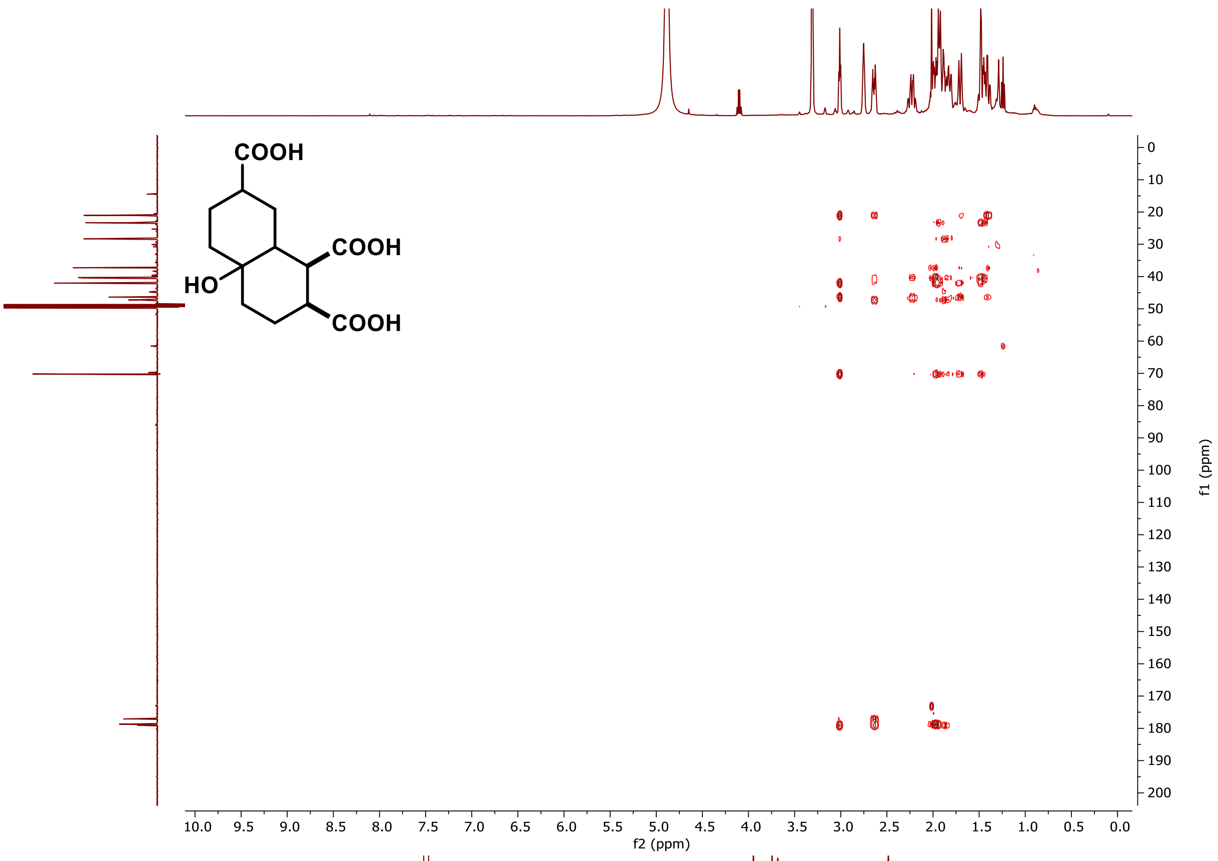
COOH
COOH
HO
COOH
f2 (ppm)
f1 (ppm)

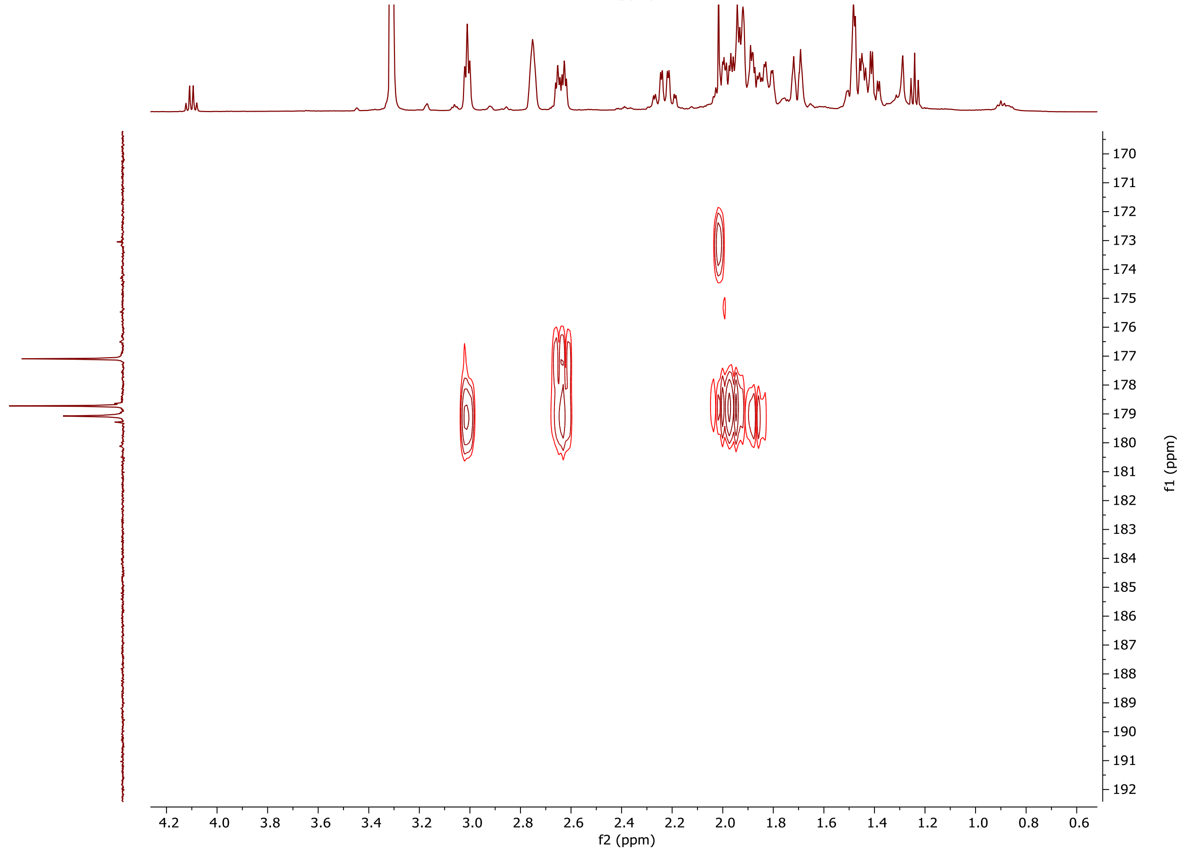
f2 (ppm)
f1 (ppm)

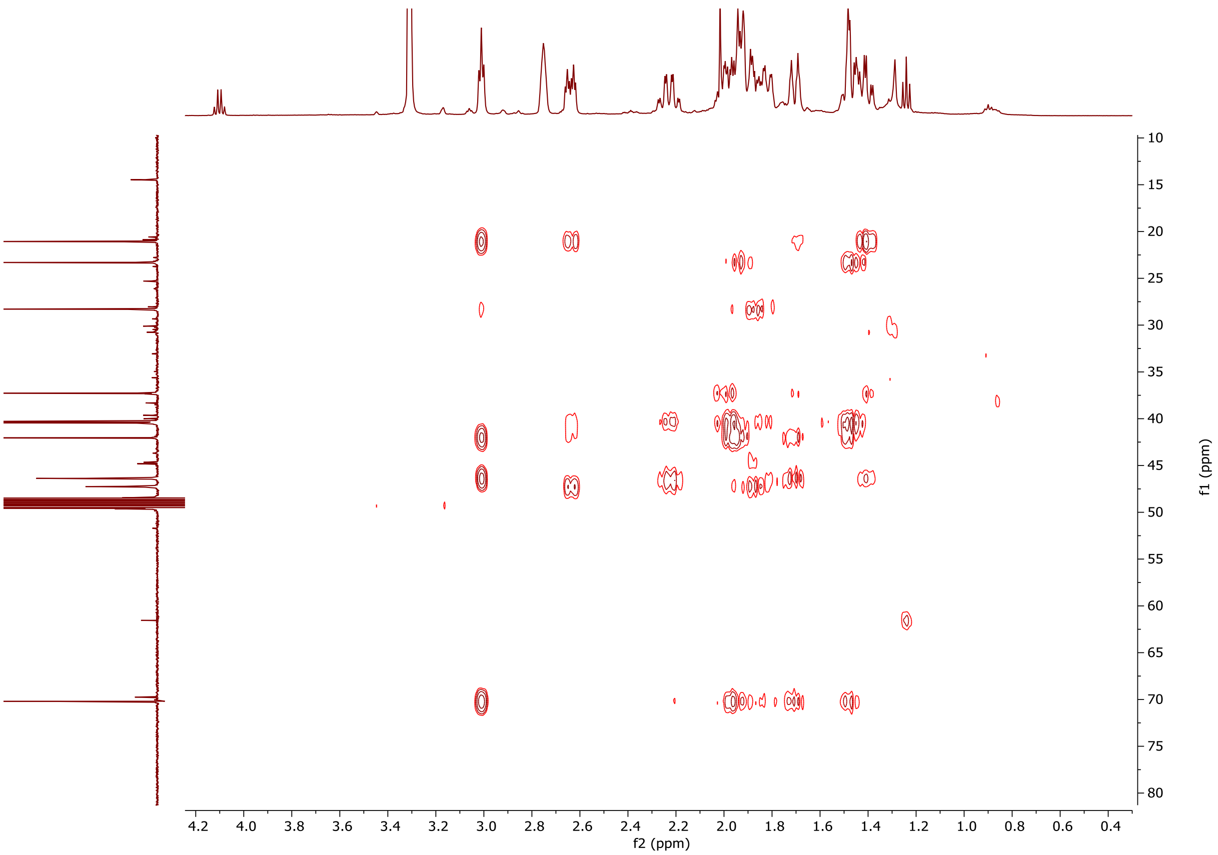

Figure S103: *HMBC spectra of CRAM tertiary alcohol* ***9****.*

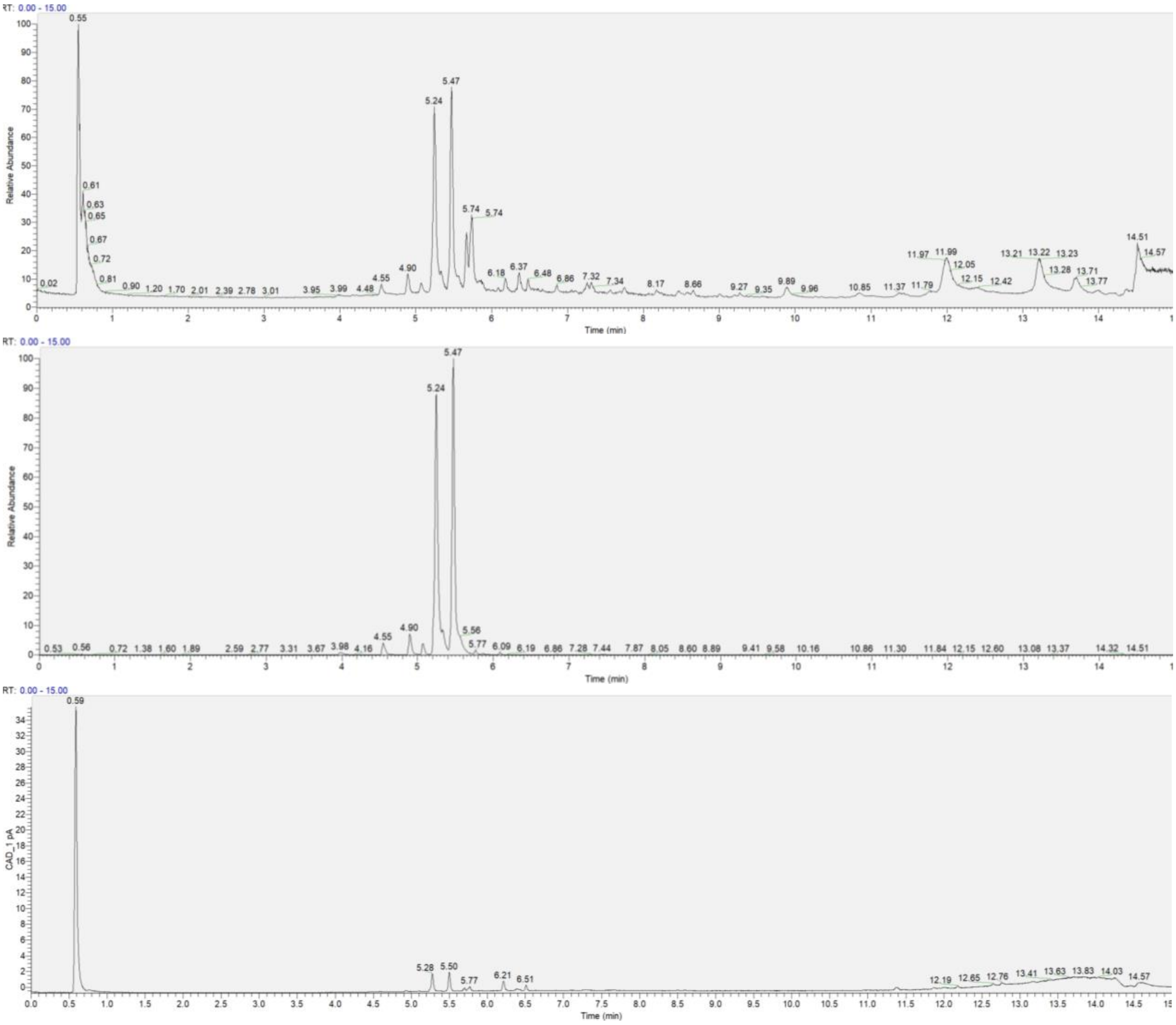

Figure S104: *TIC trace (top), XIC of title compound parent ion (middle-top), CAD trace (middle-bottom), and CAD trace (bottom) of CRAM ether* ***10****.*

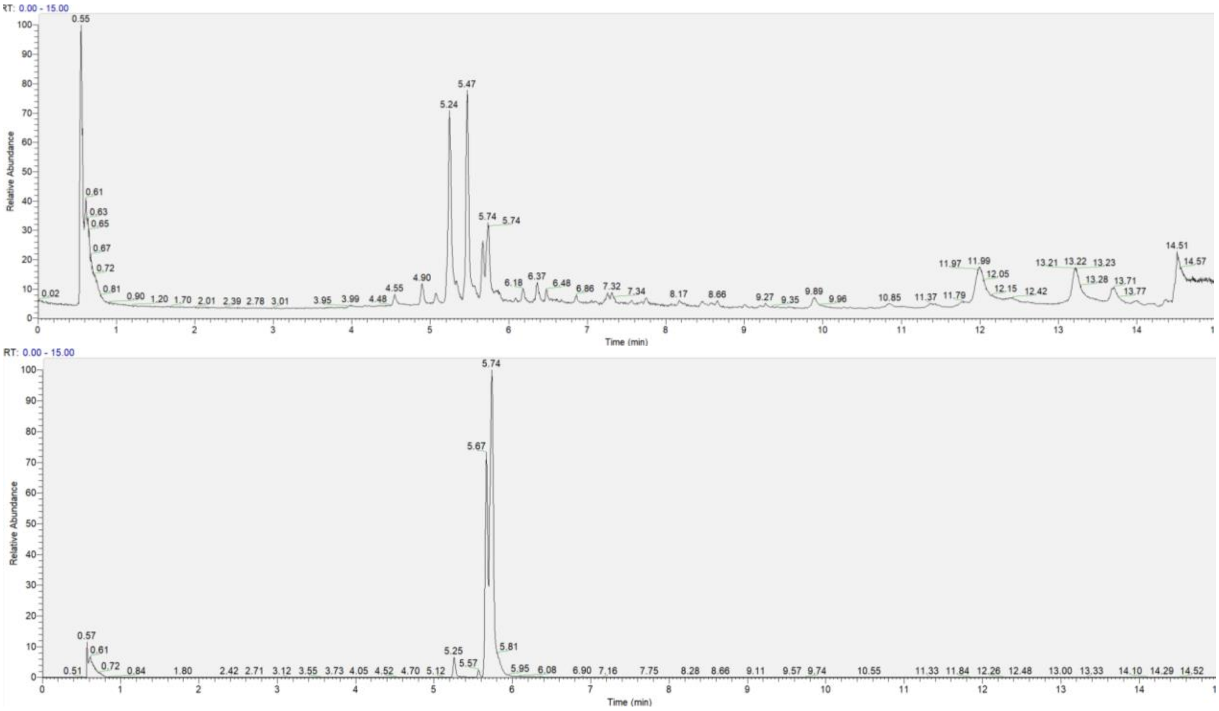

Figure S105: *TIC trace (top) and XIC of alkane **48** by-product parent ion (bottom).*

Table S6: *LC-MS data and peak identities for CRAM ether **10**.*

Apex RT	Start RT	End RT	Area	%Area	*m/z*	Identity
5.28	5.21	5.39	4.872	31.50	299.1143	Title compound **10** isomer
5.50	5.45	5.58	4.674	30.22	299.1143	Title compound **10** isomer
5.77	5.65	5.81	2.016	13.03	269.1033	Demethoxylated parent compound
6.21	6.15	6.29	2.691	17.40	395.13	Unknown
6.51	6.46	6.58	1.214	7.85	343.14	Unknown

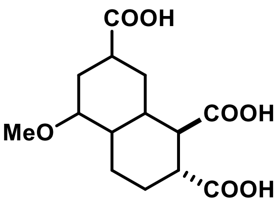

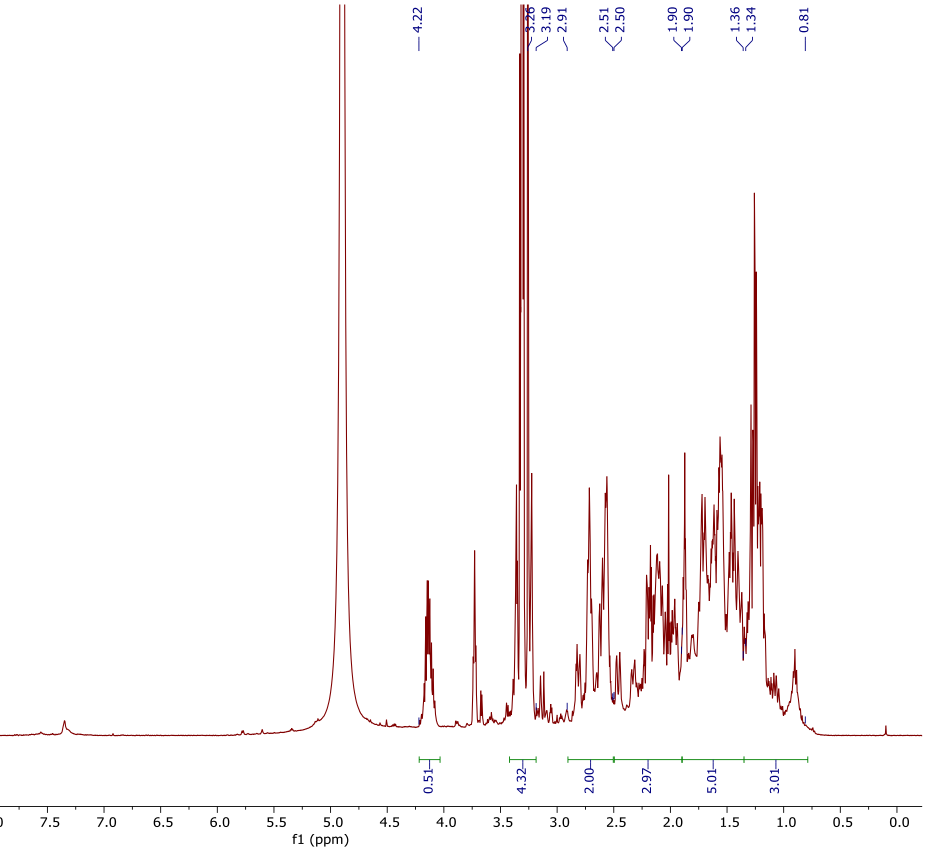

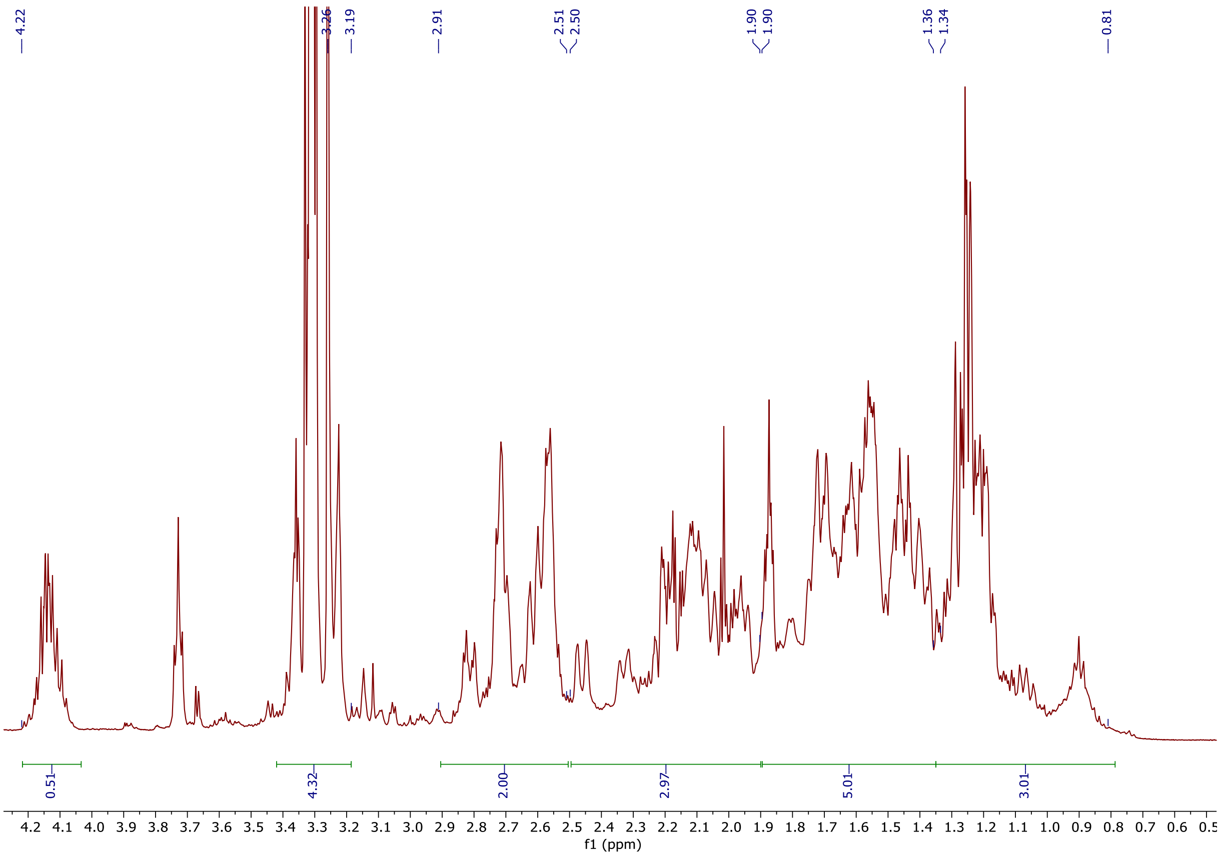

Figure S106: *^{1}H NMR spectra of CRAM ether* ***10*** *(500 MHz, MeOD).*

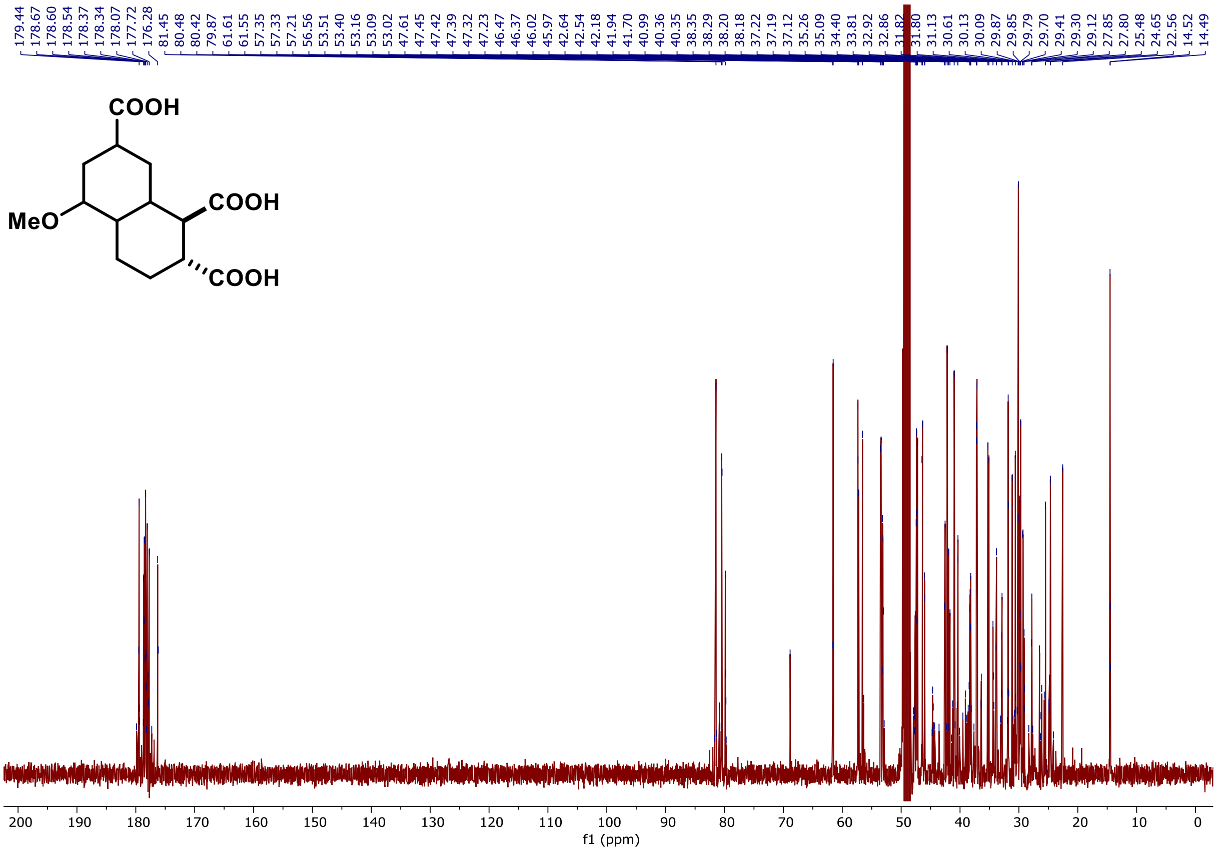
179.44
178.67
178.60
178.54
178.37
178.34
178.07
177.72
176.28
81.45
80.48
80.42
79.87
61.61
61.55
57.35
57.33
57.21
56.56
53.51
53.40
53.16
53.09
53.02
47.61
47.45
47.42
47.39
47.32
47.23
46.47
46.37
46.02
45.97
42.64
42.54
42.18
41.94
41.70
40.99
40.36
40.35
38.35
38.29
38.20
38.18
37.22
37.19
37.12
35.26
35.09
34.40
33.81
32.92
32.86
31.82
31.80
31.13
30.61
30.13
30.09
29.87
29.85
29.79
29.70
29.41
29.30
29.12
27.85
27.80
25.48
24.65
22.56
14.52
14.49
COOH
COOH
COOH
MeO
f1 (ppm)

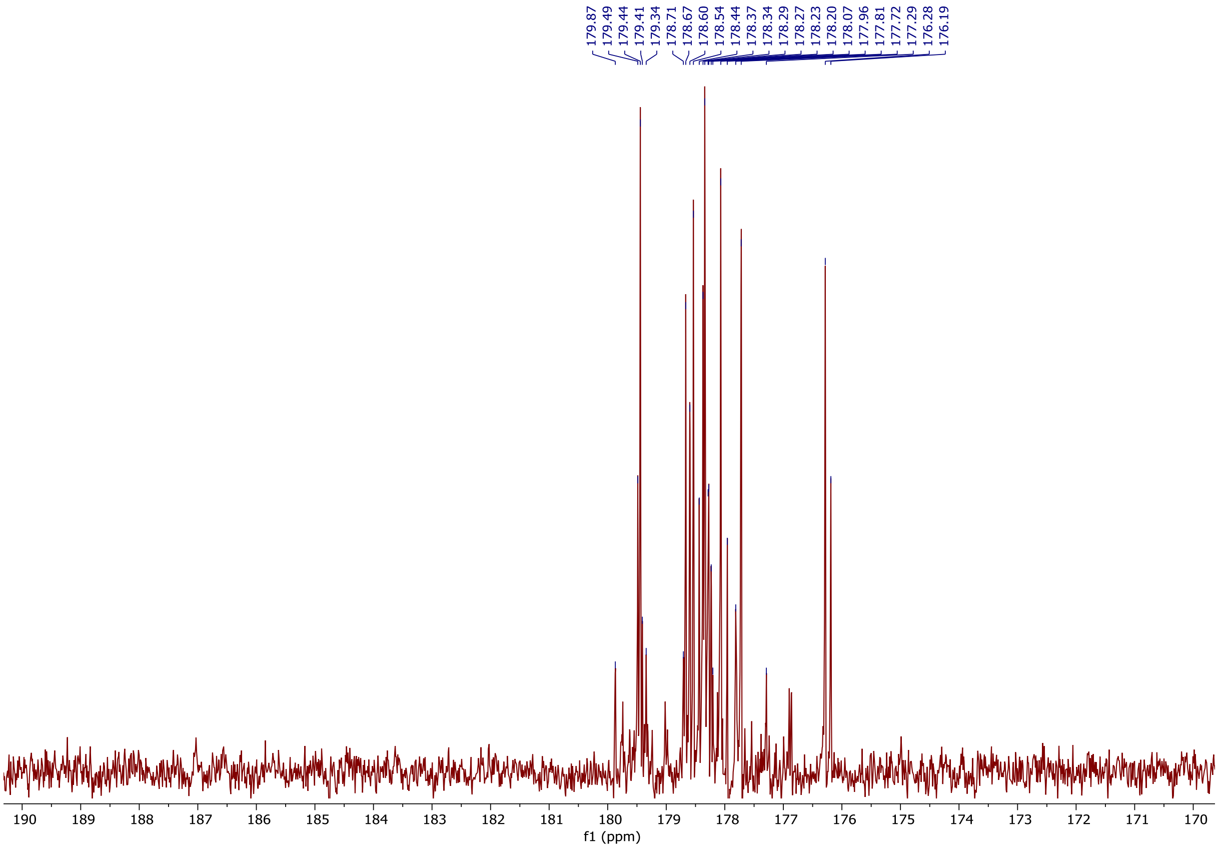
179.87
179.49
179.44
179.41
179.34
178.71
178.67
178.60
178.54
178.44
178.37
178.34
178.29
178.27
178.23
178.20
178.07
177.96
177.81
177.72
177.29
176.28
176.19
f1 (ppm)

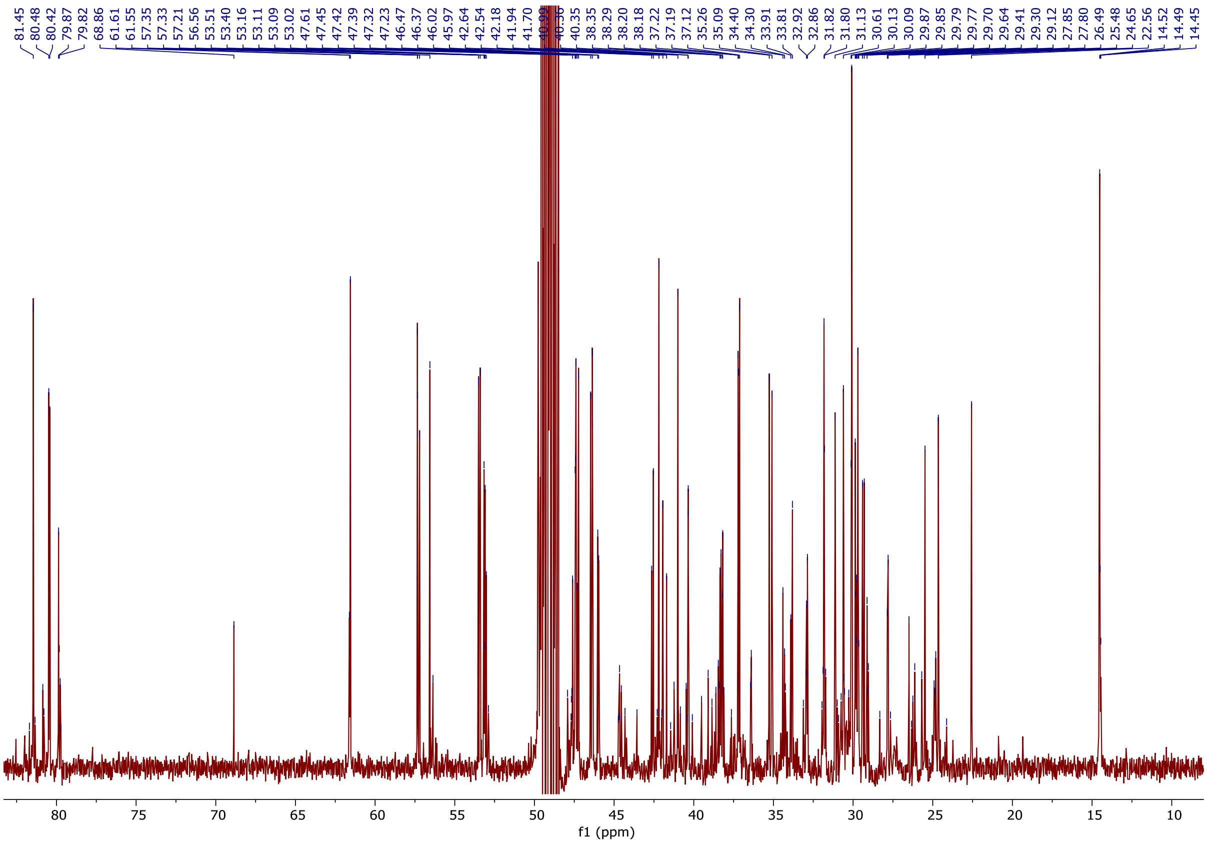

Figure S107: *^{13}C NMR spectra of CRAM ether* ***10*** *(126 MHz, MeOD).*

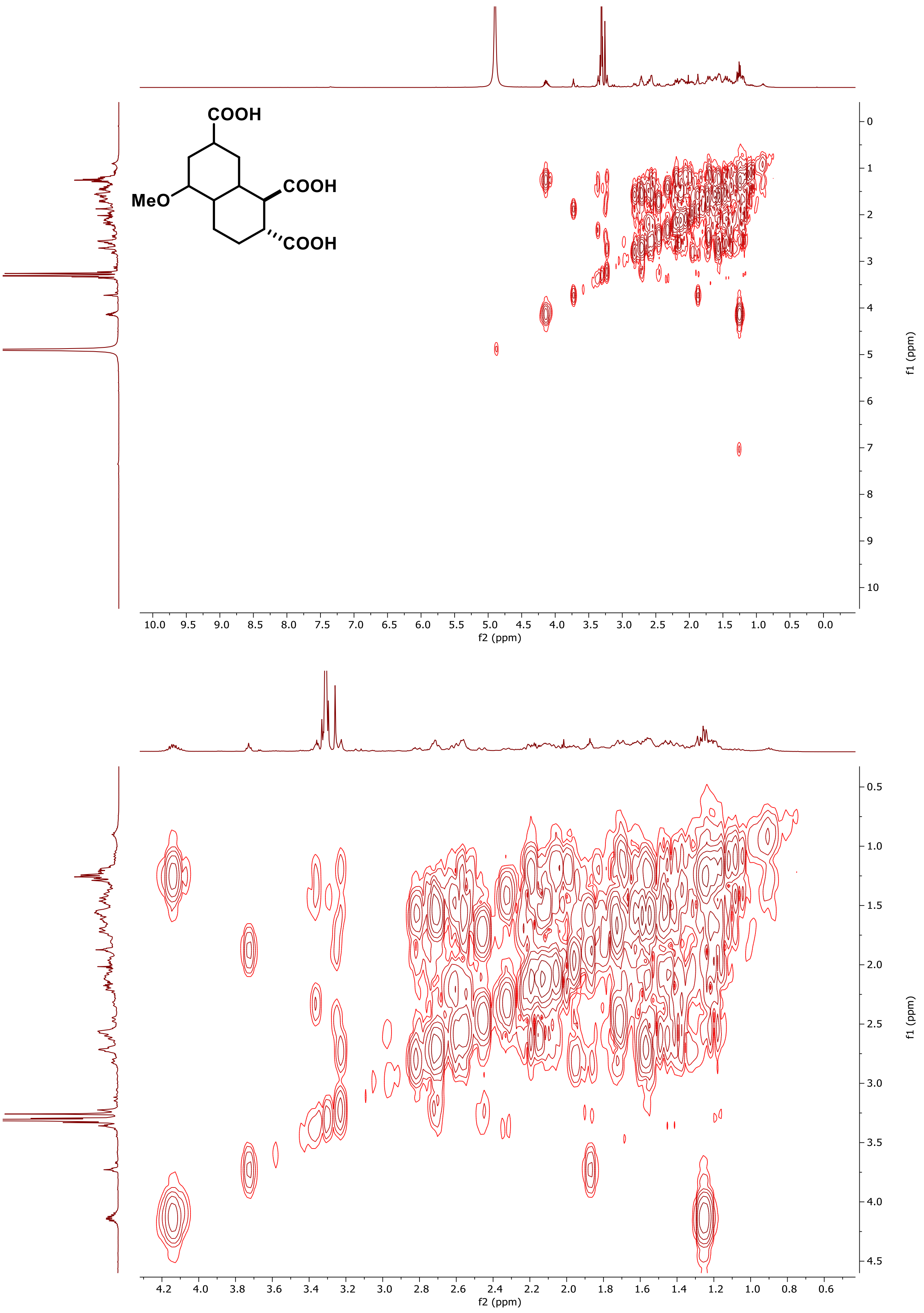

Figure S108: *COSY spectra of CRAM ether* ***10***.

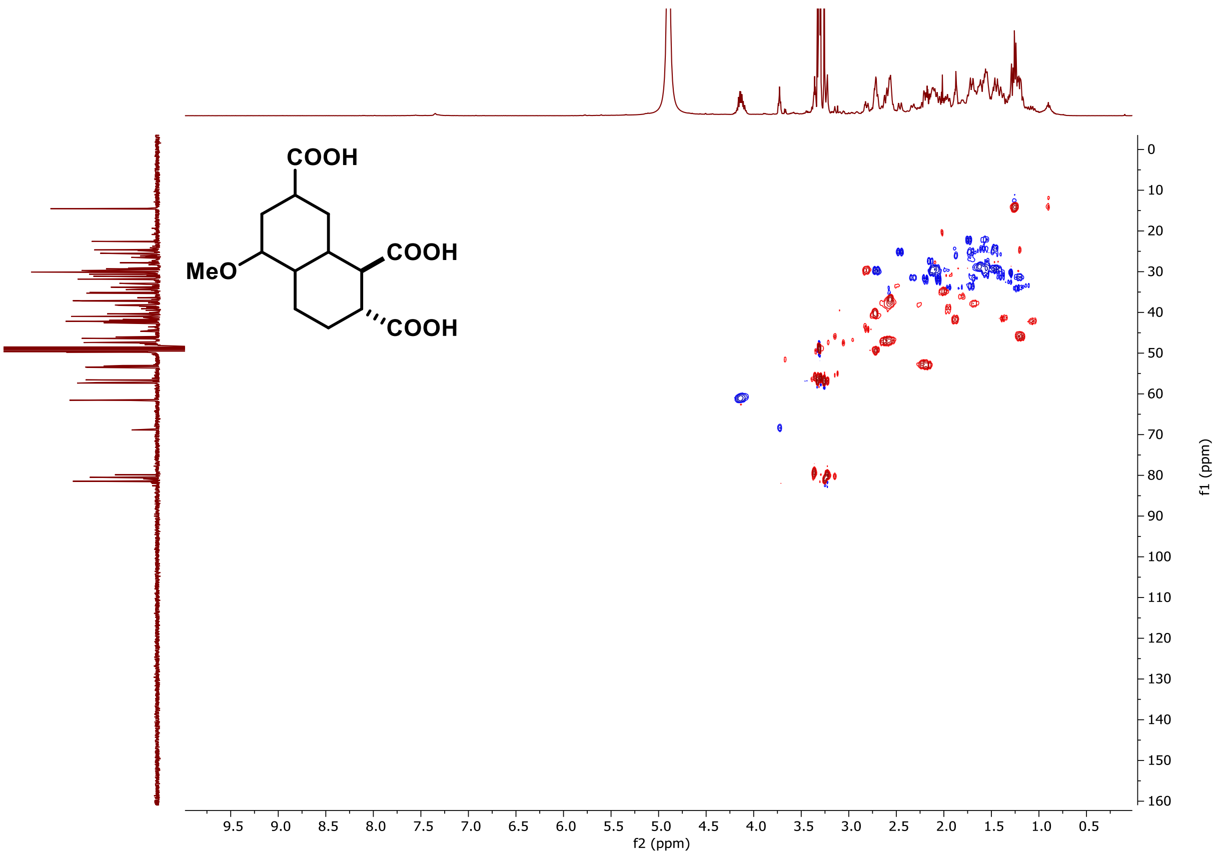

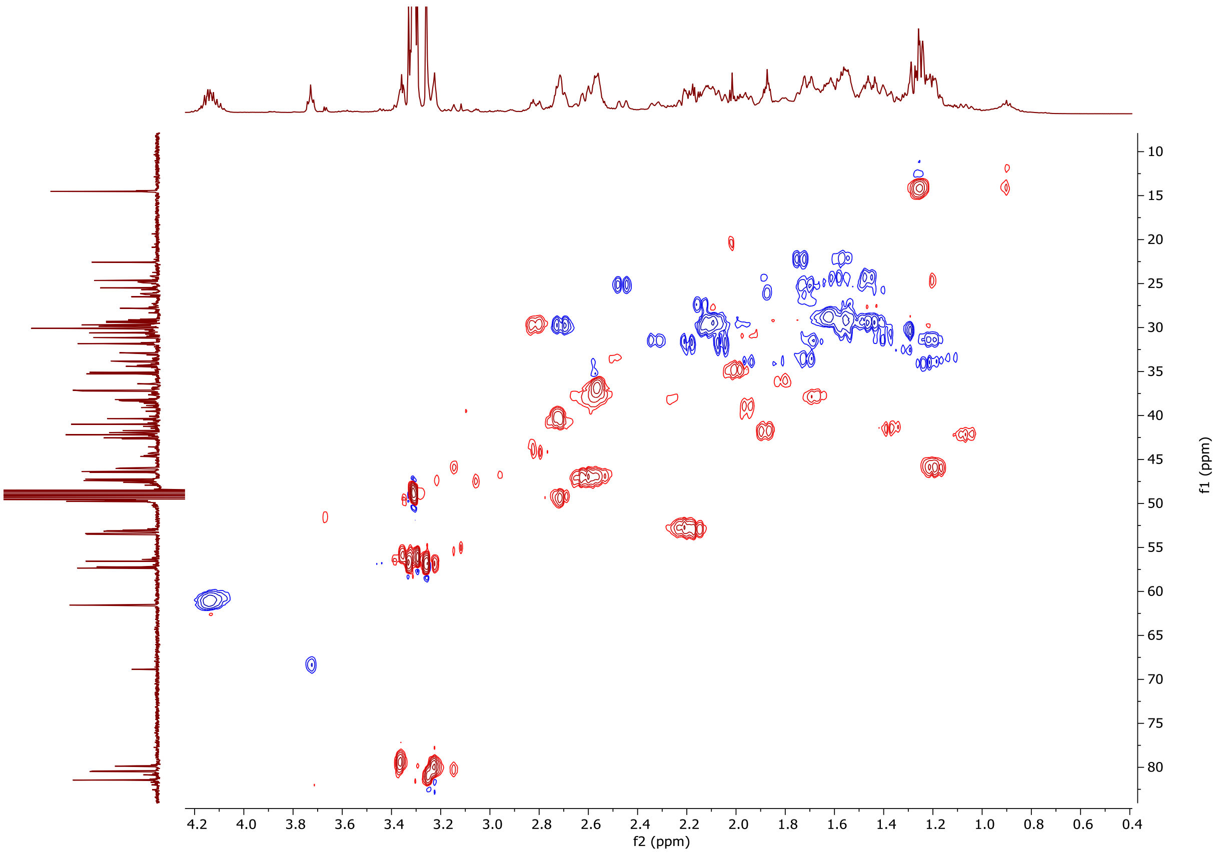

Figure S109: *HSQC spectra of CRAM ether **10**.*

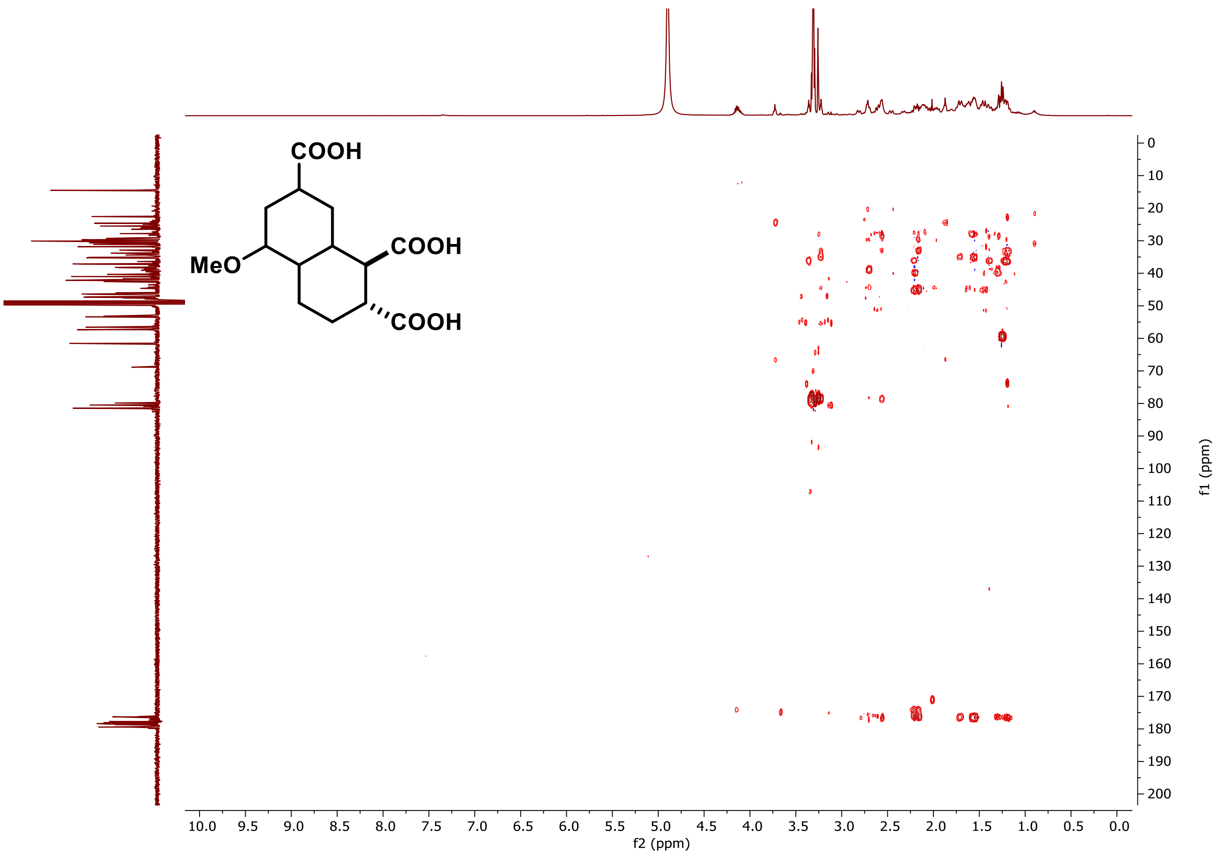
COOH
MeO
COOH
COOH
f2 (ppm)
f1 (ppm)

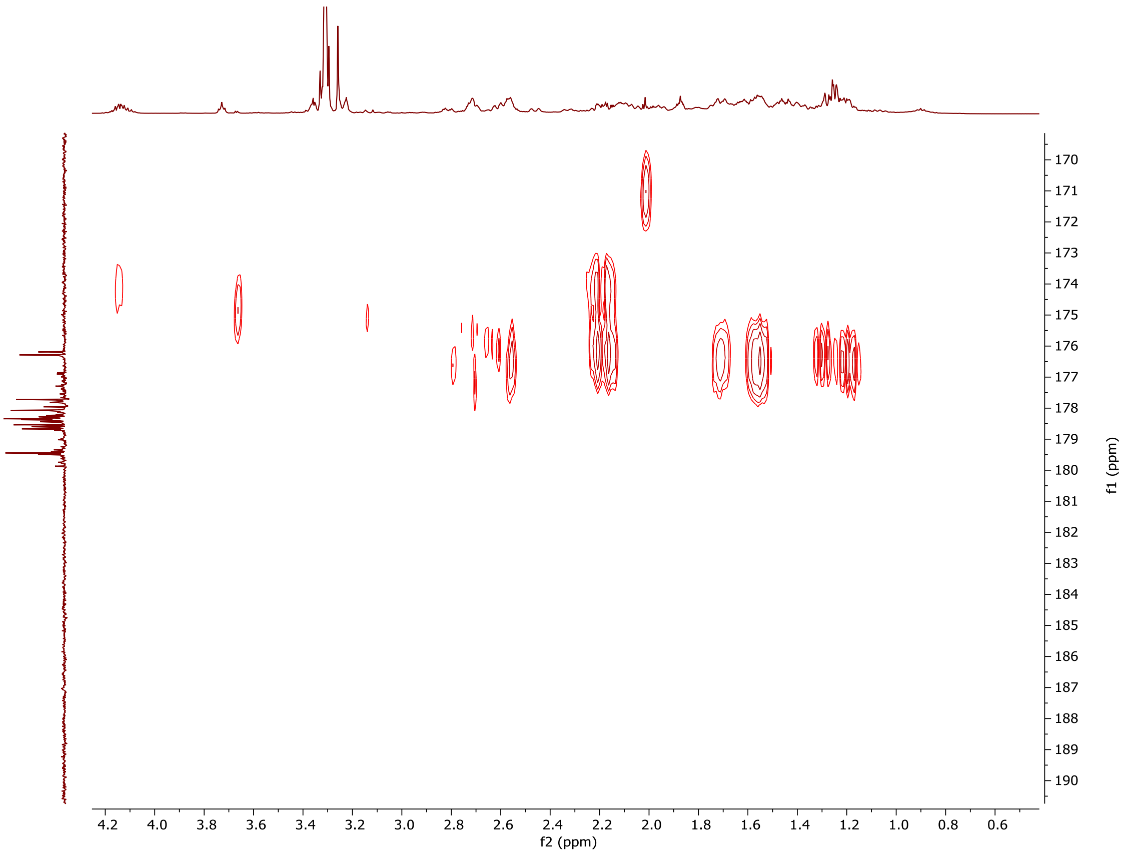
f2 (ppm)
f1 (ppm)

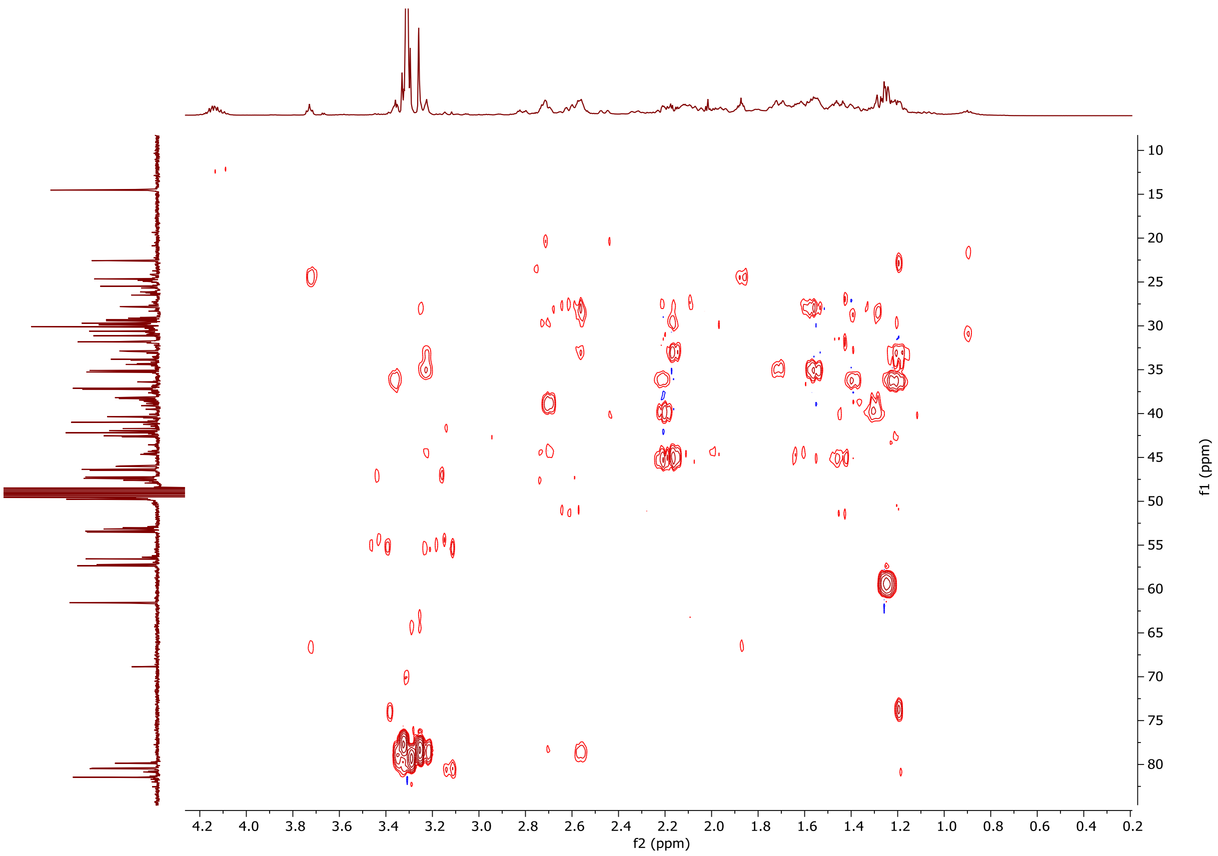

Figure S110: *HMBC spectra of CRAM ether **10**.*

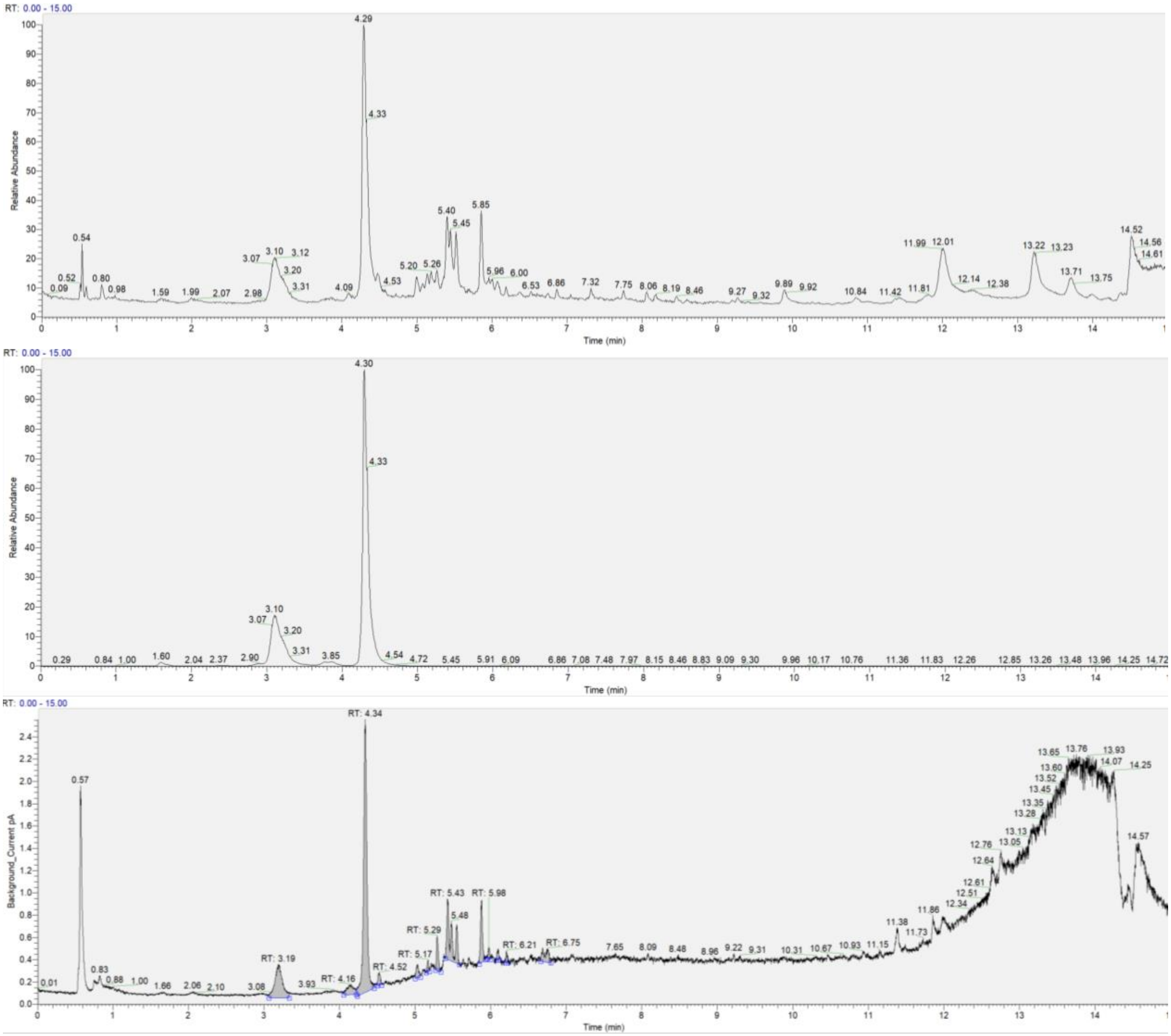

Figure S111: *TIC trace (top), XIC of title compound parent ion (middle), and CAD trace (bottom) of CRAM ketone* ***11*** *derived from single isomer.*

Table S7: *LC-MS data and peak identities for CRAM ketone* ***11*** *derived from single isomer.*

Apex RT	Start RT	End RT	Area	%Area	*m/z*	Identity
3.19	3.06	3.33	2.154	14.13	283.0830	Title compound **11** isomer
4.34	4.23	4.46	7.596	49.83	283.0829	Title compound **11** isomer
4.52	4.50	4.56	0.260	1.71	300.1180	Unknown
5.03	5.00	5.07	0.300	1.96	300.1180	Unknown
5.17	5.15	5.19	0.153	1.00	313.0942	Unknown
5.29	5.27	5.34	0.533	3.50	579.1727	Unknown
5.43	5.40	5.59	2.210	14.50	300.1180	Unknown
5.88	5.85	5.93	1.022	6.70	300.1180	Unknown
5.98	5.96	5.99	0.158	1.03	300.1180	Unknown
6.10	6.08	6.11	0.121	0.80	311.1150	Unknown
6.21	6.19	6.23	0.146	0.96	317.1526	Unknown
6.75	6.66	6.80	0.590	3.87	215.1292	Unknown

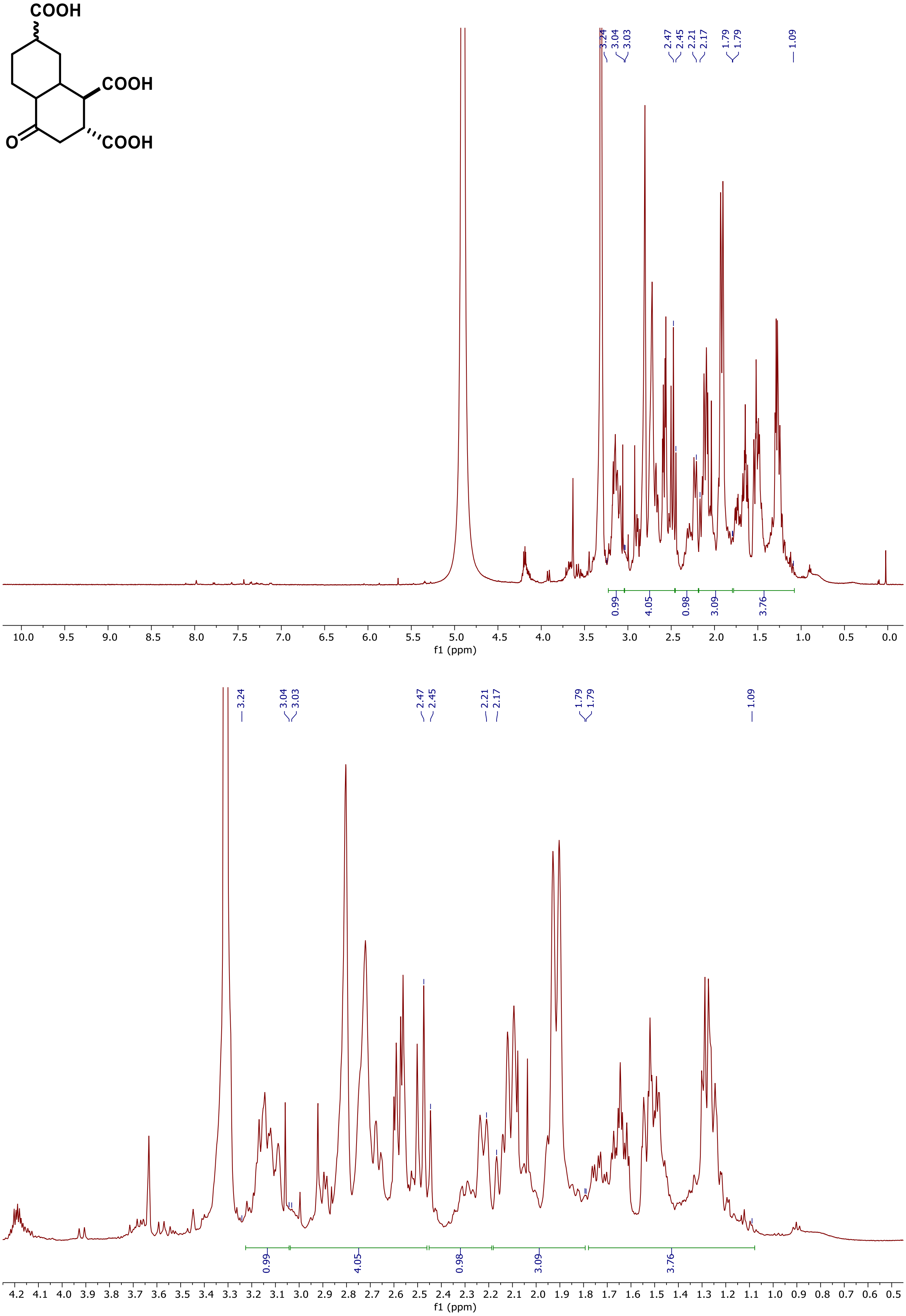

Figure S112: *^{1}H NMR spectra of CRAM ketone* ***11*** *derived from single isomer (500 MHz, MeOD).*

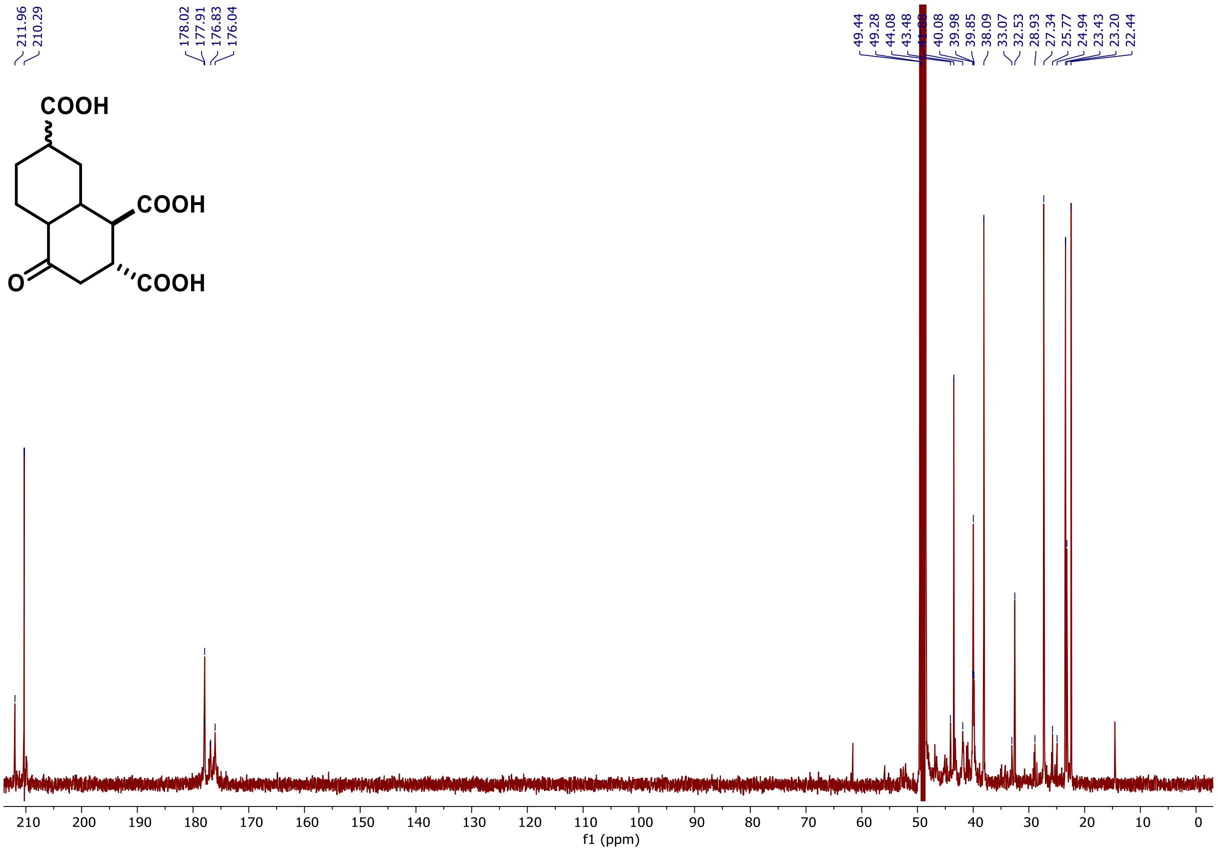
COOH
COOH
O
COOH
211.96
210.29
178.02
177.91
176.83
176.04
49.44
49.28
44.08
43.48
[illegible]
40.08
39.98
39.85
38.09
33.07
32.53
28.93
27.34
25.77
24.94
23.43
23.20
22.44
f1 (ppm)

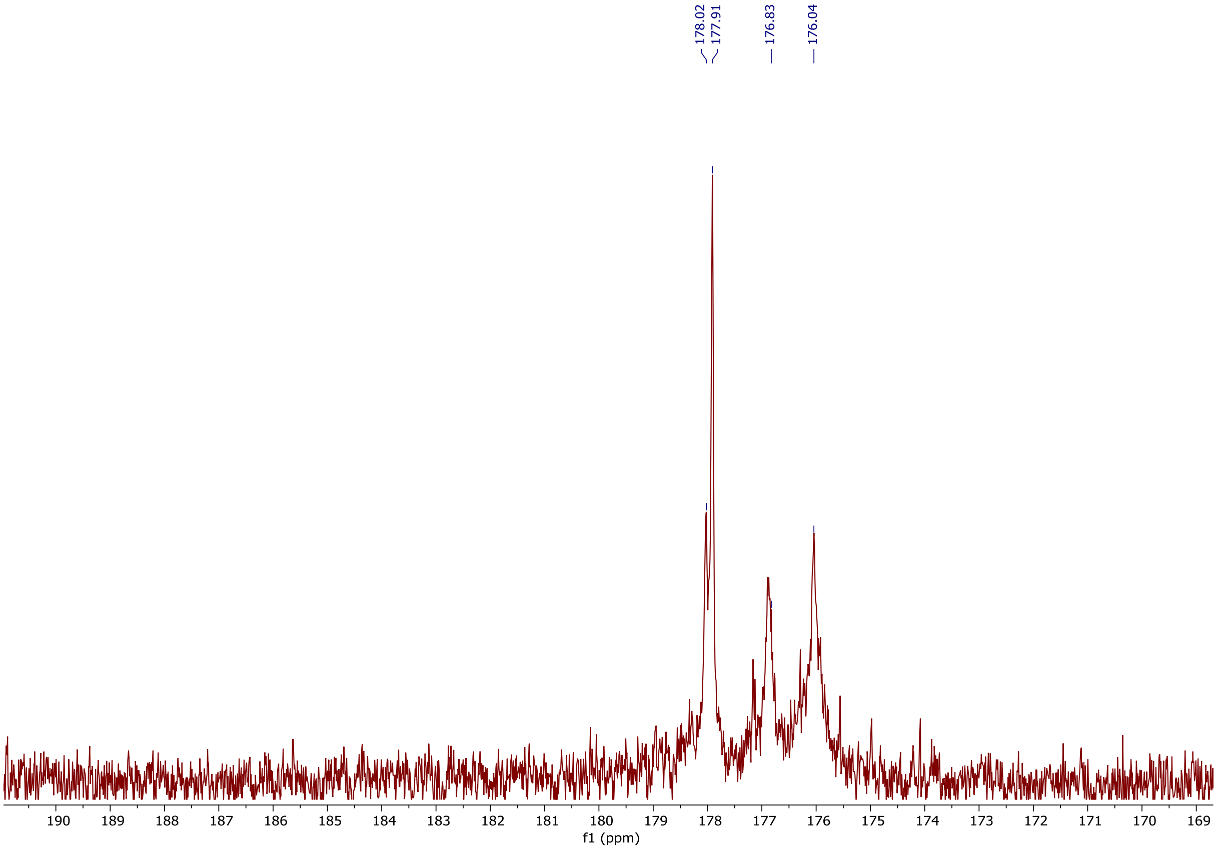
178.02
177.91
176.83
176.04
f1 (ppm)

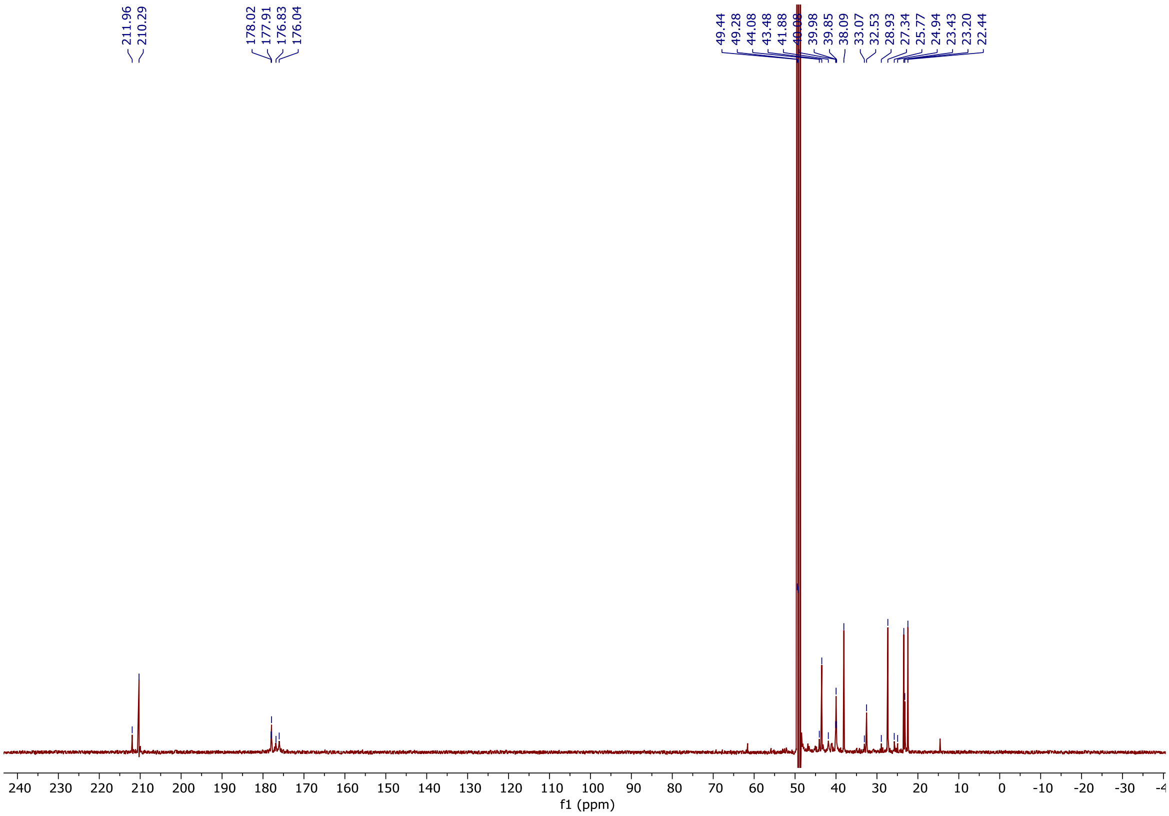

Figure S113: *^{13}C NMR spectra of CRAM ketone* ***11*** *derived from single isomer (126 MHz, MeOD).*

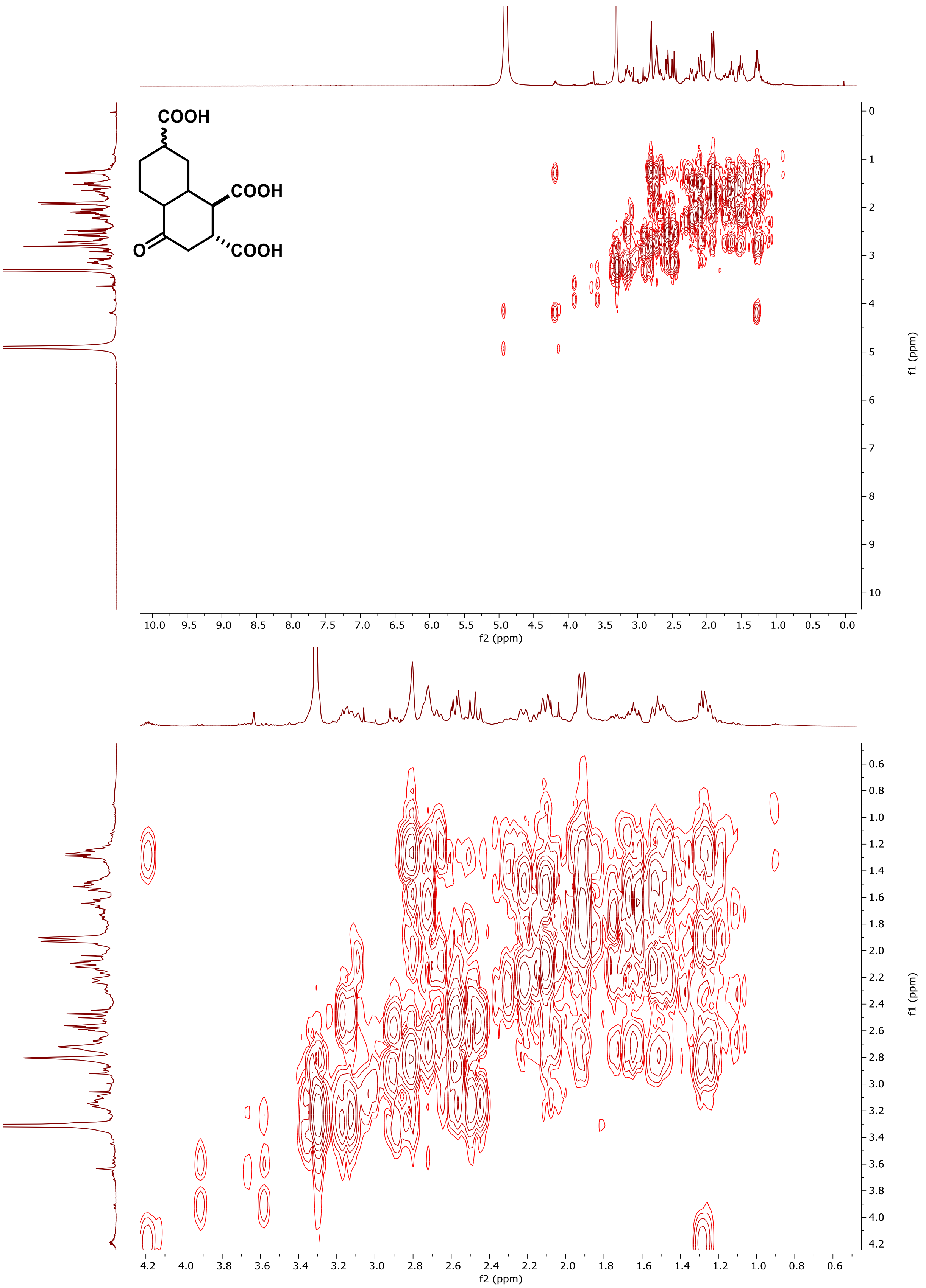

Figure S114: *COSY spectra of CRAM ketone* ***11*** *derived from single isomer.*

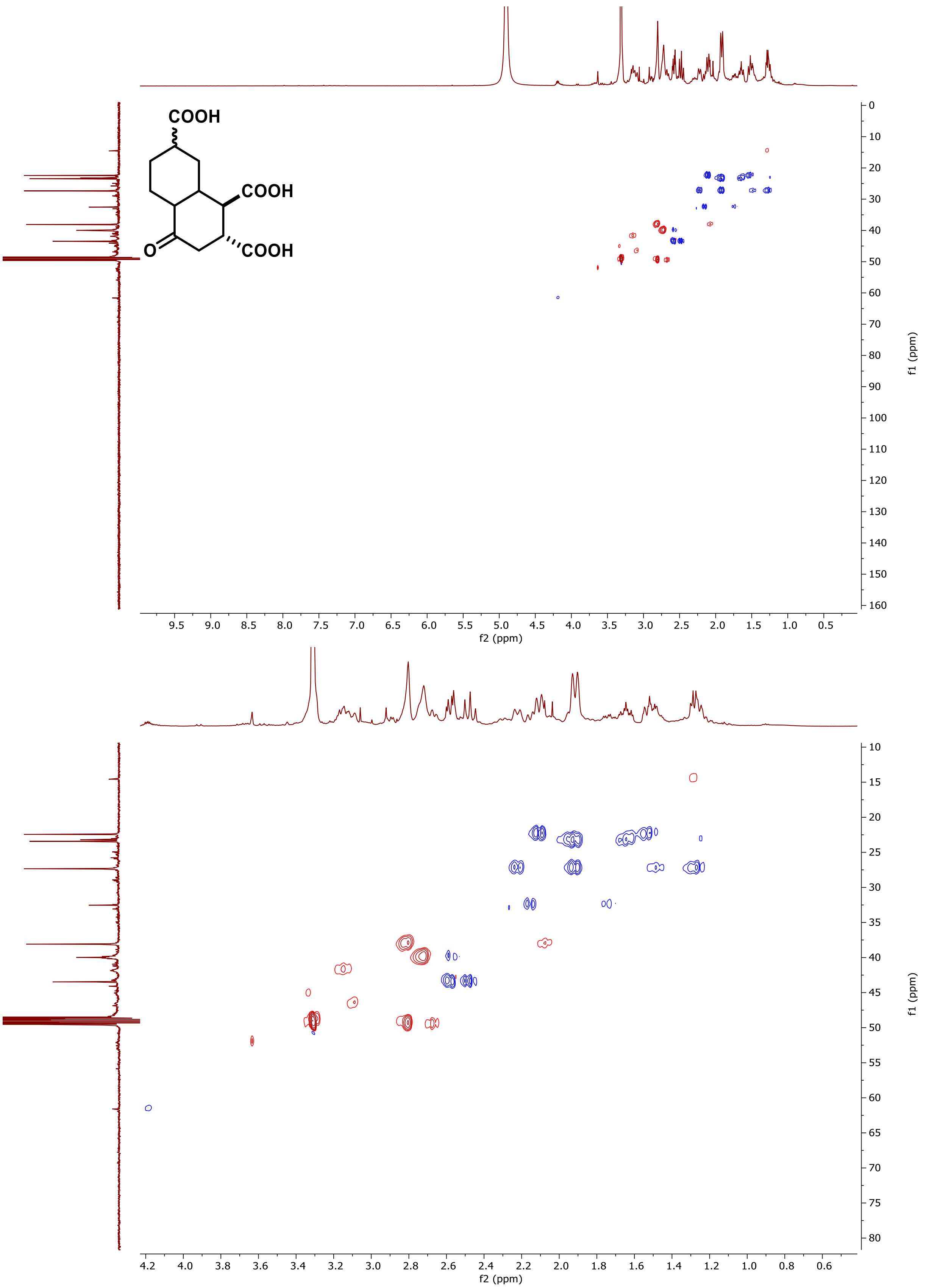

Figure S115: *HSQC spectra of CRAM ketone **11** derived from single isomer.*

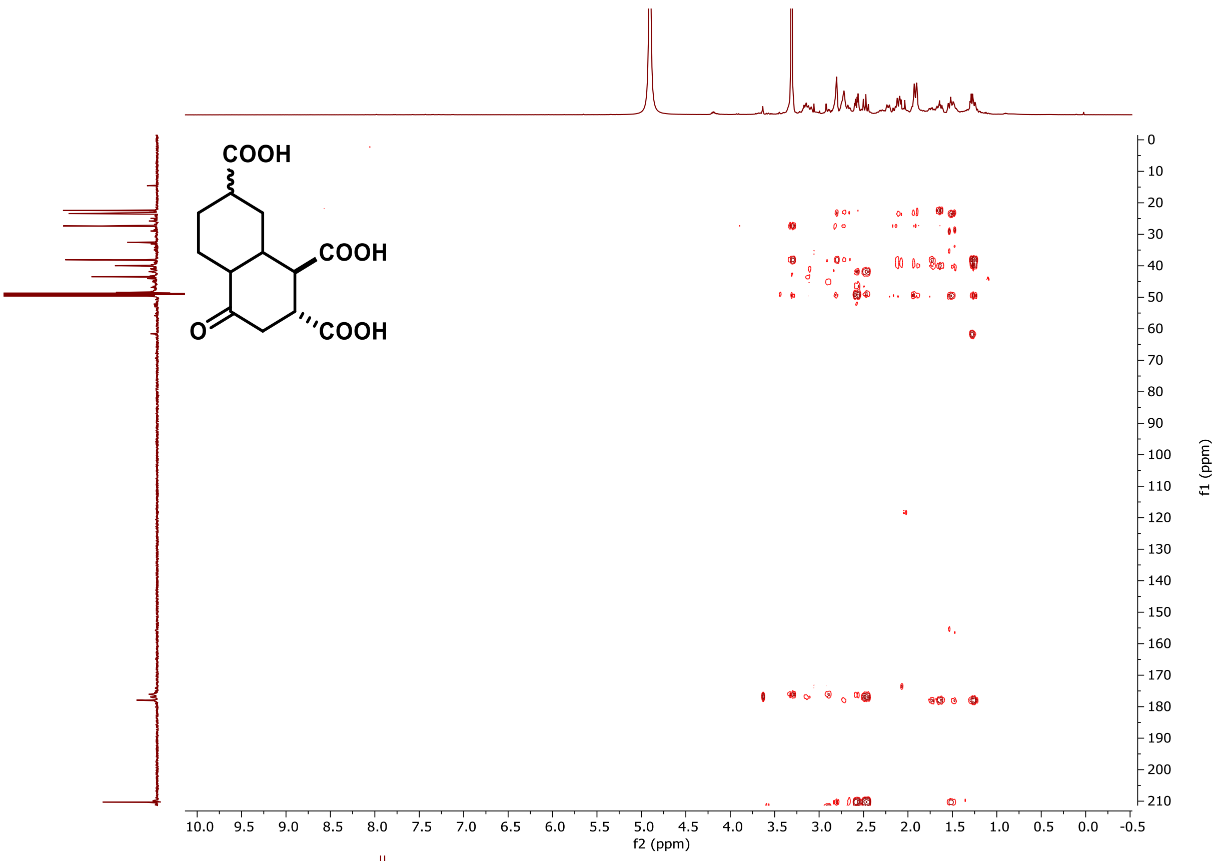
COOH
COOH
O
COOH
f2 (ppm)
f1 (ppm)

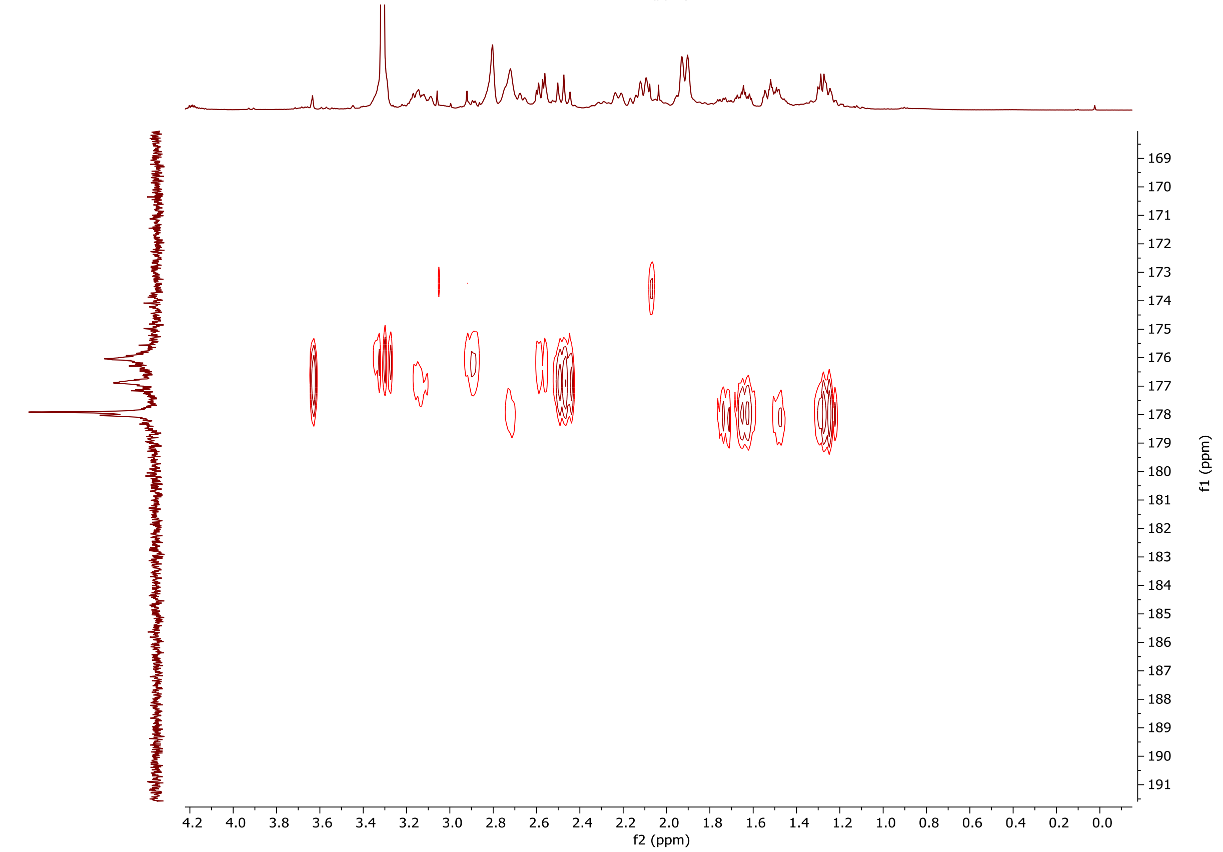
f2 (ppm)
f1 (ppm)

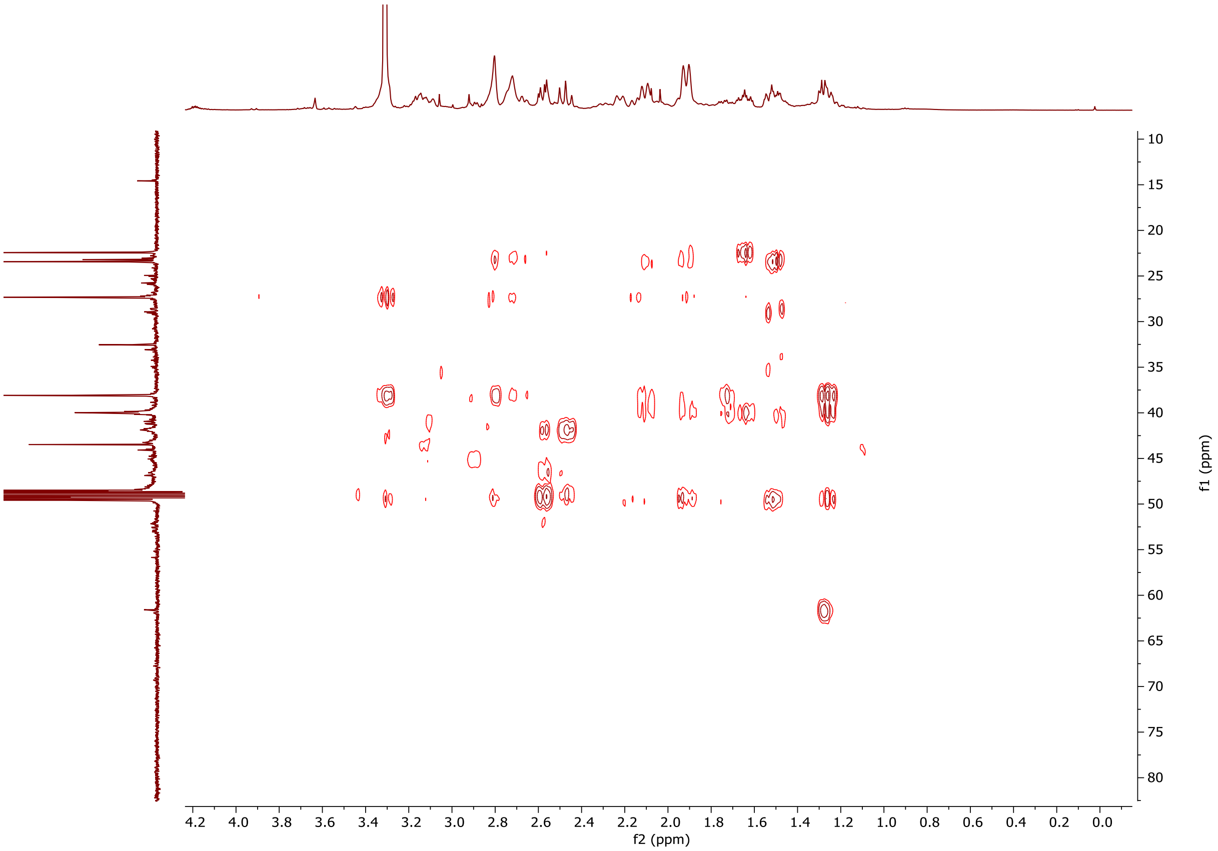

Figure S116: *HMBC spectra of CRAM ketone* ***11*** *derived from single isomer.*

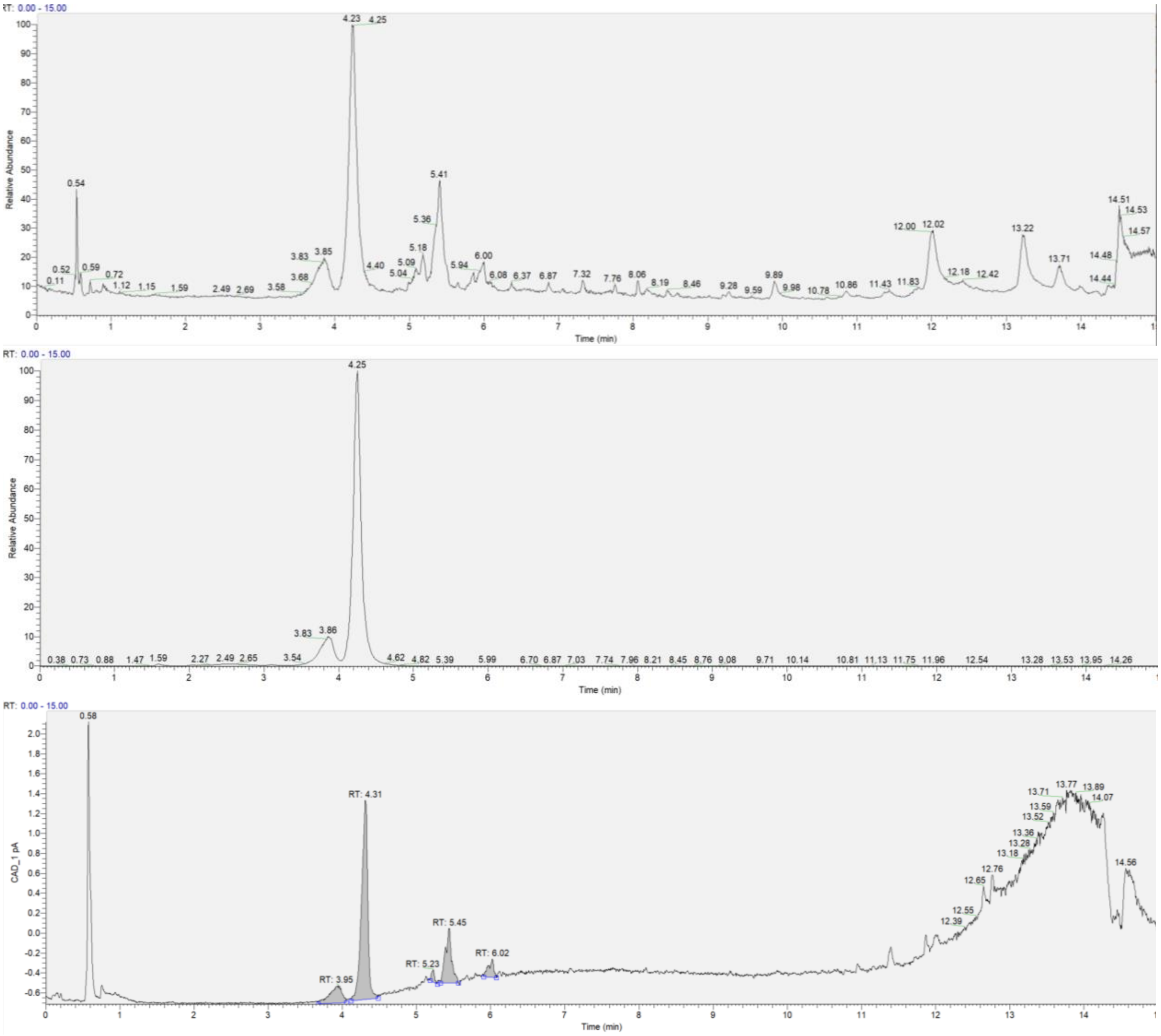

Figure S117: *TIC trace (top), XIC of title compound parent ion (middle), and CAD trace (bottom) of CRAM ketone **11** derived from mixture of diastereomers.*

Table S8: *LC-MS data and peak identities for CRAM ketone **11** derived from mixture of diastereomers.*

Apex RT	Start RT	End RT	Area	%Area	*m/z*	Identity
3.95	3.68	4.05	2.041	11.25	283.0833	Title compound **11** isomer
4.31	4.12	4.48	11.627	64.11	283.0830	Title compound **11** isomer
5.23	5.18	5.28	0.349	1.92	300.1179<br>301.1234	Unknown
5.45	5.32	5.56	3.171	17.49	300.1179<br>301.1234	Unknown
6.02	5.90	6.08	0.947	5.22	317.1526<br>318.1582	Unknown

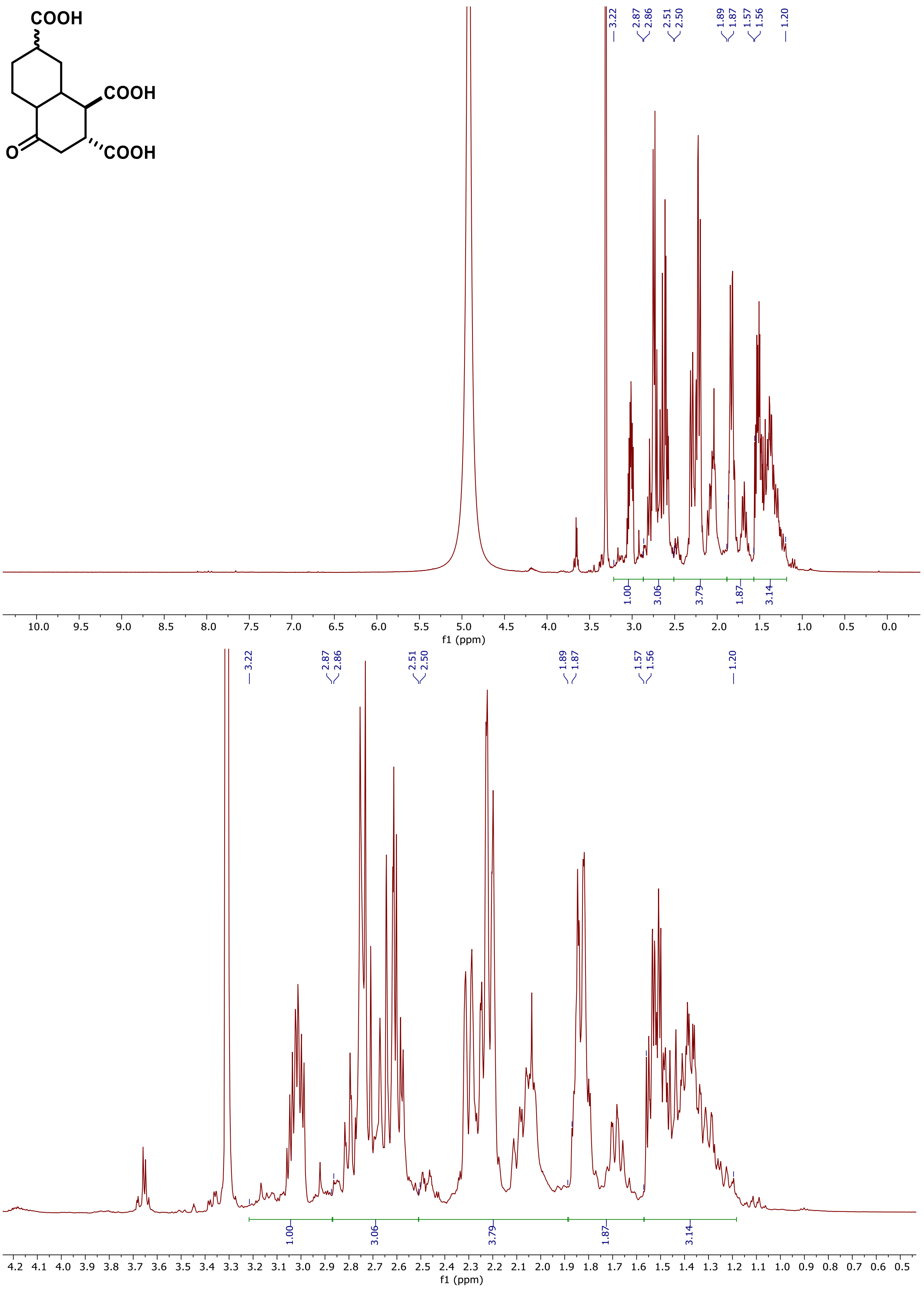

Figure S118: *^{1}H NMR spectra of CRAM ketone **11** derived from mixture of diastereomers (400 MHz, MeOD).*

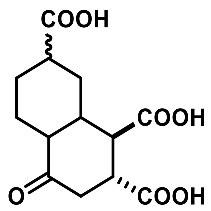

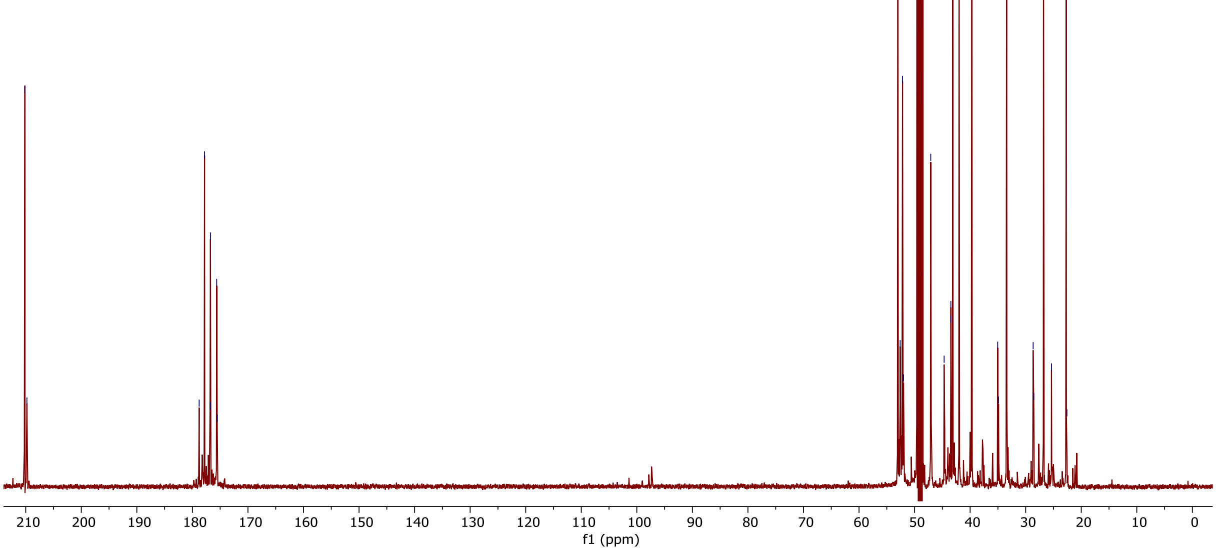

210.15
209.79
178.78
177.81
176.83
176.75
175.63
175.53
53.02
52.59
52.17
51.99
47.09
44.68
43.47
43.44
43.14
41.99
39.69
35.04
34.89
33.42
28.66
28.55
26.80
25.36
22.70
22.58
f1 (ppm)

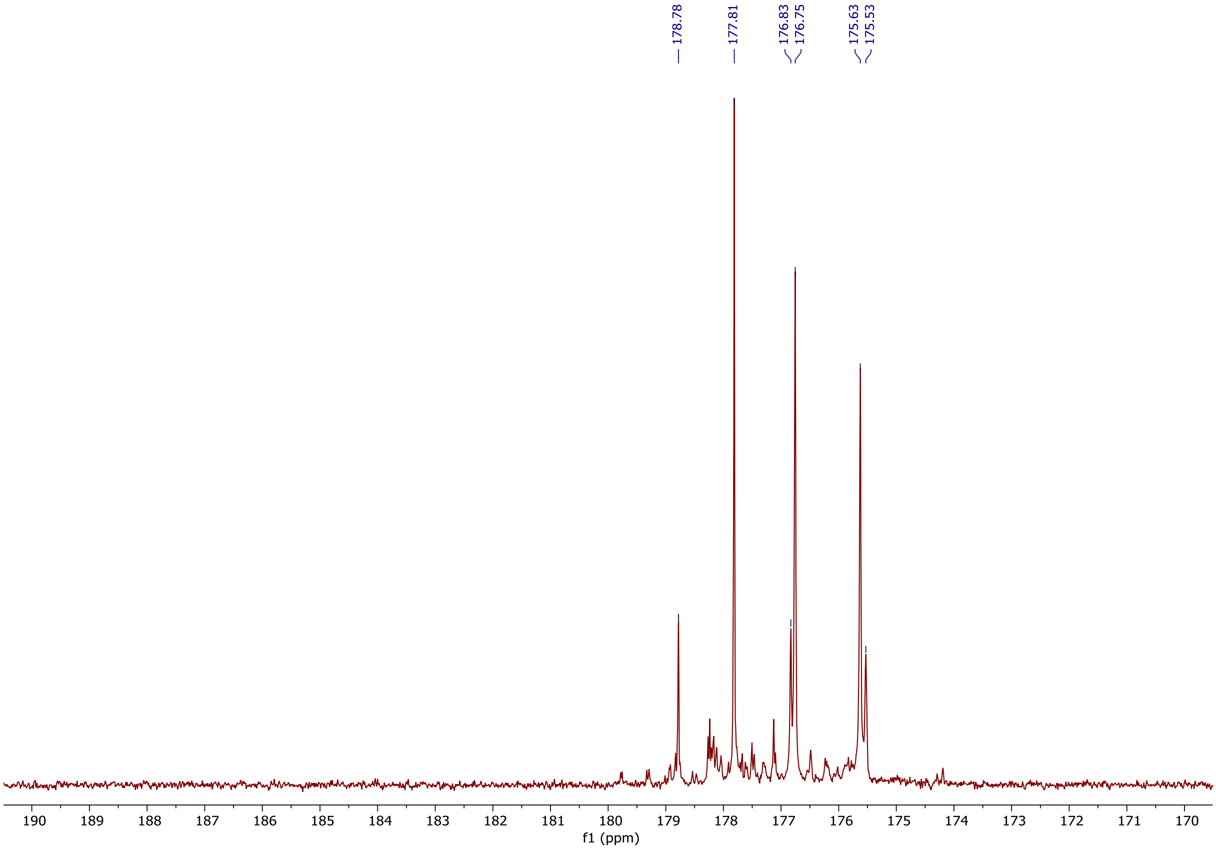

178.78
177.81
176.83
176.75
175.63
175.53
f1 (ppm)

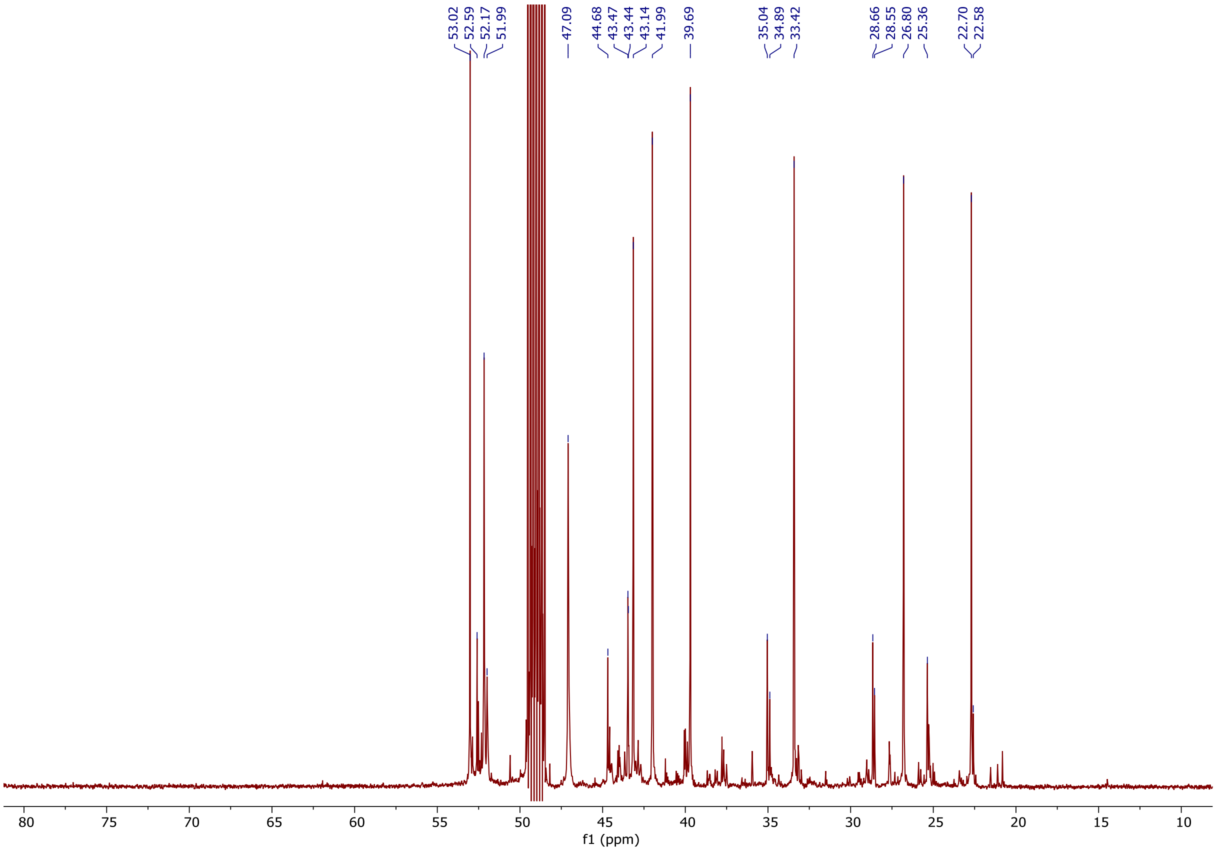

Figure S119: *^{13}C NMR spectra of CRAM ketone* ***11*** *derived from mixture of diastereomers (101 MHz, MeOD).*

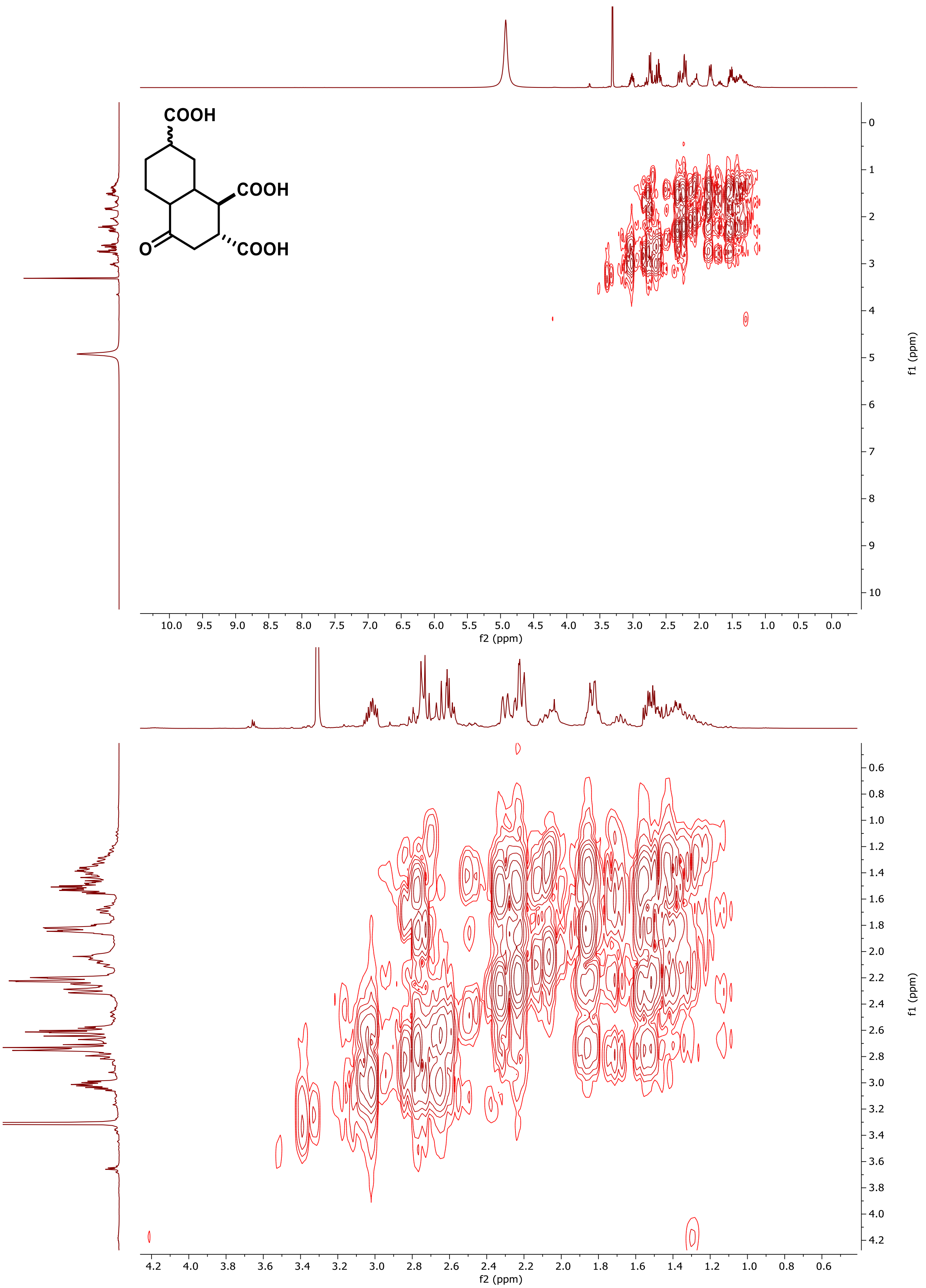

Figure S120: *COSY spectra of CRAM ketone **11** derived from mixture of diastereomers.*

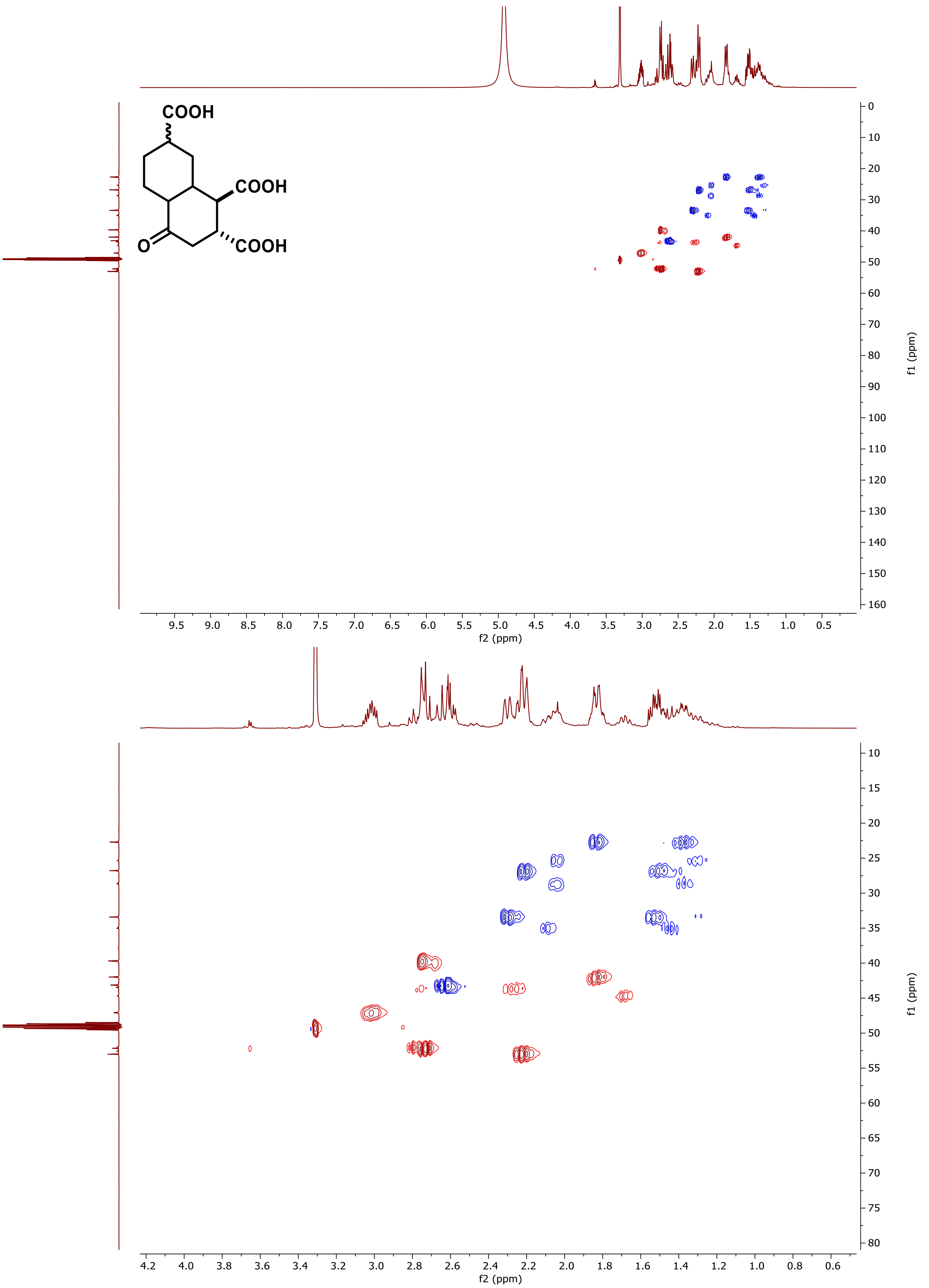

Figure S121: *HSQC spectra of CRAM ketone* ***11*** *derived from mixture of diastereomers.*

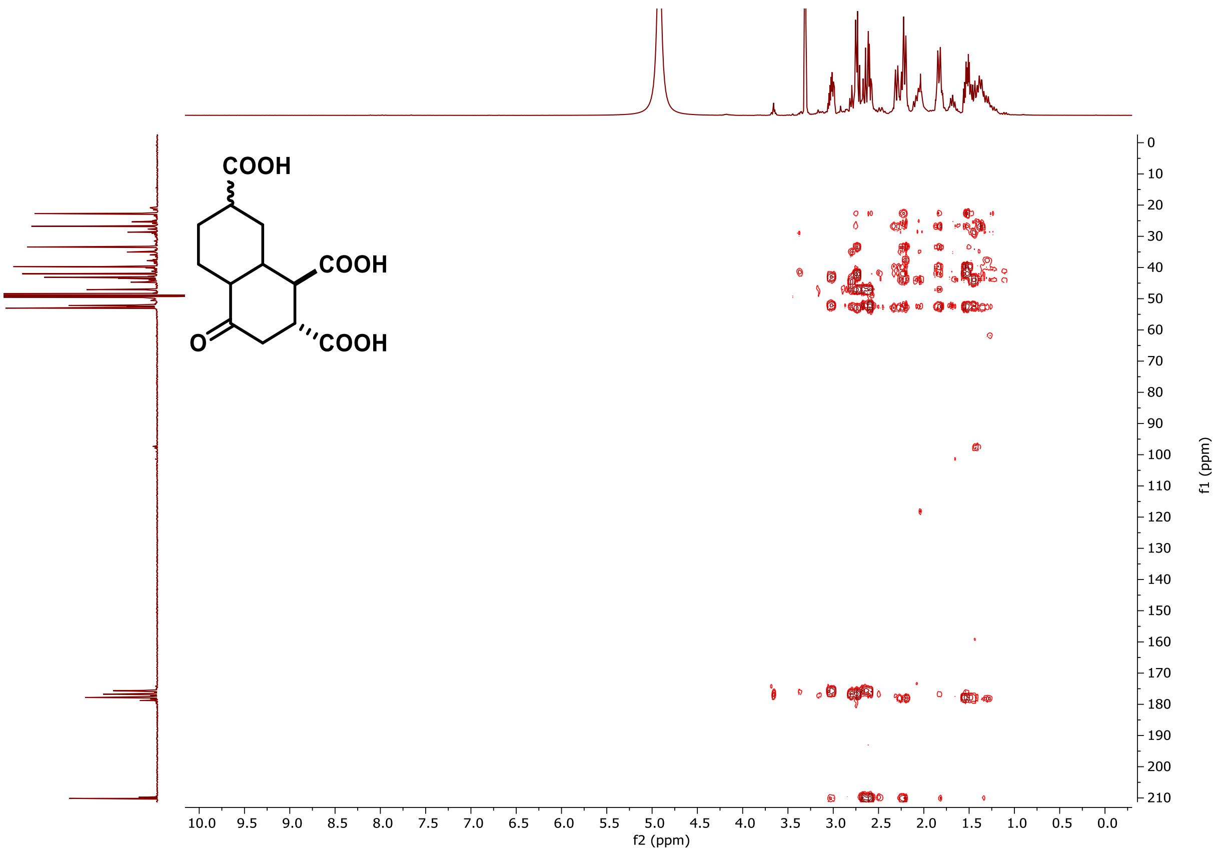
COOH
COOH
O
COOH
f2 (ppm)
f1 (ppm)

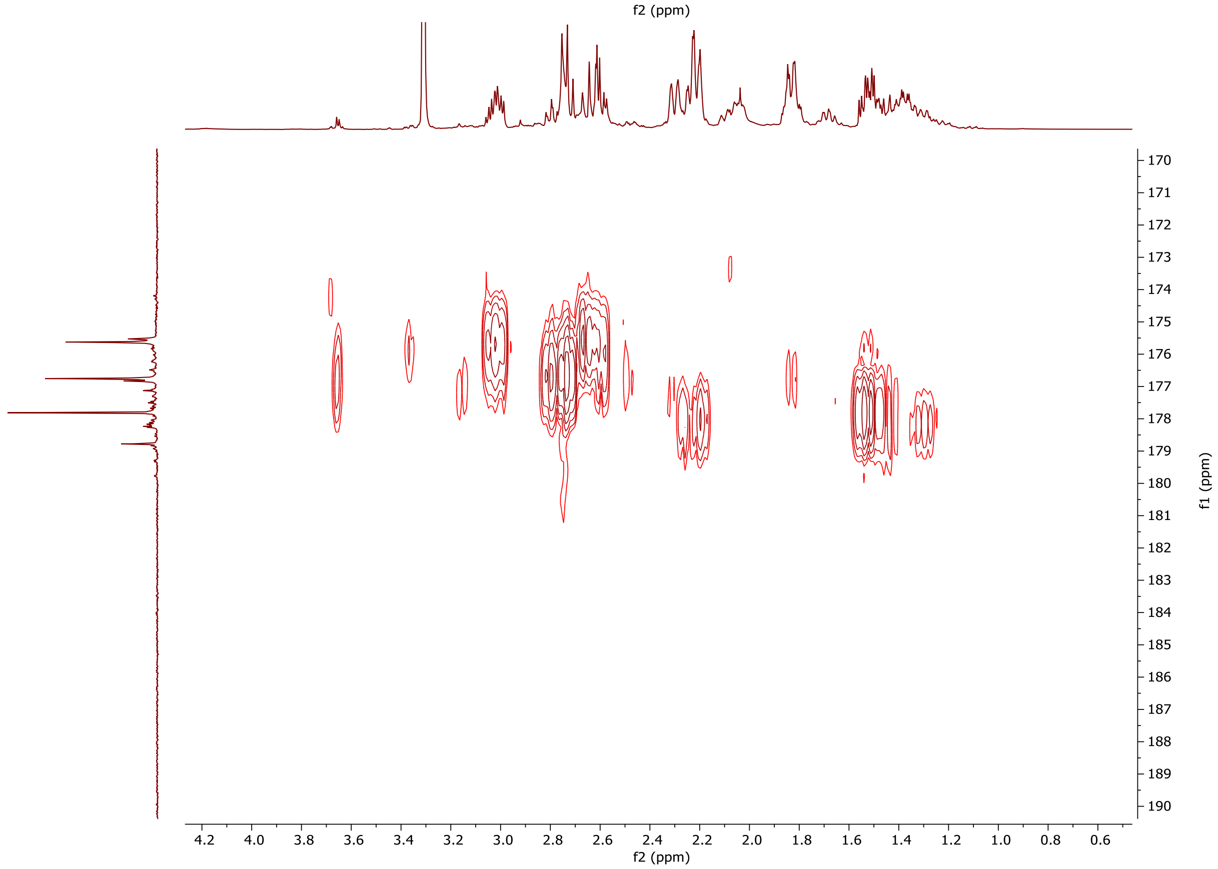
f2 (ppm)
f1 (ppm)

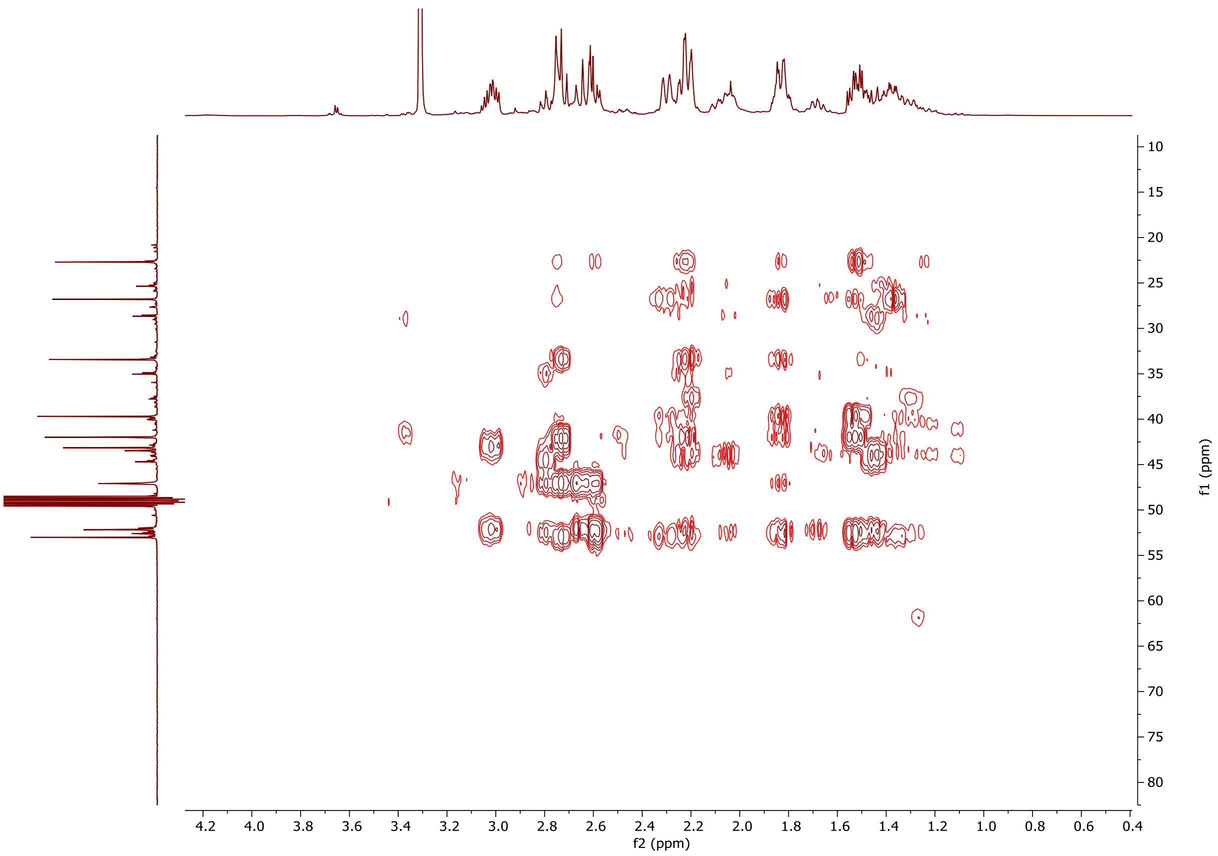

Figure S122: *HMBC spectra of CRAM ketone **11** derived from mixture of diastereomers.*

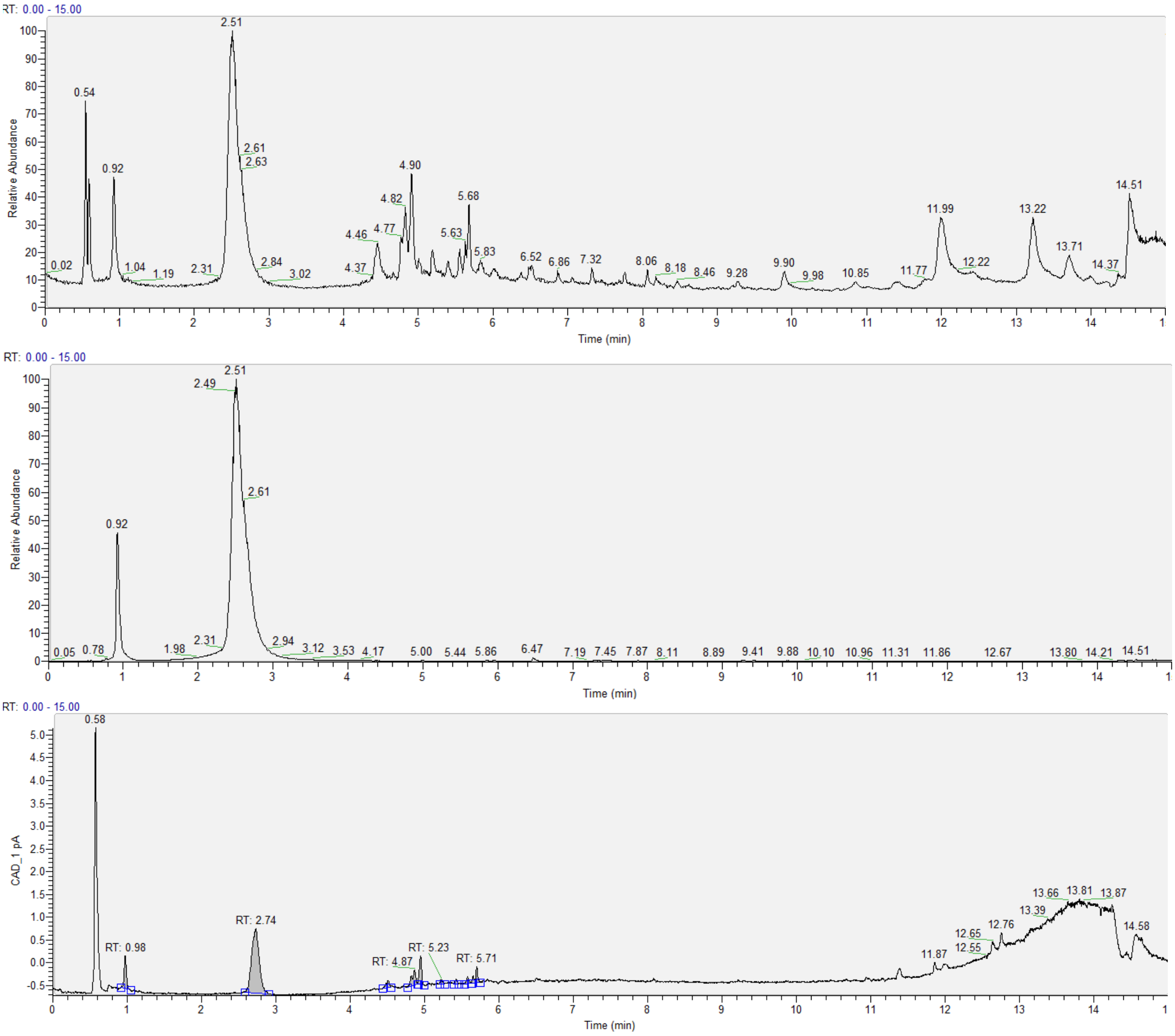

Figure S123: *TIC trace (top), XIC of title compound parent ion (middle), and CAD trace (bottom) of CRAM* α-hydroxy ketone ***12***.

Table S9: *LC-MS data and peak identities for CRAM* α-hydroxy ketone ***12***.

Apex RT	Start RT	End RT	Area	%Area	*m/z*	Identity
0.98	0.92	1.05	1.537	9.53	299.0782	Title compound **12** isomer
2.74	2.59	2.90	10.042	62.26	299.0780	Title compound **12** isomer
4.51	4.44	4.55	0.452	2.80	313.0942	Unknown
4.87	4.78	4.91	1.151	7.13	281.0673	Unknown
4.95	4.92	4.99	1.361	8.44	313.0943	Unknown
5.23	5.21	5.27	0.194	1.20	313.0943	Unknown
5.43	5.39	5.46	0.199	1.23	-	Unknown
5.59	5.54	5.63	0.343	2.13	-	Unknown
5.71	5.63	5.75	0.849	5.27	317.0444	Unknown

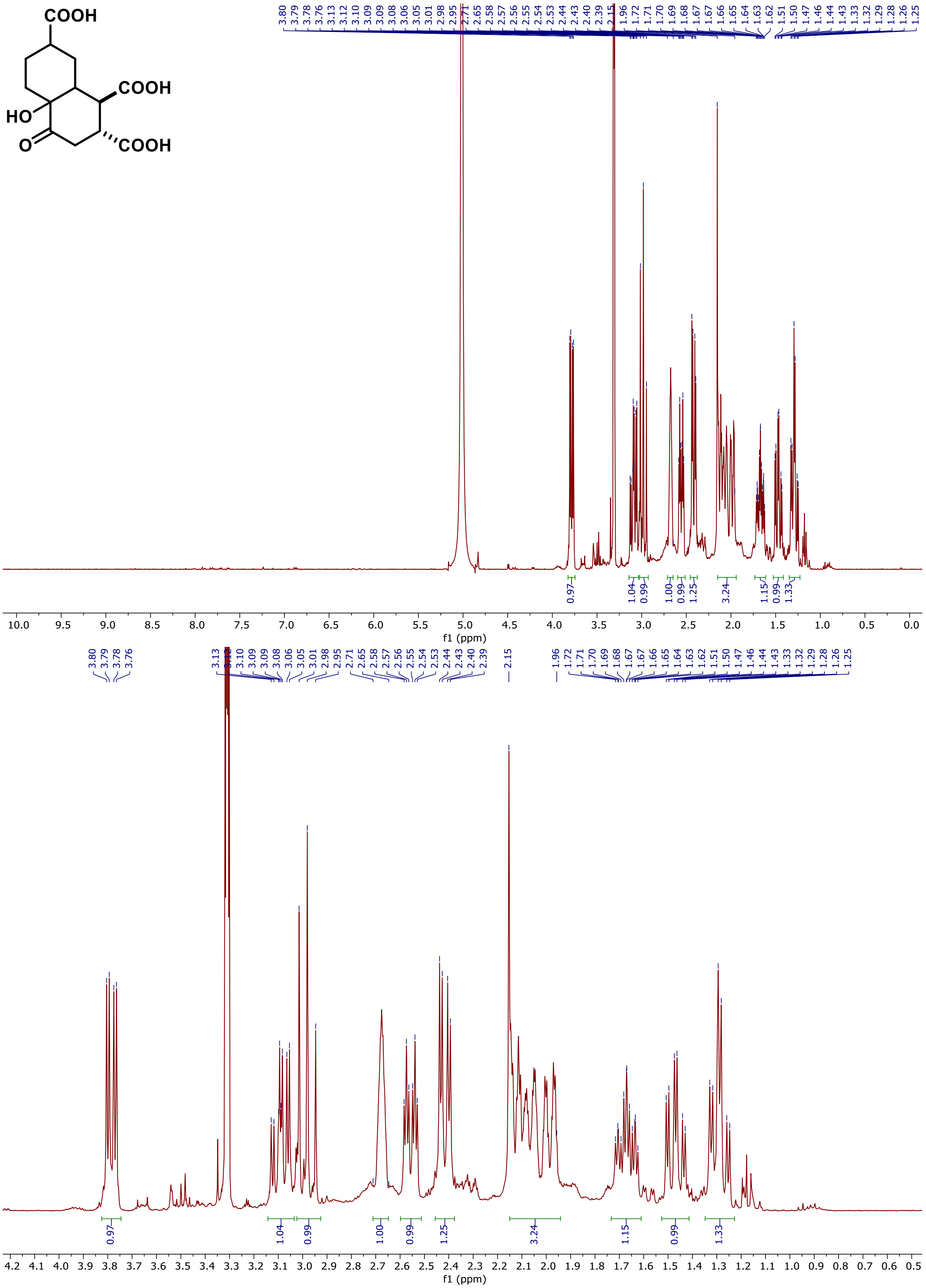

Figure S124: [1]*H NMR spectra of CRAM α-hydroxy ketone* ***12*** *(400 MHz, MeOD).*

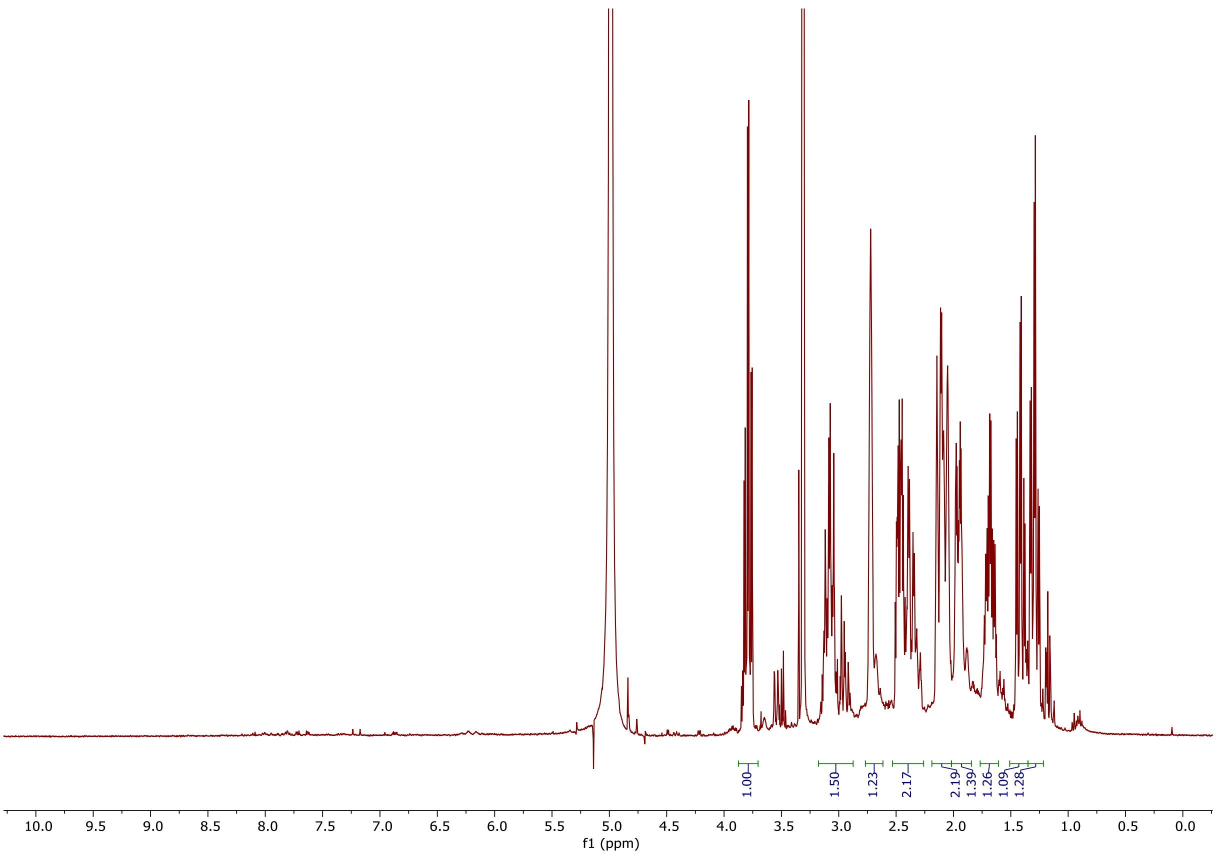

Figure S125: *^{1}H NMR spectra of α-hydroxy ketone* ***12*** *degradation product (400 MHz, MeOD).*

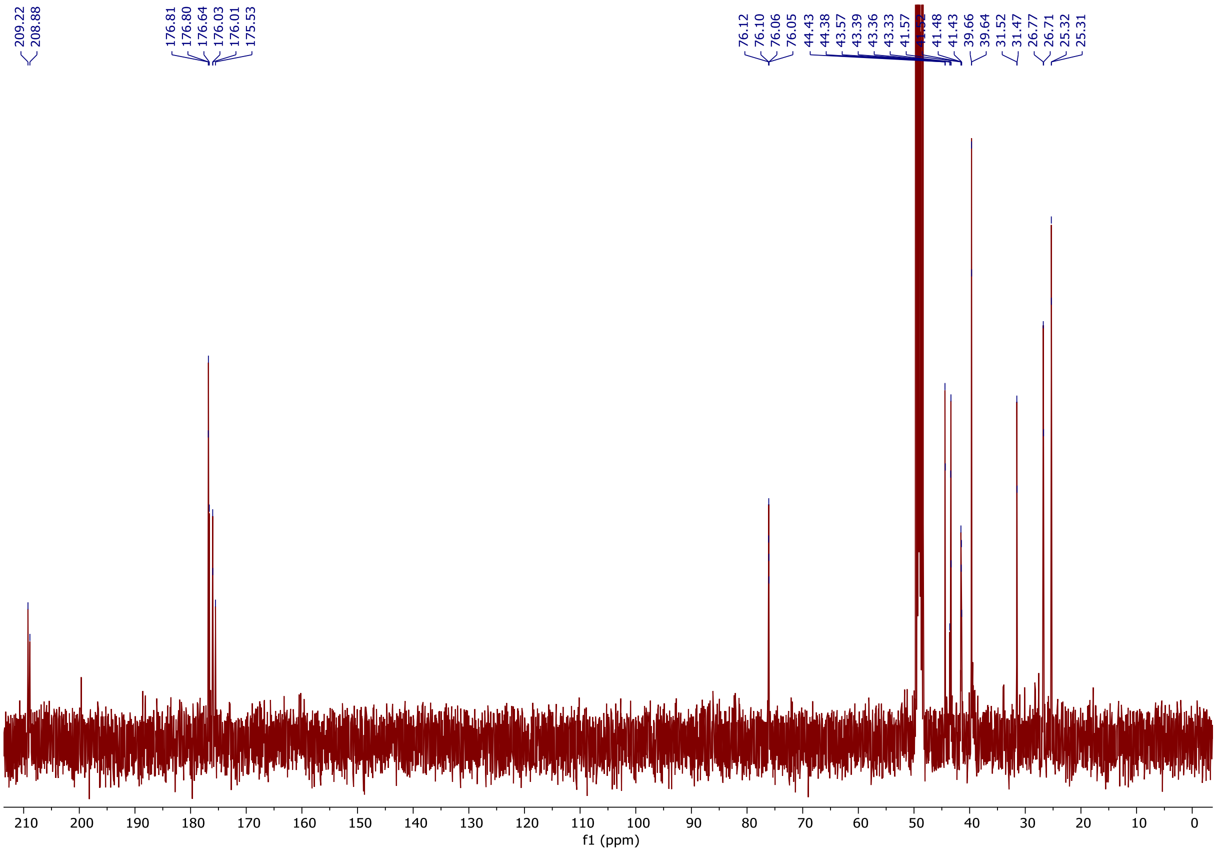

Figure S126: *^{13}C NMR spectra of α-hydroxy ketone* ***12*** *degradation product (101 MHz, MeOD).*

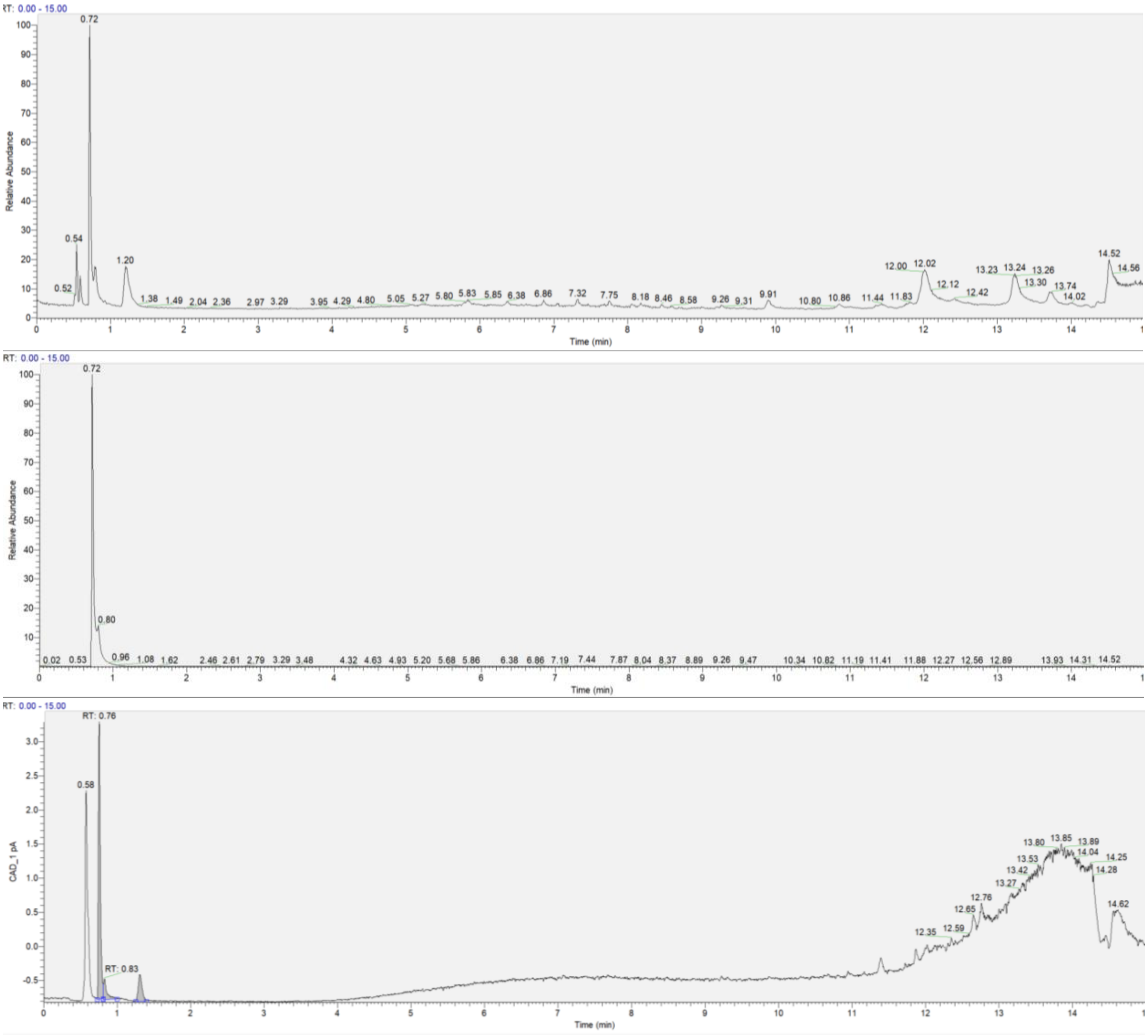

Figure S127: *TIC trace (top), XIC of title compound parent ion (middle), and CAD trace (bottom) of CRAM diol **13** f1.*

Table S10: *LC-MS data and peak identities for CRAM diol **13** f1.*

Apex RT	Start RT	End RT	Area	%Area	*m/z*	Identity
0.76	0.73	0.81	7.593	75.30	301.0935	Title compound **13** isomer
0.83	0.81	1.00	1.039	10.31	301.0938	Title compound **13** isomer
1.31	1.25	1.40	1.451	14.39	318.1281	Unknown

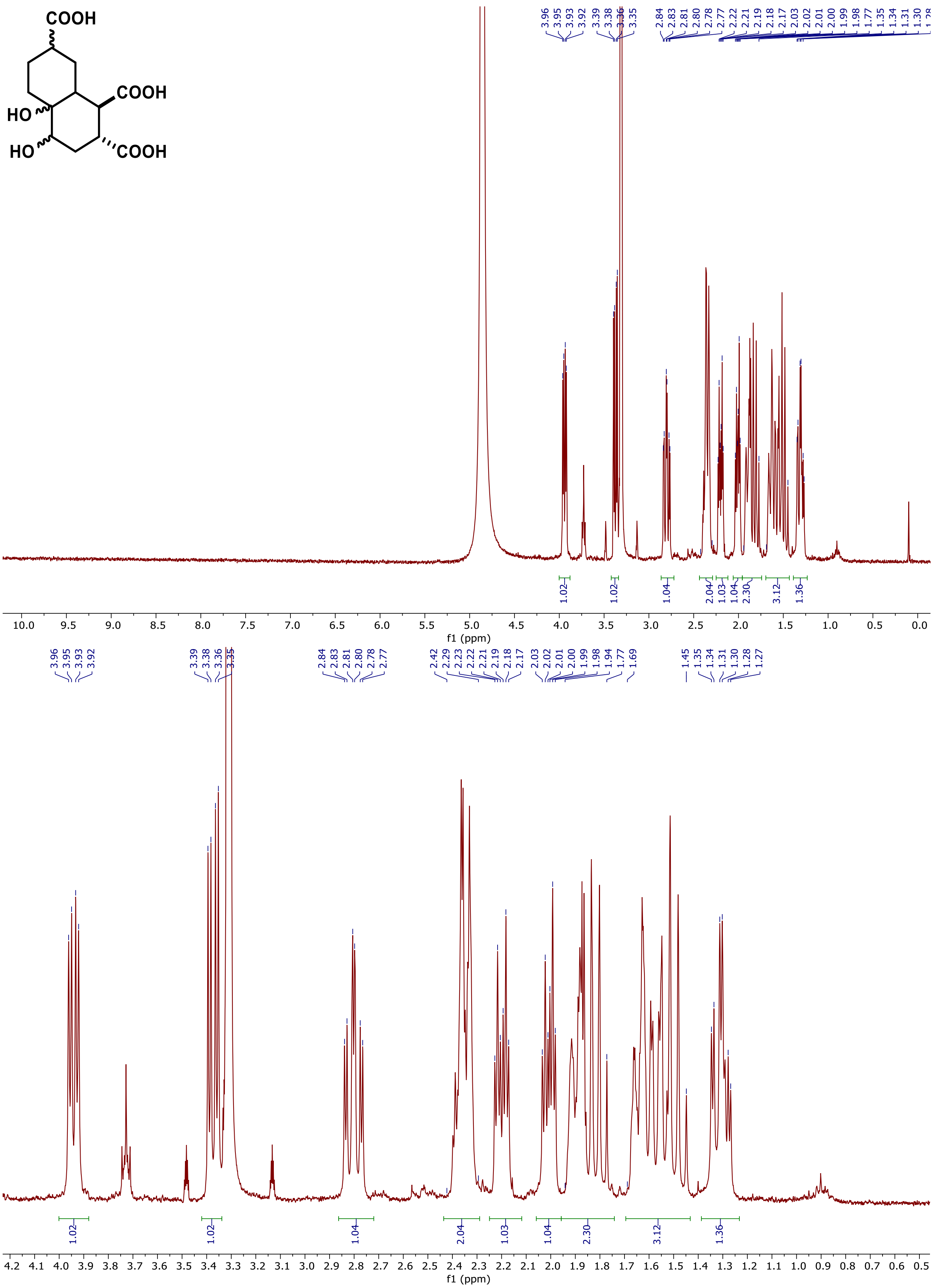

Figure S128: *^{1}H NMR spectra of compound* ***13*** *f1 single diastereomer (400 MHz, MeOD).*

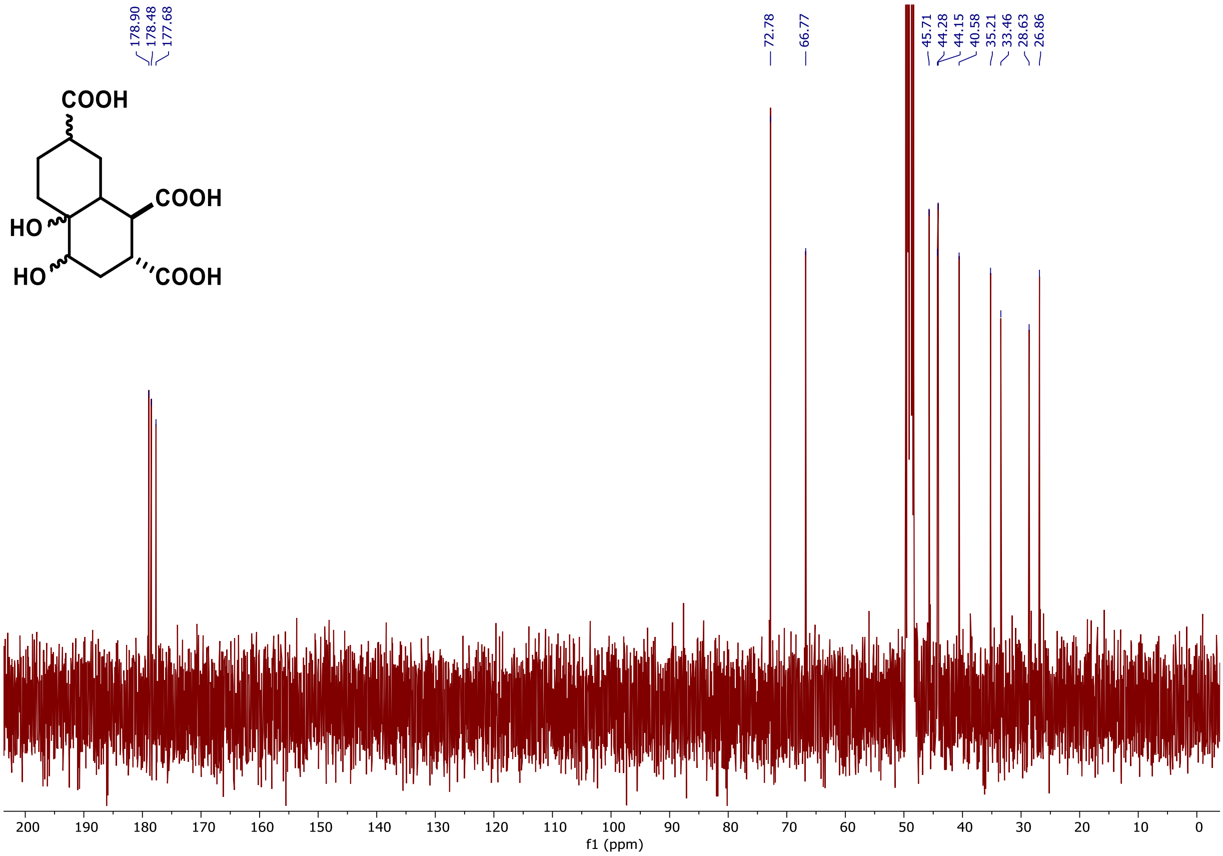
178.90
178.48
177.68
72.78
66.77
45.71
44.28
44.15
40.58
35.21
33.46
28.63
26.86
COOH
COOH
HO
HO
COOH
200 190 180 170 160 150 140 130 120 110 100 90 80 70 60 50 40 30 20 10 0
f1 (ppm)

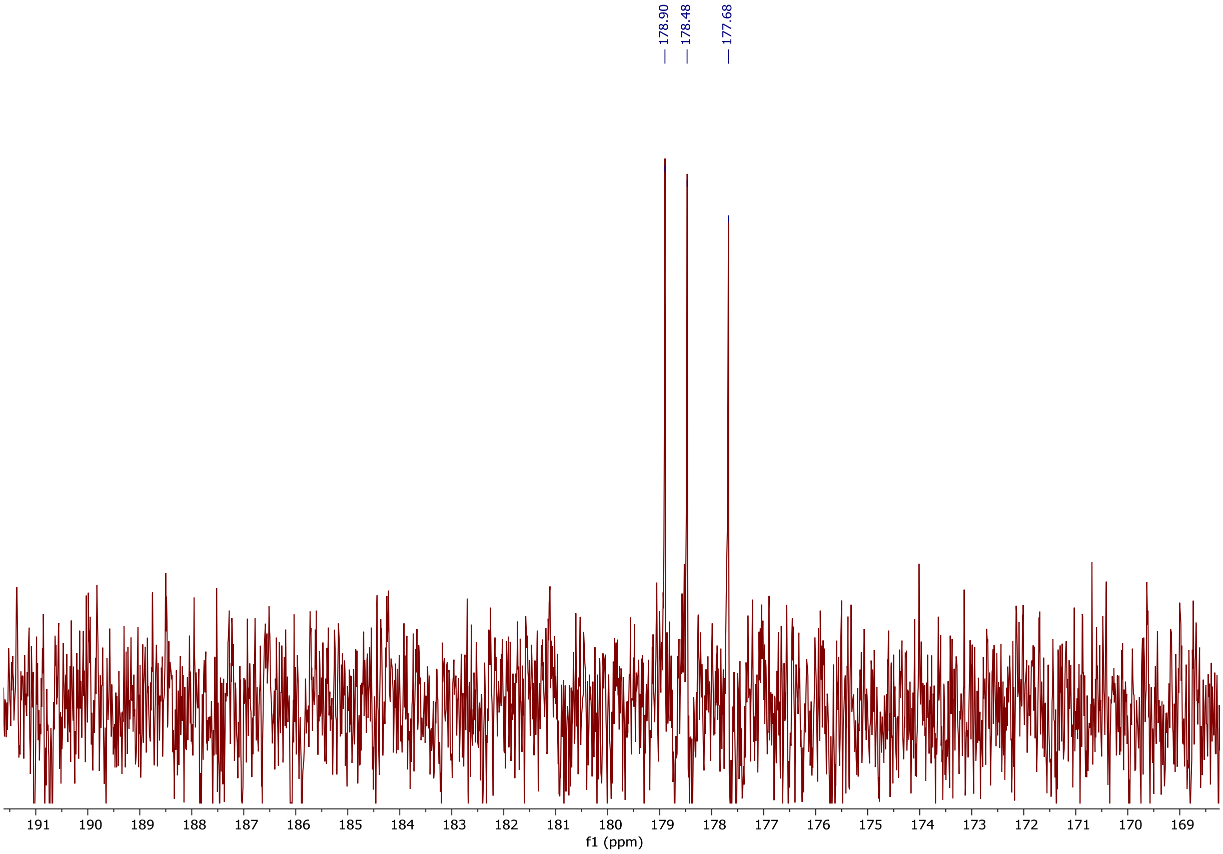
178.90
178.48
177.68
191 190 189 188 187 186 185 184 183 182 181 180 179 178 177 176 175 174 173 172 171 170 169
f1 (ppm)

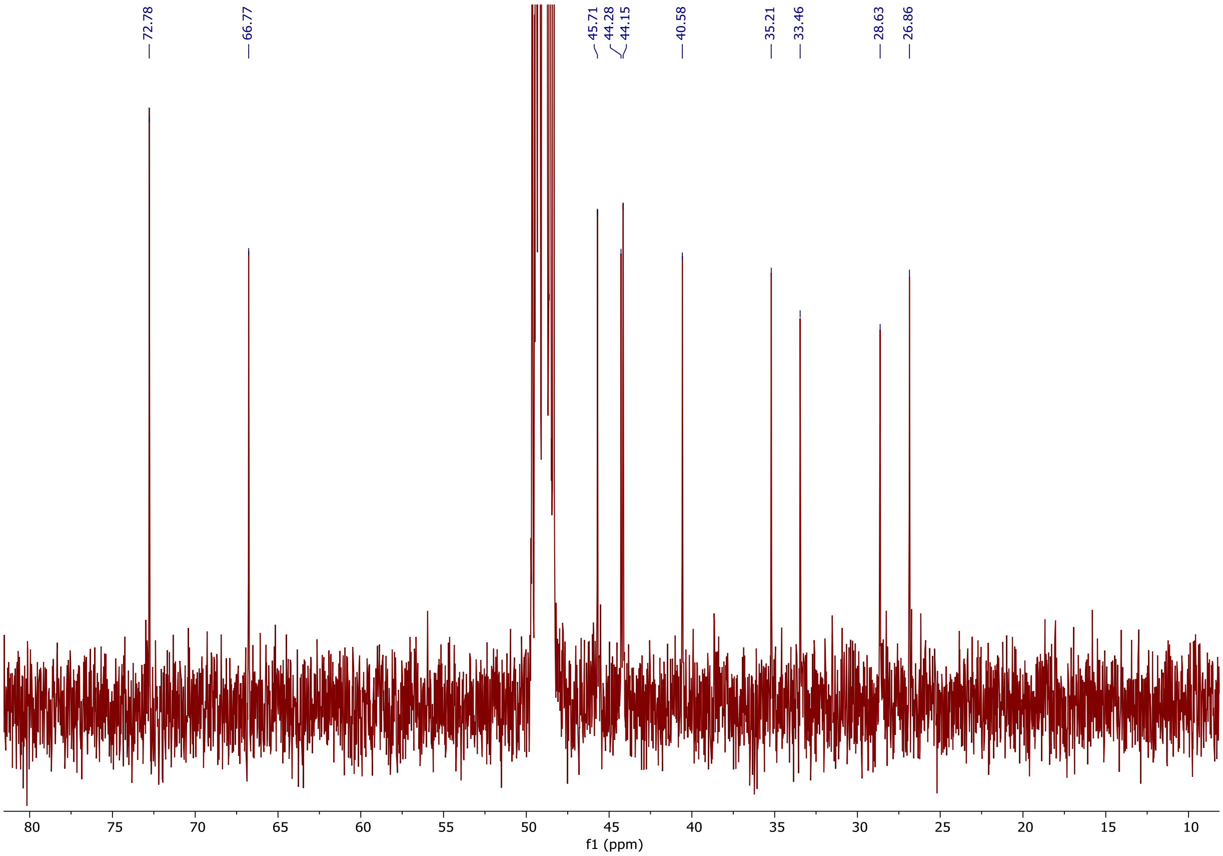

Figure S129: *^{13}C NMR spectra of CRAM diol* ***13*** *f1 (101 MHz, MeOD).*

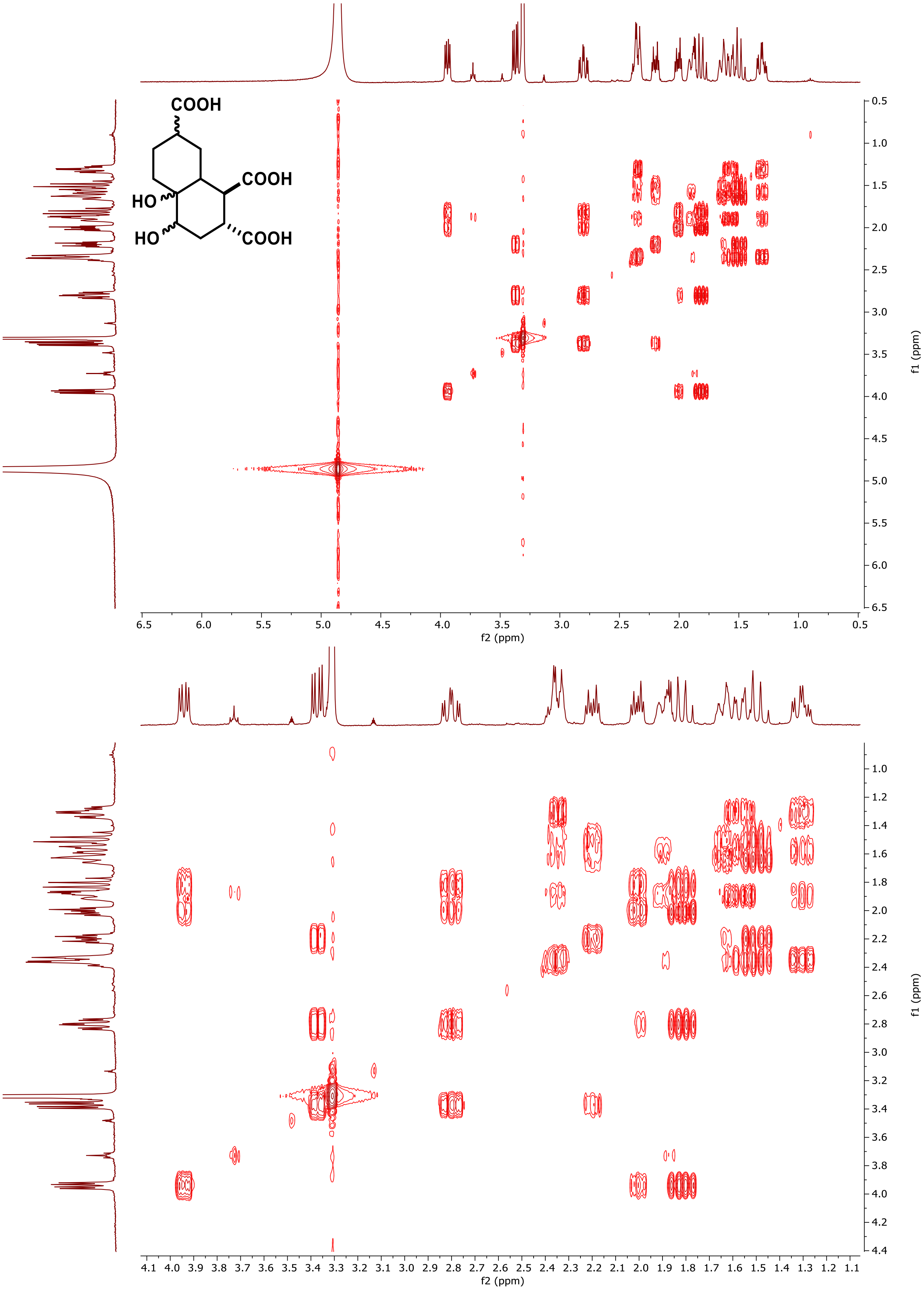

Figure S130: *COSY spectra of CRAM diol* ***13*** *f1.*

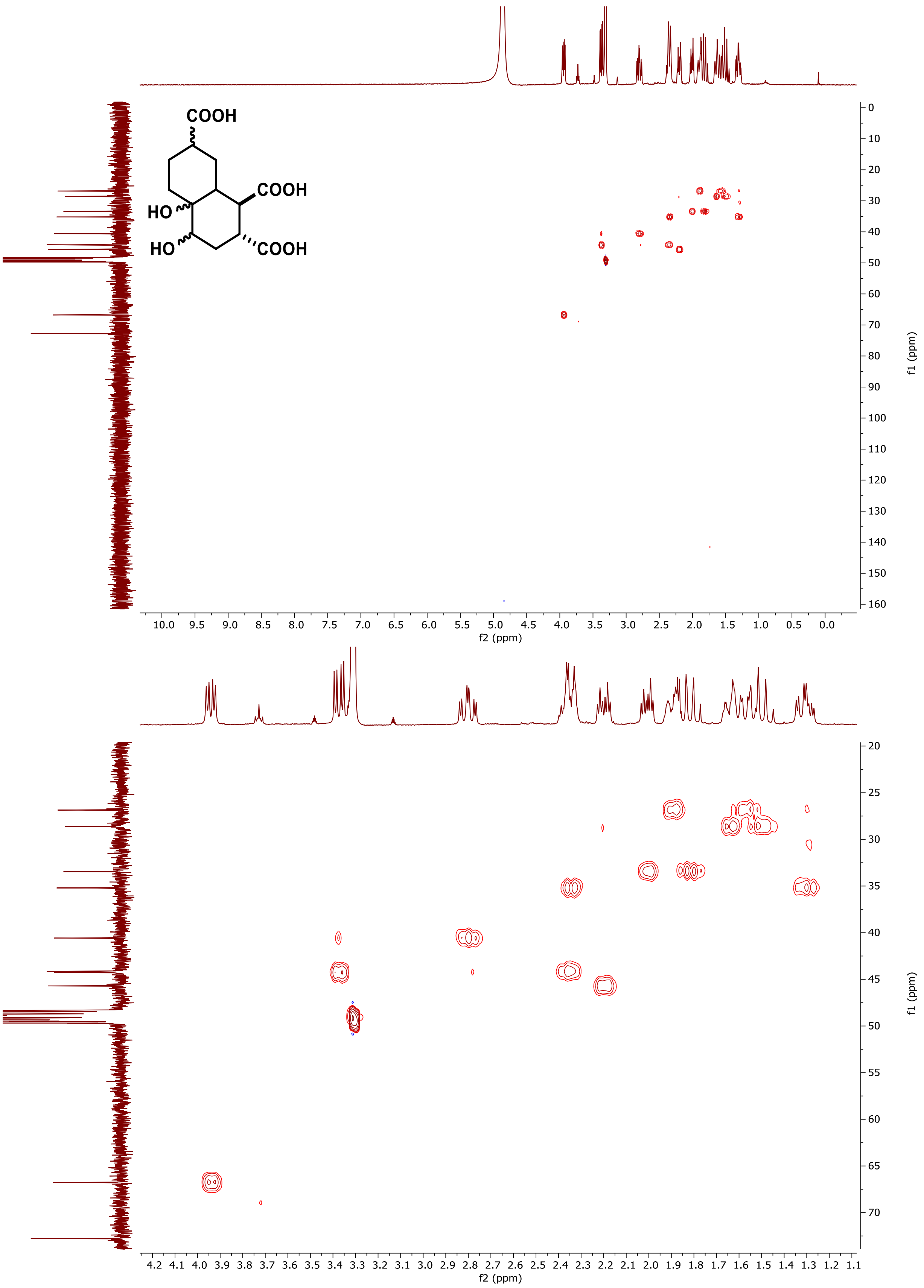

Figure S131: *HSQC spectra of CRAM diol **13** f1.*

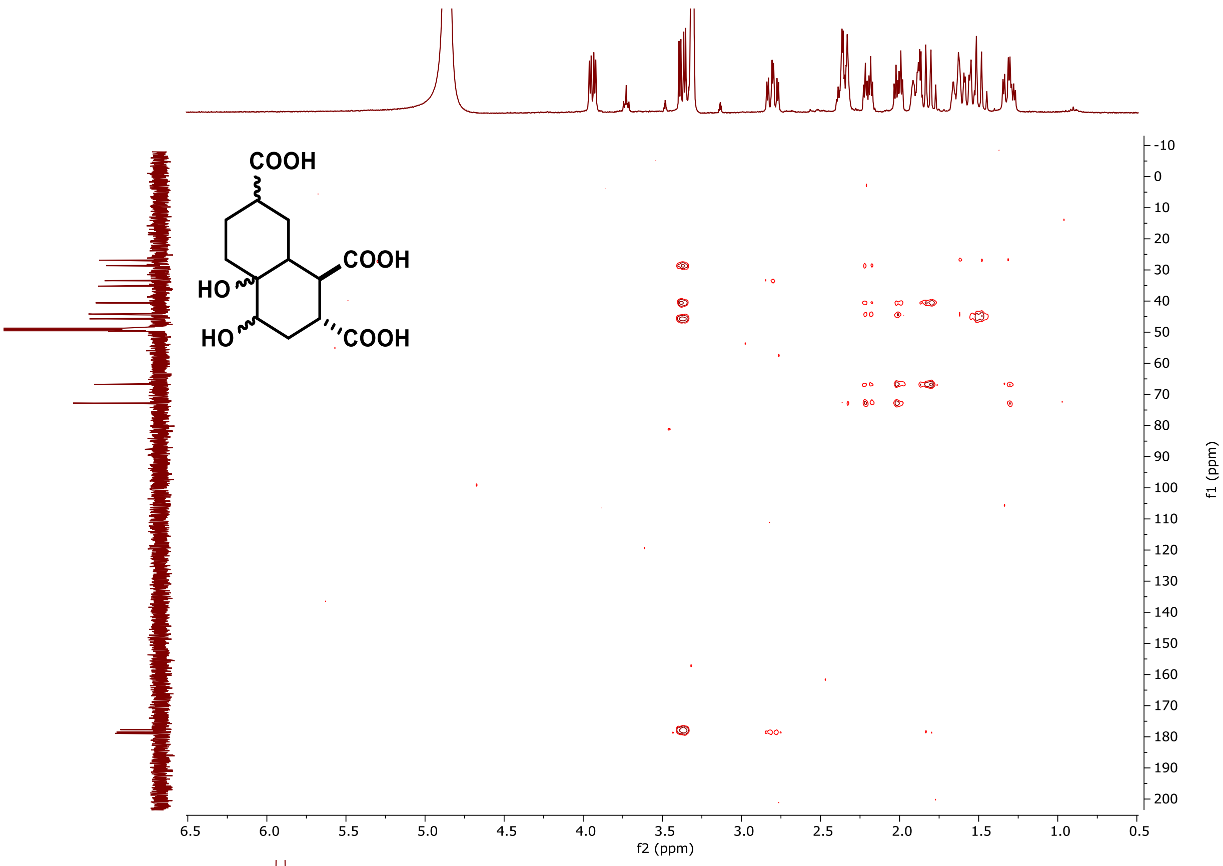
COOH
COOH
HO
HO
COOH
f2 (ppm)
f1 (ppm)

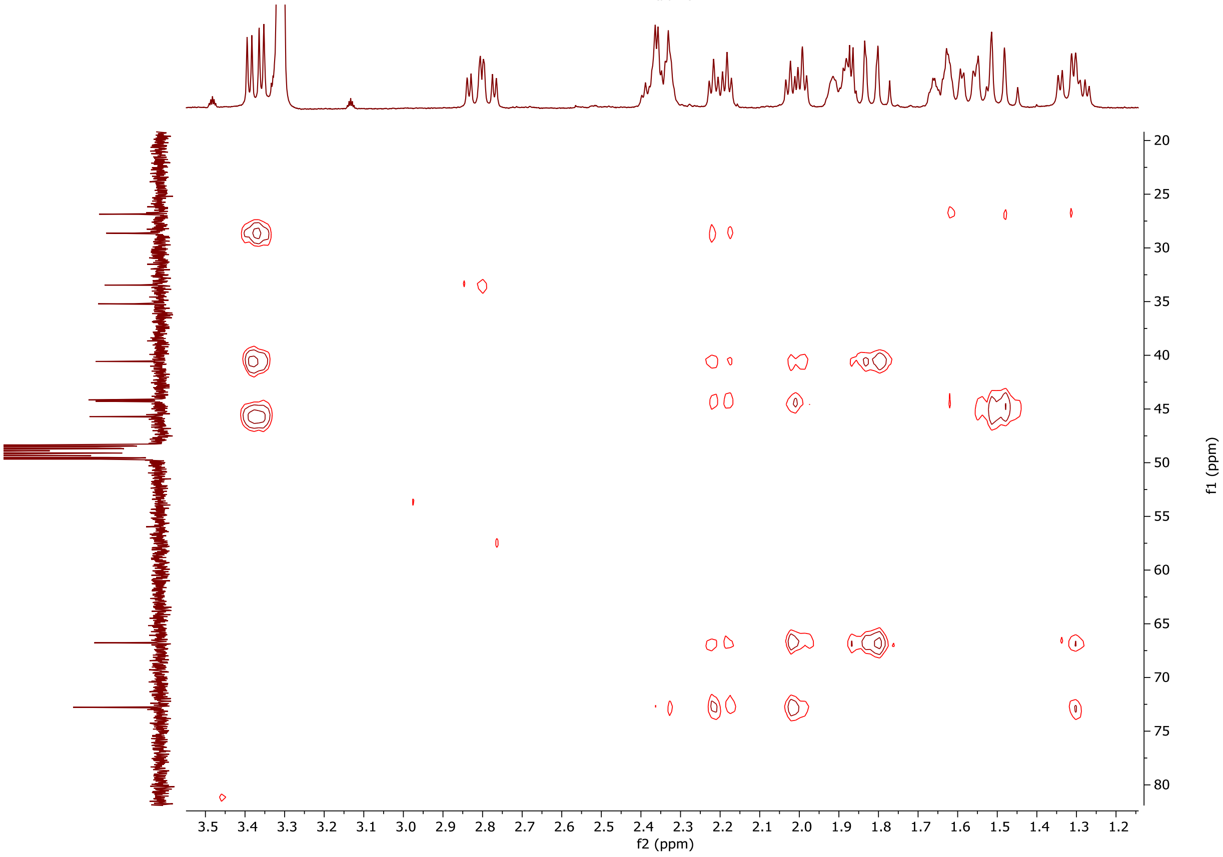
f2 (ppm)
f1 (ppm)

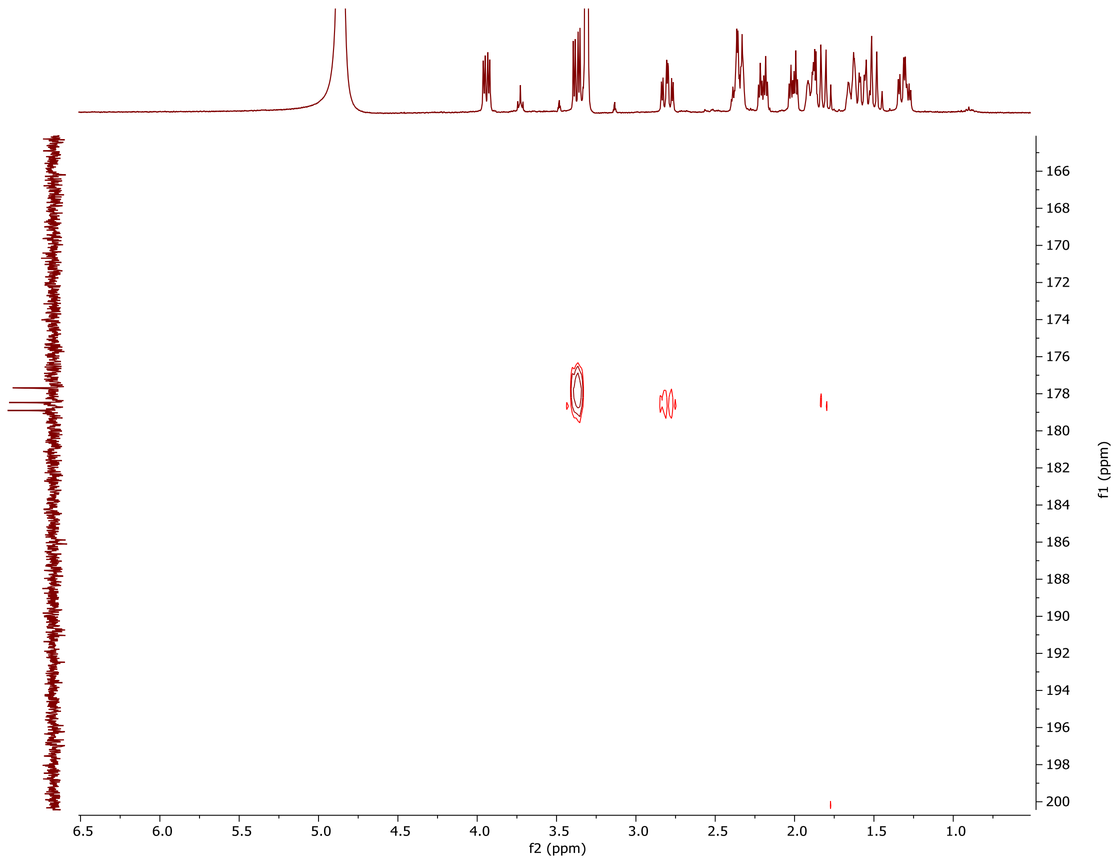

Figure S132: *HMBC spectra of CRAM diol* ***13*** *f1.*

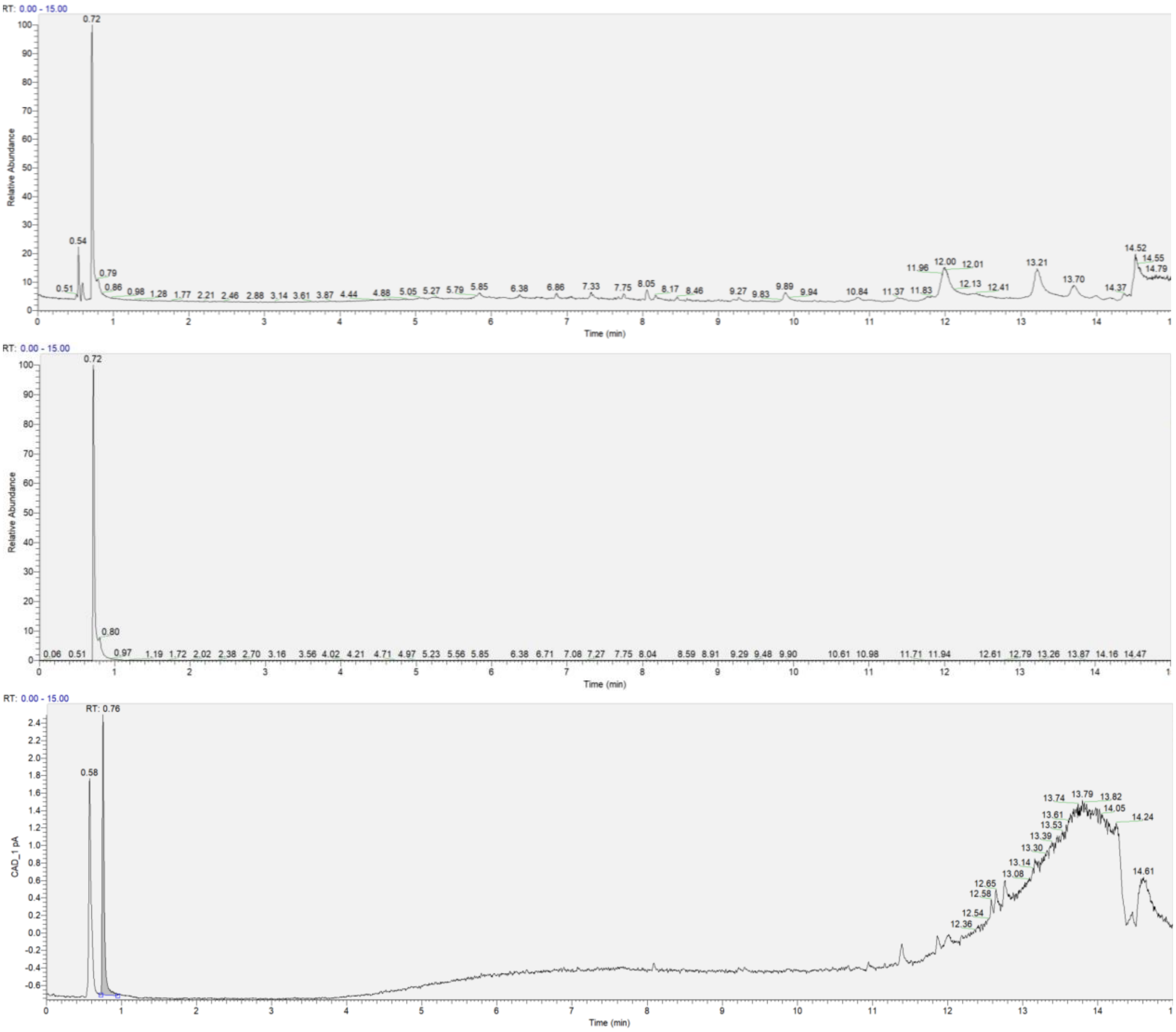

Figure S133: *TIC trace (top), XIC of title compound parent ion (middle), and CAD trace (bottom) of CRAM diol **13** f2.*

Table S11: *LC-MS data and peak identities for CRAM diol **13** f2.*

Apex RT	Start RT	End RT	Area	%Area	*m/z*	Identity
0.76	0.73	0.95	5.901	100.00	301.0937	Title compound **13** isomer

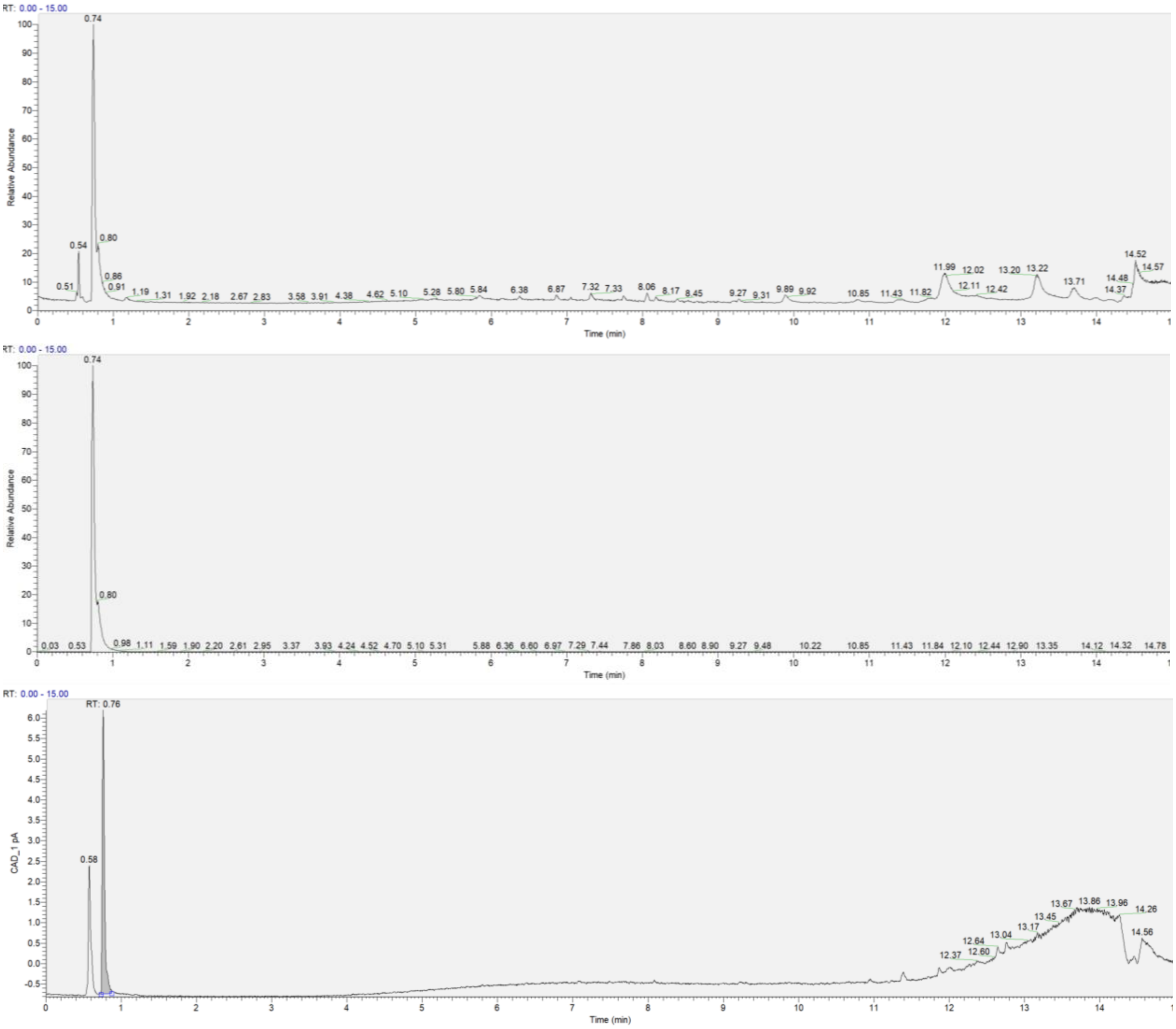

Figure S134: *TIC trace (top), XIC of title compound parent ion (middle), and CAD trace (bottom) of CRAM diol **13** f3.*

Table S12: *LC-MS data and peak identities for CRAM diol **13** f3.*

Apex RT	Start RT	End RT	Area	%Area	*m/z*	Identity
0.76	0.73	0.88	14.746	100.00	301.0935	Title compound **13** isomer

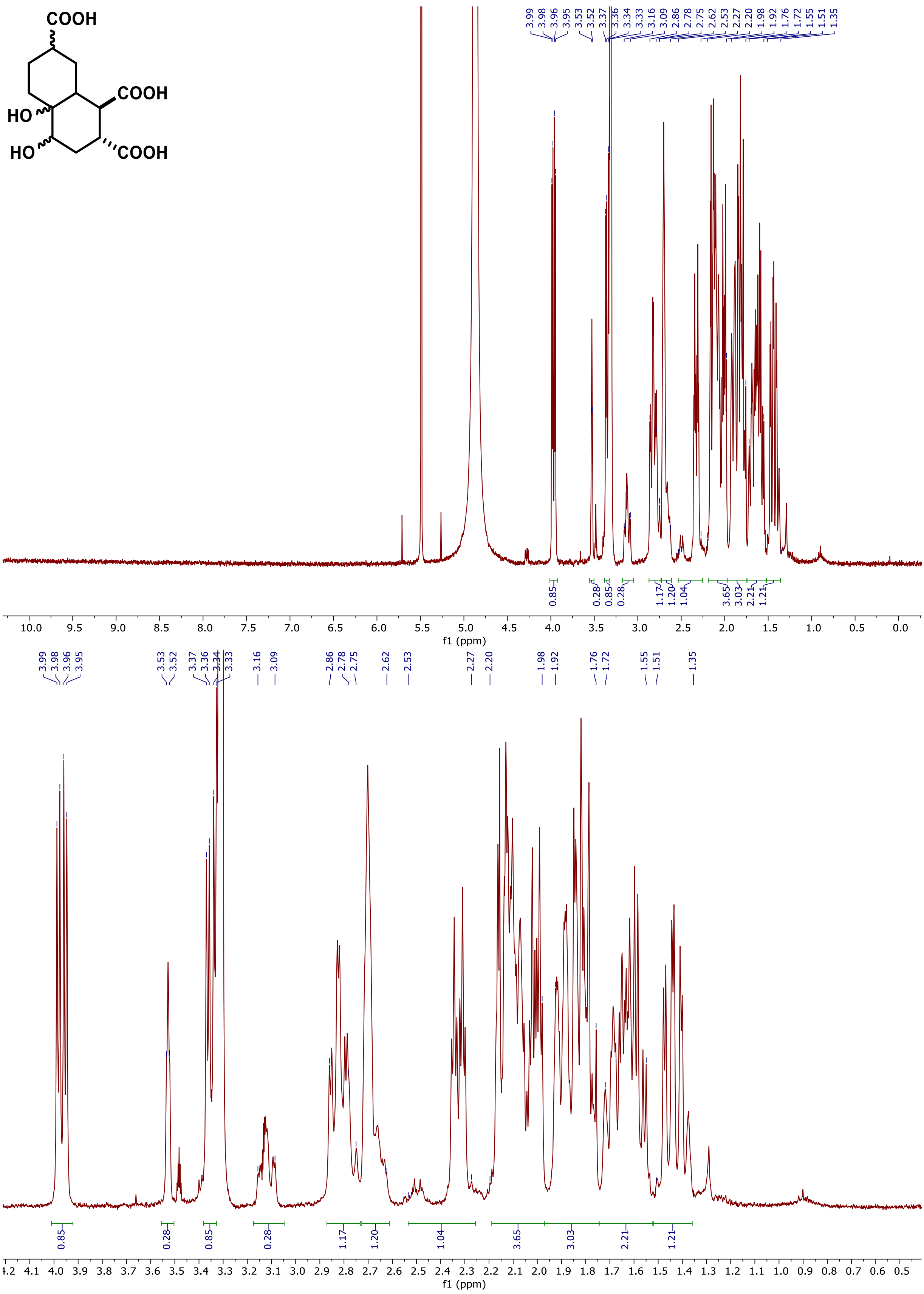

Figure S135: *^{1}H NMR spectra of CRAM diol **13** f3 (400 MHz, MeOD).*

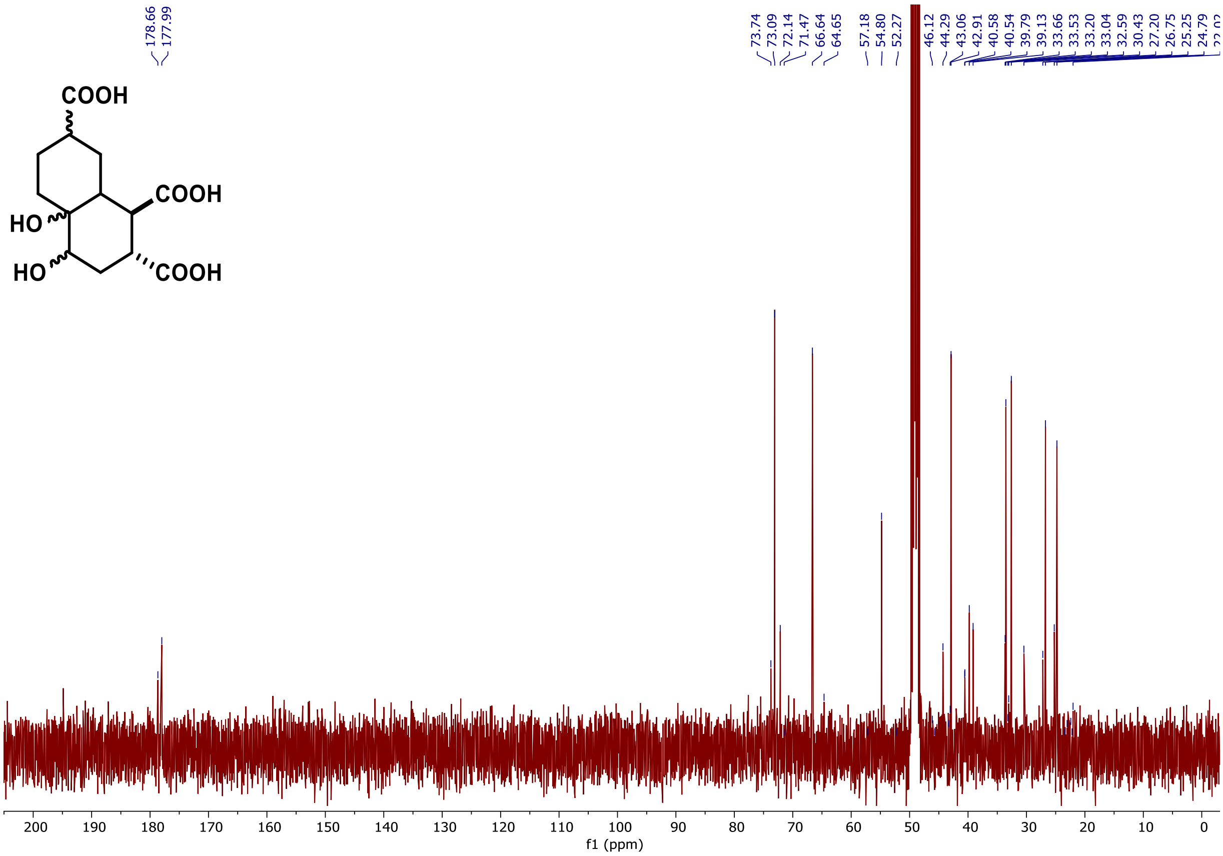
COOH
COOH
HO
HO
COOH
178.66
177.99
73.74
73.09
72.14
71.47
66.64
64.65
57.18
54.80
52.27
46.12
44.29
43.06
42.91
40.58
40.54
39.79
39.13
33.66
33.53
33.20
33.04
32.59
30.43
27.20
26.75
25.25
24.79
f1 (ppm)

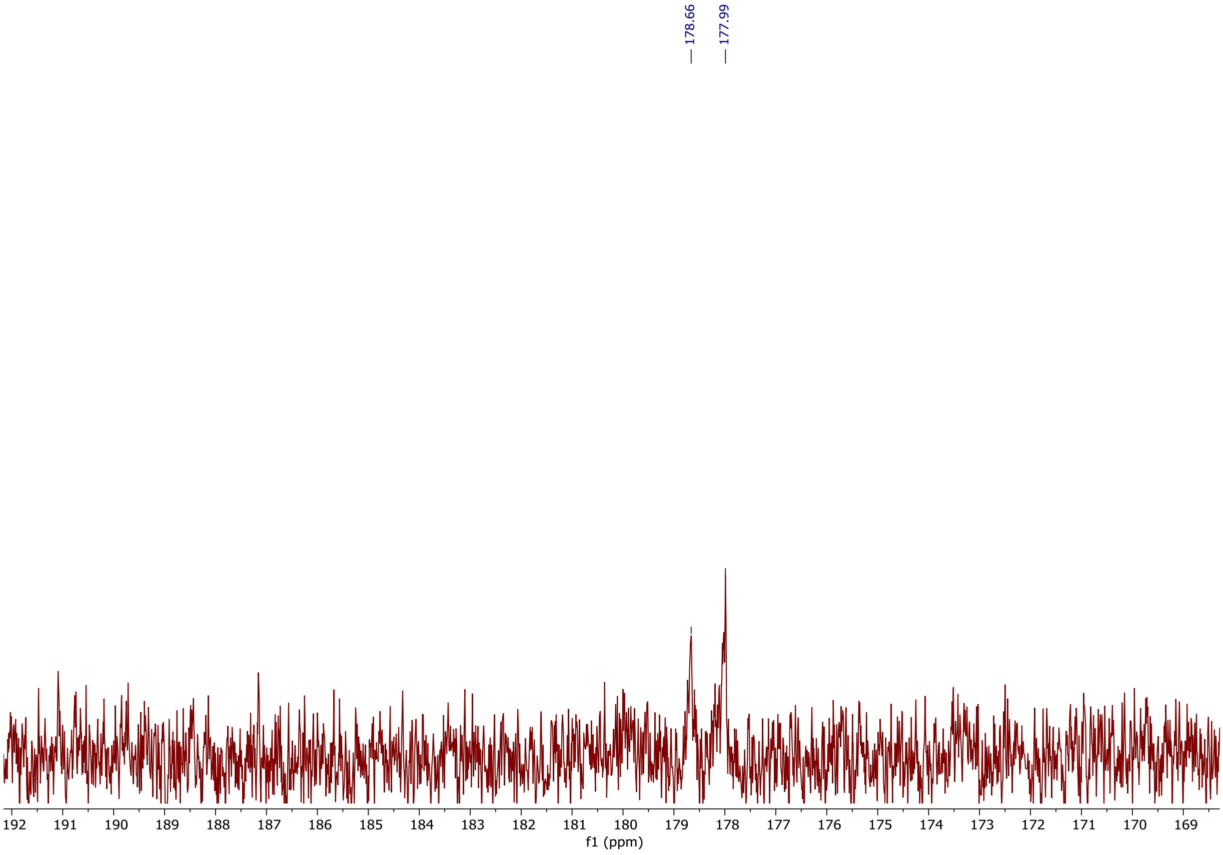
178.66
177.99
f1 (ppm)

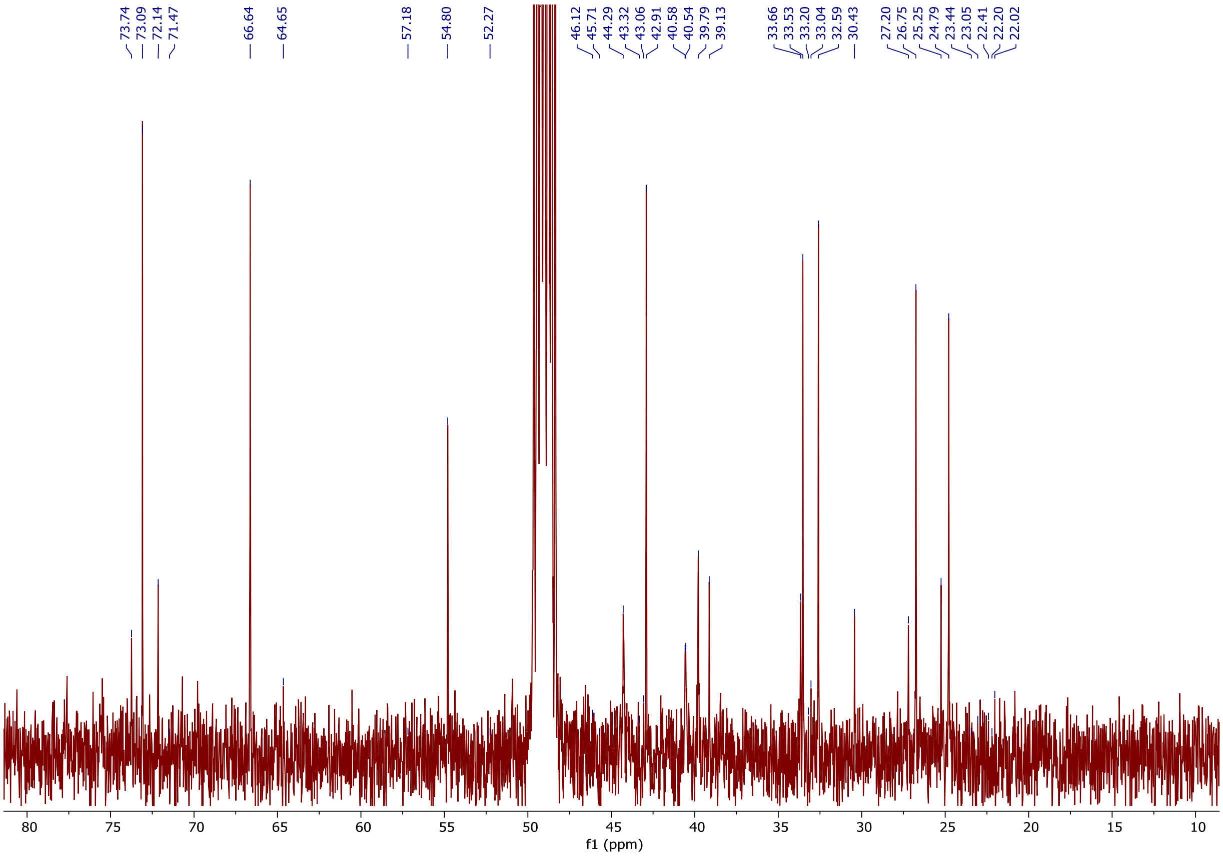

Figure S136: *^{13}C NMR spectra of compound **13** f3 (101 MHz, MeOD).*

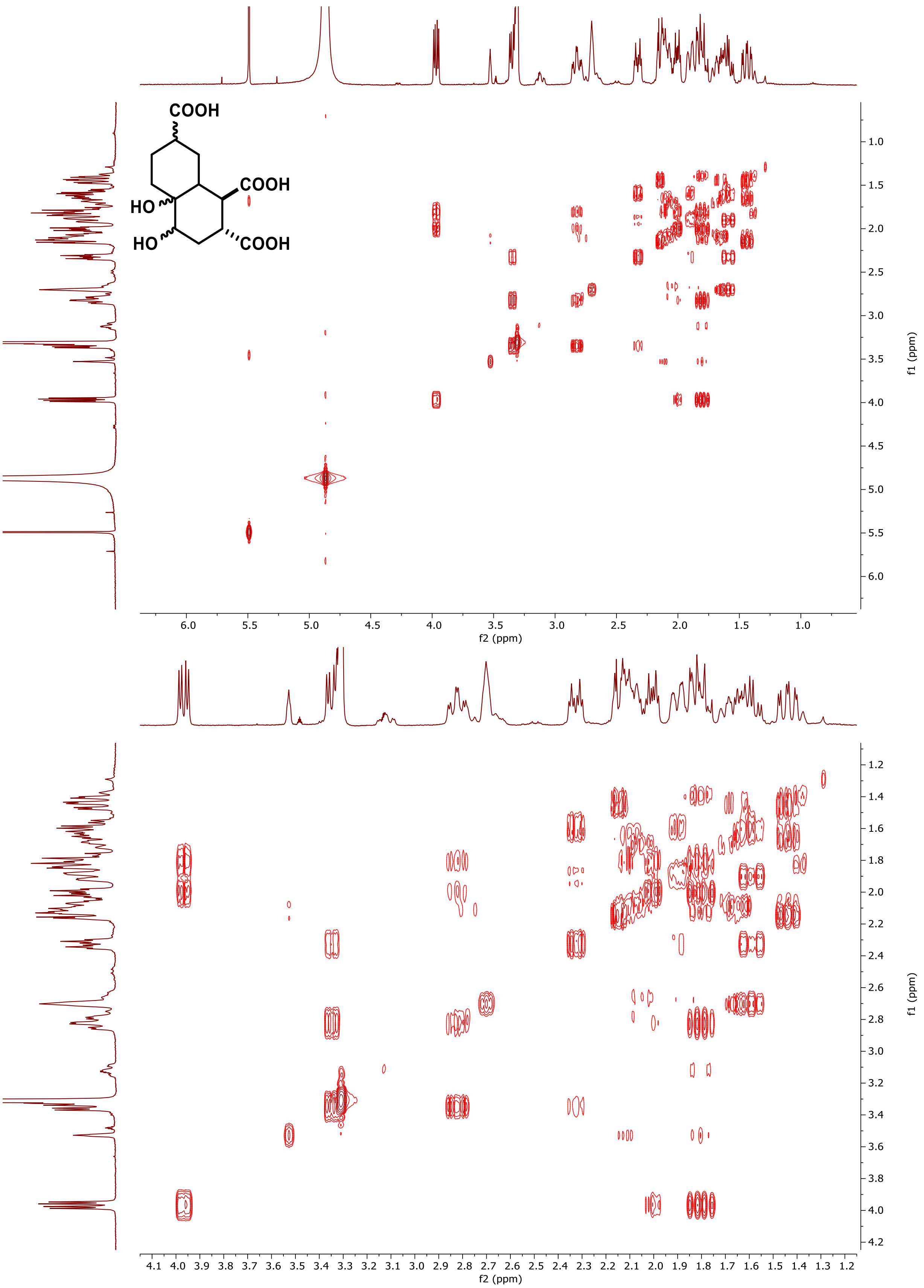

Figure S137: *COSY spectra of CRAM diol* ***13*** *f3.*

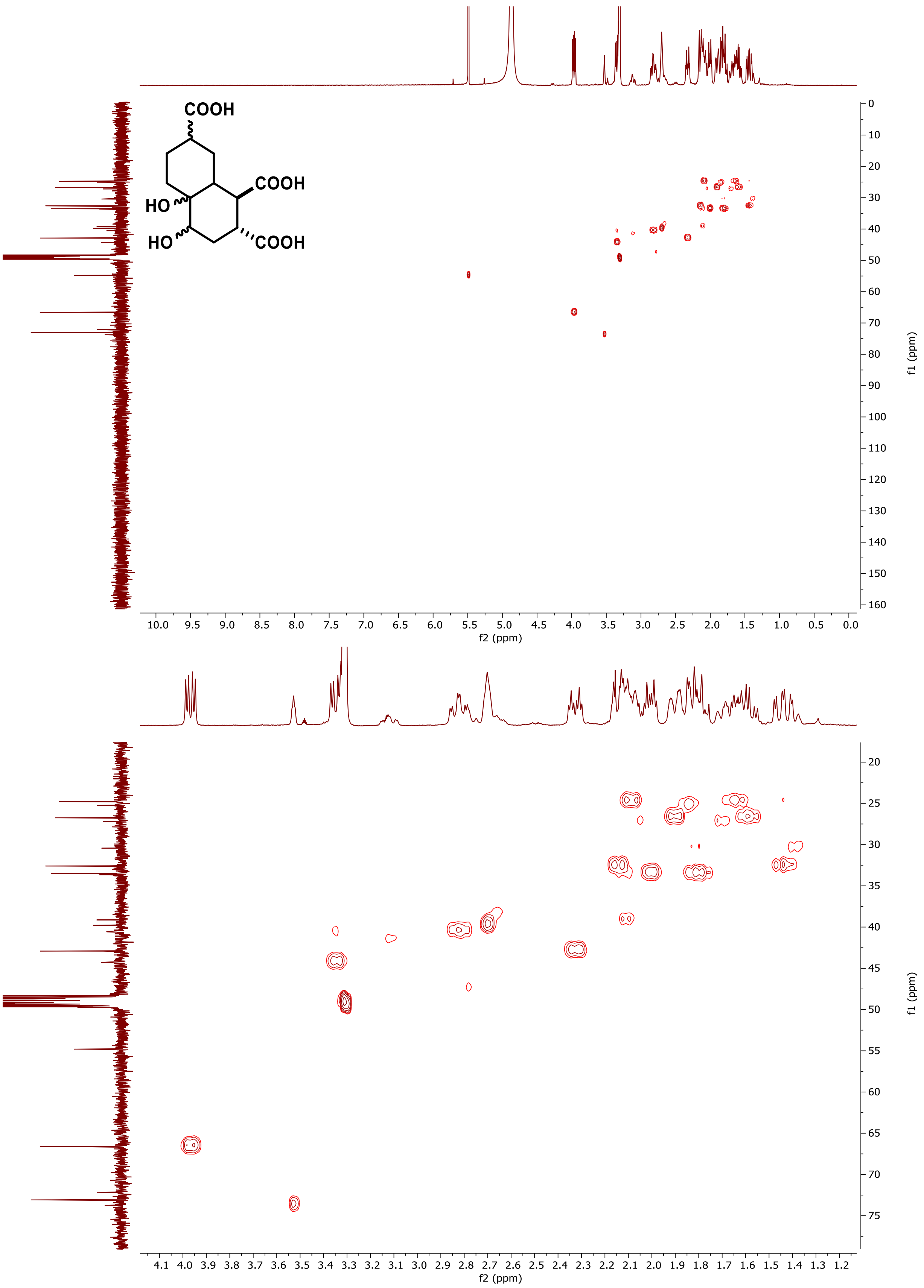

Figure S138: *HSQC spectra of CRAM diol **13** f3.*

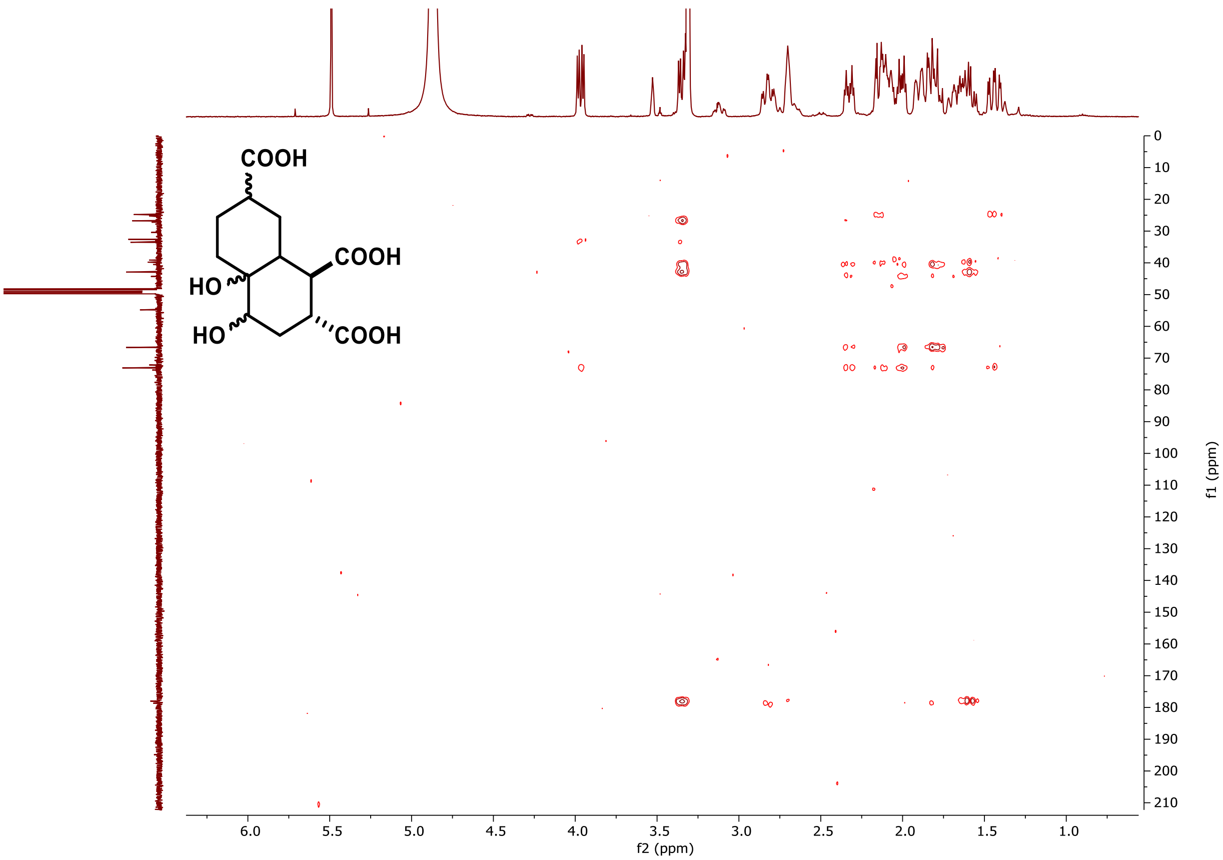
COOH
HO
HO
COOH
COOH
f2 (ppm)
f1 (ppm)

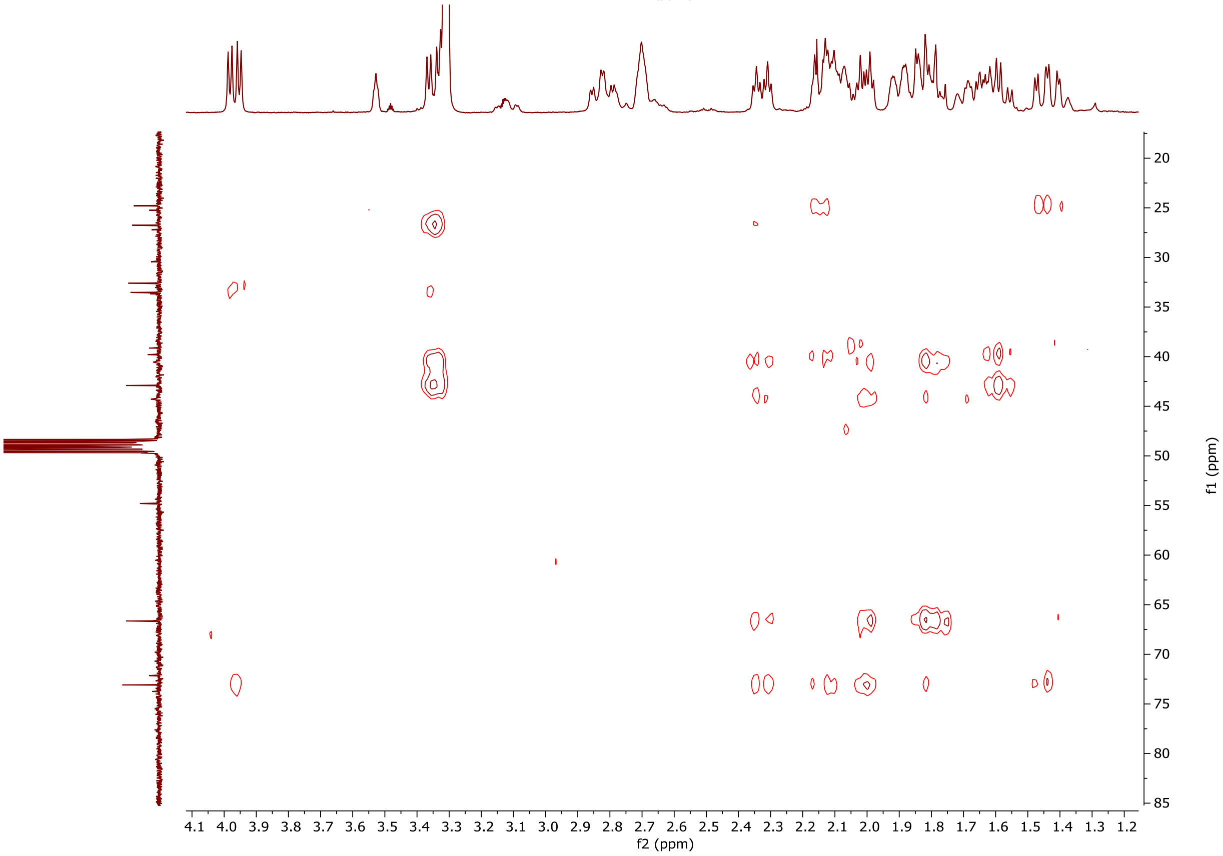
f2 (ppm)
f1 (ppm)

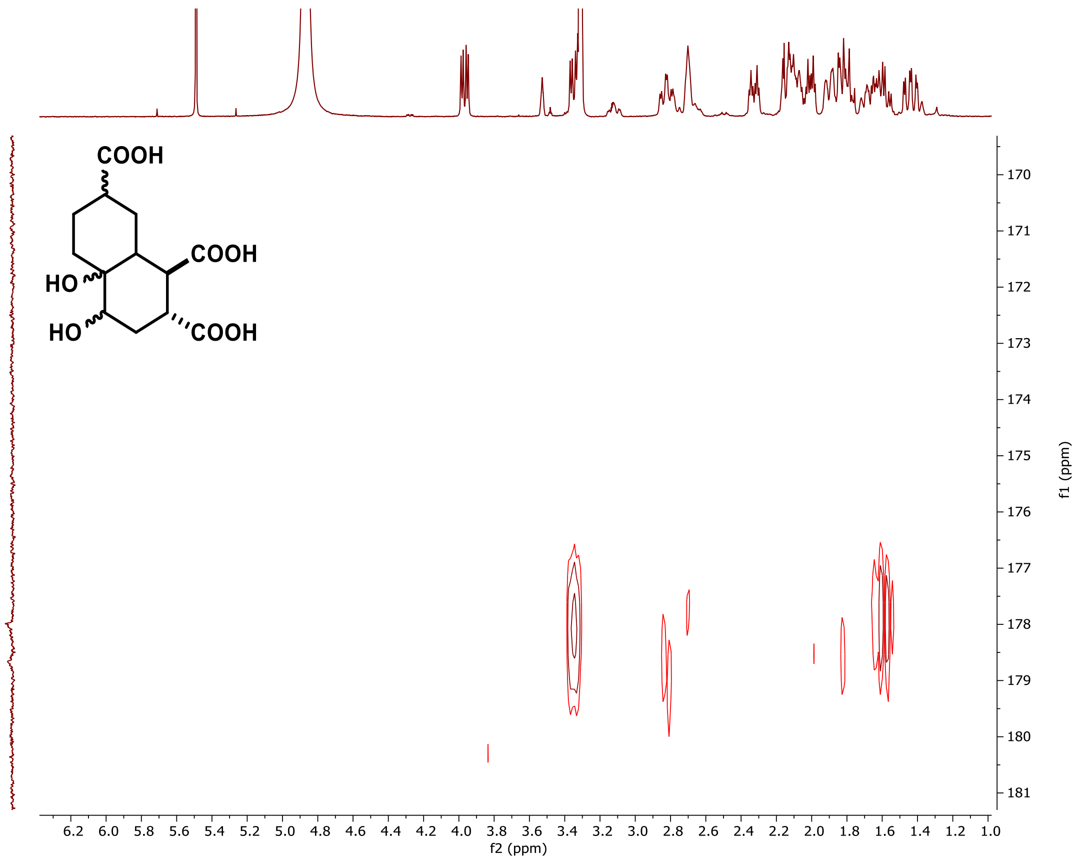

Figure S139: *HMBC spectra of CRAM diol **13** f3.*

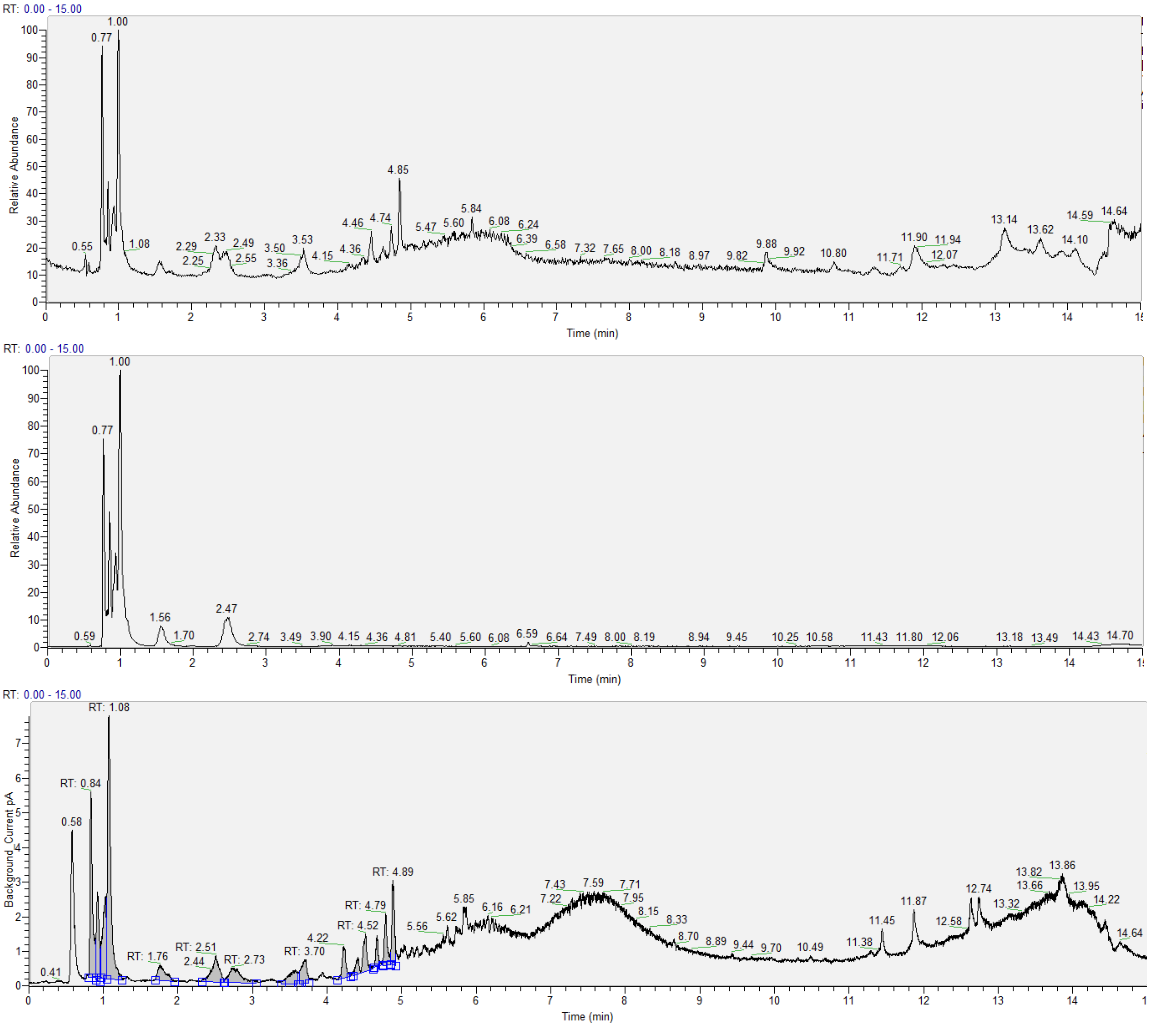

Figure S140: *TIC trace (top), XIC of title compound parent ion (middle), and CAD trace (bottom) of CRAM diacid diol **14**.*

Table S13: *LC-MS data and peak identities for CRAM diacid **14**.*

Apex RT	Start RT	End RT	Area	%Area	*m/z*	Identity
0.84	0.81	0.90	12.659	14.20	257.1029	Title compound **14** isomer
0.92	0.90	0.96	6.196	6.95	257.1030	Title compound **14** isomer
1.03	0.97	1.04	7.632	8.56	257.1029	Title compound **14** isomer
1.08	1.05	1.25	22.115	24.80	257.1029	Title compound **14** isomer
1.76	1.69	1.95	3.229	3.62	257.1029	Title compound **14** isomer
2.51	2.33	2.61	5.230	5.87	257.1029	Title compound **14** isomer
2.73	2.63	3.05	4.897	5.49	257.1029	Title compound **14** isomer
3.58	3.39	3.62	3.285	3.68	274.1434	Unknown
3.70	3.63	3.76	3.620	4.06	293.0802	Unknown
4.22	4.14	4.31	3.308	3.71	239.0925	Unknown
4.52	4.34	4.61	5.276	5.92	274.1377	Unknown
4.68	4.63	4.74	2.045	2.29	-	Unknown
4.79	4.75	4.85	3.990	4.47	274.1377	Unknown
4.89	4.86	4.92	5.681	6.37	274.1377	Unknown

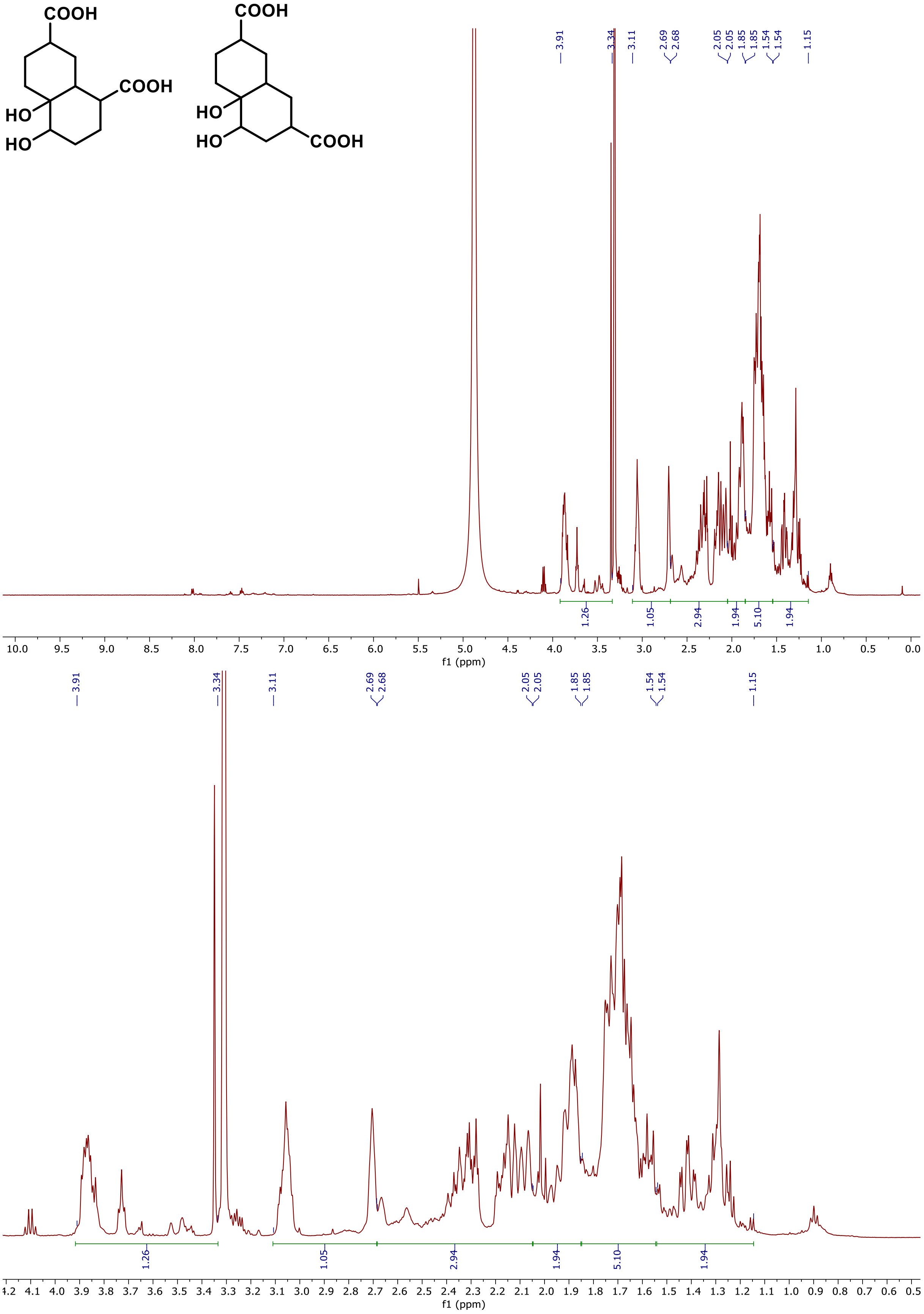

Figure S141: *^{1}H NMR spectra of CRAM diacid diol* ***14*** *(500 MHz, MeOD).*

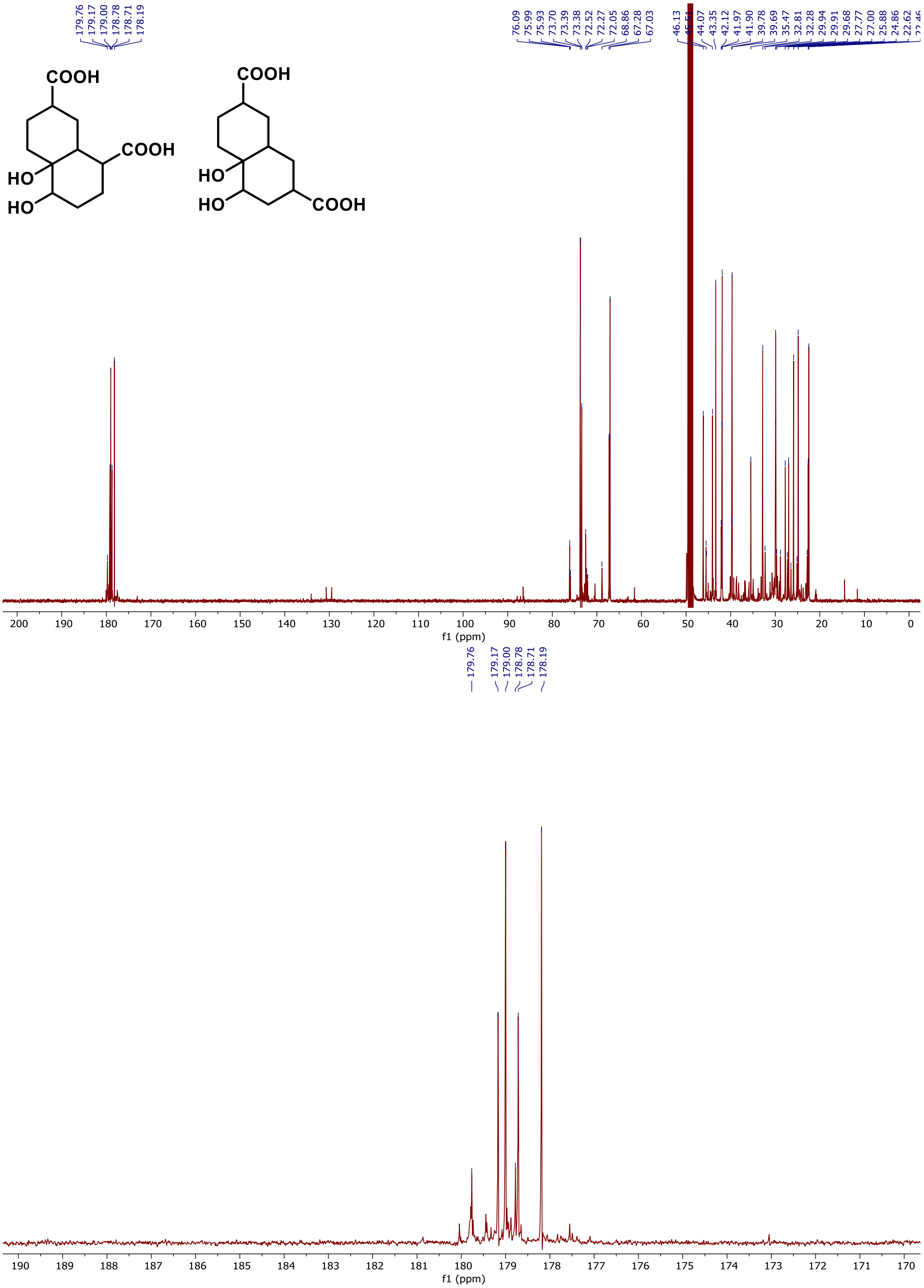
179.76
179.17
179.00
178.78
178.71
178.19
COOH
COOH
HO
HO
COOH
COOH
HO
HO
76.09
75.99
75.93
73.70
73.39
73.38
72.52
72.27
72.05
68.86
67.28
67.03
46.13
44.07
43.35
42.12
41.97
41.90
39.78
39.69
35.47
32.81
32.28
29.94
29.91
29.68
27.77
27.00
25.88
24.86
22.62
f1 (ppm)
179.76
179.17
179.00
178.78
178.71
178.19
f1 (ppm)

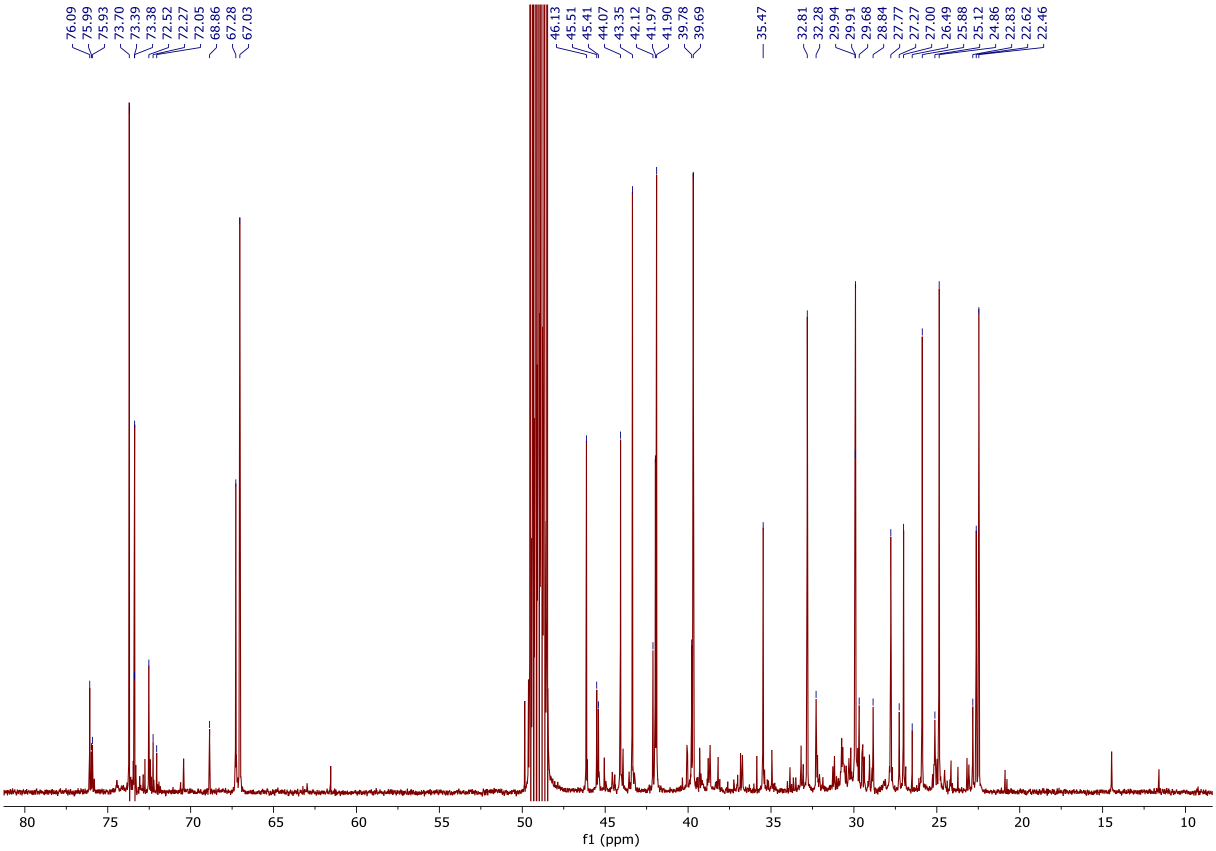

Figure S142: *^{13}C NMR spectra of CRAM diacid diol **14** (126 MHz, MeOD).*

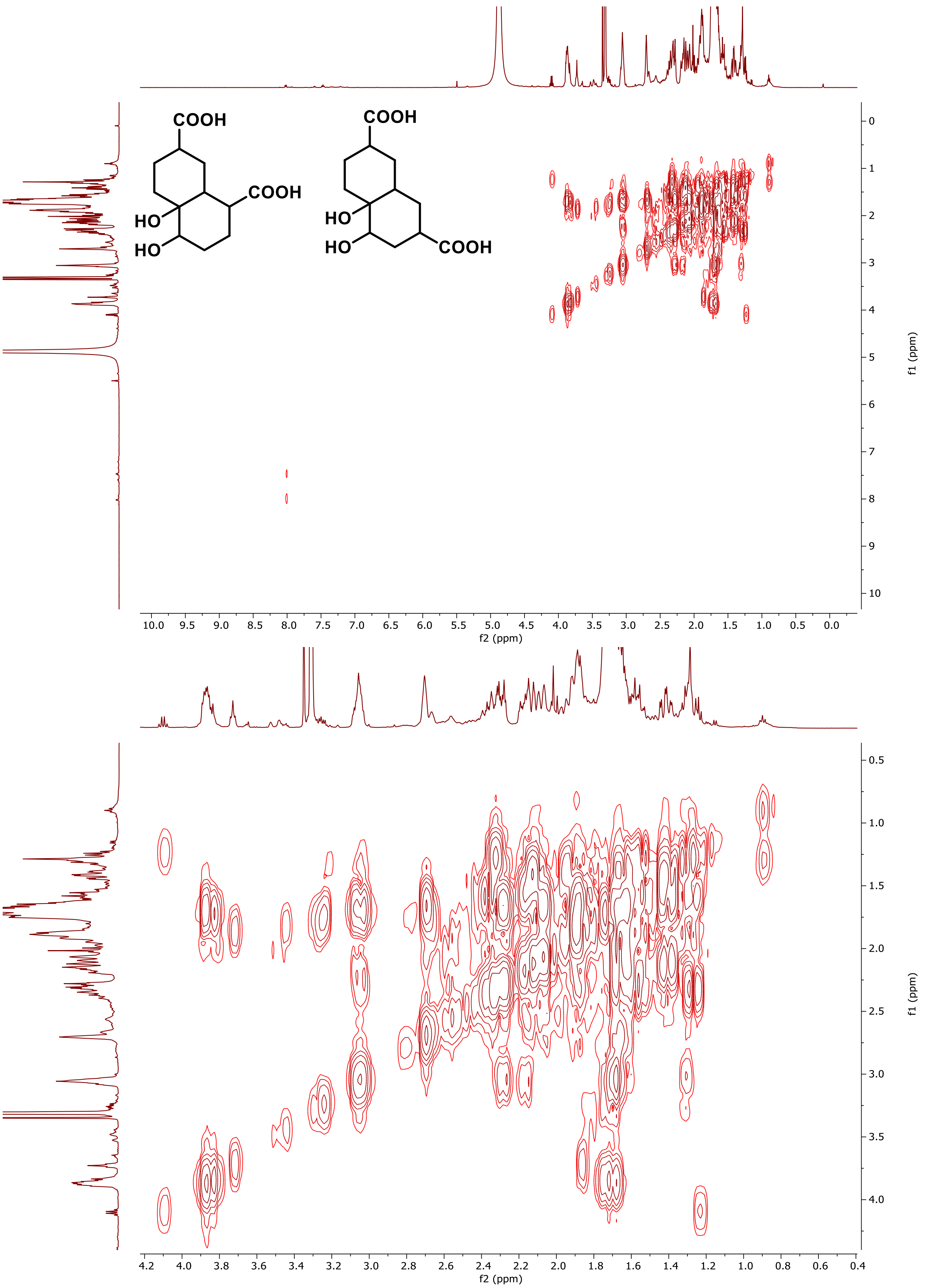

Figure S143: *COSY spectra of CRAM diacid diol **14**.*

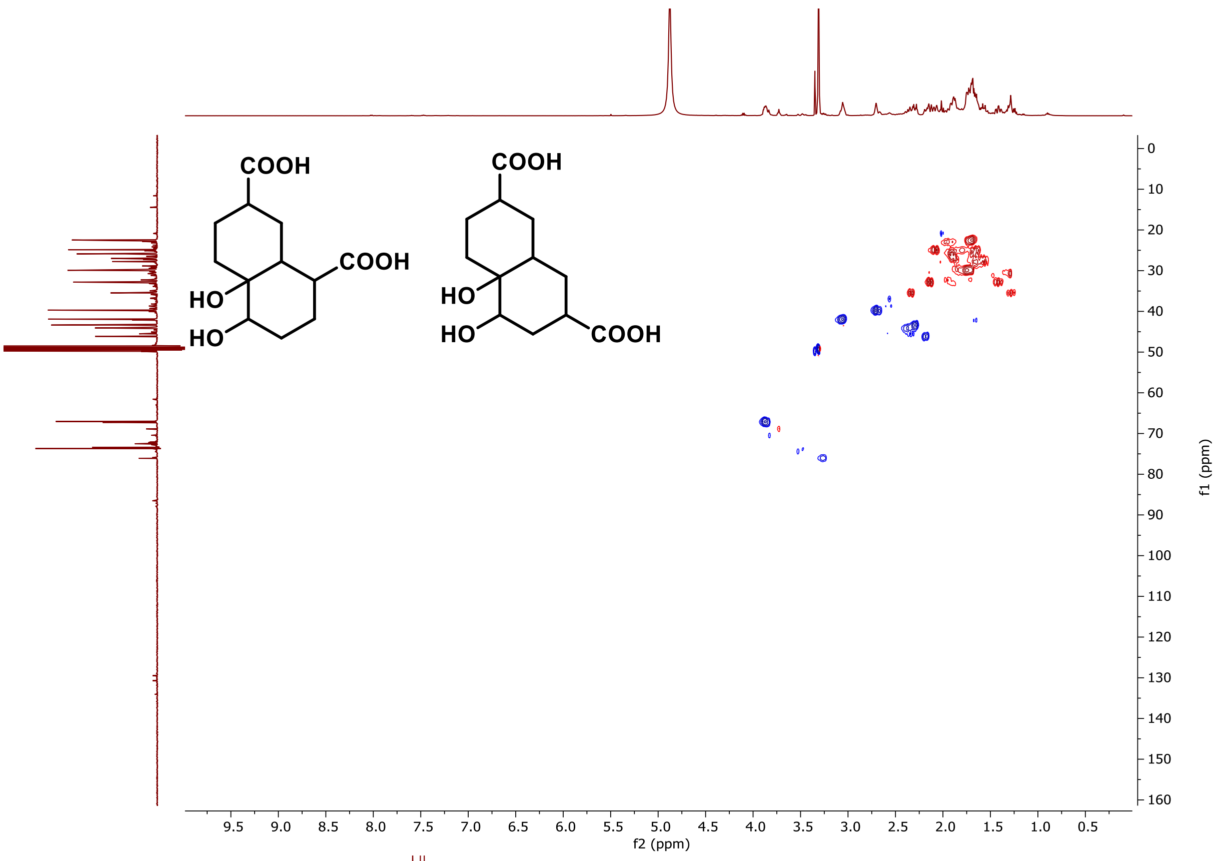

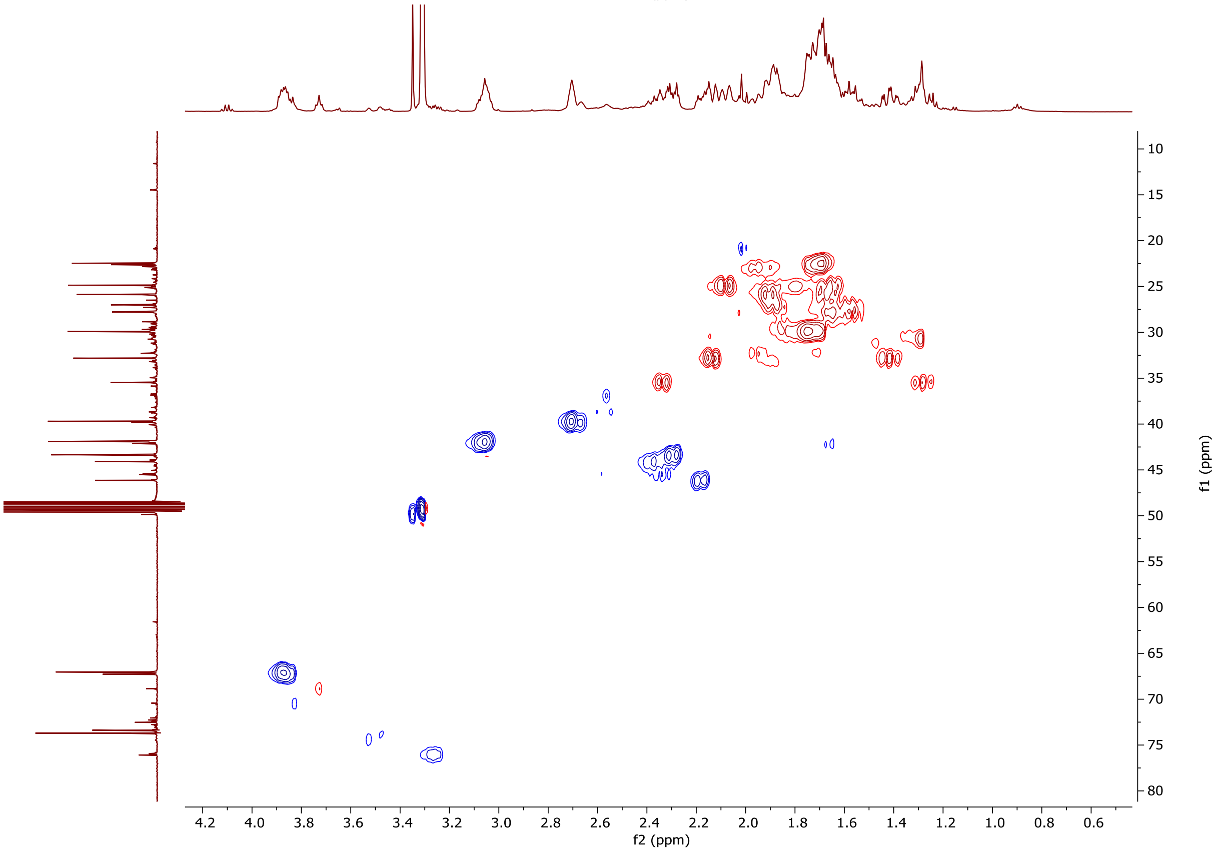

Figure S144: *HSQC spectra of CRAM diacid diol* ***14****.*

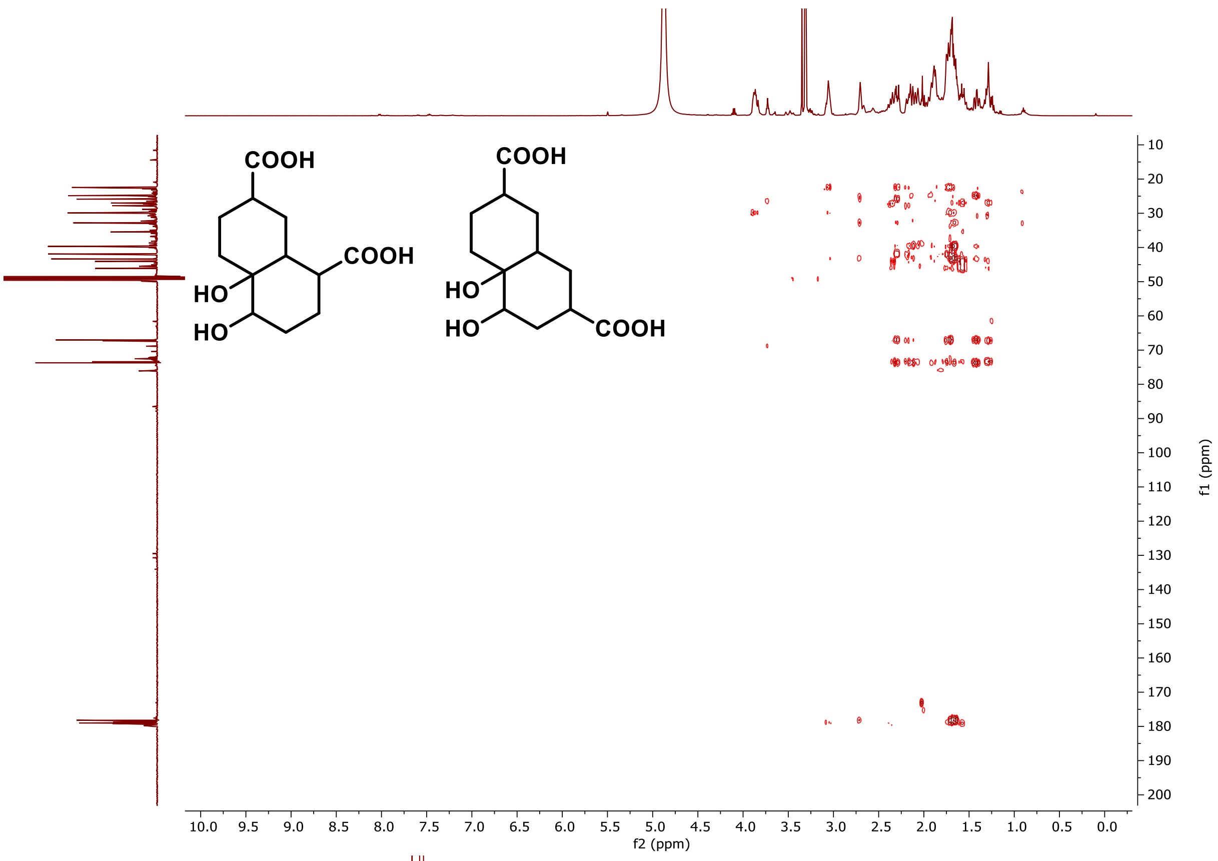
COOH
COOH
HO
HO
COOH
HO
HO
COOH
f2 (ppm)
f1 (ppm)

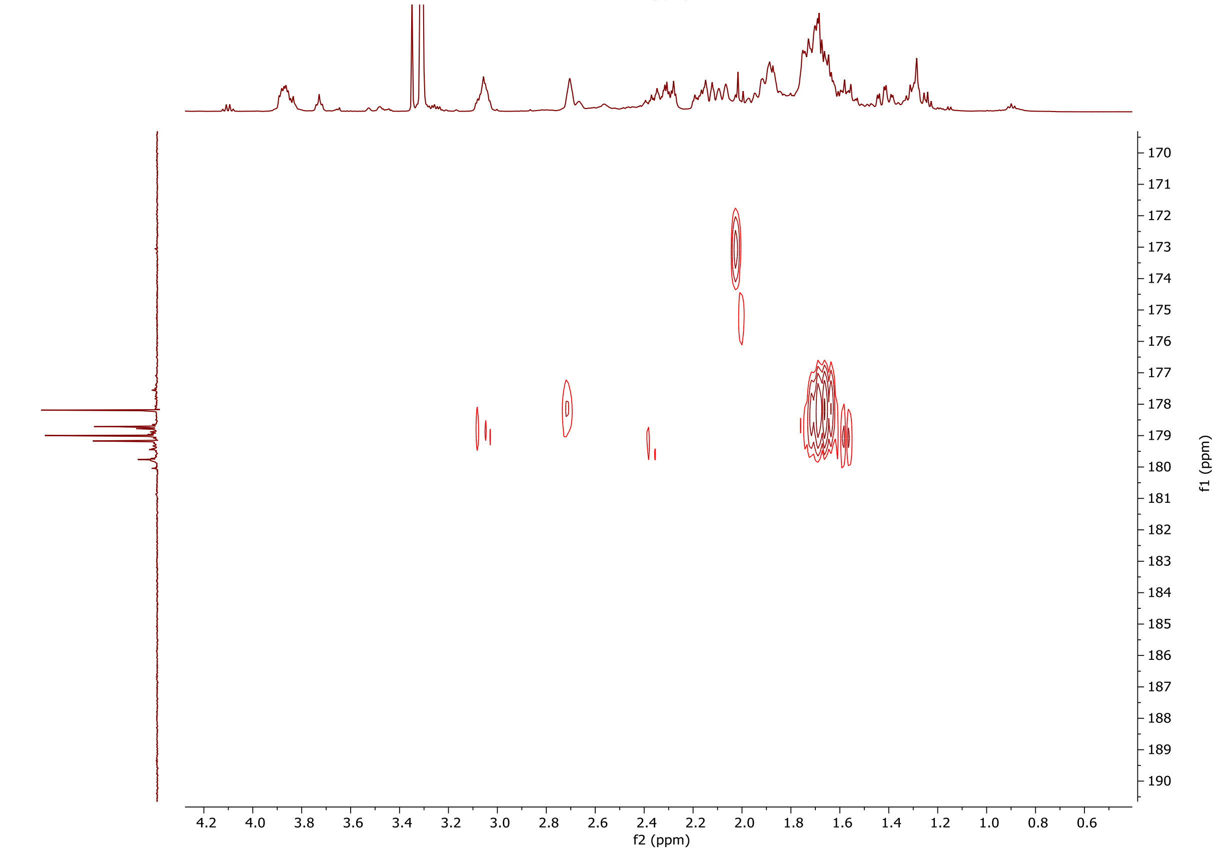
f2 (ppm)
f1 (ppm)

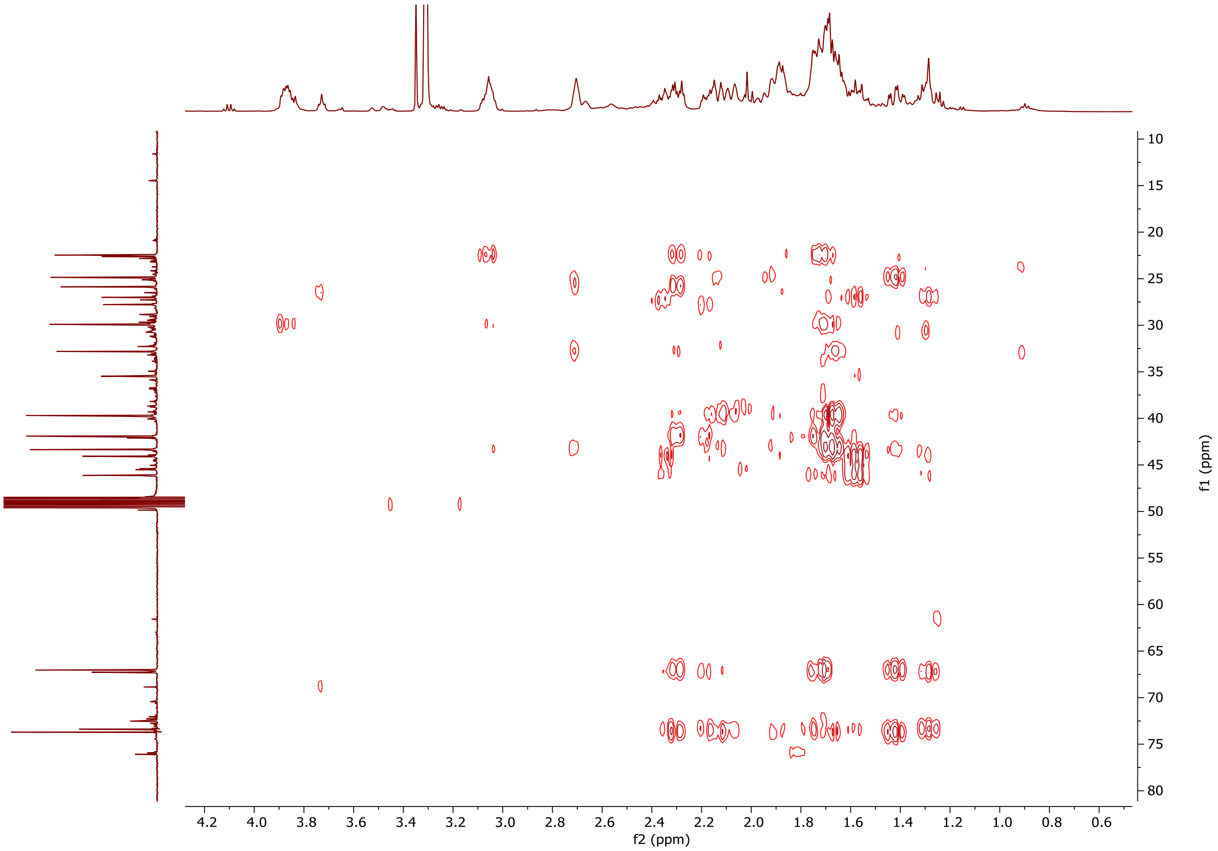

Figure S145: *HMBC spectra of CRAM diacid diol* ***14****.*

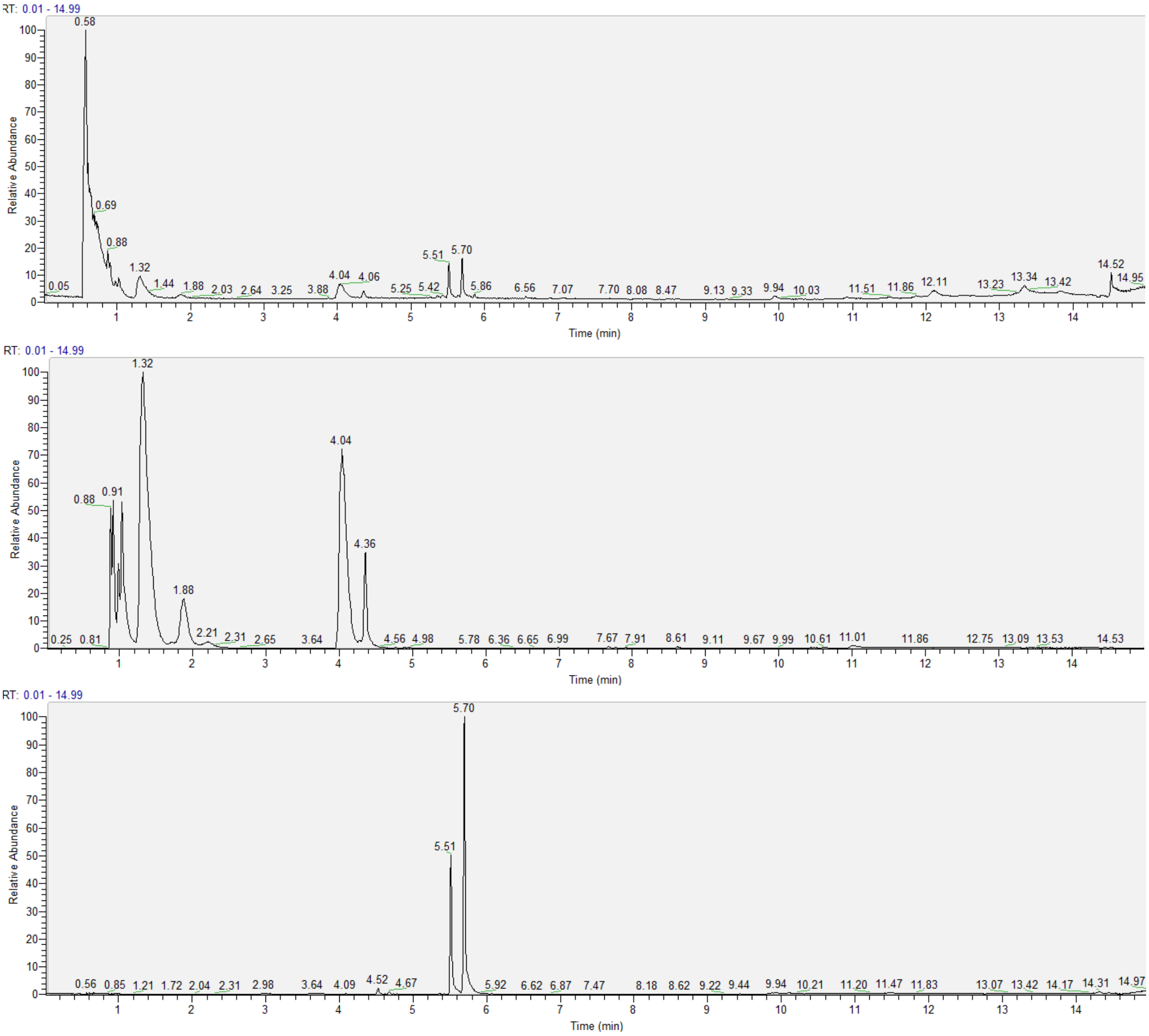

Figure S146: *TIC trace (top), XIC of diacids* ***15a*** *and* ***15b*** *(middle), and XIC of esters* ***16a*** *and* ***16b*** *(bottom).*

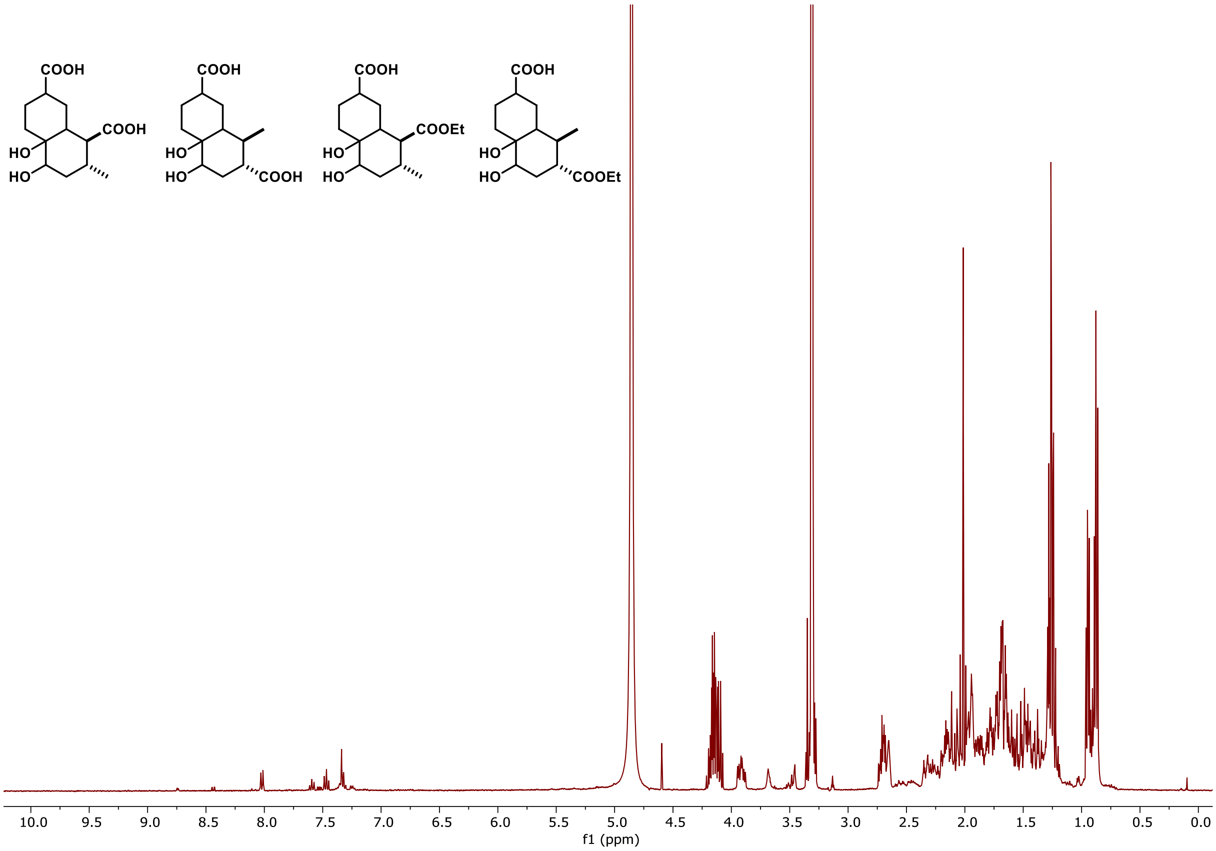

Figure S147: *Crude* ^{1}*H NMR spectra of di-acids* ***15a*** *and* ***15b*** *and mono-esters* ***16a and 16b*** *(400 MHz,* *$CDCl_3$).*

Bibliography

(1) Craig, A. J.; Moodie, L. W.; Hawkes, J. A. Preparation of Simple Bicyclic Carboxylate-Rich Alicyclic Molecules for the Investigation of Dissolved Organic Matter. *Environ. Sci. Technol.* **2024**, *58* (16), 7078–7086.

(2) Camelio, A. M.; Liang, Y.; Eliasen, A. M.; Johnson, T. C.; Yuan, C.; Schuppe, A. W.; Houk, K.; Siegel, D. Computational and Experimental Studies of Phthaloyl Peroxide-Mediated Hydroxylation of Arenes Yield a More Reactive Derivative, 4, 5-Dichlorophthaloyl Peroxide. *J. Org. Chem.* **2015**, *80* (16), 8084–8095.

(3) Wang, J.; Xiang, C.; Luo, F.; Zeng, L.; Zhang, C.; Zhang, J.; Zhu, H.; He, R.; Shao, J. Synthesis of 1, 1, 3-Polyfunctionalized Cyclobutane Derivatives from the Reaction of Sulfur Ylides with Bicyclo [1.1. 0] Butanes. *Org. Lett.* **2024**, *26* (36), 7525–7529.
